# Developing core marker sets for effective genomic-assisted selection in wheat and barley breeding programs

**DOI:** 10.1270/jsbbs.22004

**Published:** 2022-06-29

**Authors:** Goro Ishikawa, Hiroaki Sakai, Nobuyuki Mizuno, Elena Solovieva, Tsuyoshi Tanaka, Kazuki Matsubara

**Affiliations:** 1 Institute of Crop Science, National Agriculture and Food Research Organization, 2-1-2 Kannondai, Tsukuba, Ibaraki 305-8518, Japan; 2 Research Center for Advanced Analysis, Core Technology Research Headquarters, National Agriculture and Food Research Organization, 3-1-1 Kannondai, Tsukuba, Ibaraki 305-8517, Japan

**Keywords:** wheat, barley, breeding, genotyping platform, next-generation sequencing

## Abstract

Wheat (*Triticum aestivum* L.) and barley (*Hordeum vulgare* L.) are widely cultivated temperate crops. In breeding programs with these crops in Japan, effective genomic-assisted selection was performed by selecting core marker sets from thousands of genome-wide amplicon sequencing markers. The core sets consist of 768 and 960 markers for barley and wheat, respectively. These markers are distributed evenly across the genomes and effectively detect widely distributed polymorphisms in the chromosomes. The core set utility was assessed using 1,032 barley and 1,798 wheat accessions across the country. Minor allele frequency and chromosomal distributions showed that the core sets could effectively capture polymorphisms across the entire genome, indicating that the core sets are applicable to highly-related advanced breeding materials. Using the core sets, we also assessed the trait value predictability. As observed via fivefold cross-validation, the prediction accuracies of six barley traits ranged from 0.56–0.74 and 0.62 on average, and the corresponding values for eight wheat traits ranged from 0.44–0.83 and 0.65 on average. These data indicate that the established core marker sets enable breeding processes to be accelerated in a cost-effective manner and provide a strong foundation for further research on genomic selection in crops.

## Introduction

Wheat (*Triticum aestivum* L.) and barley (*Hordeum vulgare* L.) are both important temperate crops and are cultivated across wide areas globally. In 2019, wheat and barley cultivation covered 215.9 million hectares (Mha) and 51.1 Mha globally and they were the first and fifth largest crops in the world, respectively (FAOSTAT, https://www.fao.org/faostat/en/#home). In Japan, both grains were also important for land-use agriculture and for second crops in paddy fields. Processed products made from grains are deeply connected with Japanese food culture, and demands for domestic products have been increasing. Varieties that can adapt to various climate conditions and satisfy the quality requirements of processors and consumers have been developed in breeding programs with these crops across the country. To enhance the speed and efficiency of breeding in Japan, a ‘smart breeding system’, which is a decision-support system in breeding that connects various data, such as genotype, phenotype, pedigree and environmental conditions, was launched in 2017.

Advances in genotyping technologies facilitate genome-wide analysis, such as whole genome QTL analysis and genome-wide association studies (GWASs). In wheat and barley, array-based platforms have been established, and many studies on genetic diversity and GWAS for important traits have been performed using these platforms (Bayer *et al.* 2017, Wang *et al.* 2014). An array-based method has exhibited high accessibility and reproducibility; however, its cost is not suitable for routine use in breeding programs. Next-generation sequencing technologies have paved the way to generate a new concept called genotyping-by-sequencing (Poland *et al.* 2012). In this method, multiple indexed samples were sequenced once, and genotypes were defined based on the sequence polymorphisms among the samples. Since the method is relatively cost-effective, thousands of germplasms have been genotyped thus far (Thudi *et al.* 2021). However, since barley and wheat have large genome sizes, it is important to condense the representatives for sequencing to obtain effective read coverages when several hundred samples are sequenced simultaneously. Several new representation methods have been developed, such as MIG-seq and GRAS-Di (Miki *et al.* 2020, Suyama and Matsuki 2015). These methods are suitable for obtaining genotypic information using materials without prior polymorphic information and highly diverse materials.

Pangenome analyses of barley and wheat revealed high degrees of sequence polymorphisms and structural diversities among global accessions (Jayakodi *et al.* 2020, Walkowiak *et al.* 2020). The Japanese varieties are somewhat genetically separated from those of major producing countries because of geographic aspects (these varieties are far east from their origins) and climatic aspects (Japan has a relatively high temperature and high humidity). A limited number of genetic resources that are highly adapted to the Japanese climate have been introduced from East Asia and repeatedly used for breeding. Therefore, published polymorphic information among global accessions was insufficient to genetically analyze our breeding materials. In a previous study, we obtained polymorphic information from leading Japanese varieties and developed amplicon sequencing markers for genetic analysis and marker-assisted selection (Ishikawa *et al.* 2018, Tanaka *et al.* 2019). For wheat, our preliminary studies indicated that markers developed from our own materials showed higher polymorphic frequencies among breeding materials than from the established array-based markers (9K iSelect, Cavanagh *et al.* 2013), which were designed based on polymorphisms in unrelated materials.

In breeding programs, it is quite important for genome-wide genotyping platforms to be routinely adopted at low cost. In our previous study, a selected marker set customized for each segregating population could effectively construct linkage maps with high genome coverage (Ishikawa *et al.* 2020). Therefore, constructing marker sets that capture a high degree of polymorphic information with a small number of markers would be a feasible solution. Using a marker set, cost-effective and robust strategies involving amplicon sequencing of multiplex samples can be established. The method would enable us to perform marker-assisted selection (MAS) for a moderate number of markers in a short period of time. Recently, genomics-assisted selection was reported to be potentially useful for improving complex traits involving a large number of genes, such as those that affect yield and end-use quality (Varshney *et al.* 2021). Therefore, the objective of this study was to construct core marker sets for barley and wheat breeding and to validate whether the platforms can capture genetic diversity among breeding materials. Furthermore, we assessed their abilities to predict the values of important traits and discussed their further applications.

## Materials and Methods

### Resources of sequence polymorphisms

For barley, sequence polymorphism data were derived from a previous RNA-seq analysis (Tanaka *et al.* 2019) and barley 50K SNP platform (https://ics.hutton.ac.uk/50k/index.pl). To increase polymorphism information, we obtained genomic sequences using four Japanese varieties, ‘Fiber Snow’, ‘Minorimugi’, ‘Hokuriku kawa 54 (Yukihana Rokujo)’ and ‘Sukai Golden’. Genomic DNA of the four varieties was extracted from young leaves using a DNeasy Plant DNA kit (Qiagen, Hilden, Germany). Approximately 125 Gb of genomic sequences per sample were obtained using DNBSEQ^TM^ (MGI Tech Co., Ltd, China) according to the manufacturer’s instructions. Sequence reads were mapped to the reference sequence (IBSC v2; https://plants.ensembl.org/Hordeum_vulgare/Info/Index) using BWA-MEM (Li and Durbin 2009) with the “-M” option. For each variety, a genomic VCF (gVCF) file was created by GATK HaplotypeCaller with the “--emit-ref-confidence GVCF” option, and the gVCF files of the four varieties were merged and genotyped by GATK CombineGVCFs and GenotypeGVCFs with default settings. Detected sequence variants were filtered by GATK VariantFiltration with “QD < 2.0 || FS > 60.0 || MQ < 40.0 || MQRankSum < –12.5 || ReadPosRankSum < –8.0” parameters. Only biallelic and homozygous polymorphic sites were used for selecting target sites.

For wheat, previously obtained polymorphic information was used for selecting target sites (Ishikawa *et al.* 2018).

### Designing amplicon sequencing primers

For barley, polymorphic sites for designing primers were selected across the genome with more than 10 coverage of sequences. In total, 1,310 primer sets were designed to amplify approximately 150 bp products spanning target polymorphic sites by batch Primer3 software with default parameters (Supplemental Table 1). The designed primers were tested for polymorphic rates using genomic DNA of 70 representative barley varieties across the country (Supplemental Table 2). Based on the results of the test, we selected effective markers for breeding as a core set. Amplicon sequencing primers were also designed for known important genes and tested using the representative varieties mentioned above. Effective primer sets for nine genes were included in the core set (Supplemental Table 3).

For wheat, a core set was extracted from the 3,053 primer sets listed in Ishikawa *et al.* (2020). Markers in the core set were selected based on mapping qualities, polymorphic rates and locations in the genome (Supplemental Table 4).

### Plant materials and DNA extraction

Leading varieties, introducing varieties and advanced breeding lines across the country were used to assess the core marker sets. A total of 1,032 (520 in 2018 and 512 in 2019 harvest year) barley accessions were collected from eight breeding stations in Japan and were subjected to genomic DNA extraction. For wheat, a total of 1,798 (823 in 2018 and 975 in 2019) accessions were collected from eight breeding stations in Japan. Breeding stations that provided materials are listed in Table 1. Approximately 50 mg of young leaves was collected from each accession, and total genomic DNA was extracted in 300 μL extraction buffer containing 1 M KCl, 100 mM Tris-HCl (pH 8.0) and 10 mM ethylenediamine tetraacetic acid (EDTA; pH 8.0). DNA was precipitated with 100 μL isopropanol, washed with 200 μL 70% ethanol, and dissolved in 100 μL sterilized distilled water. Extracted DNA was directly used for the following analysis.

### Genotyping by amplicon sequencing

Tag sequences that were compatible with the fusion primer for the second PCR were added to the 5ʹ terminus of each primer. Primers in the core marker set were mixed in one tube. The reaction mixture for the first PCR contained a total volume of 10 μL consisting of 1 × Platinum^TM^ Multiplex PCR Master Mix (Thermo Fisher Scientific), 3 μL of the primer mix and 1 μL of extracted DNA. The PCR program consisted of a denaturation step at 95°C for 2 min, followed by 32 cycles of 30 s at 95°C, 90 s at 60°C and 30 s at 72°C, and a final extension for 10 min at 72°C. The product from the first PCR of each sample was diluted 100-fold with sterilized distilled water, and 2 μL of the diluted product was used as the template for the second PCR.

The second PCR was performed to add index sequences to the amplicons of each sample. The 10-μL PCR mix contained 1 × Platinum^TM^ Multiplex PCR Master Mix (Thermo Fisher Scientific), 400 nM forward and reverse fusion primers, and 2 μL of the diluted first PCR product. PCR was run using the following steps: 2 min at 95°C, followed by 15 cycles of 30 s at 95°C, 90 s at 60°C and 1 min at 72°C, and a final extension step of 10 min at 72°C. All second PCR products were mixed in equivalent volumes (2 μL of PCR product per sample). The pooled product was purified using Agencourt AMPure XP Reagent beads (Beckman-Coulter, Fullerton, CA, USA) as follows: 12 μL of pooled products, 24 μL of TE buffer and 36 μL of well-mixed AMPure XP beads were vortexed. After a 10-min incubation at room temperature, the sample was placed onto a magnetic separator for 1 min, and the supernatant was discarded. The beads with attached samples were washed twice with 180 μL of freshly prepared 70% ethanol. Finally, the purified PCR products were suspended in 40 μL of low TE buffer. The quality of the amplicon library was assessed using LabChip GX Touch (PerkinElmer), and a DNA 5K kit was used to define the region covering all PCR library peaks (200–250 bp for barley, 350–450 bp for wheat). Sequencing was performed by an Ion GeneStudio S5 (Thermo Fisher Scientific) using a 530 chip for the wheat library and a 540 chip for the barley library. For some wheat samples, library preparation and sequencing on a DNBSEQ^TM^ (MGI Tech Co., Ltd) were performed by a custom amplicon sequencing service (Bioengineering Lab. Co., Ltd., Japan, https://www.gikenbio.com/) following the manufacturer’s instructions. Adapter sequences were removed by Cutadapt (Martin 2011) with default parameters, and low-quality sequences were further trimmed off by Trimmomatic (Bolger *et al.* 2014) with “LEADING:20 TRAILING:20 SLIDINGWINDOW:4:20 MINLEN:30” parameters.

Published reference sequences of barley (IBSC v2; https://plants.ensembl.org/Hordeum_vulgare/Info/Index) and wheat (IWGSC RefSeq v1.0; https://wheat-urgi.versailles.inra.fr/Seq-Repository/Assemblies) were used for mapping sequences from accessions. To reduce the calculation time, reference sequences were reconstructed by connecting sequences of target regions and genes with 1,000 Ns junction spacings. Sequence reads were mapped to the reference sequences by BWA-MEM (Li and Durbin 2009) with the “-M” option, and mapped reads were filtered by Samtools (Li *et al.* 2009) with the “-q 20 -F 0x100” option. For each sample, a gVCF file was created by GATK HaplotypeCaller with “--emit-ref-confidence GVCF --allow-nonunique-kmers-in-ref true” options, and the gVCF files were merged and genotyped by GATK CombineGVCFs and GenotypeGVCFs with default settings. Detected sequence variants were filtered by GATK VariantFiltration with “QD < 5.0 || FS > 30.0 || MQ < 30.0” parameters. The resultant VCF files were used for further analysis.

### Analyses of genotypes

Genotype data were analyzed using TASSEL v.5 (https://www.maizegenetics.net/tassel) (Bradbury *et al.* 2007). Detected variants with both more than 50% missing data and more than 20% heterozygous genotypes were filtered out of all accessions. This filtration yielded as many polymorphic sites as the number of target sites in both wheat and barley. Calculations of the minor allele frequencies, principal component analysis with the covariance matrix, and linkage disequilibrium analysis were performed by the functions implemented in TASSEL v5.

### Genomic prediction of traits

Using phenotypic data collected from eight barley and wheat breeding stations each, genomic predictions of trait values were performed using the ‘rrBLUP’ package of R (Endelman 2011). Before starting the analysis, phenotypic data were scaled by the combining the breeding station and harvest year using the ‘group_by’ and ‘scale’ functions in the ‘dplyr’ package in R. Prediction accuracies were assessed by the two following methods: one was a fivefold cross-validation, and the other was a cross-year prediction. In the fivefold cross-validation, one-fifth of the samples were randomly selected and used as a test set, and the remaining samples were used as a training set. A prediction model constructed by the training set was applied to the test set to predict values. Accuracy was evaluated by the correlation coefficient between the predicted and observed values of the test set. We repeated the procedure 100 times and calculated the mean and standard deviation of the correlation coefficients. In the cross-year prediction, we set samples in 2018 as a training set and predict values of samples in 2019 (2018 > 2019) and vice versa (2019 > 2018).

## Results

### Construction of the core marker sets for barley and wheat breeding

Based on the results of the test samples, amplicon sequencing markers were evaluated in terms of missing rate, mapping quality and minor allele frequency (MAF). Markers with less than 0.3 missing rate were selected. For the mapping quality, markers were categorized into the classes described in Ishikawa *et al.* (2018). Markers in classes 1 to 3, which show no or less interference from off-target or homoeologous sequences, were selected. Markers with high polymorphic rates were preferentially selected. To retain markers across the genomes, the selection criteria of polymorphic rates were changed according to their chromosomal locations. Differences in missing rates and MAF between selected (CoreSet) and unselected (Other) markers are described in Supplemental Fig. 1.

For barley, the number of markers per chromosome ranged from 104 (1H) to 116 (5H), and 768 in total (Table 2). The physical distances between the markers were an average of 6.0 Mb, and the maximum interval was 31.3 Mb on 5H chromosome. Based on the physical positions of terminal markers, coverage of each chromosome achieved more than 99.4% and that of whole reference sequences was 94.6%. For wheat, the number of markers per chromosome ranged from 38 (1D and 4D) to 52 (7A and 6B) and was 960 in total (Table 3). The mean physical interval between markers was 14.9 Mb, and maximum interval was 257.8 Mb on 4A chromosome. The coverages ranged from 97.2% (3A and 5A) to 99.8% on 6D and was 95.5% in total. The genotyping platforms composed of the selected markers were named ‘HvCoreSet_v1’ for barley and ‘TaCoreSet_v1’ for wheat.

### Availability of the core sets for capturing polymorphisms among breeding materials

**Fig. 1.**
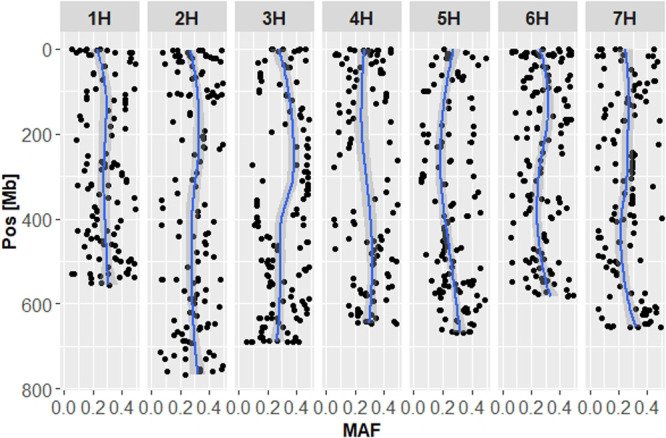
Distribution of minor allele frequencies (MAFs) along with barley chromosomes. MAFs were calculated from genotypes of 1,032 barley accessions obtained by HvCoreSet_v1. Blue lines indicate locally weighted scatterplot smoother (LOESS) curves. Gray area indicates 95% confident interval.

For barley, HvCoreSet_v1 was adopted for 1,032 breeding materials across the country. The distribution of minor allele frequency (MAF) along with the seven chromosomes is depicted in Fig. 1. Locally weighted scatterplot smoother (LOESS) curves of MAF showed that the core set could evenly capture polymorphisms across the entire genome. In each breeding station, however, MAF distributions showed some low polymorphic regions as follows: 1H of TAES and FARC, 2H of TAES, WARC, FARC and KARC, 3H of TAES, WARC and FARC, 4H of NAES, 6H of TARC, NAES and WARC, 7H of WARC (Supplemental Fig. 2). The MAF distributions of NICS were similar to those of all accessions.

For wheat, TaCoreSet_v1 was adopted for 1,798 breeding materials across the country. It was also shown that the core set could capture polymorphisms across the genome (Supplemental Fig. 3). Unlike the barley platform, MAF distributions along 21 chromosomes were similar among breeding stations (Supplemental Fig. 4). However, slightly lower MAF regions compared to those using all accessions were observed in the 7A chromosome in FARC, HARC, KAES, NICS and WARC.

### Genetic diversity among barley and wheat breeding materials

**Fig. 2.**
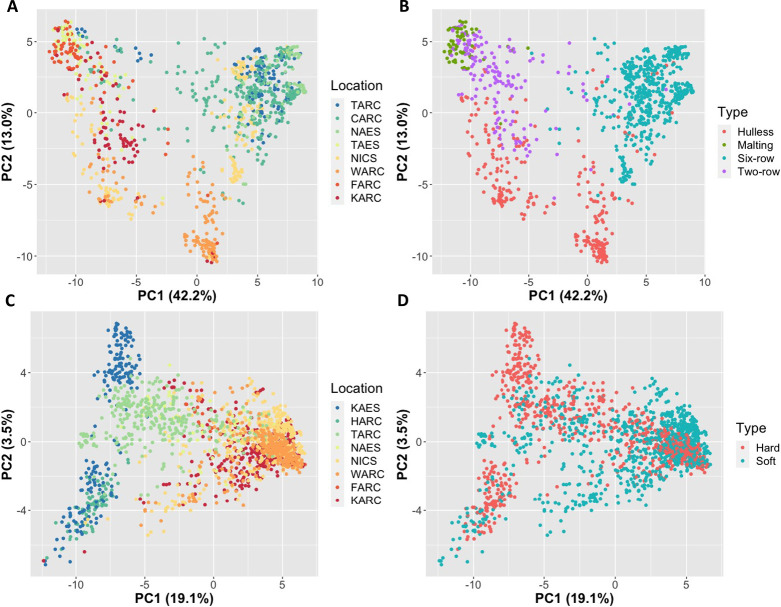
Scatter diagrams of PC1 and PC2 derived from principal component analysis using genotypes obtained by HvCoreSet_v1 and TaCoreSet_v1. Diagrams of 1,032 barley accessions grouped by breeding stations (A) and market types (hulless, malting, six-row and two-row) (B). Diagrams of 1,798 wheat accessions grouped by breeding stations (C) and kernel types (soft and hard) (D).

To investigate genetic diversity among accessions, we performed principal component analysis (PCA) using a covariance matrix. As a result of barley, PC1, PC2 and PC3 explained 42.2, 13.0 and 6.4% of the variation, respectively. A scatter diagram of PC1 and PC2 showed three main clusters: Cluster 1; high PC1 and high PC2, Cluster 2; low PC1 and high PC2, Cluster 3; middle PC1 and low PC2 (Fig. 2A). Based on the locations of the breeding stations, it is assumed that PC1 explained genetic differences along with latitude, and PC2 seems to distinguish accessions of WARC from others. When the accessions were classified based on barley market types (hulless, malting, six-row and two-row types), it was revealed that the clusters clearly represented these types that affect end-use properties (Fig. 2B).

For wheat, PCA of all accessions revealed that PC1, PC2 and PC3 explained 19.1, 3.5 and 3.2% of the variation, respectively. Scatter diagrams of PC1 and PC2 exhibited a continuous distribution, but to some extent, clusters were formed by accessions of each breeding station (Fig. 2C). TARC accessions showed the largest variation among the breeding stations. Since high PC2 accessions of KAES were classified into spring type (no vernalization requirement), it is assumed that PC2 captured genetic differences between spring and winter type. Unlike the case of barley, kernel type that affected end-use properties did not show clear clusters except for hard spring accessions in KAES (Fig. 2D).

**Fig. 3.**
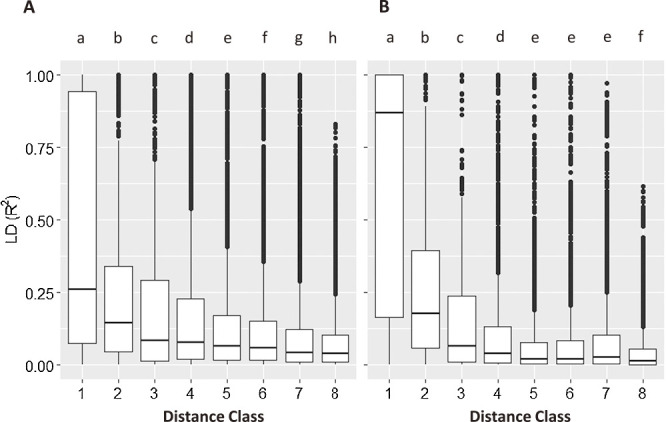
Linkage disequilibrium (LD) decay across the barley and wheat genomes. LD was compared by the degree of r^2^ values among eight classes of physical distances of barley (A) and wheat (B). Different characters on each plot indicate significant differences among distance classes at a level of 0.05 assessed via the Tukey HSD function in R. Distance 1: <1 Mb, 2: 1–5 Mb, 3: 5–10 Mb, 4: 10–50 Mb, 5: 50–100 Mb, 6: 100–200 Mb, 7: 200–400 Mb, 8: >400 Mb.

To evaluate historical recombination events in the breeding programs, we performed linkage disequilibrium (LD) analysis using all accessions. r^2^ values were compared with the eight classes based on the physical distances between polymorphic sites. Since intra-chromosomal LD decay analysis indicated the threshold r^2^ = 0.1 is in the 90th and 90th percentiles for wheat and barley, this cutoff was considered the minimum threshold for a significant association between pairs of polymorphic sites (Yadav *et al.* 2021). In both barley and wheat, high LD (median r^2^ > 0.1) was observed in less than 5 Mb distance classes (Fig. 3). In the more than 5 Mb distance classes, high r^2^ values were retained to over 400 Mb in barley, while r^2^ values were settled down more than 50 Mb distance classes in wheat. The degree of LD decay was different among chromosomes; for example, chromosomes 3H, 5H and 7H in barley tended to maintain high LD at approximately 10 Mb (Supplemental Fig. 5).

### Prediction of trait values using the core marker sets

The ability to predict trait values would be the most desired application of genome-wide genotype data in breeding programs. Therefore, using preliminary data from 2018 and 2019 harvest years, we assessed prediction accuracies using the developed genotyping platforms.

**Fig. 4.**
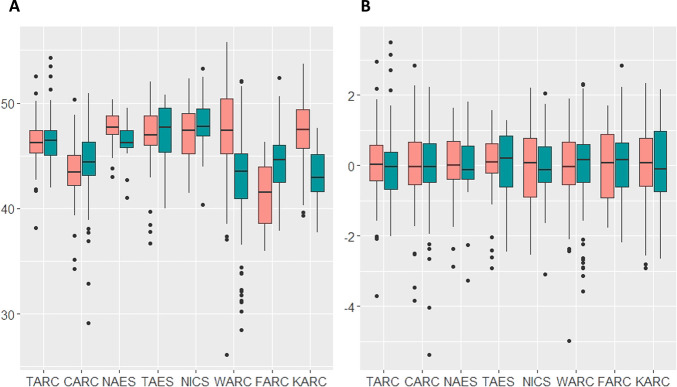
Phenotypic distributions of 1,707 barley entries with the combinations of breeding stations and harvest years. Distributions of whiteness of pearled grain values (A) and those of scaled values by each combination (B). Red and blue boxes indicate data of samples harvested in 2018 and 2019, respectively. All six traits are described in Supplemental Fig. 6.

In barley, we selected the following traits as examples: days to heading after sowing (DH), test weight (TestW), thousand grain weight (TGW), grain protein content (GPC), glassy kernel rate (GlassR) and whiteness of pearled grain (White). The distribution of these traits was different among breeding stations due to the differences in growth conditions, evaluation procedures and equipment (Fig. 4A, Supplemental Fig. 6). Therefore, to adjust these differences, trait values were scaled by combining the breeding station and harvest year (Fig. 4B, Supplemental Fig. 6). Using the scaled values, prediction accuracies were calculated by fivefold cross-validations and cross-year predictions. The prediction accuracies of traits ranged from 0.551 for DH to 0.739 for TGW by cross-validation (Table 4). For the cross-year prediction, TGW showed the highest (0.555) among the traits investigated, while GlassR was the lowest (0.276).

Eight traits, DH, TestW, TGW, GPC, flour yield (FlYd), flour color L* (FlL), flour color a* (Fla), and flour color b* (Flb) were used to assess prediction accuracies in wheat (Supplemental Fig. 7). As the result of cross-validation, Flb, FlYd, GPC and Fla exhibited high prediction accuracies (>0.7), TGW and FlL were middle (approximately 0.6), and DH and TestW had relatively low prediction accuracies (approximately 0.4) (Table 4). Predictabilities between years drastically declined for DH and TestW, while Flb, FlYd and GPC maintained high (>0.5) cross-year prediction accuracies.

## Discussion

Chromosome-level reference sequences have been available in both barley and wheat (Appels *et al.* 2018, Mascher *et al.* 2017). Using this information, a small number of polymorphic sites that effectively evaluate genome-wide diversity among samples of interest can be selected. In this study, we constructed core marker sets that consisted of 768 and 960 markers for barley and wheat, respectively. Although the numbers of markers were smaller compared to that of other genome-wide genotyping systems, the mean marker intervals were 6 Mb for barley and 15 Mb for wheat, and the genome coverages were approximately 95% in both barley and wheat (Tables 2, 3). The MAF distributions along with chromosomes indicated that these marker sets were useful for capturing polymorphisms without any regional bias (Fig. 1, Supplemental Fig. 3). Recently, low-cost genotyping platforms based on random amplicon sequencing (GRAS-Di) have been developed (Enoki and Takeuchi 2018). This method has merit when materials without any prior information are utilized; however, it is difficult to control the polymorphic rate and distribution. When we used genetically close materials such as advanced breeding lines within a breeding station, more sequence data per sample were needed to obtain sufficient polymorphisms across the genome due to the low polymorphic rate in randomly amplified products (data not shown). Therefore, polymorphisms can be achieved only by our targeted sequencing strategy that obtains sufficient polymorphic data among breeding materials with a limited number of reads per sample.

The PCA of barley revealed that there are well-described genetic relationships among breeding materials with respect to breeding stations and end-use properties (Fig. 2A, 2B). PC1 seems to explain the variation in the genetic background conferring photoperiod sensitivity, because the axis values relate to the latitude of breeding stations. In addition, the distinct cluster of WARC by the PC2 axis is due to hulless accessions of the breeding station. This is the first comprehensive diversity analysis of barley breeding materials in Japan. It was revealed that barley breeding materials were genetically differentiated by end-use properties. The MAF distributions along with chromosomes also revealed that quite low polymorphic regions existed within accessions in a breeding station (Supplemental Fig. 2). In addition, when compared with wheat, LD analysis revealed that relatively large chromosomal blocks had been inherited in barley breeding history (Fig. 3). Therefore, it is necessary to improve genetic gain to introduce various germplasms and to enhance recombination frequency. Wheat accessions were roughly categorized into northern accessions (HARC and KAES), northeastern accessions (TARC and NAES) and western accessions (NICS, WARC, FARC, KARC) (Fig. 2C). The genetic diversity of wheat was coincident with previously reported relationships among materials (Ishikawa *et al.* 2014, Kobayashi *et al.* 2016). Accessions of TARC showed the highest genetic diversity among those of other stations and were distributed between northern and western accessions. The high genetic diversity of the TARC breeding materials is thought to be due to progenies derived from the cross between northern and western accessions. The hard spring type of KAES formed a unique cluster in the PC1 and PC2 scatter diagrams (Fig. 2C, 2D). This indicates that winter lines were rarely used for spring wheat breeding as parents and vice versa. Genetic dissections about low temperature requirements have been conducted, and some main effector genes, such as *Vrn-1* and *Vrn-3* homoeologous genes, have been isolated (Yan *et al.* 2004, 2006). These data would be useful for selecting candidates from populations between spring and winter accessions. Therefore, it is important to enhance the genetic exchange of materials with different vernalization requirements.

Annually, various phenotypic data based on a common format were collected at each breeding station across the country. To investigate whether the core sets developed in this study can be applied to predict trait values from different breeding stations, we assessed prediction accuracies with the two methods. Prediction accuracies varied among the traits, and low prediction accuracies (less than 0.3) in the cross-year prediction were observed in GlassR of barley and DH and TestW of wheat (Table 4). However, there were traits with more than 0.7 accuracies by cross-validation. Genomic prediction for end-use quality traits in soft white winter wheat has recently been reported (Aoun *et al.* 2021). In this paper, approximately 0.6 prediction accuracies were achieved using a high-density genotyping platform, and the authors concluded that the accuracies were sufficient to justify implementing genomic selection in breeding. Prediction accuracy was affected by various factors, such as population structure, size of training population, heritability of traits and marker density. Among them, Plavsin *et al.* (2021) reported that the number of markers had a minimal impact on genomic selection accuracy, suggesting that when genomic resolution is reached in a high LD species (i.e., wheat), marker density no longer represents a limiting factor. In this preliminary study, the applicability of the core marker sets for genomic selection in breeding programs across the country was revealed. The level of accuracy required for practical use in breeding cannot be simply stated, because it involves a balance among genotyping cost, time and effort. However, when a cost-effective genotyping method becomes available, there are breeders who will want to introduce genomic selection into their breeding programs, especially for selecting yield and quality traits that are difficult to evaluate. In these cases, even if the accuracy is not high, it will be beneficial for initial screening of many candidates (personal communication).

To date, due to the cost of genotyping, a genome-wide genotyping platform has mainly been applied to diversity and/or genetic analyses using established varieties and lines. However, the core marker sets developed in this study achieve a genome-wide survey during the breeding process. This includes several important changes from traditional breeding schemes. One application is background selection during the development of introgression lines. This method reduces the number of backcross processes and shortens the time for developing lines. Genome-wide surveys during the process of breeding also facilitate the creation of novel allele combinations. We can find rare recombination events in suppression regions along chromosomes that provide valuable information for selecting individuals. The other is a haplotype analysis. Haplotype analysis combining several polymorphisms may improve the accuracy of genomic prediction due to better capture of linkage disequilibrium and genomic similarity (Sallam *et al.* 2020). By sequencing amplicons, polymorphisms can be detected in the flanking region of the target site. Using these polymorphisms, multiallelic analysis can be performed instead of single-SNP analysis, which may be incapable of capturing underlying allelic diversity. In this study, we found that 165 out of 768 (21.5%) barley markers and 189 out of 960 (19.7%) wheat markers contained polymorphisms other than the target site. These markers are more informative to assess genetic diversity among breeding materials than those representing a single polymorphic site.

Genome-wide genotyping platforms have been well used for genetic dissection analyses such as genome-wide association studies (GWASs) (Liu and Yan 2019). The greater the number of markers is, the greater the chance of detecting marker-trait associations by GWAS. The platforms developed in this study are not suitable for performing GWAS because the number of markers is limited to keep costs down. However, using genotypes derived from the core sets, we found some marker-trait associations by GWAS with a mixed linear model considering population structure and kinship (data not shown). The *fra* and *wax* genes of barley are known as genetic factors related to glassy kernel rate (GlassR), and their diagnostic markers have been developed (Fujita *et al.* 1999, Saito *et al.* 2018). When adding genotypes of these markers into those of the core set, significant marker-trait associations between the genes and GlassR could be detected. This result indicates that adding markers associated with traits into the core sets is beneficial for increasing the power of GWAS as well as genomic prediction. Advances in sequencing technologies will allow us to obtain much data at a low cost. It is expected that information about genes and/or regions related to important traits will steadily accumulate. Therefore, adding markers that relate to traits of interest and contain multiple polymorphisms will be the direction for updating the core marker sets.

Data-driven breeding will be established near the future incorporating trait values, pedigree information and genomic information with FAIR (findable, accessible, interoperable, and reusable) norms. The genotyping platforms described in this study will be a model for large genome species in which whole genome sequencing of multiple candidates in breeding stations is unrealistic.

## Author Contribution Statement

GI designed the study, collected the data and wrote the manuscript. HS analyzed the amplicon sequencing data and wrote the manuscript. NM and TT analyzed barley genome data. ES constructed reference sequences for analysis of target amplicon sequencing data. KM performed sample preparation for genotyping. All authors read and approved the final manuscript.

## Supplementary Material

Supplemental Figures

Supplemental Tables

## Figures and Tables

**Table 1.** List of barley and wheat breeding stations across the country

Abbriviation	Name	Barley	Wheat	Location	
				Latitude	Longitude
KAES	Kitami Agricultural Experiment Station, HRO		✓	43.75	143.72
HARC	Hokkaido Agricultural Research Center, NARO		✓	42.90	143.07
TARC	Tohoku Agricultural Research Center, NARO	✓	✓	39.76	141.14
CARC	Central Region Agricultural Research Center, NARO	✓		37.12	138.27
NAES	Nagano Prefecture Agricultural Experiment Station	✓	✓	36.66	138.29
TAES	Tochigi Prefectural Agricultural Experiment Station	✓		36.61	139.86
NICS	Institute of Crop Science, NARO	✓	✓	36.01	140.04
WARC	Western Region Agricultural Research Center, NARO		✓	34.50	133.39
		✓		34.23	133.78
FARC	Fukuoka Agriculture and Forestry Research Center	✓	✓	33.50	130.57
KARC	Kyushu-Okinawa Agricultural Research Center, NARO	✓	✓	33.21	130.49

HRO: Hokkaido Research Organization, Agricultural Research Department. NARO: National Agriculture and Food Research Organization.

**Table 2.** Summary of the core marker set for barley breeding (HvCoreSet_v1)

Chr	No. of marker	Marker interval [Mb]			Position [Mb]		Cover size [Mb]	RefSeq size^*a*^ [Mb]	Coverage [%]
		Max	Mean		Start	End			
1H	104	20.1	5.4		1.4	556.5	555.1	558.5	99.4
2H	109	28.0	7.1		2.3	766.3	764.0	768.1	99.5
3H	110	21.0	6.4		0.1	699.1	699.0	699.7	99.9
4H	109	29.5	6.0		0.1	646.0	645.9	647.1	99.8
5H	116	31.3	5.8		0.4	669.6	669.2	670.0	99.9
6H	115	30.2	5.1		0.3	582.4	582.1	583.4	99.8
7H	105	27.0	6.3		0.6	656.1	655.5	657.2	99.7
Un	0							249.8	
Total	768	31.3	6.0				4570.8	4833.8	94.6

Un: Unknown. ^*a*^ IBSC v2 (https://plants.ensembl.org/Hordeum_vulgare/Info/Index).

**Table 3.** Summary of the core marker set for wheat breeding (TaCoreSet_v1)

Chr	No. of marker	Marker interval [Mb]			Position [Mb]		Cover size [Mb]	RefSeq size^*a*^ [Mb]	Coverage [%]
		Max	Mean		Start	End			
1A	47	65.9	12.8		3.4	591.2	587.8	594.1	98.9
2A	51	109.8	15.5		2.5	778.4	775.9	780.8	99.4
3A	42	115.3	17.8		13.9	743.8	729.9	750.8	97.2
4A	46	257.8	16.4		2.9	742.4	739.5	744.6	99.3
5A	49	112.6	14.4		0.3	689.9	689.6	709.8	97.2
6A	39	206.1	16.2		0.6	615.5	614.9	618.1	99.5
7A	52	174.3	14.2		8.4	733.4	725.0	736.7	98.4
1B	43	82.1	16.3		4.3	687.7	683.4	689.9	99.1
2B	51	116.9	15.7		4.6	789.9	785.3	801.3	98.0
3B	51	56.3	16.4		0.2	822.2	822.0	830.8	98.9
4B	41	180.8	16.7		1.8	670.4	668.6	673.6	99.3
5B	47	105.6	15.3		7.7	712.7	705.0	713.1	98.9
6B	52	47.1	14.1		0.2	718.9	718.7	721.0	99.7
7B	44	105.9	16.9		2.9	730.2	727.3	750.6	96.9
1D	38	132.7	13.3		0.9	494.2	493.3	495.5	99.6
2D	49	87.6	13.5		0.4	648.5	648.1	651.9	99.4
3D	40	84.0	15.6		5.0	613.4	608.4	615.6	98.8
4D	38	98.1	13.6		1.3	506.1	504.8	509.9	99.0
5D	50	70.8	11.5		0.6	562.7	562.1	566.1	99.3
6D	43	124.1	11.3		0.8	473.4	472.6	473.6	99.8
7D	39	112.8	16.6		1.3	631.8	630.5	638.7	98.7
Un	8							481.0	
Total	960	257.8	14.9				13892.7	14547.5	95.5

Un: Unknown. ^*a*^ IWGSC Ref Seq v1.0 (https://wheat-urgi.versailles.inra.fr/Seq-Repository/Assemblies).

**Table 4.** Prediction accuracies of target traits obtained using two different methods

Species	Trait	Cross validation			Cross-year		
		Mean	S.D.		2018 > 2019	2019 > 2018	Mean
Barley	DH	0.5551	0.0374		0.3870	0.3341	0.3606
	TestW	0.6162	0.0426		0.4223	0.4337	0.4280
	TGW	0.7390	0.0400		0.4843	0.6259	0.5551
	GPC	0.5624	0.0514		0.3964	0.4349	0.4157
	GlassR	0.5585	0.0467		0.2603	0.2915	0.2759
	White	0.6642	0.0308		0.4821	0.4432	0.4626
Wheat	DH	0.4394	0.0332		0.1793	0.1897	0.1845
	TestW	0.4425	0.0445		0.2734	0.2580	0.2657
	TGW	0.6082	0.0347		0.3810	0.2990	0.3400
	GPC	0.7330	0.0208		0.6227	0.5397	0.5812
	FlYd	0.7370	0.0456		0.5281	0.5247	0.5264
	FlL	0.6677	0.0557		0.3740	0.4087	0.3914
	Fla	0.7212	0.0503		0.3953	0.3480	0.3716
	Flb	0.8292	0.0288		0.6955	0.6425	0.6690

DH: Days to heading after sowing; TestW: Test weight; TGW: Thousand grain weight; GPC: Grain protein content; GlassR: Glassy kernel rate; White: Whiteness of pearled grain; FlYd: Flour yield; FlL: Flour color L*; Fla: Flour color a*; Flb: Flour color b*.
