# Supplementary material for: Developing core marker sets for effective genomic-assisted selection in wheat and barley breeding programs
Source: Breed Sci. 2022 Jun 29;72(3):257–66. doi: 10.1270/jsbbs.22004 (PMC9653188; doi:10.1270/jsbbs.22004)
Supplement: Supplementary file 1 — Supplemental Figures [file 72_257_s1.pdf]

doi: 10.1270/jsbbs.22004

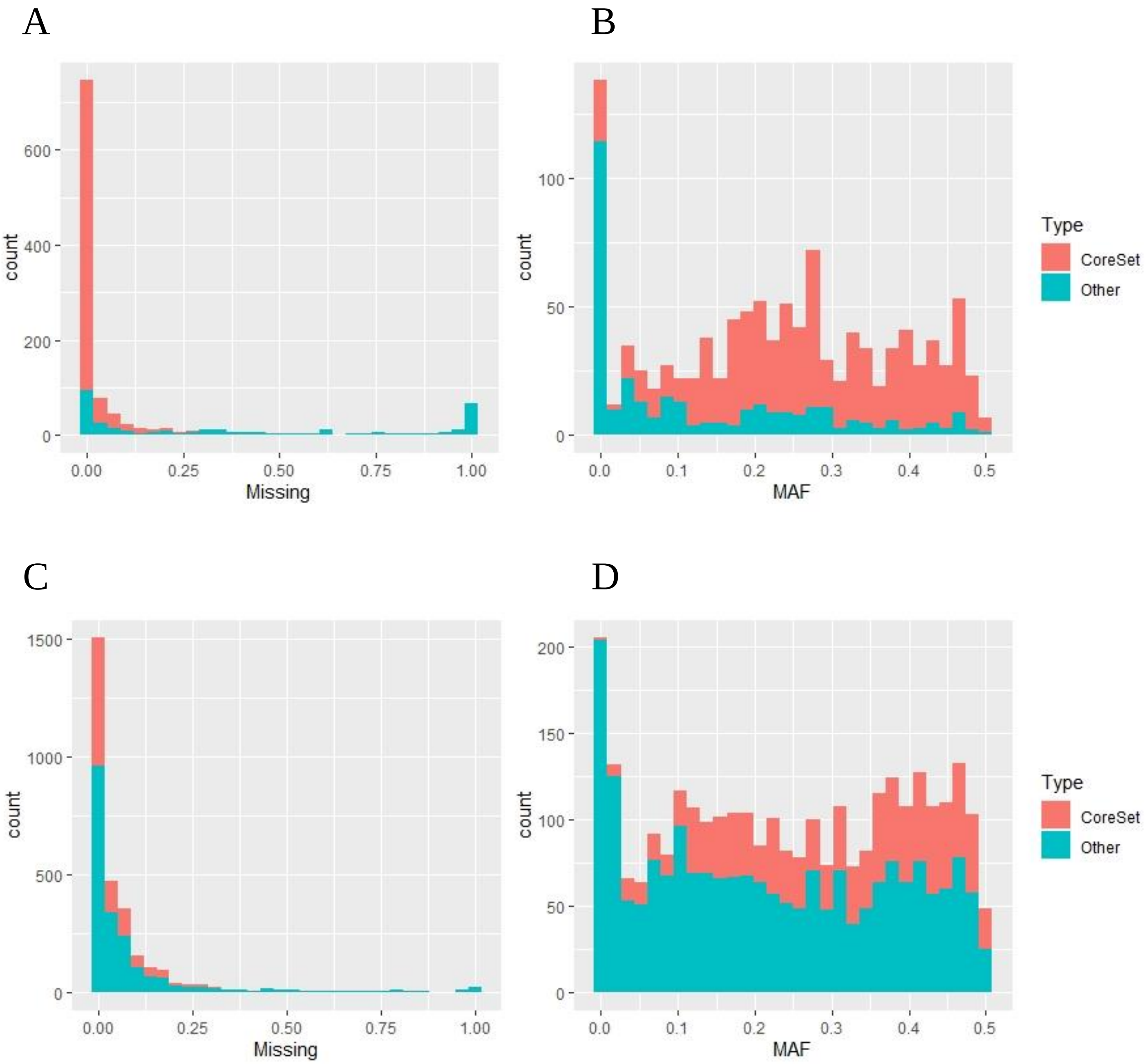

Supplemental Fig. 1. Distribution differences in the missing rates and minor allele frequencies (MAFs) between selected (CoreSet) and unselected (Other) markers. A and B: Missing rates and MAF distribution of barley, respectively. C and D: Missing rates and MAF distribution of wheat, respectively.

A

B

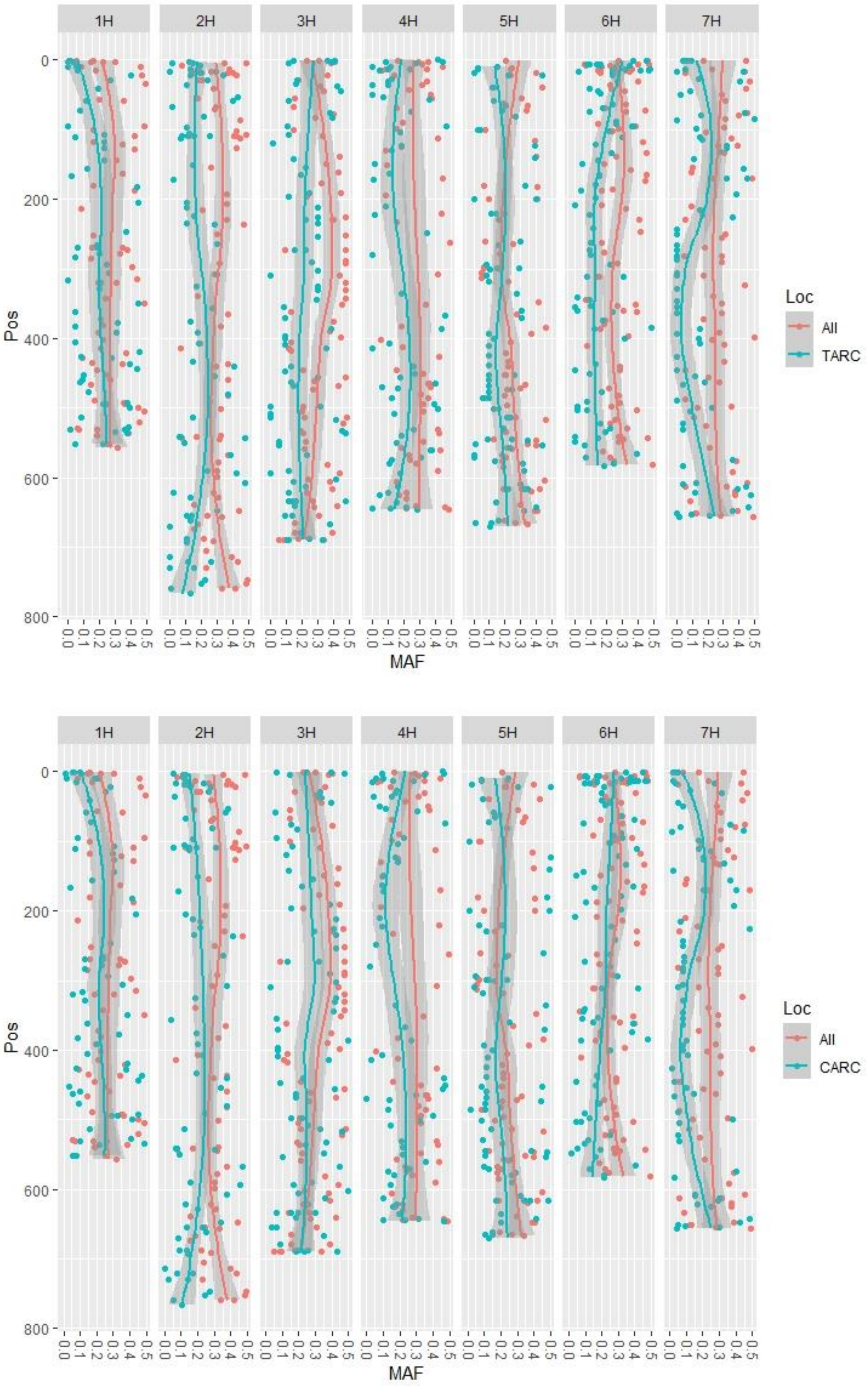

1H
2H
3H
4H
5H
6H
7H
Pos
MAF
0
200
400
600
800
0.0 0.1 0.2 0.3 0.4 0.5
Loc
All
TARC
CARC

C

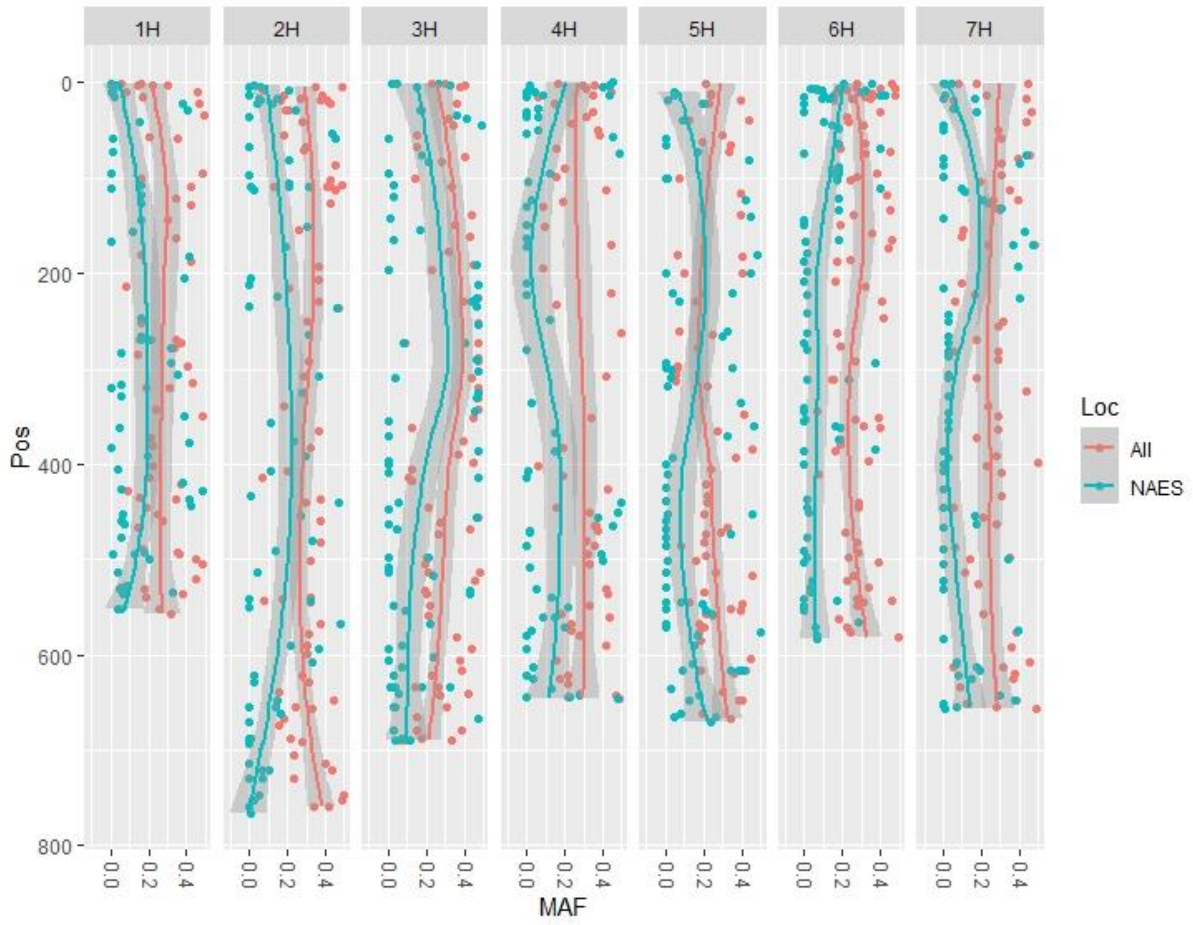
1H
2H
3H
4H
5H
6H
7H
Pos
MAF
0
200
400
600
800
0.0
0.2
0.4
Loc
All
NAES

D

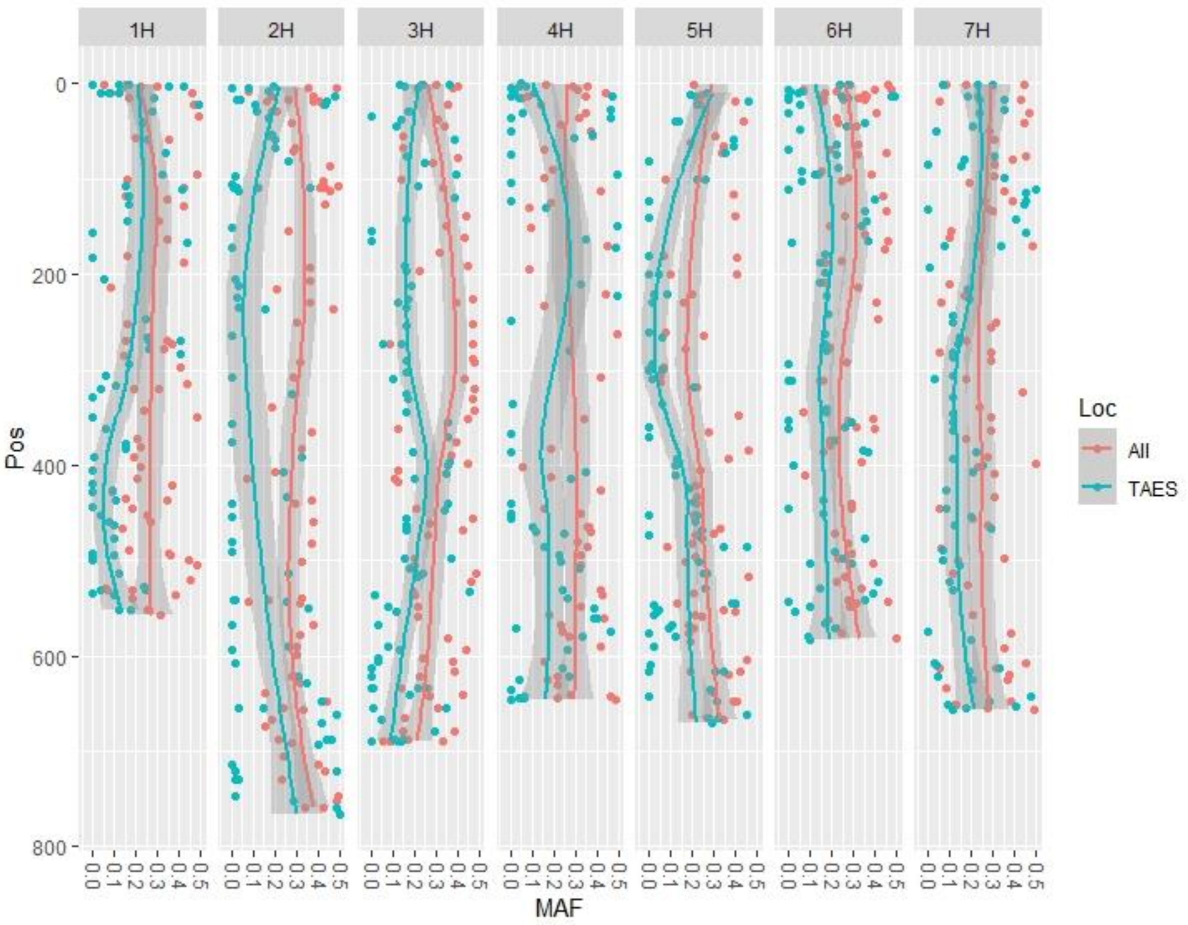
1H
2H
3H
4H
5H
6H
7H
Pos
MAF
0
200
400
600
800
0.0
0.1
0.2
0.3
0.4
0.5
Loc
All
TAES

E

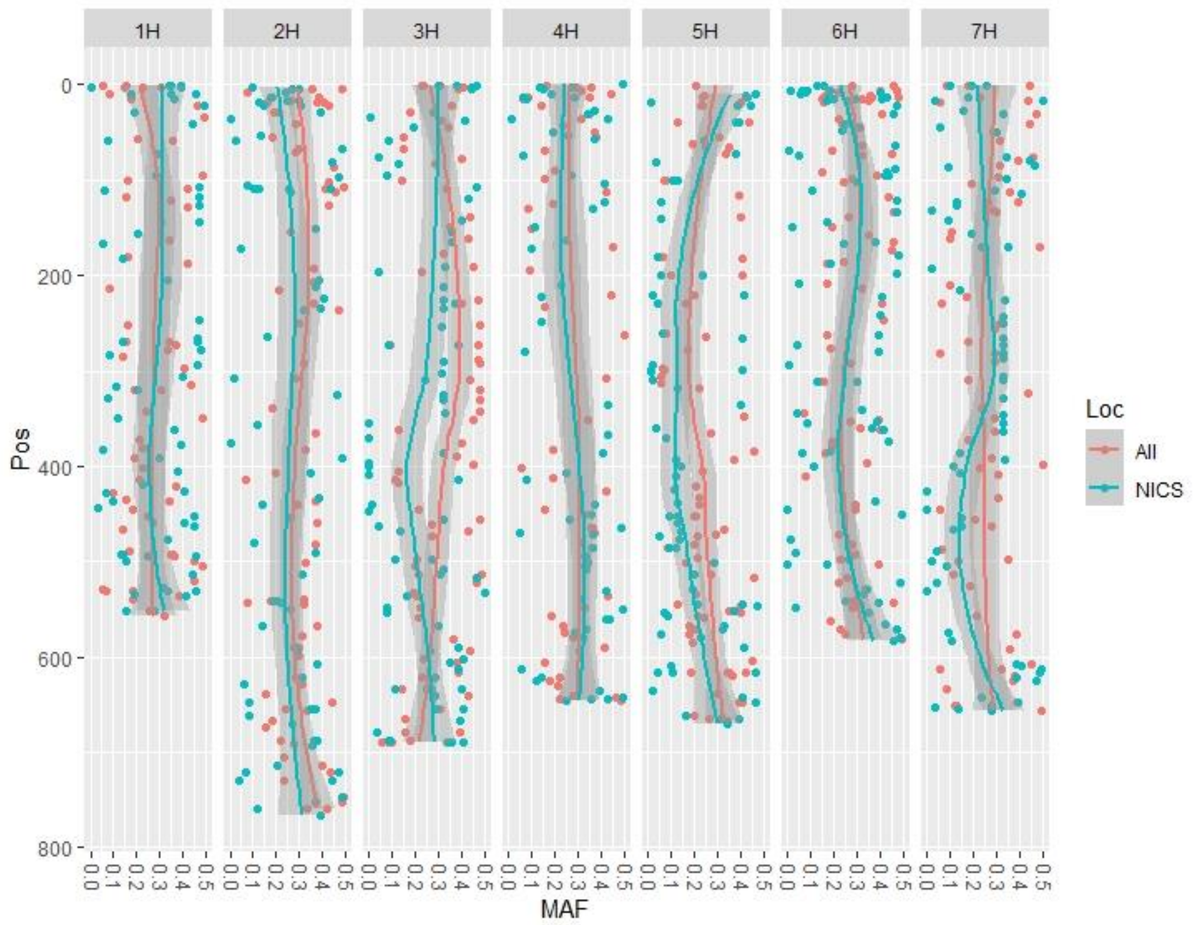
1H
2H
3H
4H
5H
6H
7H
Pos
MAF
Loc
All
NICS
0
200
400
600
800
0.0 0.1 0.2 0.3 0.4 0.5

F

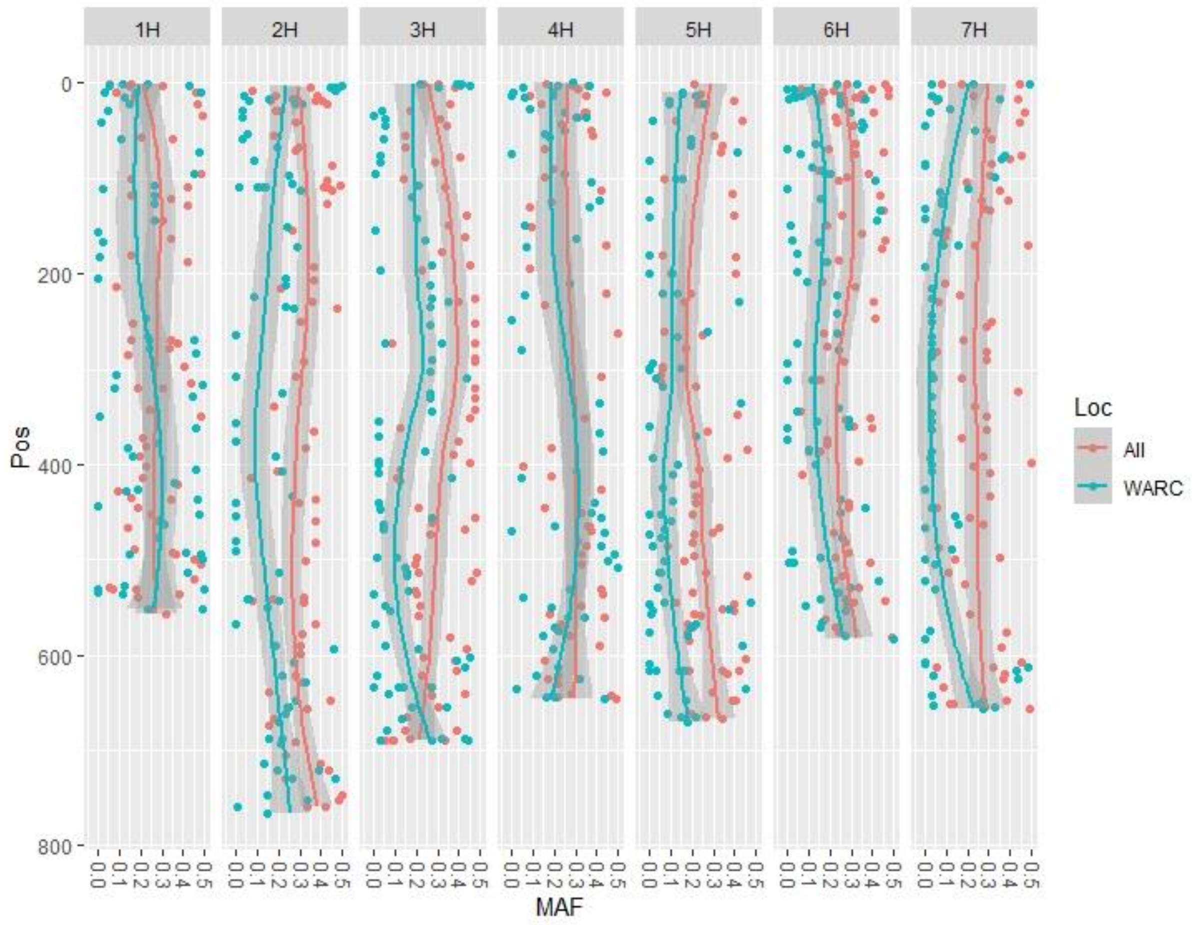
1H
2H
3H
4H
5H
6H
7H
Pos
MAF
Loc
All
WARC
0
200
400
600
800
0.0 0.1 0.2 0.3 0.4 0.5

G

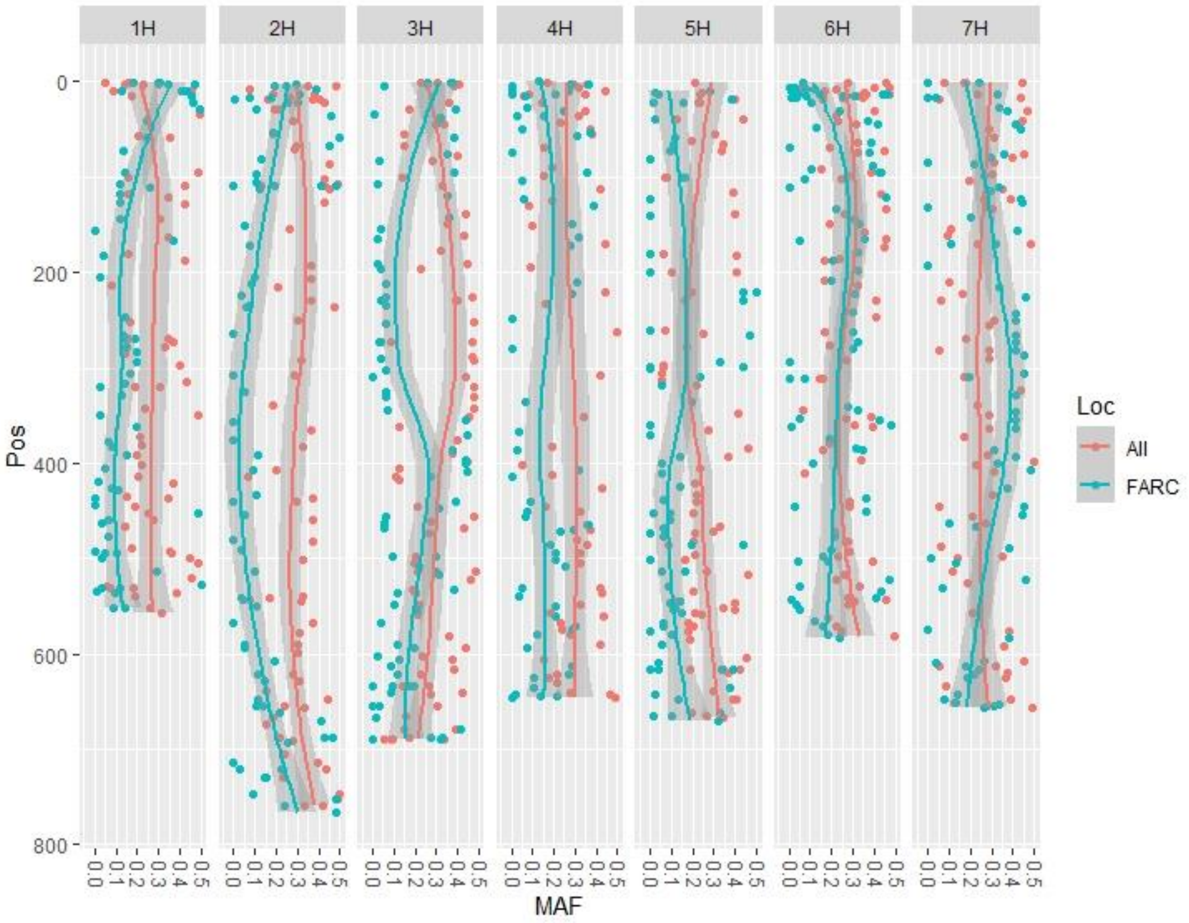

H

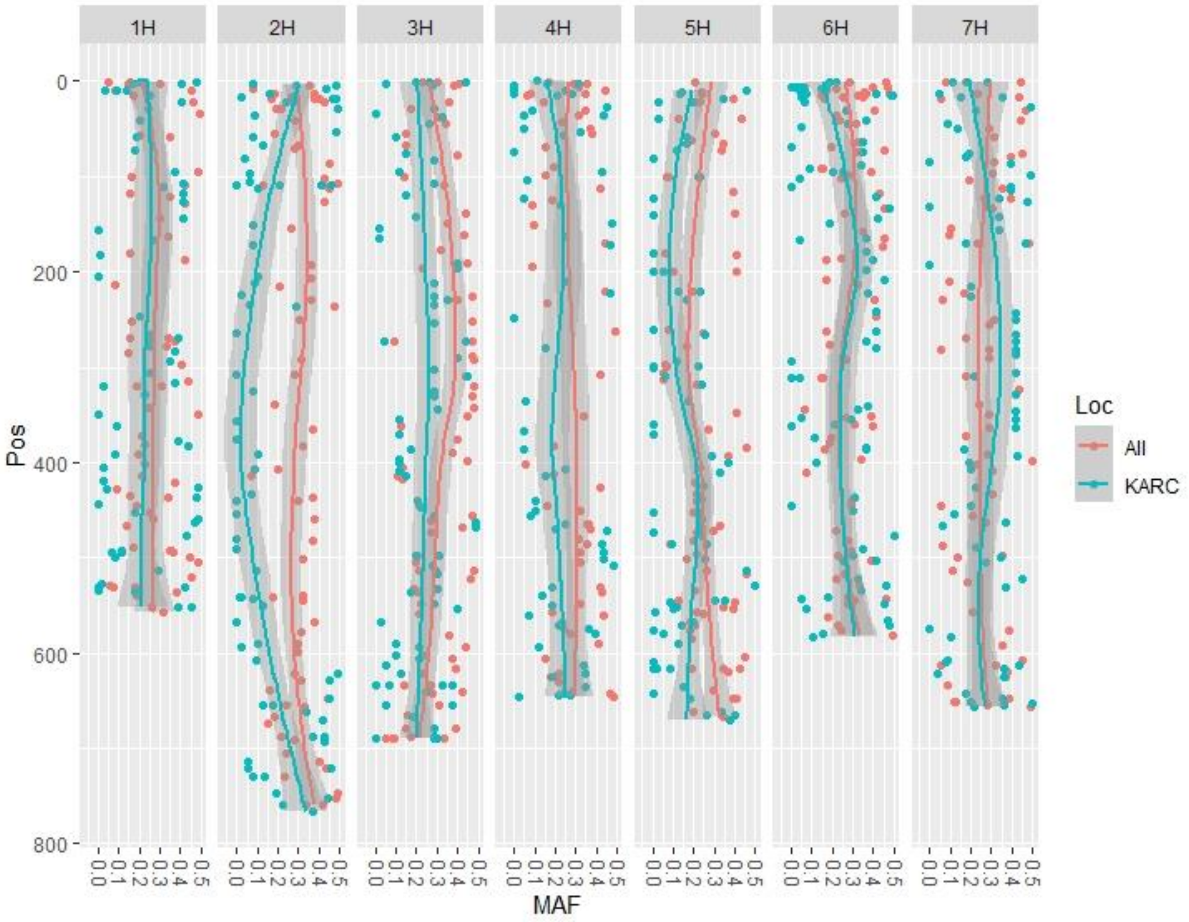

Supplemental Fig. 2. Distribution of minor allele frequencies (MAFs) along with barley chromosomes for each breeding station. Red and blue lines indicate locally weighted scatterplot smoother (LOESS) curves of MAF using all accessions and each breeding station, respectively. Gray area indicates a 95% confident interval. A: TARC, B: CARC, C: NAES, D: TAES, E: NICS, F: WARC, G: FARC, H: KARC.

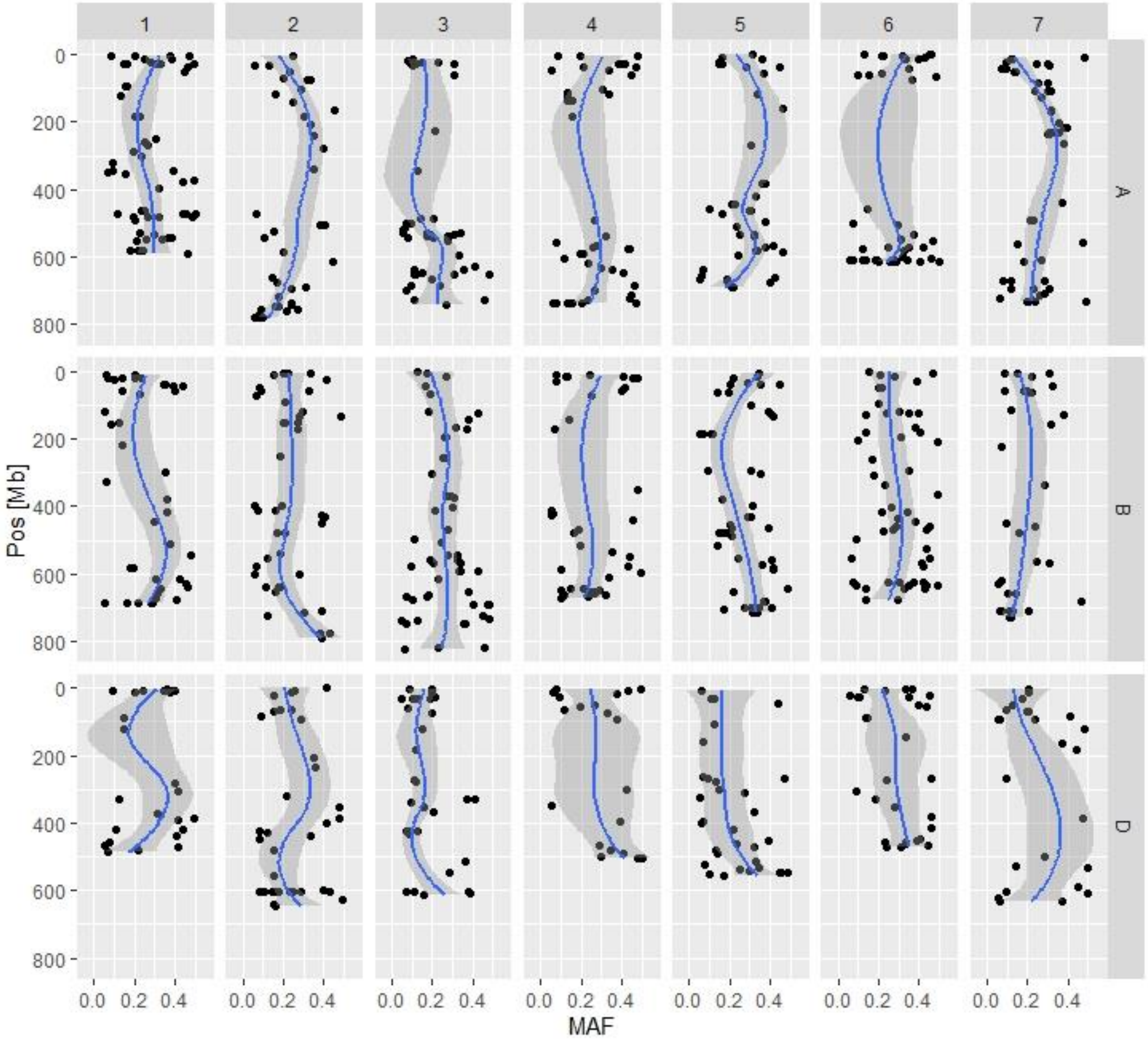

Supplemental Fig. 3. Distributions of minor allele frequencies (MAFs) along with wheat chromosomes. Blue lines indicate locally weighted scatterplot smoother (LOESS) curves. The gray area indicates a 95% confident interval.

A

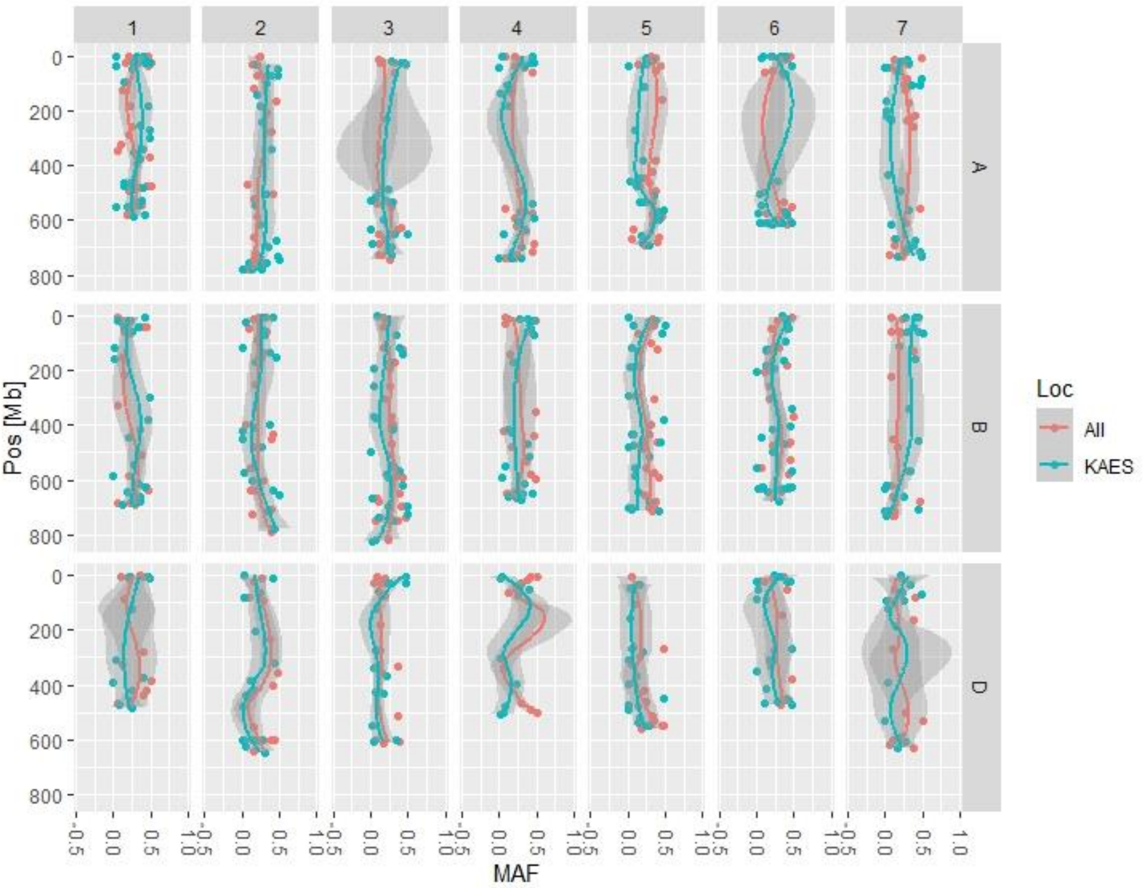

1
2
3
4
5
6
7
A
B
D
0
200
400
600
800
Pos [Mb]
-0.5
0.0
0.5
1.0
MAF
Loc
All
KAES

B

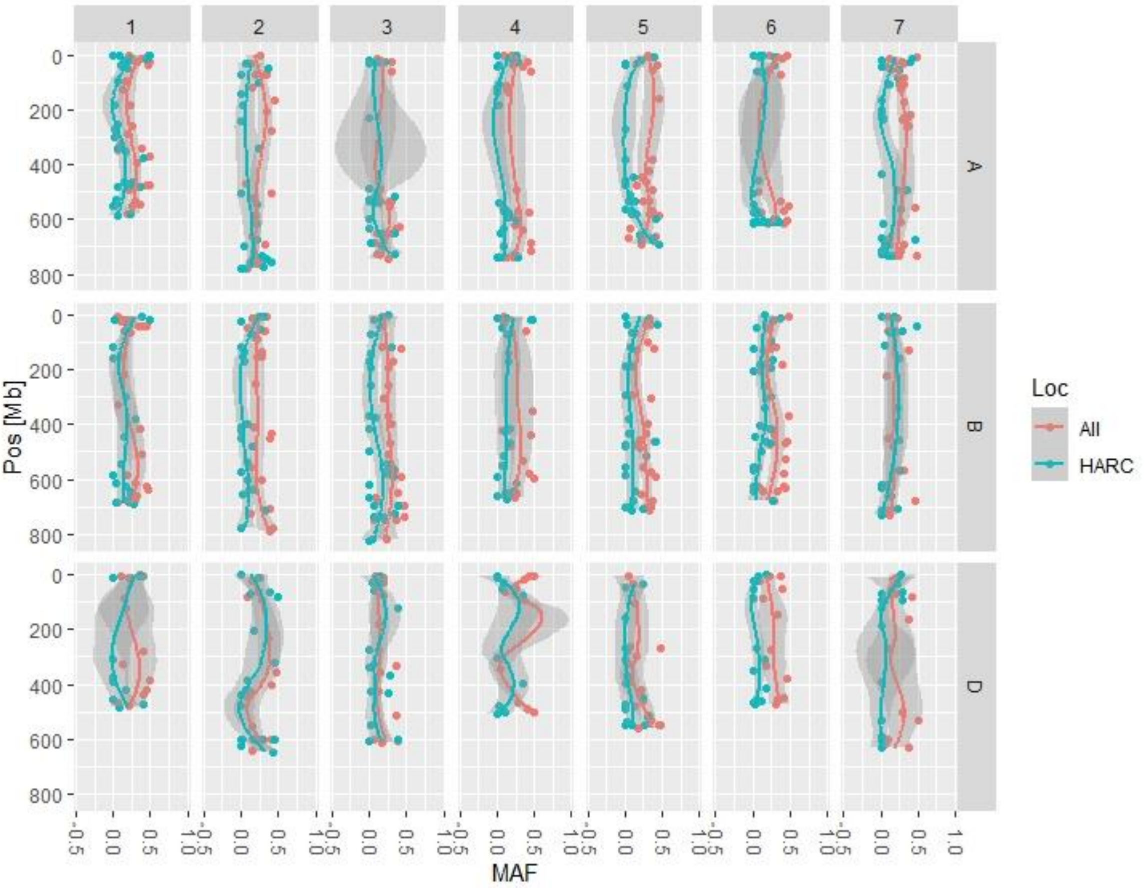

1
2
3
4
5
6
7
A
B
D
0
200
400
600
800
Pos [Mb]
-0.5
0.0
0.5
1.0
MAF
Loc
All
HARC

C

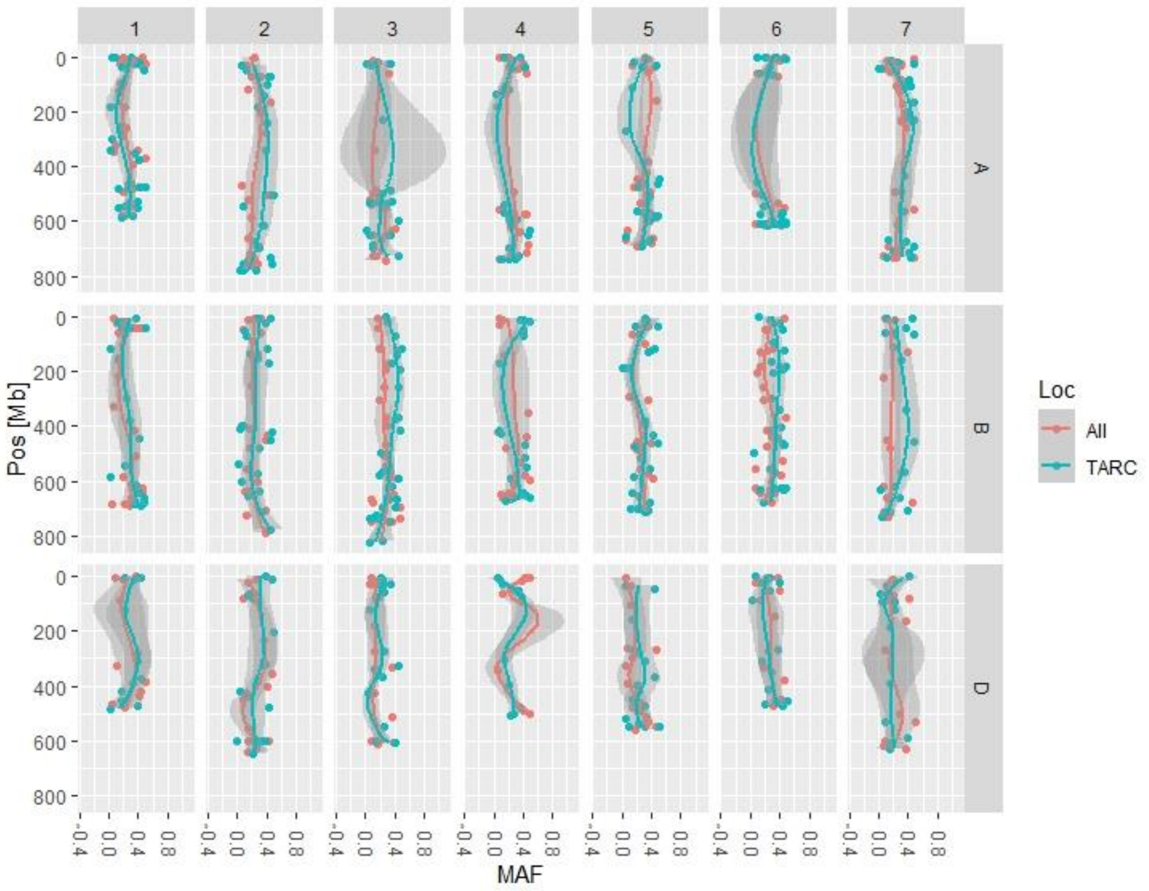

1
2
3
4
5
6
7
A
B
D
Pos [Mb]
0
200
400
600
800
-0.4
0.0
0.4
0.8
MAF
Loc
All
TARC

D

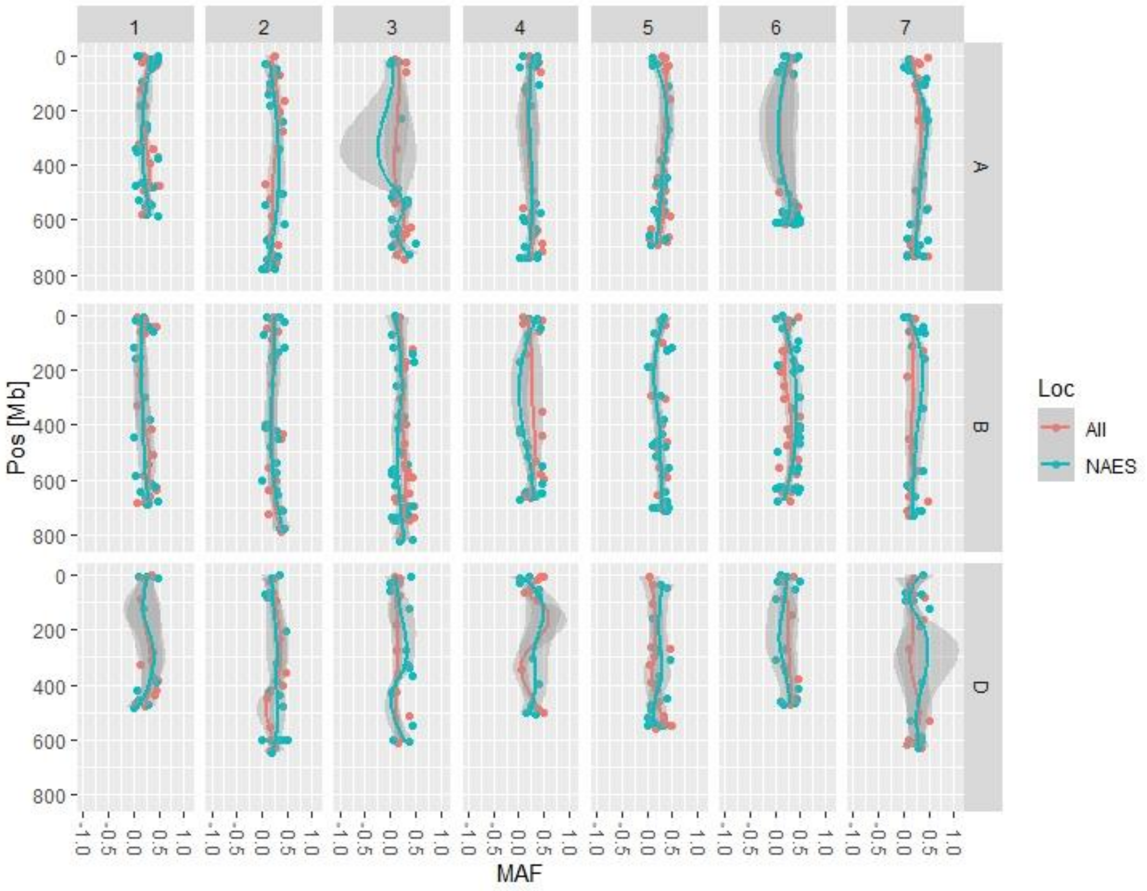

1
2
3
4
5
6
7
A
B
D
Pos [Mb]
0
200
400
600
800
-1.0
-0.5
0.0
0.5
1.0
MAF
Loc
All
NAES

E

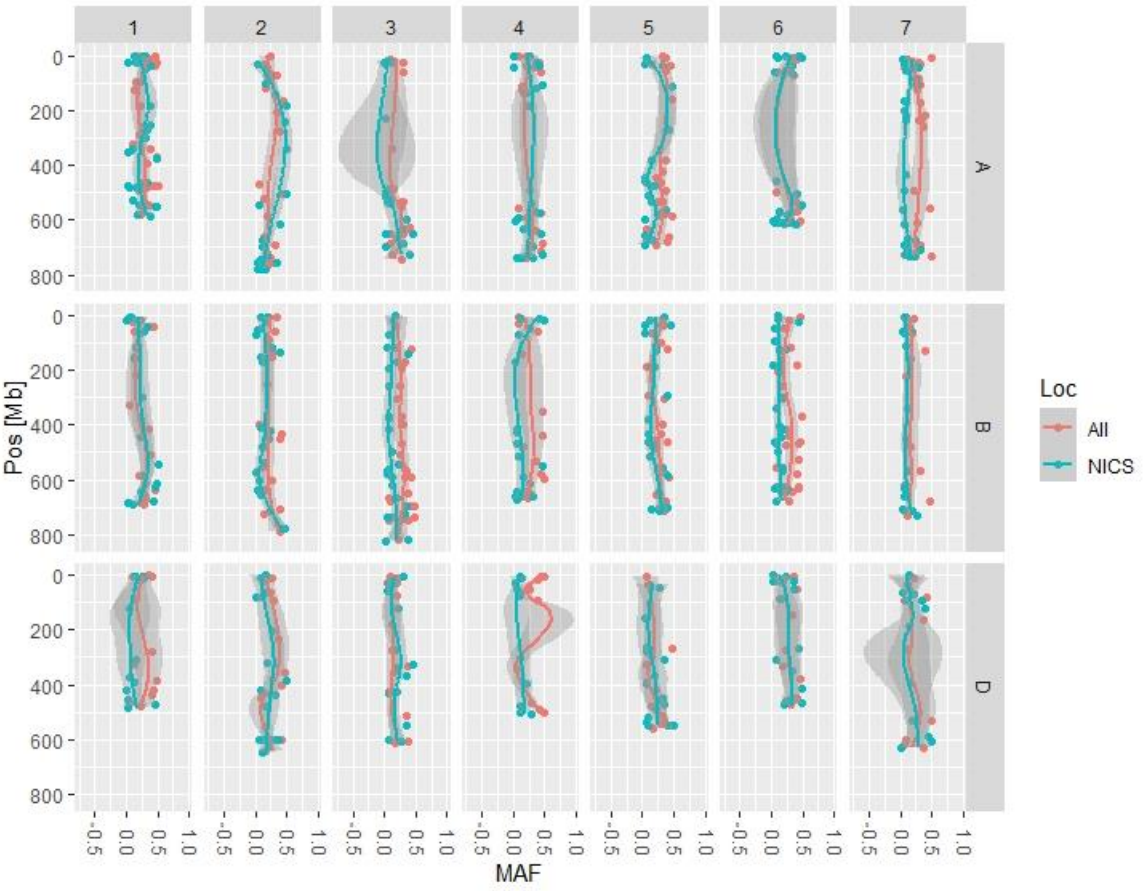

1
2
3
4
5
6
7
A
B
D
Pos [Mb]
0
200
400
600
800
-0.5
0.0
0.5
1.0
MAF
Loc
All
NICS

F

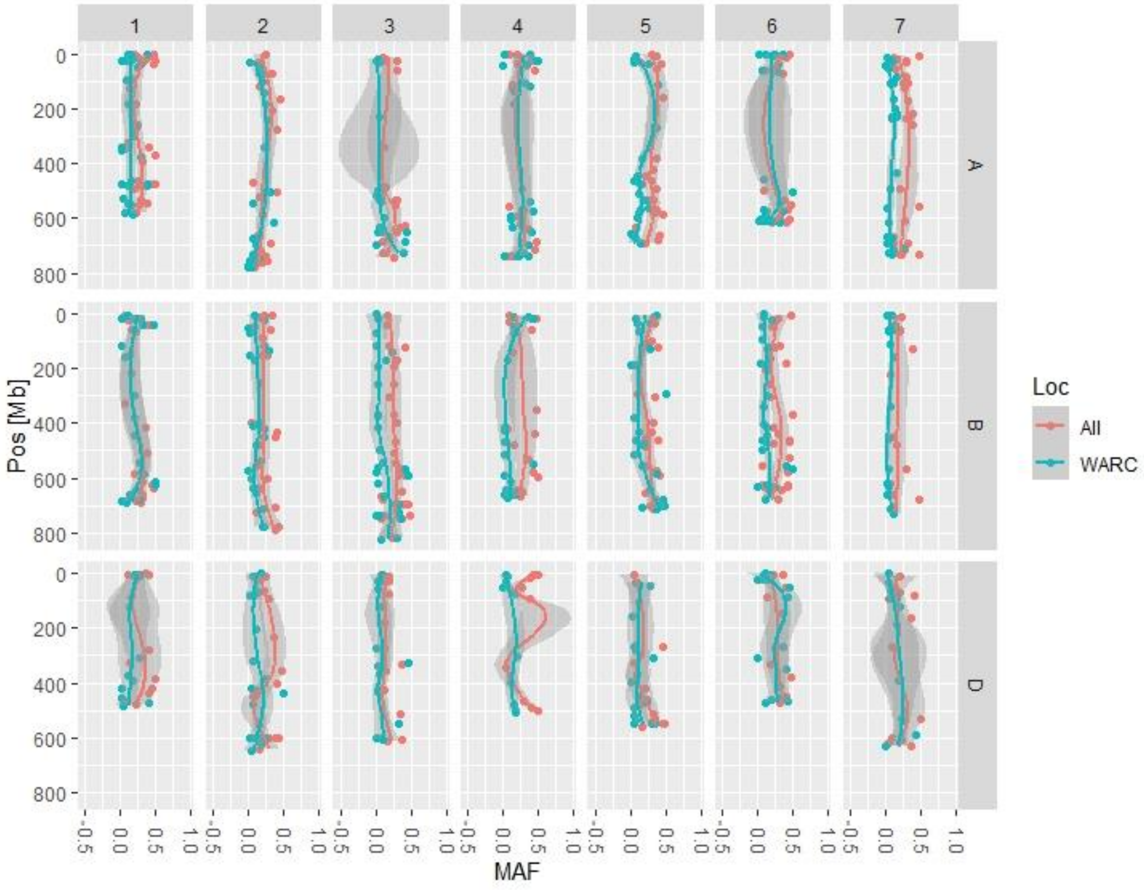

1
2
3
4
5
6
7
A
B
D
Pos [Mb]
0
200
400
600
800
-0.5
0.0
0.5
1.0
MAF
Loc
All
WARC

G

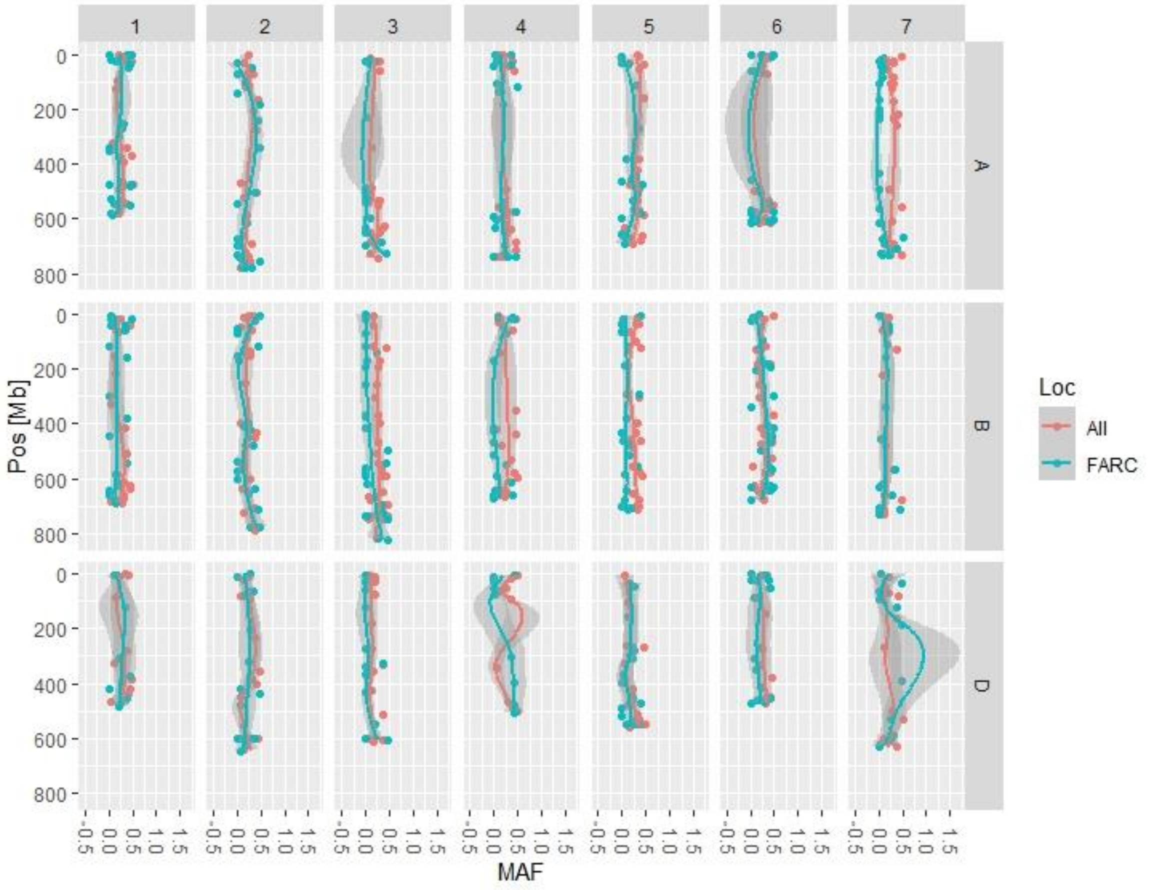

H

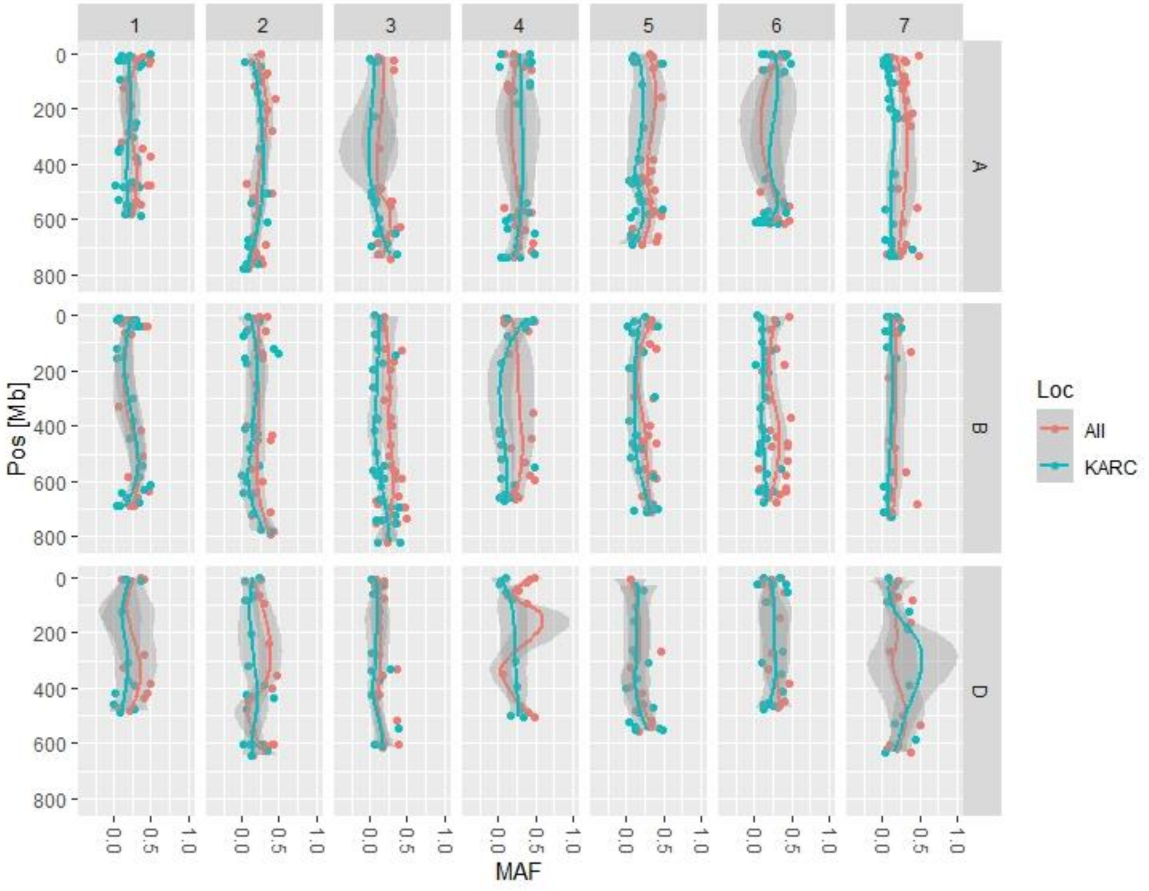

Supplemental Fig. 4. Distribution of minor allele frequencies (MAFs) along with wheat chromosomes for each breeding station. Red and blue lines indicate locally weighted scatterplot smoother (LOESS) curves of MAF using all accessions and each breeding station, respectively. Gray area indicates 95% confident interval. A: KAES, B: HARC, C: TARC, D: NAES, E: NICS, F: WARC, G: FARC, H: KARC.

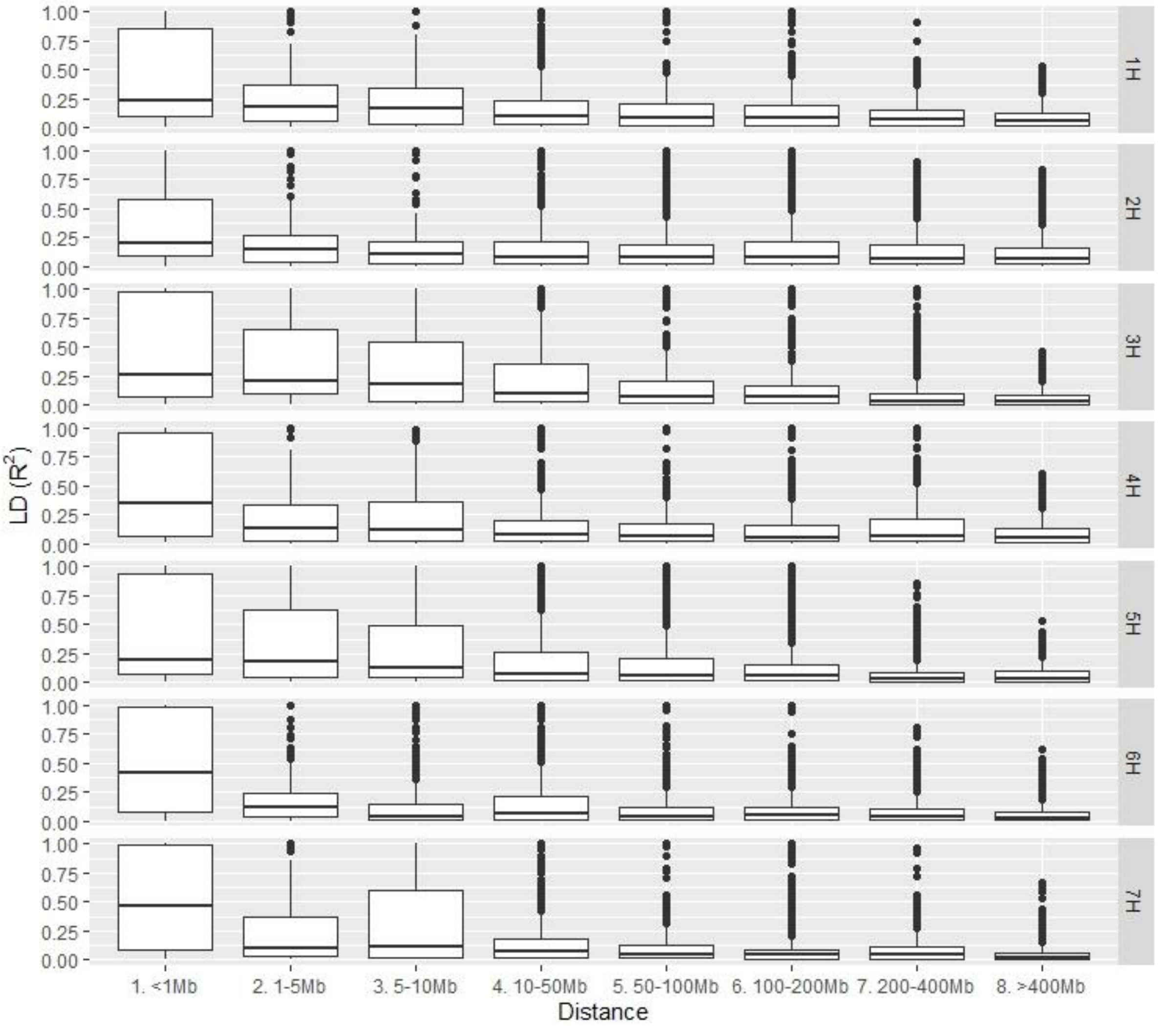

Supplemental Fig. 5. Linkage disequilibrium (LD) decays of each barley chromosome as a function of physical distances between polymorphic sites. LD were evaluated by the degree of r2 values.

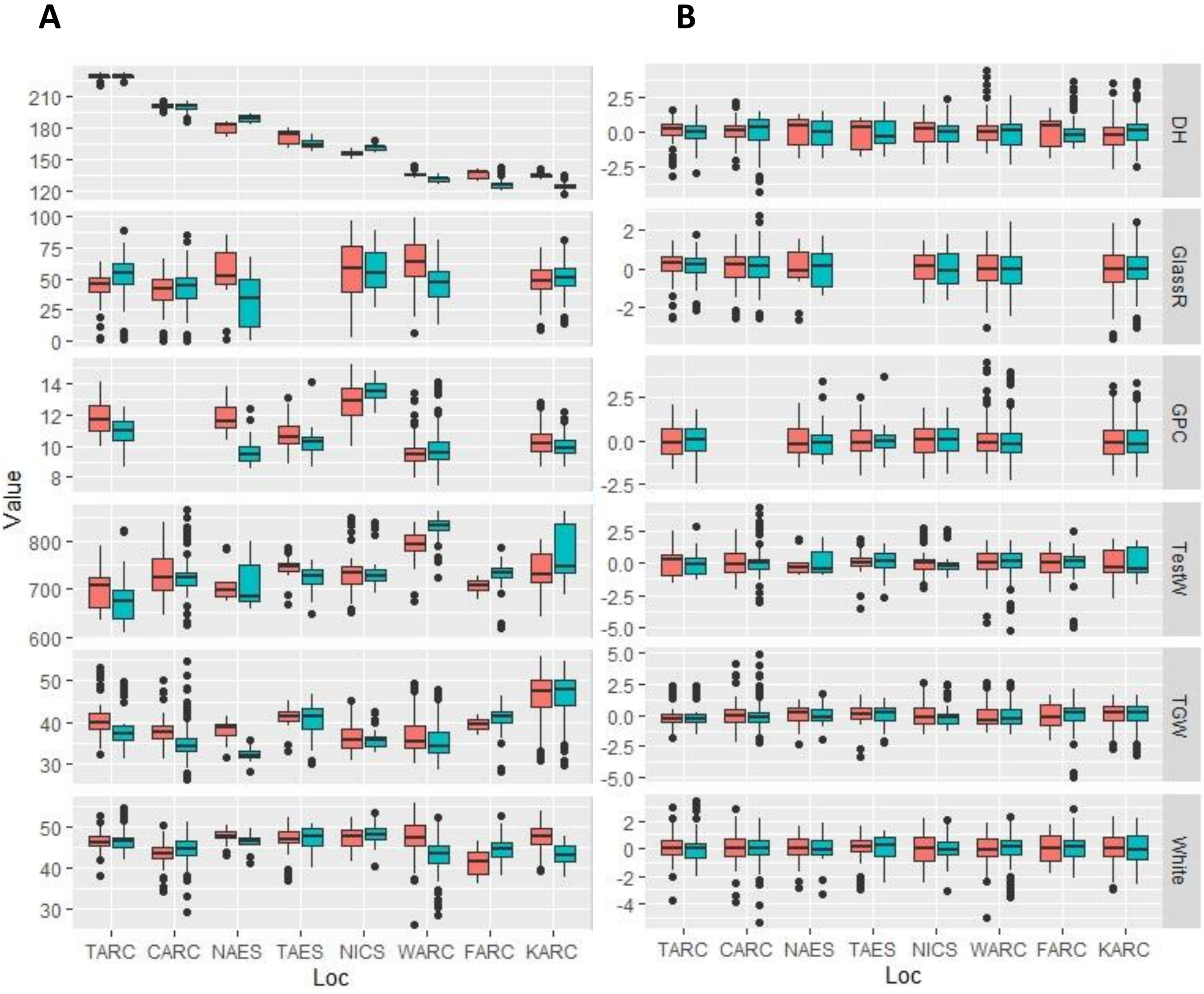

Supplemental Fig. 6. Comparison of six trait values among eight locations and two years in 1,707 entries of 927 barley accessions. Distributions of raw (A) and scaled values by each combination (B) are described. Red and blue boxes indicate data of samples harvested in 2018 and 2019, respectively. DH: days to heading from sowing [day]; GlassR: glassy kernel rate [%]; GPC: grain protein content [%]; TestW: test weight [g]; TGW: thousand grain weight [g]; White: whiteness of pearled grain [%].

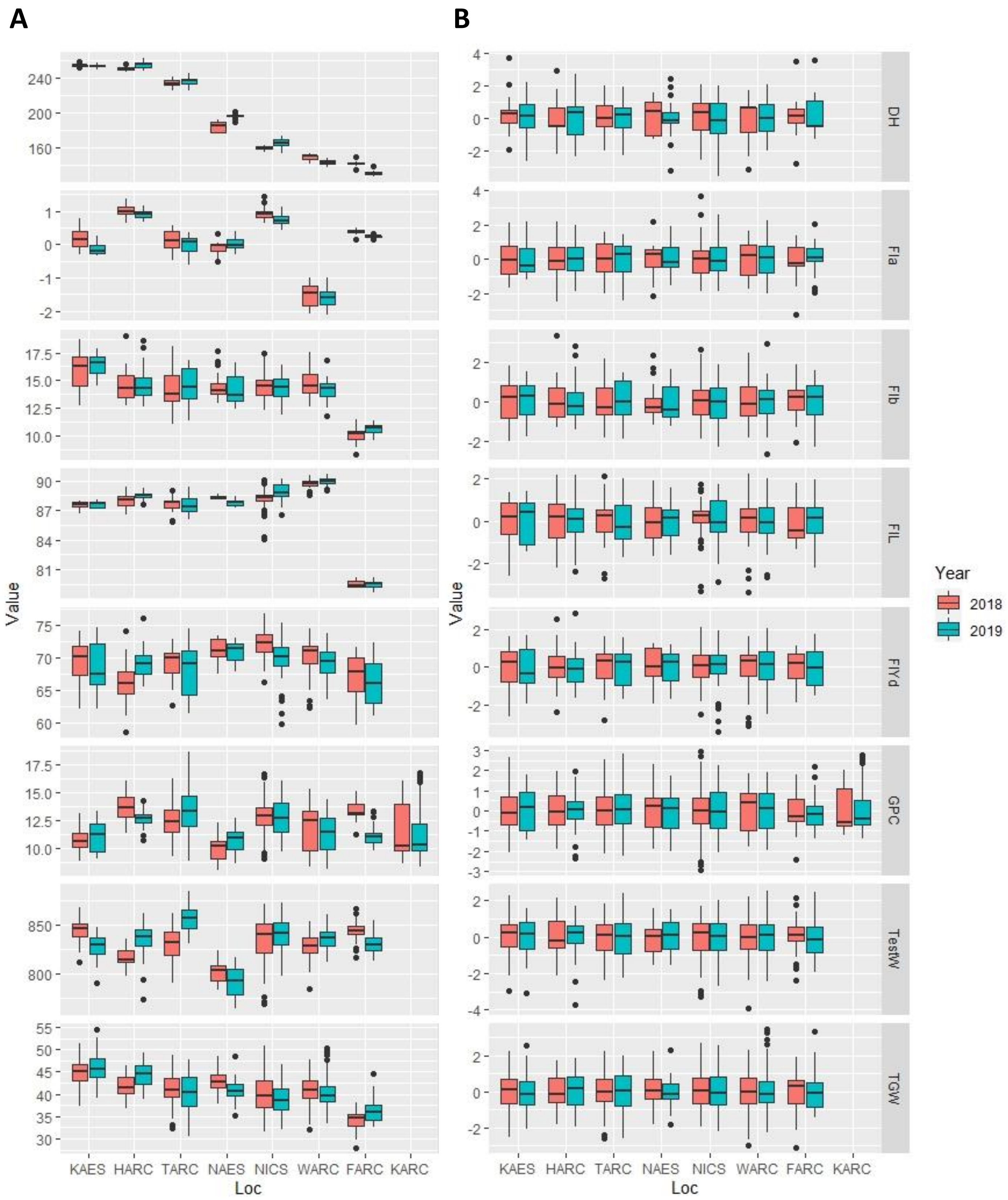

Supplemental Fig. 7. Comparison of eight trait values among eight locations and two years in 2,319 entries of 1,670 wheat accessions. Distributions of raw (A) and scaled values by each combination (B) are described. Red and blue boxes indicate data of samples harvested in 2018 and 2019, respectively. DH: days to heading from sowing [day]; Fla: flour color a* Flb: flour color b*; FlL: flour color L*; FlYd: flour yield [%]; GPC: grain protein content [%]; TestW: test weight [g]; TGW: thousand grain weight [g].
