# Supplementary material for: Developing core marker sets for effective genomic-assisted selection in wheat and barley breeding programs
Source: Breed Sci. 2022 Jun 29;72(3):257–66. doi: 10.1270/jsbbs.22004 (PMC9653188; doi:10.1270/jsbbs.22004)
Supplement: Supplementary file 2 — Supplemental Tables [file 72_257_s2.pdf]

doi: 10.1270/jsbbs.22004

Supplemental Table 1. List of amplicon sequencing primer sets for barley.

No.	Name	Type	Chr	Pos[Mb]	F_primer	R_primer
1	ICSB1215	CoreSet	1H	1.4	GTTCCCATCCACTCGTGCT	TGGTTGGTTGATGGTGATGT
2	ICSB0927	CoreSet	1H	3.5	GGATGCGTTACGAAGGGATA	CCATTCTTTGCATCATTCTCC
3	ICSB0351	CoreSet	1H	9.2	TGGATTGGATAGGCCAAAAG	TCACCGACCAATCATTTCAA
4	ICSB1216	Other	1H	9.8	GTTGGTGCATGCTATCGTTC	CGATGAACTGCAATGAACAAG
5	ICSB0187	Other	1H	13.0	ATTCTCCGGTTTTCGTCAACAT	CTGTATGCTACTTACGTCGTGGAG
6	ICSB1217	CoreSet	1H	14.3	GACCTGAAGGCGTACCTGAG	TTGTCCGCACGATCACCT
7	ICSB0253	Other	1H	18.4	GACATGCATTTTGTACACATAGTTCT	AGTTTTGTGAAATTTGTCGATTCA
8	ICSB0352	Other	1H	18.8	CTTCGTTCTTGGCTGATGGT	ACTAATGTTCTGGGGCAACG
9	ICSB1218	Other	1H	19.5	CCTAGCCTCGCACATCTTTT	ATCGTCCCAGGGATGCTG
10	ICSB0001	CoreSet	1H	22.6	AATGGTTCCATGTTTCAGGGAG	TCCTAAGGGCAATGGTAATGGT
11	ICSB0928	Other	1H	22.6	GGACCATGGATGATGGTGAC	CCCCATTCGAACGTAACAAC
12	ICSB0353	CoreSet	1H	29.1	GATGTGGTGTGATGAGCGTA	GGTCGGTTTTCAACATAATGC
13	ICSB0002	CoreSet	1H	34.1	ATGTAGCTTGGGACTGACGGAG	AGGAGTGTGCTCAGTGTGCAGT
14	ICSB0929	CoreSet	1H	39.8	TTGCTGTGACCAGATGATGG	GCAAAGGGGAAGAACAGTTG
15	ICSB0354	CoreSet	1H	41.1	TTTTCCAGGGAAATCAGTTCA	AGCTCAGGTGATTGCAGCTC
16	ICSB0930	CoreSet	1H	43.7	TTTTCGTTGTTTCCTTCATGC	CACGACTAGTATGGACATAATCAGAA
17	ICSB0355	CoreSet	1H	57.5	TGCCTTCCAGGATGGAGAT	GAGAAACAGACGTCGAGCAA
18	ICSB0356	CoreSet	1H	58.9	CTGCAATTATCAACCGGAGA	CGTTGGTATGAGCACAGCAG
19	ICSB0357	CoreSet	1H	63.2	GCACTTGGAGGACCCTAACA	GCTGCAATTTGAGACCACCT
20	ICSB1219	Other	1H	68.6	CGCTCGAACAAGCCATAGA	ATTTTTGCGTTTTGGATTGG
21	ICSB0358	CoreSet	1H	72.0	GCTTCCTCATCGCTGACTTC	GGAAAGAGATCTAGCCCGAAA
22	ICSB0359	Other	1H	80.3	TGGTACAAGCGGCCTTAAGTA	GCATTGGTGAAACAAGCAGA
23	ICSB0931	Other	1H	81.8	TGGCATGATAATTTGTGTGC	CCATCTTACCCATTAAATTCGTC
24	ICSB0003	CoreSet	1H	91.6	TGATTATAGGTAAACACGTCACACG	AGCTGATGATCGACTTCAATTTAG
25	ICSB0125	Other	1H	91.6	AGATGCATAGAAAAATTAAGGCCC	CTATATGCATGAGAACAGGACAGC
26	ICSB0126	Other	1H	91.6	CGTCCACTTGTATTTCACTCAAAA	AAAATGATAAACAAAATCCCTTCA
27	ICSB0127	Other	1H	91.6	TGATTAAATGAACACCATAGCACA	TGCCTATTTTCCCACTATTTCTTC
28	ICSB0360	CoreSet	1H	95.1	GCAGGGATTCTCACACCAAG	TCGTCTCCCAGTAGCTTTCTTC
29	ICSB0932	CoreSet	1H	95.1	AAGTTTAGTGGCGCGAGGAT	GTTGTCATTCGGGAAAAGGA
30	ICSB0361	CoreSet	1H	100.5	TTTGTACCATTGTCGACTCCA	AGTCTTGCCTGCGCAATAAC
31	ICSB1220	Other	1H	106.5	TTGCAGCTTTCGTAGTTGCT	TGCTGAAAGTATCCCTGATCC
32	ICSB0362	CoreSet	1H	107.3	ACAGCCGATCGCCTTATACA	GTTCAAACCGGAAGGTGAAG
33	ICSB0363	CoreSet	1H	108.6	GTTCCTGGAGGAGGTGCTCT	TGCCTCAAGTTGCTTTTCCT
34	ICSB0364	CoreSet	1H	111.6	TCCAAGGTCAGCACAGAAAA	TGGTTTTCCAATCCAAGAGC
35	ICSB0365	CoreSet	1H	117.0	CGCCTACCTTCTGATGATCG	AACCCTCTGCGCATAGACAG
36	ICSB0366	CoreSet	1H	118.6	GCTCACCAAACAACCCTGAC	TGGGTGATGGGAGTGTACTG
37	ICSB0933	CoreSet	1H	121.5	TTATTGAATTGCACCCATGC	CAAAGGACATCCTTAACCCAAG
38	ICSB0367	CoreSet	1H	125.9	GTTCAGTTGATGGCTGCTCA	TCAATTGCAATCTCACGATG
39	ICSB0368	CoreSet	1H	128.2	CATCCCCCAGACGATCAATA	TTGGACAGGGTTCTTGAAGG
40	ICSB0369	CoreSet	1H	143.4	CGTGAAAGCAGGTCAAACAA	GTCCCACTTTGTGGCTGATT
41	ICSB0934	CoreSet	1H	144.6	TCTCTCACTTGATTCCATCCAA	ACTCCGACGTGTTCGAGCTA
42	ICSB1221	CoreSet	1H	157.0	CATCCAATATGGGCACATACAA	TGAGTTTAACCGGTGGCATT
43	ICSB0935	CoreSet	1H	162.9	CAAAAGACGAGTTTGCGACA	CTTCAAGGGAAAAACCAACG
44	ICSB0370	CoreSet	1H	167.3	GCGAACAACAGCAGAACCTT	CCTTTTTCTTCCATGCTTGC
45	ICSB0371	CoreSet	1H	180.0	CGTCCAAGATGAACTGACCA	TGCTCTTATGTCGGGACTGA
46	ICSB0936	CoreSet	1H	182.6	AACATCGCCTCCATCATAGC	AACGCGTCGTGTGTGTAGAG
47	ICSB0372	CoreSet	1H	187.6	CAAGCCTTGCATGGATTTTT	AAGCAAACCCCTCCTTCAAT
48	ICSB0937	CoreSet	1H	204.1	TCCTTCTCAGGGAAGTGGAA	TCAGGAGATGTCTCAGATGTCAA
49	ICSB0373	CoreSet	1H	213.2	TGTAATCGGAAGATGCTCGAC	GCCGTCACCGACATAACTTT
50	ICSB0938	Other	1H	223.6	CTAACCCAAGTCCGATCACC	GCCTCGGAACACAAGAGAAC
51	ICSB0939	CoreSet	1H	233.3	AGAGGCTTTTCAAGCTTCCTA	TGTAGCAGTTATATTTTCCATTGTCC
52	ICSB0940	CoreSet	1H	246.4	TCGCGGTATACAAGTCACCA	TGGTTTCGGAAATGGGATTA
53	ICSB0374	CoreSet	1H	251.2	TGTCATGTTGAACGCTATGGA	TCGGAAAGTAAATACTGCTTGACA
54	ICSB0941	Other	1H	257.7	GCACTTGAGCAACCAGATCA	TTTGCATTTTGCATTTTGTGA
55	ICSB1222	CoreSet	1H	265.3	ATTACTCCAAACCGGCAGTC	ACTCAGTGGCGATAACATTTTT
56	ICSB0942	CoreSet	1H	268.6	GGATGAGCTAATGGGTTGATG	CATGTGCTTGCTGCTCCTAC
57	ICSB0375	CoreSet	1H	269.2	GCTAAGGAGGGTGAGCCATT	AATGCATGGCCTAAATAATGC
58	ICSB0376	CoreSet	1H	270.0	GCTCTAGTGGCAGTCATATCAGA	ATGTGCGGGCACTAGTGTTA
59	ICSB0377	CoreSet	1H	273.3	AGGTGAAATCACGGAGATGG	CCTCCATATGGTTGCACAAG
60	ICSB0943	CoreSet	1H	277.8	AGCCGTTCTGGATCTTCTTG	CGCTTAACATTCGATGATGC
61	ICSB0233	Other	1H	282.8	AATGCATTCCTTACAGTTTCTTGAG	TGGAAATAAAATTGCAGCAAGTAA
62	ICSB0378	CoreSet	1H	282.8	AGGGAAACACCAAGCAAAAA	TTGCGTTGAGAAGGGTGAT
63	ICSB0379	Other	1H	282.8	CGGGAATCTACCCTTGTTTG	CATTCTCTGCGATCATCCAA
64	ICSB0004	CoreSet	1H	285.8	TATGATGCTTGGACTGTTCATGG	GTACTTTTCAGCGTATGGACGAT
65	ICSB0169	Other	1H	285.8	AGCTGAGTATTCAACGTGATTTTG	GAGGAACAGCACCTTTGACTTTAC
66	ICSB0170	Other	1H	285.8	GCATTACAGGTCGCTGGTATACAT	ACATGTATTCTGTCCATCAGGTTT
67	ICSB0380	CoreSet	1H	293.0	AGCTAGCTGCAACGACTTCC	TTGCACTAGTAGGAGCGGACT
68	ICSB0381	CoreSet	1H	298.0	GCGCGTCTGTGTGTGTTTAT	GCGCCCAGAAAGTTAGTTCA
69	ICSB0944	Other	1H	298.0	TTGGCATGCAGGTTATGCTA	GATGTGGGTCATCCCAAAAG
70	ICSB0382	CoreSet	1H	306.4	GGTAGCTCTTTTCGGGCTGT	AATTCCCAGCGGTATTCACA

Supplemental Table 1. List of amplicon sequencing primer sets for barley.

No.	Name	Type	Chr	Pos[Mb]	F_primer	R_primer
71	ICSB0945	Other	1H	309.9	GGAGGGCAGAGAGATGATAGG	CCACTCCCAGCCAAGCTC
72	ICSB0383	CoreSet	1H	314.3	TCCACATCGCGAGATTCATA	CACAGGAGCAGGTCATCAAC
73	ICSB0005	CoreSet	1H	317.2	TCTTGACTCTGGTTGGGAGTAAAC	GATCAGCACAGTCCATCACCAC
74	ICSB0946	CoreSet	1H	320.0	TTAGCACGAACCTCCTTTCC	CACCTGAAGAGTGCAAGCAT
75	ICSB0384	Other	1H	320.6	GCAGAAAGAGCAATGCATCC	TGAAATATATAATTGGGGCTATCG
76	ICSB0385	CoreSet	1H	328.0	GCGATGCTGTTGTCTCACAC	CATCTGATGATGGCTCAAACA
77	ICSB0386	CoreSet	1H	336.1	AAGCATGTACAACTGCAACACC	CCTTGTTAATTTCGGTGGTTCT
78	ICSB0387	CoreSet	1H	342.7	GCCAACCCAGAATGCAAGTA	GGTACAACGACAGCCTTGTG
79	ICSB1223	CoreSet	1H	349.5	TCATGAGCCTGCCATATTCA	CTCCAACAACAGCTCAACCA
80	ICSB0388	CoreSet	1H	350.5	CAGCAAATTGTGCAAAACTACA	AGCTCAACAGGGATGCAAAT
81	ICSB0389	Other	1H	357.3	CCGCGATGAAGAGGAAAATA	TCATTACTCATCCGAATAATGCTC
82	ICSB0947	Other	1H	357.9	CGTGAGGCATGATACTCCAA	AGCCCAGGATATTGTGGATG
83	ICSB0390	CoreSet	1H	362.2	GTGCAGCTTCATGGTACAGC	TCTCCTTTTTGTGCCTTGCT
84	ICSB0006	Other	1H	364.7	AGGTTGATAGGGTGGTCGAATTT	TGGGACTCACATTCTTGTGATATT
85	ICSB0160	Other	1H	364.7	ATCTTTGATCCTGTTAAGACTCCG	CAAATTCGACCACCCTATCAAC
86	ICSB0391	CoreSet	1H	372.3	TCTGAGTCGGGTGCTTTCTT	CCTTGCCATTATCCCATTGT
87	ICSB0392	CoreSet	1H	376.9	GGCACTTTGGCACAGAAGG	CCACTCCTAAGATGCCTCCA
88	ICSB1224	CoreSet	1H	380.8	TGTAGCAATTCAGCATAGGAAC	AGACGGGCACAAAAGAGAAG
89	ICSB0393	CoreSet	1H	382.6	GGCAGAAGAGGCTGAATACG	CATGTCTGGCATTTTTGGAA
90	ICSB0394	CoreSet	1H	386.0	GGTTGCTTGAGAAGCATGAA	GTGCAACTGCAACACAGTGA
91	ICSB0007	CoreSet	1H	392.0	CTTGCTCAGCTGTTTCTTCAACTA	GCTAGCTTCAAACCCACAAAAT
92	ICSB0158	Other	1H	392.0	GGACAATAATGTCTGCTGAAGCTA	TAATCTTTTGGCAAGGTGGAACTA
93	ICSB0159	Other	1H	392.0	TCTTTCAAGTTAGAAAATGGCTCC	AGCAAGAAGGTCTTCATTCTCTTC
94	ICSB0395	Other	1H	394.0	GAATGAGCGGAGCGTCTG	CGTCTTCATCACCGACAAGA
95	ICSB0396	CoreSet	1H	401.2	TTGCCAATACACGACTCATCTC	GTCGGTGCGTCTTTCTAGGT
96	ICSB0948	Other	1H	401.4	TTCTGTGCAGCAATGAGGAC	TGCAATTATGTGGACCGAGT
97	ICSB0397	CoreSet	1H	404.9	GGAGGAGCCATTTCAACTGT	GCGAAACCATTTTCGCTATG
98	ICSB0398	CoreSet	1H	410.4	TCCACCTCAAGAGATTTCAGC	AAGGAGCCTTTTCTTTGTGC
99	ICSB0399	CoreSet	1H	414.9	CTCAGAAAATTGGCTGCCTTA	TCAAGTCATCTGAGCCAACAA
100	ICSB0949	CoreSet	1H	418.9	GACGAATGGACAGATGAGGAA	TTGGCTTTTTGTAGTTTTCAGG
101	ICSB0400	CoreSet	1H	420.7	GTTGTGGCCTCACCAGAAAA	GGCCTGCCATTCTCTTTCTC
102	ICSB0401	CoreSet	1H	427.2	ACAAATACATCACCGGCAAA	ATCAGCGGAAACACCAAAAA
103	ICSB0008	CoreSet	1H	428.4	ATTTCCGAAAGAAGACGCAATA	TTACTGCTCACTTCACATGACCTT
104	ICSB0950	CoreSet	1H	428.4	TGATGTAACCAGAGAATTGCTTC	CAAAATCTATTAACGGTCTTTTGC
105	ICSB0402	CoreSet	1H	435.7	TCCCTGTCCCATTATTTGTTG	AATCGCTGAACCCAGTTCC
106	ICSB0951	CoreSet	1H	437.3	AACTGGTCCAATTGTTACATCA	TCTCTGATTTGCACCGGTTT
107	ICSB0952	CoreSet	1H	444.0	TGCTTCGGTTAAGGTTCCTC	CACGCCAGGTTTCAAGATTT
108	ICSB0403	CoreSet	1H	444.5	CACATTACCCGCCAAAACTT	ACGTCTCGATCCATTTAGCA
109	ICSB0009	CoreSet	1H	445.6	TCTGCGTCGAAAGTAACTTCTGTA	ATTCAGCTCTTGTGAGACGAAAAT
110	ICSB0404	CoreSet	1H	450.2	TGCATGAACGGATAAATACGA	AATATCCAATGAACCCCCTTG
111	ICSB0953	CoreSet	1H	452.3	TCTTCTGCCACCTTCCAACT	CTAGGTGGCTCTCGCAAAGT
112	ICSB0010	Other	1H	454.1	TTTCTTCCAACACACTAGCAGAAG	GGCATCCAACATCTTACTGACAAT
113	ICSB0109	Other	1H	454.1	TTTCTTCCAACACACTAGCAGAAG	GGCATCCAACATCTTACTGACAAT
114	ICSB0405	CoreSet	1H	459.0	GCTGTCTGCCTATTGATCTCG	TGGACACACAGACCAACTGAA
115	ICSB1225	CoreSet	1H	462.1	GGTCGTGTTTCGCTCTGG	CGCAGCTGCAAATAGGAGAT
116	ICSB0406	Other	1H	463.5	CGGAAGTTGATTACATCTTAAACCA	ATGCCCTTCACAGAGCATTC
117	ICSB0011	CoreSet	1H	466.3	GCTGTGTCACTCTTTGAATGTTCT	CAATCTAGCACAGATTCATCACCT
118	ICSB0407	CoreSet	1H	477.3	ACATAGCCGCTCTGGATCAC	CCTCAAAATGCTAGCCTCCA
119	ICSB0408	CoreSet	1H	489.3	CTCCGACCCTTACTCTGCTG	ATCTCGGCGGATGAGATTTT
120	ICSB0954	CoreSet	1H	489.8	CTCGCATTCACGAGTAACGA	TAGAACGCCACAAAATGCAG
121	ICSB1226	CoreSet	1H	493.3	GCGTGTGGCTCTTGATGAAC	GAGGCTGCATTCTACATGCTT
122	ICSB0098	CoreSet	1H	494.1	CAACCTGAAGCTGATGCTGTTA	GGAGCTTGAATTTTCATTGTTTG
123	ICSB0409	CoreSet	1H	494.9	CGGCCTGAATGTGTAGAACA	CAGGACCATTGCCAGTTACA
124	ICSB0410	CoreSet	1H	499.6	GCGACTGCCTGATGTTCTTT	CAGGCATACCCAGATGTGAA
125	ICSB0411	CoreSet	1H	504.5	CTTGGATTCACCGCAAATCT	CGTATGCAGCAAGACCTTCA
126	ICSB0412	CoreSet	1H	513.1	TATTGTGGTGCAGGGAGGA	TCATGAAGAACTGGGGAATGA
127	ICSB1227	CoreSet	1H	519.8	CATCGGGACGTGAAGACATC	TGTCACCGTCCTGATTGAAA
128	ICSB0413	CoreSet	1H	526.7	AGGAAGGTGGGGGTTCTCTA	CCGATTCCCCAGTCAAAAT
129	ICSB0955	Other	1H	526.9	GTGCGGTCTACAAGGGTACG	AGTTTGTGTTGGGGAACGTC
130	ICSB0414	CoreSet	1H	528.5	CGAAACAGAAATGCAGGTTG	ATTTGCACCCACATCAGTTG
131	ICSB0956	CoreSet	1H	530.2	GGAACTAGTTGACTCTTAAATTACAGG	GACTATGAACTGATTAAACACTGTGG
132	ICSB0957	Other	1H	532.1	GCAGCAACACATTGCAAAAC	AATTTCGTGACCGACCAGAC
133	ICSB0958	CoreSet	1H	535.2	TCATTTGACTAGTTAATGCCAAACA	CGTTCCCAATCTGAAACGAT
134	ICSB0012	CoreSet	1H	536.9	CTTCAAGCAGCAGCCTTTTTAG	TTGTAATCTCATCGAGAAGCACAG
135	ICSB1228	CoreSet	1H	536.9	CAGATTGTCGATCCCGGTTA	ATGGTTGTTCGTTGTGCTGA
136	ICSB0415	CoreSet	1H	539.8	GCCAGAGCACCCAATAATGT	TGGGAGCACTTCAGATTATGC
137	ICSB0013	CoreSet	1H	551.6	CTAAGAACATCGTCTGTGGCCT	AGAAGCTGGAGATCGTGGACTT
138	ICSB0177	Other	1H	551.6	GTATATCAACCCACAGAGCAAATG	ATGAAGGATATTCAGTCTCAAGGC
139	ICSB0416	CoreSet	1H	551.9	AAATGGCTGCAAAATGGAAG	ATGAGTGCAGCGGCATTATC
140	ICSB0417	CoreSet	1H	556.5	CGTCCATGATTGGTAACCTTG	ATCATCGTGGACGTTTGACA

Supplemental Table 1. List of amplicon sequencing primer sets for barley.

No.	Name	Type	Chr	Pos[Mb]	F_primer	R_primer
141	ICSB1229	CoreSet	2H	2.3	CAGATTTTAGGCACACAGAGTCC	AGATGAGATGAAGCACCGTTT
142	ICSB0014	CoreSet	2H	3.7	AGACACTCAAATTGCCTAGACAGG	CTTTGGTTACTTGTATCTCCAGGC
143	ICSB0107	Other	2H	3.7	GATAGCCTAGAGGAGGAGGTTCAG	CACGGTGTGTGAAGGATGTAAT
144	ICSB0108	Other	2H	3.7	ATCCTTCACACACCGTGAAGTT	ACTTTAGGCATTTGACTGAAGCAG
145	ICSB1230	CoreSet	2H	4.2	CACCATTGTTGAACCTGTGC	CGATCTAAAAATGCGGTGAA
146	ICSB0015	CoreSet	2H	7.3	GAACTTGGCTGAGATGAATTTGAC	CAGAAGTAACCAGCAAAATTCCAG
147	ICSB0249	Other	2H	8.0	TAACCATGTCCATCTTTAACAACG	CTCAATTGAACCTGCAAGTGGT
148	ICSB0418	CoreSet	2H	13.5	GCAGAGAAGTGAGGCCAGTT	TGGAGGACAGTGCTTTGTTG
149	ICSB0959	Other	2H	13.8	AATGAGAAAGCTCCCGAACA	GGGGTATATAGGGAAGCTTATACAAA
150	ICSB0960	CoreSet	2H	17.2	GAGGTTACGCCGAACTGGAT	ATGTCGAGGTCCCGGTAGTT
151	ICSB0961	CoreSet	2H	17.8	TCAGCTGATGAAATGCAATCT	CACGTGTAGCCACCCAAAG
152	ICSB1231	CoreSet	2H	18.8	GGGTGGCCATACTCTGGTACT	CGAGCTTTCCCCTAAAAATAATG
153	ICSB0238	Other	2H	21.6	CAGCAGCTCCATCAATAAAACC	AGACTGCAGACGCTCTCTCTCTC
154	ICSB0016	Other	2H	21.8	CTTAAGTCATGACCTGCAAAGGAT	TGCTTGTATCAAGTTCCTGCTATC
155	ICSB0148	Other	2H	21.8	GCACTCAGGCGATATAAAACATT	GCACTTTGCAGTTTTTGAACTAAG
156	ICSB0149	Other	2H	21.8	CCATCCTCTGGATTAAGTGCTCTA	TGCCAAGGGATCAATAAGAACTAT
157	ICSB0150	CoreSet	2H	21.8	GTACCCTCAGAGGAGTTTATCAGC	TTGGTCATCTGATAACACCAAGAA
158	ICSB0962	CoreSet	2H	21.8	TGGCACTCAGGCGATATAAA	AGTGAAGACAGCGCACTTTG
159	ICSB0419	CoreSet	2H	22.6	TGCTGGCAACAAACTGAAAG	AGGTTGTCCATCCAGACCAG
160	ICSB0244	Other	2H	22.8	TGACTGACACACTGATACATCTGG	ACCGCTATTTATGATTATCGCATC
161	ICSB0259	Other	2H	27.5	CGAAGAGCGATATTTTAGCCATAG	TGGTTTTGTACTTTCAAGTGTTGG
162	ICSB0963	Other	2H	29.7	TGGCCGTGTTCATGTTTTT	TTGTTTGCCCAAAGCCTAAC
163	ICSB0420	CoreSet	2H	35.8	GCGATGCGACAAGGTAAGAT	GCCAGATAAGAGCAGGGATTC
164	ICSB0017	CoreSet	2H	40.3	GTTCTCACACGCACTCAAACTATT	ATTCATGCAAAGATACAGGAGACA
165	ICSB0179	Other	2H	40.3	ACCTTGTGGATCAGAATCCCTA	GTTATAACATGTCAGCAGCACCTC
166	ICSB0180	Other	2H	40.3	CAGCTGTTACTTTAACTGACTCGG	AATAAGAACAGCAACCTTGCCTC
167	ICSB0257	Other	2H	50.4	AAAAATAGTAAGCGAGTGTTTGGC	GCTAATAGCGTATTTTGACGTGG
168	ICSB0213	Other	2H	52.0	TGGCATATGAATGTACTGGATGAT	AAAATTCCATTTAATGCACTCACA
169	ICSB0018	CoreSet	2H	53.9	AGACGACGAGAACTTCGAGAAC	CATTCTTCCTTCATTCGATGACTC
170	ICSB0421	CoreSet	2H	54.6	TCTCGTCGATATGGTCATCG	AAGACACGTTGTAGTGCTGGAA
171	ICSB0422	CoreSet	2H	58.5	AGTAGCAGCAGCGTTTCCAT	TCAACAAAACCACACGGATG
172	ICSB0232	Other	2H	65.9	ATGAACACGTTTAACAGTTTAAAGAC	AAGGTGGCAGTGTGAGTTAATTTT
173	ICSB0423	CoreSet	2H	66.4	TGACAGCCGACGTAAAACAG	CTCAGGTTCCCAGAGAACGA
174	ICSB0424	CoreSet	2H	69.9	AGAGCTTTGATACGTGCACAA	CGTACCTGCAAGCTAACCAA
175	ICSB0425	CoreSet	2H	81.8	CTAATAGGAGTGACTGGAAATGAAC	GCATTTTTCATATTTGCAATGCT
176	ICSB0426	CoreSet	2H	86.5	TTTGCCAACATGTCTTCTGTCT	TCATATGCGAAGGACATGAAA
177	ICSB0964	Other	2H	94.5	CAGGAGTCCTTCATCCTCCA	TGCGTTGGGTCATCACTTT
178	ICSB0427	CoreSet	2H	97.4	TTCCACGCTGAAATTGTTTG	GAATTGCCAATGCCCACT
179	ICSB0019	CoreSet	2H	102.5	AGGATCTTACCGTTCCAGCTCT	GGAGACTGGCTGTAATCAGTGTAT
180	ICSB0428	CoreSet	2H	105.0	CGCTGATTTTTCAGCCATTC	GCATTCGTGACGGAATTTTT
181	ICSB0429	CoreSet	2H	108.0	CCGAATGGGTGAGAAATGAT	AAACAGGCCTCCAAGTATGC
182	ICSB0965	CoreSet	2H	108.6	CAGCAAGAGAGACGGGAAAG	TGCAAGCTCAAGAGGCAGTA
183	ICSB1232	CoreSet	2H	109.0	CTGTGTAGGTCATCACGGAGA	CCGTGCATAGCATACCTTCC
184	ICSB0020	CoreSet	2H	113.0	GGGTTTTGCTTCTGACTCTTCTAT	AGGCAGAGCATTCTTCTTCAGTAT
185	ICSB0430	CoreSet	2H	113.0	AAGTATCAACAAATGGGCTGCT	GCTGCTGAACAGCAACTGAG
186	ICSB0431	Other	2H	119.3	AAACATGAAGGCAGCTTTGG	CATCCATCGCAAGGACTTTA
187	ICSB0966	Other	2H	121.6	ATTTGTTCGTTGCCGGTGTA	AACTCGTACGGGGTCGATG
188	ICSB0021	Other	2H	124.1	GACACTTTGCCAAATAGTTCGTT	GACAGCAGCTTGGTTAAAATATCC
189	ICSB0112	Other	2H	124.1	GTACTGCAGAACAGAACTGCAAC	ATCATACCTCACTCGCTTCACTAA
190	ICSB0113	Other	2H	124.1	AGGATGAAAAGAGAACGATATTGG	ATCATCCATAGAAGCAGAGTCCTT
191	ICSB0114	Other	2H	124.1	TCAGTAGAGGATGAAAAGGAGACA	CCTTATCGGTTAACCTGCATACTT
192	ICSB0432	CoreSet	2H	126.2	CGCATATGTCTTGCGAGCTA	TGCCTGGTATTATGGGGAGA
193	ICSB0433	CoreSet	2H	135.7	GCCGAAAGAGCCCAAGTTAC	AAAGTGGAATTCAGGCTTCG
194	ICSB0434	CoreSet	2H	137.2	AAATTGTCCATTCCCCCATAG	TCGAAATGTGGGAGAGGAAG
195	ICSB0435	Other	2H	140.0	CGACCTCAGCTTCAACCAGT	GTATCGTGCCGGAGAAGTTG
196	ICSB0967	Other	2H	148.0	GTCGAGCACAAGGAAGAAGG	GCTTCTGCCAATGTGGGTAT
197	ICSB0436	CoreSet	2H	150.9	CGTTGGCTGCTGTCTGTAAA	AAAACACTTACCAGCGCACA
198	ICSB0437	CoreSet	2H	155.0	TTTTTCGGGGTAGTTCTACATTG	CGTGTCAATGTGTCATGAGG
199	ICSB0438	Other	2H	167.4	CCAATTAAATAGTGCAAGTGTTCA	ACCCTGGTGGGTGTGGTAT
200	ICSB0968	Other	2H	167.7	TGGCTATGTGAAAGTGCGTTA	CCGCTGTAAGTTAGTCATCAGTG
201	ICSB0439	CoreSet	2H	172.2	CCACTTGAATTTCTGCTGGA	GCAAAGCCATAAGCACTTCC
202	ICSB1233	Other	2H	179.1	CCCGGTATGACATGAGAGGA	CTCTCATCAATGCCAACAAGA
203	ICSB0440	CoreSet	2H	193.5	GACTCACTGAGGCAGCTTGG	TTTTCGCTGCGACTATTCCT
204	ICSB1234	CoreSet	2H	205.3	ATACACCCCCTTCTCGACCT	GAGGTGGGCGTTCATCTTG
205	ICSB0441	CoreSet	2H	206.0	GTAGGTGGCGAGGGTCTTGT	CAAGCCCAGACCCAGAGTAA
206	ICSB0442	CoreSet	2H	212.5	CCCGACGACTGATATTGTCC	CAGGGCGCTAATCTTCATTC
207	ICSB0969	CoreSet	2H	215.0	TTCCAATTCGCATCAAAGGT	AAAATCGCATAGGCCATCAC
208	ICSB0443	CoreSet	2H	224.8	TGGGTTAAGTTAAGGCTATGCAG	TCGTGAGCTTGACTTGTCTCAT
209	ICSB0444	CoreSet	2H	230.2	GAGCGTAGCAAGGGCATATAA	GTGGCCTCCTGTGTTGTTTA
210	ICSB0445	CoreSet	2H	233.9	CCCTACAATTGTTCCCATCG	AACTTCGACTTCTAGGCAAAGC

Supplemental Table 1. List of amplicon sequencing primer sets for barley.

No.	Name	Type	Chr	Pos[Mb]	F_primer	R_primer
211	ICSB0970	CoreSet	2H	236.4	GGGTTTGATCCCCACTACTACA	GGGCGGTCATAGACACGAG
212	ICSB0971	Other	2H	240.6	GTACTCCCTTCGTTCGGAAA	TCCGTCCGGAAATACTTGTC
213	ICSB0446	CoreSet	2H	250.3	GCTCGTGAAATAATTGGTTGC	TTATGCTAAGTCGCCGATCC
214	ICSB0447	CoreSet	2H	257.8	GAAATGGACGGATTCGTGAG	AGATAGACGCCGTCACCAAC
215	ICSB0448	CoreSet	2H	265.0	TGCTCCATTCTCCACTTTCC	CTCCGGAGGCTGTTAGACTG
216	ICSB0972	Other	2H	283.5	GACGTTAGGTAGGGCAACGA	ATCATCCTTCCGTCCTCCTC
217	ICSB0449	CoreSet	2H	293.0	TGCTTTAAGATCTGTACGACACAA	AGAAAACTTTGCCGTGTTGG
218	ICSB0973	CoreSet	2H	307.6	TTCCATGTGTGGCCAATAAA	CTTGGCAAGCAGAGAAGAACT
219	ICSB0974	CoreSet	2H	324.8	GAAGCTTCGGCAACAACAC	CAGCGGCTTCAGGTACAAG
220	ICSB0975	CoreSet	2H	339.7	GAGACCTTCTGCCCACGTT	CTTCGTCGCTCCGATACCT
221	ICSB0976	CoreSet	2H	356.9	TGTTTTAAATCGCGTTGCTG	GAATCCCGATGCACAAGATA
222	ICSB0450	CoreSet	2H	365.7	GGAGCAGAGAGCCATCGTC	AGTATTGCAACCTCGGTGGA
223	ICSB0977	CoreSet	2H	375.8	CCACACACACATGCTTCGAG	GGAAGAAGGGGGAGGACTAA
224	ICSB0451	CoreSet	2H	383.1	CCTTGTTTGCTGCTTCCTTT	ACGCCCCTGTCTTTCTTTTC
225	ICSB0452	CoreSet	2H	392.1	CTCTGCTCCTGGTGATGAGA	TGGCTCTTGAGGCAAGTCAG
226	ICSB0978	Other	2H	392.6	CGAAGGGGGAGGAGAACT	AACAGCGTGAAACCATCAAA
227	ICSB0453	Other	2H	397.8	CACTCGAGGAACTCGTGACA	GAGCTCGTGGCAAAGAAGAT
228	ICSB0454	CoreSet	2H	407.3	AAATCTCGTCGATGCAATCA	GTTCGTTCGATCCTCTCTCG
229	ICSB0979	CoreSet	2H	409.8	CACACAGTTGGGGACCCTAT	CGACTAATCCCATTTTAATTACAGTTC
230	ICSB0455	CoreSet	2H	414.1	TCTTGTTTGGGAGTGTGTGG	GCCTGGTCGTAGGCTCTG
231	ICSB0456	CoreSet	2H	432.9	TGAGTTTGTGAGTTGCGAAGA	TCTGCACCGAGATGTTAAGC
232	ICSB0457	CoreSet	2H	436.6	ACCGAAGCCCAAAAGATACC	GGGGATTGTTTGAATTTTATGC
233	ICSB0458	CoreSet	2H	440.3	TTTTGTGTACACGGCAGACAC	CGAAGAATCTTTGCTACTGGCTA
234	ICSB0980	CoreSet	2H	440.8	GATTCTTCTTCAACCCCACCT	GACCACTGGCATAGGCAGA
235	ICSB0459	CoreSet	2H	454.3	TGCAAGACCACATCAGAGGA	TGCCTCAAAGAAAGCAGCAT
236	ICSB0460	CoreSet	2H	459.1	GCTGCTCAACTACCGCAAG	GCGTACGTACCCGATGAACT
237	ICSB0981	Other	2H	460.0	TTGAAAGCTTCAATCACACAAAA	TCATGTGGGAACAATTGTGAA
238	ICSB0461	CoreSet	2H	467.8	ACTGGGAGCAGTACCACAGC	GCCCCCAAATGAATCTACAA
239	ICSB0982	CoreSet	2H	479.9	TGTCCAAAGCCTACACATCAA	ATGAAAGTTGGCGGTACCAA
240	ICSB0462	CoreSet	2H	482.2	AACGAGTAATAACCGGCATCA	CGTTAAGCGTACCCATCCTG
241	ICSB0463	Other	2H	483.3	TTGGTTTCAAGCTTGGCTTA	TCGCCCTATGGTCTGAAGTT
242	ICSB0464	CoreSet	2H	490.7	CAACCATGTGAATGCCAGTAA	GCGAGTAGAGCATCCTGCAT
243	ICSB0465	Other	2H	496.7	AAGCATAACTTCAGGTAAATTGAAA	TTTGCTACACATCGTGCTTTT
244	ICSB0466	CoreSet	2H	501.3	ATGACCTTCCTCCTCCCATC	GGCTCACCCAATCGAGAAAT
245	ICSB0983	Other	2H	502.5	GCTACATCAGAACCCGCAAG	AATTTTTGGCCTCGAAGAGC
246	ICSB0467	CoreSet	2H	513.4	TGGAACCGAGAGAACATCTG	CCCGACCACATAACACCTTG
247	ICSB0468	Other	2H	523.4	TCCCATGGACATAAAACTTGC	CGATCCTGCATACACAAGTCA
248	ICSB1235	Other	2H	524.4	ACATCGTGCCGGTCATCT	GGCCATCTCCTGTGCAAT
249	ICSB0984	CoreSet	2H	534.4	TGACCGACATTCTTCATTTCC	AAGGTAGGGTCGGACTATCG
250	ICSB0469	CoreSet	2H	539.3	ACATGTTTGCGCGACGAAT	AGGAGAAGGAGCACAAGCAC
251	ICSB1236	CoreSet	2H	542.0	CGCTGAACTGGATCACGTC	GTACTACTACGGCGGGCAGA
252	ICSB0985	CoreSet	2H	543.3	TTATGCACGTGTGGGATAGC	CATGGTTCGAGGCAGTTTTT
253	ICSB0470	CoreSet	2H	544.6	TGTTCCTTGGACAGGGTAGG	ATGTTCCTTTTCCCCTCGTC
254	ICSB0471	CoreSet	2H	550.8	TCACAAACAGTGTCCTTGCAT	GCACAGTGGATGGTAGCAAA
255	ICSB0472	Other	2H	559.7	TAATTCGGTGTGCCTGTCTG	CGTCAGTTTTCCCAATTTCG
256	ICSB1237	CoreSet	2H	567.4	GAAAAAGAAAAAGGCCAAGGA	CACCAGTGTCAGCTGAGGAA
257	ICSB0986	CoreSet	2H	568.4	CAACACCACAATAATTCCACAAA	CATGCCATATTTACCTATGTTTGC
258	ICSB0473	CoreSet	2H	577.7	CAGGGTTGAACGGACATGAT	GACAGATTGCGTTCCTGAGC
259	ICSB0474	Other	2H	580.1	GCGTTCGCCTATATCACTGC	CTAGAAGCAGCACGGAGGAA
260	ICSB0987	Other	2H	585.5	CCACTACATCAACCGCGTTA	ATCCTACGCACACGAAGACC
261	ICSB0475	CoreSet	2H	589.5	GGCCACTCTCTAACCAGTCG	CCGATTCCTTGGTCCATATTT
262	ICSB0476	Other	2H	592.3	TATTGGCAAGCGACAATACG	TCCAATTCGAACTAGCAACTGA
263	ICSB0988	CoreSet	2H	593.5	GTGCCTTTCCCTCAAGATGT	TCGACCGAATTTACATACAGACA
264	ICSB0477	CoreSet	2H	598.8	CAGCGTCTCGTGGGTCTC	TACCGAGCCTTCAGAACTGC
265	ICSB0478	Other	2H	602.5	AACAGCTGCCCTCACTTGAT	GTTTTGGCCATGACTGCTCT
266	ICSB0989	CoreSet	2H	607.2	GTTCTTGTTGCTTCGGCTTC	CGTTAAAGCACCAGCCAACT
267	ICSB0479	Other	2H	617.5	TCAAAAGAAATAATCCCGTTGG	CAGCAAAGCTGCTACAACCA
268	ICSB0480	CoreSet	2H	621.0	ACAAAACCTTCCCTTCCACA	GCCAAGATGACCTTGACGAT
269	ICSB0119	Other	2H	621.1	AAAAGAGAGAGAAGGACTCGTCG	AAAAATTGGTATGGTAACCCCTTC
270	ICSB0120	Other	2H	621.1	GTAGTTCCCTGATGTACGAAGCTC	ACTTTGTGTTTCGTTCTCACGTTT
271	ICSB0121	Other	2H	621.1	ACATCTACTATGCCACTGAGCAAC	CAGCAGAAGTCTTGAAGAAGACAG
272	ICSB0022	CoreSet	2H	629.0	AGGAATGTTAGACCCAGCAGATAG	GGAGTTTCACTTATTCTTATTGCCA
273	ICSB0481	CoreSet	2H	629.0	ACCTGACCGTGTTTGGTCTT	AGAAAGCTGATTTCCCCACA
274	ICSB0990	Other	2H	630.6	GCACAAAAGCAAAGTCACCA	TCCAAGAATTATTTGGTTGATGC
275	ICSB0482	Other	2H	633.9	CAAGAGAAGATGCGATAGCAGA	GGAATCCAGGTTTGAAGACG
276	ICSB0483	CoreSet	2H	639.3	GCAAGACCCTTTTGCTATGG	TTTTGCCACCCCACTCTAAT
277	ICSB0484	CoreSet	2H	647.7	GGACGAAGAGTACCGTGGAG	GCATCAGAGGCAAATGGAAC
278	ICSB0991	CoreSet	2H	654.3	CTTGGACCCACATGAATCAA	GTGCTCTTGTGCGACGTTTA
279	ICSB0485	CoreSet	2H	654.6	TCCCTGCGTAACATAGGTGT	ACCTGTATACCGCCGAAAAA
280	ICSB0486	CoreSet	2H	657.0	TGAAATCCTCGTGCTTCTCT	AATGCTACTGCAGCGTCTAGC

Supplemental Table 1. List of amplicon sequencing primer sets for barley.

No.	Name	Type	Chr	Pos[Mb]	F_primer	R_primer
281	ICSB0487	CoreSet	2H	661.0	AGGGTGGACACCATCAGAAG	CTGACACATTGCACTCACTCC
282	ICSB0992	Other	2H	662.4	GAGCCTCGGGGGTCACTA	AACTATGTGCGGCCAAGGT
283	ICSB0488	CoreSet	2H	667.0	TGTCCTTAGCAGATCTTTGACC	GCATTTGAGGCTTAGCTGGA
284	ICSB0489	CoreSet	2H	670.8	AGTCGGCTTCGTCCTTTAAC	GCTAATTCCAGGTTGCCTCA
285	ICSB0490	CoreSet	2H	674.2	CCATGTGGAAGTCCTCTGCT	GGAGTAGAAGCGCCTGAAAG
286	ICSB0993	CoreSet	2H	688.0	GCCTCATCGATTAATTCAGCA	GGGTTGATTGTACGTTCATGG
287	ICSB0491	CoreSet	2H	688.5	CCCATAGTCAAAGCCAGGAA	GGATGCTCATGGACACGAT
288	ICSB0023	CoreSet	2H	691.3	TTGCAGCTCTGTAGTCATTACCAT	ACTGCTGTGAAGATTGAGAATGTC
289	ICSB0492	CoreSet	2H	692.5	CTGTGTCAACAGCACGTGAA	GATTCGTTGGGTTCAGAGGA
290	ICSB0493	Other	2H	704.4	GCCTCTGGGCTAACATGAAG	CACGCTAAGCTTGACGAATG
291	ICSB0994	CoreSet	2H	705.5	ATCACCGTCCATCATTCGAC	ATCTCGTTCAAGCGGTGTCT
292	ICSB0494	Other	2H	705.7	TGAGCGCTTGCGTCATACTA	AGCGTGCTGATGGGTTTTA
293	ICSB0495	CoreSet	2H	714.1	ACATGTGGTGTGCTTCCTCA	CACTTCCTGCGCTAAATCCT
294	ICSB0496	Other	2H	719.7	GCGGTATTGTAGCTTACTTTCG	TTAATCGGTACCATTCCTTGC
295	ICSB0995	CoreSet	2H	721.0	AAGCGGTCTTCTCCCCTTT	CTCAATAGCGTTCGCCAATC
296	ICSB0996	Other	2H	728.7	GAAATGGGAGGGGGTAAGTT	CCCATAATGTGACGGAGGAT
297	ICSB0497	CoreSet	2H	729.1	GATCACCGTCCTCATTGTCA	ACATTGCGTCTGCTTGTGTC
298	ICSB0024	CoreSet	2H	747.0	TGGCTTGGTATACTCAGAAAAGTG	AAGATGACATCACATCATTTCCC
299	ICSB0103	Other	2H	747.0	GTCACGACTCTAGGTCATTTCCTT	AACCAATTTGTTTCTGTCCATCTC
300	ICSB0104	Other	2H	747.0	TGCTCTGCTTAATCAGTTGAACAT	TAGAAGCAATTGTCTGTCCAAGAG
301	ICSB0498	CoreSet	2H	747.0	CTTTCTGCAGTCGGTGACAG	GGACCATCAATGGGTGAAAT
302	ICSB0115	Other	2H	750.7	GACACCACGTCGACTTCTTCAG	ACAGAGACAGCAAAAGGCAAAG
303	ICSB0499	CoreSet	2H	752.9	ATGTCCTTGAAGGCCTGGAC	CCCATCACCAACACCCTAAC
304	ICSB0997	CoreSet	2H	759.2	GGGAAGGAGCAGCATGAAT	CAATGCCTCATTGGAGCAC
305	ICSB0500	Other	2H	760.0	TTTCGAACAGCCTCTGACTCT	TTGTAGATGACTGTTAATCTCCATGT
306	ICSB1238	CoreSet	2H	760.1	GAGGCCCATTTTCGTGACTA	ACCACCACCACCACAGTACC
307	ICSB0998	Other	2H	761.3	CACCCACCCACATTTTGAC	TTCTCGCATGAGCGAAATC
308	ICSB1239	Other	2H	761.3	CCTTCTGCTCTCTGGTCCTG	TGCACTAATTTTGTTTCTGAAAATG
309	ICSB1240	CoreSet	2H	766.3	ACGCAGAAGCAACCAAGAAG	GTGTGGGTCCACTTCCACTC
310	ICSB0501	Other	2H	767.4	CGCGATGTACAGGTTCACAA	TCCTTGGTAGCTTTCAGCTCTC
311	ICSB0502	CoreSet	3H	0.1	GACCCTTTGCTATGGAGCAG	GTCATGGCCGGAGTGAAG
312	ICSB1241	Other	3H	0.4	TCCATCTCCACCCAGTTGAT	CTGAAGAAGAAGCGGAGCAG
313	ICSB1242	CoreSet	3H	1.1	ACCTTCTGCCGCCTCCTC	CCCGAGATGGAAATGGAAG
314	ICSB1243	CoreSet	3H	1.1	ATGGTCGGCCTTATAAGCAA	TCACGGCTATATTTTCTGTGGA
315	ICSB0999	Other	3H	2.0	TGATGCGCTGATCTTCTTTG	GCAGCTTCAGGTCGTCATTT
316	ICSB1000	CoreSet	3H	2.3	TGAAGAATAGGATGCCAAGGTT	CATGTGCAATACTTATGTCAAATCT
317	ICSB1001	CoreSet	3H	3.6	GCGTAAACAAGGTTCGGCTA	TGGAAATATGGTTGGCCTTT
318	ICSB0025	CoreSet	3H	5.0	GCGATACAGATTATGAAGGGCTAC	ACATCCTCTAGCGAATAGCTTCTG
319	ICSB0151	Other	3H	5.0	AGGGTTATTCCCCAGATAACCTAA	TGCATCCACACACCTACTTTATTC
320	ICSB1002	Other	3H	17.6	CCAGCGAAGTCCATTCAATAA	GAGGAGAAAGAGGCCTGGAG
321	ICSB0503	Other	3H	17.9	CGATCGGCCTCTGCTAGTT	TGAACCTGCATGGACATGAT
322	ICSB0504	CoreSet	3H	22.5	AGCTGCATCTGTGCAGACTA	TCTTCCGGTGCTTTATTGCT
323	ICSB0505	Other	3H	27.0	CCCCACTTGAAAGTTGAAACA	CTGGCTGAACAAAAACAAACC
324	ICSB0026	CoreSet	3H	28.2	TCTTGAATCTGGGTGAGCATATAA	CAAGGGAAAGATGCAAGAAACTA
325	ICSB1003	CoreSet	3H	33.5	TTTGCATTATCAGCAAACTGC	TGTCACCAGGGTTGTCTTGA
326	ICSB0506	CoreSet	3H	37.6	GCTCTGAGCCACTAGACTATCACA	CCACCATGTTCACTTCAACG
327	ICSB0197	Other	3H	39.5	GTTTTGGAGTTGTGCAGAAATTAG	CGCAAATATAAGGAGATTACCTGC
328	ICSB0182	CoreSet	3H	44.2	GAACGAGTTATTGAGCTGGAAGAT	CAACTTCTCTTCAAGGTTTTCACA
329	ICSB0027	CoreSet	3H	44.3	AATCGTCAGTCTGAAGGAAGAAAT	CAGTGGCAAGTTCTTGTCTGTACT
330	ICSB0217	Other	3H	44.3	ATTCTAGATGAAAGGATCCAGACG	GGTGAAAAATAGTCCAATCCTGTT
331	ICSB1004	Other	3H	45.5	ACCGGGGCCCTAAAATATC	TGTTTCGGACCAGGATTGAT
332	ICSB0507	Other	3H	49.3	TACATGTGCGTTCTGCCAGT	AGCTAAACCACCATGGGATG
333	ICSB0508	CoreSet	3H	55.9	CTTGCTGGGTACCATCACCT	GGGGACCATTCTTGGAACTT
334	ICSB0509	CoreSet	3H	58.9	CAGCCACATTGGGTCTTACA	AACCCAAGCACGAGCATTAT
335	ICSB0510	CoreSet	3H	76.1	TCGGGATCATCCTAGACCAC	CATGGATCCAGGCATTATTGA
336	ICSB1005	CoreSet	3H	77.0	TGTTGTTTCTCATCATCATTCTCA	TGAAAACAATTCAAGAGGATGG
337	ICSB1244	CoreSet	3H	82.3	GACAAGGACCTCTGGCTCTG	CTTGTCTGCCTGCCCAGT
338	ICSB0511	CoreSet	3H	95.7	GCACCACCATATAGACACTGC	AATGTTACTCGTACCTAAGAAATGG
339	ICSB0512	CoreSet	3H	101.2	TAAGGTTGGCCTGTGTGGAG	CGAAGAGGACTATATGGCTTGA
340	ICSB0513	CoreSet	3H	107.2	GCCACTTTACGGTCTGTGGT	TCCAAAATGCATAAGCCAAA
341	ICSB0514	CoreSet	3H	109.1	AATAAAAACTGAACAGCACAAATCA	ATGCAGTAACCTTCCCGTCT
342	ICSB1245	Other	3H	113.2	GTCGGCGATGTTGCACTC	GGTGATGGTCGGCGAGTA
343	ICSB0028	CoreSet	3H	119.6	GCGATAGGTAGGCTCTTTAGATTG	GTTGGGTGGTCTTCTTCTTCCT
344	ICSB0515	Other	3H	122.5	GAGCGCTGCTCCATAGTTTT	CGACGCAAAGGACACCATA
345	ICSB0516	Other	3H	130.8	TTAATGCTTGGGGAGGAAGA	ACAAGGTGGGCAGTTCAAAG
346	ICSB0264	Other	3H	135.0	AGGATGGTCATCGGTTAATGTTAT	GTCTTTGCTGGTTAGGGTTCTC
347	ICSB0246	Other	3H	138.8	CTCTAGGATGTGGTCTTTTGGATT	TGCAGAATTATAGCTTCTCGTCAA
348	ICSB0029	CoreSet	3H	139.2	ATACAGTGTCAGCCGTGTAAGTGA	CTTCAGTGACTGCTCCATCCTC
349	ICSB0256	Other	3H	141.1	TCTGTACAAGAAAAAGCAACCTTC	TAGTAACTTGAGCCTGCATCTGTC
350	ICSB0517	CoreSet	3H	141.6	AATCAAACTGGTTGCTCATGG	TCTAGCCACTTGGTAGTTCTTGA

Supplemental Table 1. List of amplicon sequencing primer sets for barley.

No.	Name	Type	Chr	Pos[Mb]	F_primer	R_primer
351	ICSB0518	CoreSet	3H	148.4	CGGGTCGACTGGATGAGTAG	TCGACATGAGATTCATTTTTGG
352	ICSB1006	Other	3H	155.0	AAAACATATAACAATGTCACCCAAAA	GCTGAAAATAAGCTTGGACACC
353	ICSB0519	CoreSet	3H	155.1	CCAACAGATATAAAAGAGACATGTGG	ATGGTCAAGTTGCCAACCAT
354	ICSB0520	CoreSet	3H	161.1	CAGGATGTTATGAATTGTGATGC	CAACAAACTGCTGTTTATGCTGA
355	ICSB0521	CoreSet	3H	165.0	GAGCGCGCGTATTAGAATTT	CGTCGTTCTCCATTCCATTT
356	ICSB1007	Other	3H	171.0	TTTAACGACGAGGCACATGA	GATGCACTCCCCCTCTTGTA
357	ICSB1246	CoreSet	3H	176.5	ATAGGCAAGCCAAGTGCTTC	GGTGTTCGGCAGTGACCTAT
358	ICSB1008	CoreSet	3H	191.5	TCAACTCTCCATGCTATGCTG	CCCTGGAATGACGGATAAAC
359	ICSB0522	CoreSet	3H	195.6	AATTCTGCGCCCAGGTTG	AAAAGACGACGCACCACAG
360	ICSB0523	Other	3H	198.2	TCGCTGTCATATAATCGAGGTG	TGTCAAGGCATTGCTAGTGG
361	ICSB0524	Other	3H	202.3	TGCCATCCACGAGTAATACG	TTTGTGGAAAAGTATTCATAGCAA
362	ICSB0525	Other	3H	207.3	TTGGACCAATCATTCTCTCC	CTTTTCCCCGTGAAACACCT
363	ICSB1009	CoreSet	3H	210.5	AAGAGTAAGGACGGGGAGGA	GCATATGGAGGCATCTTGAAA
364	ICSB0526	CoreSet	3H	212.0	CATGTAATCCCCTCAGCTATTG	GGAGAAGAGAAATGAGCAGCA
365	ICSB0527	Other	3H	216.2	ACACTCTCTCCTCCCAAGCA	TGCTCCTGATATCTCTGATCCTT
366	ICSB1247	CoreSet	3H	226.4	AGGAATGGGCGTTGGAAT	TCTGCAGAAGCAGAACTAGCA
367	ICSB1010	CoreSet	3H	229.5	TGGAACAAGCTCTTCGGAAC	TCTTCGAAGGGTTGGAACTG
368	ICSB0528	CoreSet	3H	234.0	CCAGGAAGAACCCATATCGAA	GCCATGGACATCATTCAGTG
369	ICSB0529	Other	3H	238.6	CGAGGATCTTCCCTGCTGTA	GCAAGTCGTTGGACTCTCCT
370	ICSB1011	CoreSet	3H	251.9	TGCTTGTGTTCAATTTGCTG	AACCAAGCTGCAGGGATCTA
371	ICSB0530	CoreSet	3H	254.5	TCCTGTCCCCTTCTTTTGTG	ATTCAAGCGGCCTACAGCTA
372	ICSB0201	Other	3H	254.8	ATTTTTGTTCTCCTCTTGTCTTGC	GAAATTAGTAGCTCCTCGAGGTCA
373	ICSB0531	Other	3H	256.3	TTTCTCACTGTGCTGGATTGA	TCATGCCACAGAGTTTTGGA
374	ICSB0532	Other	3H	267.7	CGGGATCAGCCAGACAATAA	CTGGCTTCGGTTTGATGAAT
375	ICSB1248	Other	3H	269.3	TGTGTACTAAAAAGAAATAGCTTTCG	CTGCTTCAGAGGATGATGGT
376	ICSB1012	CoreSet	3H	272.4	TGTGTGATGTAGCCCCCAAG	CTGGCACAGACTCAACCTCA
377	ICSB0533	CoreSet	3H	279.1	TCATCATTTTAGCGCACTCG	CCGATATGCAGGTGATCAGA
378	ICSB0534	CoreSet	3H	287.8	CCTCTGATGCGGGCATATTA	CCAGAATCAGTAAAAATCGAACA
379	ICSB1013	CoreSet	3H	289.8	TTCTAAACGCAAAGGTTGAGC	GGAGTGCTCGGGTTCTATCC
380	ICSB0535	CoreSet	3H	292.1	GTGAGCAAGTCCTCCCAATA	AATCAGGCGCCATCTCAG
381	ICSB0536	Other	3H	296.7	AATGACAATTGCGTGCAAAA	GCGCATCATTTTTGGAAGAG
382	ICSB0266	Other	3H	299.4	GATGATAATGTCTACTTTCTGCACG	TTCTTCACTAAACACCAAGCTTCC
383	ICSB0200	Other	3H	302.8	TTCCGTTAATAATTATCCCTGTCC	ATTGAATGTGAGGCTGATTGTACT
384	ICSB0537	CoreSet	3H	302.8	CCATCTGTTCTCCCTTACGC	CATAAGCTGGCATGCTTGG
385	ICSB1014	CoreSet	3H	310.0	TCACACCCCGTTTTTACCTT	CATTCAAGCACACTCACAAAAA
386	ICSB0538	Other	3H	315.7	ACCGTGAAGGCAGCTACAAT	TGTTTCCAGCCTTTTCTCGT
387	ICSB0539	CoreSet	3H	319.5	CACGCTGATATGTGGGACAC	TGCTCGTAACACAATGGTCCT
388	ICSB0540	CoreSet	3H	324.4	GCAGAATGCTATTTGTCTGGTC	TGCAGAAATGTCACGCAGAT
389	ICSB0541	Other	3H	329.3	TAGTGTCGCACCTGTCATCC	TGATTGTCGGCAGCTTACTG
390	ICSB1015	CoreSet	3H	330.9	TCGGGAGATAGTTCTAGCTGGT	GAACTAAGCGCTTCCACGTC
391	ICSB0542	Other	3H	337.0	GGAGAAACCGGTCACGTAAA	CGGGTTGTGCTAGAGTTGGT
392	ICSB0543	CoreSet	3H	341.9	CGTGTCTGTAACCACTGAATGC	GCGAATGGAAGATAAGTTGAGC
393	ICSB1249	CoreSet	3H	343.6	AGGGTAGAGGGCAGTGGTG	GGCTCCATCATTGTCGACTC
394	ICSB1016	Other	3H	349.7	AAACCTAGACCGAGCAGCAG	TCGCACTCATGTGCATAAAA
395	ICSB0030	Other	3H	351.9	AGGTGAGGTTTGTGGAATTCAT	CACTTCACAAGATAACACTCCAGC
396	ICSB0544	CoreSet	3H	352.1	TATGGTTCCTCTTCGCGTTT	GGGCAGAACCAGACACAAAA
397	ICSB0267	Other	3H	353.0	CTTGCAAGATCAAGATCAAAGAGA	AGTACTGCTTGCTGCTACCCTCTA
398	ICSB0545	CoreSet	3H	355.4	TCCTCGCAGGAAAGAGTAGC	CTAGTCGGGAAAGCCATGTG
399	ICSB0546	CoreSet	3H	361.8	ATCCACGATGTGCTGGATG	CCTACAGATTTCCCCAAGCA
400	ICSB1017	Other	3H	369.5	GGGATTATTTGCAACCCAAA	TTGGAATCAAATCTCTTTTCAAAT
401	ICSB0547	CoreSet	3H	370.2	ACTCGATGCTTCCTGGTTGT	AGCTCCAATGACACCTCCAT
402	ICSB0548	CoreSet	3H	376.2	CGCAACTGGAGGATCAAGTA	TAAATGGCCCAAATTTCACC
403	ICSB0549	Other	3H	379.5	GGGTCACACCTGCTTTTGTT	TTCGCGTCCAAGGACTTATC
404	ICSB0550	Other	3H	381.2	TGCCTAAGGATGCCATATCA	TGATGCTGAGAAGGTGATGG
405	ICSB0551	CoreSet	3H	386.8	TCTCTGTTCTCGCCTTAGCAG	GCAAAATTACGCCTCAGGAA
406	ICSB1018	CoreSet	3H	390.1	GTCAAAGGATGCCTTCCAAA	TCAATGGTACCGAGGTGAAA
407	ICSB0240	Other	3H	390.6	AACCAGCCAAATCATGTGTTAGT	CTGTCACTTGCATCTTGGTAAACT
408	ICSB0190	Other	3H	394.0	TAATCAACACTGTGGAGATCAGGT	GTTTCATGAACCAGTTCCTTCTGT
409	ICSB0552	CoreSet	3H	396.2	AAGCCCTGTTTGACAACATAA	CATATATCGTGCCCCACTGC
410	ICSB1250	CoreSet	3H	399.3	AGTCCAAGGCCAAGTCAAGA	TCCGTTTCCTCTCGTGTTCT
411	ICSB0553	CoreSet	3H	399.6	TGTAGGCGGCTTTAGGTACA	GCTCCGATGTTGCCATCTTA
412	ICSB0554	CoreSet	3H	405.7	TGAGCGTCTGGTTTATCTGG	AGTTCATTTGCCAGCAGCTC
413	ICSB0555	CoreSet	3H	408.8	TGACCCTTTGCATACACCAA	GTGAAATGCTATCCGGTGGT
414	ICSB0203	Other	3H	409.5	GCCGTTAAGTACAGGCTCCTAC	CCTCCTACCTCTCGATCTTAACTC
415	ICSB1019	CoreSet	3H	413.6	CCAATTCAAAACCATGCAGA	GACACGCGGAGAGAAAACTC
416	ICSB0556	Other	3H	413.8	TGCTGCAATTTTAGGTCCTT	GCAATGCCTACAGATGTATTTTCA
417	ICSB0207	Other	3H	416.6	TCTTTTCTTTTTGTGTAACCAATCTG	CGAGACTGAACATGAAGATGTATG
418	ICSB0557	CoreSet	3H	417.3	GCAAGTCTGCAGCCATGTTA	TTGAAGGAAGCCCTCCTGAT
419	ICSB0269	Other	3H	421.0	CTCGTTTCCTCCTTCTCCAAAA	GGAGAAGTAGTGACTCTTTCAGAACA
420	ICSB1020	CoreSet	3H	429.1	CTTCCATGAGGGATGCATGT	CTCGGTTCCCCCTTTGTTA

Supplemental Table 1. List of amplicon sequencing primer sets for barley.

No.	Name	Type	Chr	Pos[Mb]	F_primer	R_primer
421	ICSB0192	Other	3H	432.1	GGGACACATGCAACTAAATTGTTA	GACTCGGCATATAGCTTGTGTTG
422	ICSB0558	CoreSet	3H	441.1	GCTCCCAGACCAATCAACTC	CTTCAATAGTTGTCGCCACTG
423	ICSB0559	CoreSet	3H	444.7	ACGTGGAGTGCAATCAGTTG	TCAGCCAGCTGAAGTTCTTG
424	ICSB0560	CoreSet	3H	447.5	GTTCTGCCACTCTGCCAATC	GGTCGATGGCTTACTTTTGC
425	ICSB0561	Other	3H	454.0	ACAATGGGGCATGAATTGTT	GTCCTTTTGGGCAAAGCTC
426	ICSB1021	CoreSet	3H	456.0	TGCCAATAAGGAAGCAGACC	CCACCATAATCATGACCCAGA
427	ICSB1251	CoreSet	3H	461.6	CTAAGTCTGGATGCCAGCAA	ACTTCTTCTGGCGCTGCTT
428	ICSB0562	CoreSet	3H	463.2	TTCTAGTTGATGCTGCACTCTGA	TCCACAAATGAGCCCTAGACT
429	ICSB0563	CoreSet	3H	468.3	GGTGCAGGTGCAACTACTGA	GCAGCTGGGCTGTAGAAATC
430	ICSB1022	Other	3H	470.9	CACAAACTGAGCAATCTCACG	ACGAAGCCCACAATTCTCTC
431	ICSB0564	CoreSet	3H	472.7	TGTCACTCGGAATAGCAGCA	GACCTCGCTACCGGAAAGTT
432	ICSB0565	Other	3H	485.6	TTTGCGAACACAACTCGTTC	CAGAGGTGCAAACGGAATTT
433	ICSB0239	Other	3H	491.4	GGCTGTACAAGACACAACAAAGAG	ACTGCTGTCACCAAGACTTGAAC
434	ICSB0229	Other	3H	493.1	GATTAGACGAGCCCTCTTTCTTAG	TCTCCCTTTTGATGCATAGAATTT
435	ICSB1023	CoreSet	3H	493.7	CGGAGTGCGAGAGAAGAAAT	GAGGTGAAAGCCTTGCTCTG
436	ICSB0031	CoreSet	3H	497.6	GAGAAGGGAAAGCATGAATCTCT	ATATATCTGCACATCCCCTTATGC
437	ICSB0145	Other	3H	497.6	AAGAACGGTGAGTTCTAAAAGTTTG	TATGAGCACACCTGCTCAAGCTA
438	ICSB0146	Other	3H	497.6	CATAAATGACAGGAAGAACATCCA	TGCTAGAGGAAATTGGTATCACCT
439	ICSB0147	CoreSet	3H	497.6	GAGAAGGGAAAGCATGAATCTCT	ATATATCTGCACATCCCCTTATGC
440	ICSB1252	CoreSet	3H	497.8	TTTCATTGACAGGAAACATGC	TCCAGGTTGTTCTCAACTTCA
441	ICSB0566	Other	3H	499.4	TCCTCGGGAATAGCTCTGAA	TCAGTGTTCATTTGGCAACC
442	ICSB0567	CoreSet	3H	504.0	GTGCCAAACTTCATCCTTGG	GCTGGCCCAGTTTTCCTACT
443	ICSB1024	Other	3H	505.7	TGAACCCTATTTATCCGTTTATCC	GATTCATCGCCAATTGAAGG
444	ICSB0568	CoreSet	3H	508.8	CACGATCGTTCGATCATTCA	AGCAGAGGATCAAGGCAAGA
445	ICSB0569	CoreSet	3H	513.0	TTACATCTGGGCTTGCTCTG	ACACAACAATCTGCCCCTCT
446	ICSB0032	CoreSet	3H	513.4	CTCCAGAGATACCTTGGTTTGTTC	AAACAGATTAGAGCAGCTGGAAGT
447	ICSB0163	Other	3H	513.4	CACTTTGAGTTTGAGGGTTTCTTT	TCCTCGTTCAAAGGATATTAGGAG
448	ICSB0164	CoreSet	3H	513.4	TCTCTTGCTACATTGGCTTCATTA	AGCAAAGGAAGAGAATGACAAACT
449	ICSB0570	CoreSet	3H	517.5	TTTTTGATCTAAACCGGCATT	GGATCATATGTTTCCCCAAGG
450	ICSB0571	CoreSet	3H	521.9	GATTGGCAATGGTGCACTT	ACTGCACCAACCCATGAACT
451	ICSB1025	Other	3H	528.8	TCCGAGCAGAGCAGGACT	CAAAGCACGGATATGAACGA
452	ICSB0033	CoreSet	3H	532.2	CTCCAGATCTGCTTCTGTTATGAA	TGCACTATGGAGTTTGATTACGAT
453	ICSB1026	CoreSet	3H	533.4	GCCTCAGTGTCCGAAGAGTC	CAATGACTTGAGCCACATCG
454	ICSB0572	CoreSet	3H	535.5	TATTCTTTGGTGCCGGAGAG	TTCTCTGATGTGGCAAAAGC
455	ICSB0215	Other	3H	542.0	GTGTGTTGCTACTGAGAGGAGTTG	CAAGATGTATCAAAGACATCCAGG
456	ICSB0573	CoreSet	3H	549.1	TCAGGGCGAATTTAATCTGG	CATCGTGGGTGTGACTGAAC
457	ICSB0574	CoreSet	3H	553.6	CAGCAAGCTTCTCAAAATCC	TTGGAGTGGTTCAAATTCGTC
458	ICSB0575	CoreSet	3H	558.0	TCTCCAGCACTGATGGTTCA	CTGTGCTGTCAGAGGTATCATCTT
459	ICSB1027	Other	3H	561.1	TTGAACATGTCATTGCACTCAA	TCGCAAGTAACCCATGTTTG
460	ICSB0576	Other	3H	566.7	CCCGCACTATGTGATTTGGT	TCAAATGATGTGATCTTCCAAAC
461	ICSB0034	CoreSet	3H	568.0	AATTGATGGCAAATGCAAGAGTAT	ATAGCATTTCTCCTGGATGTATGG
462	ICSB0128	Other	3H	568.0	CTGAAGAGTGTGTTTGGATCAGAG	TCCTCCATCCAGTCTTCAAAAATA
463	ICSB0129	Other	3H	568.0	AAAAAGCTTCGACTGGGGGTAG	AGCTGTGGAAGTCATTCTCATCA
464	ICSB0577	Other	3H	574.9	TGGGTGCTGAGTAACAATCATC	TGGGAACCAAGGTTTGTTTT
465	ICSB1028	CoreSet	3H	582.3	TCCTGAGCAGAAACTTCGTGT	ATACATCCAGGCCCACCAAC
466	ICSB0578	CoreSet	3H	589.5	CATAACTAGCAACCAAGACCTCA	AAATGCTGAAGCACCAGAAAG
467	ICSB0579	CoreSet	3H	593.2	CGAGCCTGATTCTGGTTGAT	AGCGGATTTGAAAAACGATG
468	ICSB1029	Other	3H	599.9	CCGGACAAGTTCTCTCGTTC	TCGAAACTCCCTGACCTCAC
469	ICSB0580	CoreSet	3H	602.7	CGAGGAAGTTTGACCTGGAA	CCTACATTGGGAAGCTGAGG
470	ICSB0581	CoreSet	3H	605.2	ATGGAAGCGATGAAGGAAGA	ACAGCTCTGGGTGCTTGATT
471	ICSB0332	Other	3H	608.7	ACGAATCCAACAGTACTTGAAAGA	ATGTGCGCACCTGTATAGTGTTAG
472	ICSB0296	Other	3H	608.9	AAAGTTCACCACCCTTCTTCATC	CAGTGTAAGCACCCACACACTATT
473	ICSB0326	Other	3H	608.9	AGCAAATTTGTGAGAGTTTGCTC	CCTCTCATCTCCTCAATTTGATCT
474	ICSB0582	CoreSet	3H	612.5	GCGTAGTTTTTGTGGCAAGA	CAACAGGTTCCCCACTTTGT
475	ICSB0035	CoreSet	3H	616.2	ATCTCACTTCATCTGCATCCAAGT	ACAAGATAACAAGGTTGTGGAGGT
476	ICSB0583	Other	3H	616.5	TGAGCAAACCACACTTGCAT	TCAGGGCTTGTAATGGTTACCT
477	ICSB0221	Other	3H	616.6	TGACAAATTCTTTATCATGAGGGA	ATAAAATGGGAAACATGCTATCGT
478	ICSB0222	Other	3H	616.6	CTAACAACACACTGATCCTCGTCT	GTGTCTCAACTTTGTACAAGGGAA
479	ICSB0584	CoreSet	3H	622.0	AGGTACTGCACCAAAATTGC	TGAAAGGGGAACCTAAAGCA
480	ICSB1030	CoreSet	3H	634.3	CTCGAATCCGACCACATCTT	TGTGGACATGCGTCCAATTA
481	ICSB0585	CoreSet	3H	634.5	CTGGAACTCCCGGTTGTTAG	CAGGGTTTTTGCCCATAGAA
482	ICSB0586	CoreSet	3H	640.0	TGGCTGCACAAGTACAGATTG	AGCAAACCAATCCACCCTAA
483	ICSB0587	CoreSet	3H	643.3	TCCATGCTTCGTGGTACAGA	TTTTCACAAATGCCAAGCAG
484	ICSB0334	Other	3H	643.8	CTTTGGCACTTCTACAATGTGTTT	AGTCGTGTGTGCTGTTGTTTTCT
485	ICSB0301	Other	3H	644.7	TATTGGATTTCAAGGTTGGAAGTT	CCAGGTGCAGATAGTTTTACTGAA
486	ICSB0308	Other	3H	645.2	TTGCTTTCTGTTGAGACGAATAGA	CGGTTTTGCTAATCCCTTCTACTA
487	ICSB0036	Other	3H	645.5	GGTGTTCAACGAGATCAAATTC	GTGTAGAGCGTGTTGAAGTCCC
488	ICSB0175	CoreSet	3H	645.5	GGTGTTCAACGAGATCAAATTC	GTGTAGAGCGTGTTGAAGTCCC
489	ICSB0176	Other	3H	645.5	GGGACTTCAACACGCTCTACAC	GTACACTCGCCACTCCTTCATTAG
490	ICSB0312	Other	3H	660.7	TCATGCATAGCTTATCATCACATC	CAATCAAACCCATCCAGATAATTT

Supplemental Table 1. List of amplicon sequencing primer sets for barley.

No.	Name	Type	Chr	Pos[Mb]	F_primer	R_primer
491	ICSB0588	Other	3H	661.5	TGACTGACCAGAGTCAGATTGAA	CAACCGTTTTTCGTGTTTGA
492	ICSB0331	Other	3H	663.0	CTCACGCCTCTTATCCAACTTATC	CTAGCAGGTGTTGTCCTTGTGTAT
493	ICSB0037	CoreSet	3H	664.6	GTAGCCAATGATCACCGTTCTACT	TCTACTGTGCATATTAGGATCACG
494	ICSB0181	Other	3H	664.6	TCAACATGGTTGAGCATTATTAGG	TTAAGCACTTCAAAGGATGATGAA
495	ICSB0349	Other	3H	664.6	GGAGCCTACTATGACAGAGGAGAA	CTGGAAAAGAAGAATACCAAGAGG
496	ICSB1031	CoreSet	3H	667.2	CCCAAACATTGCCTCAGAAA	CAAACAATGAAATGCGGTTG
497	ICSB0333	Other	3H	667.6	TACAGAGCTGATGATCACCCTAAG	TAAGGACGAGCATATAAATGCCAA
498	ICSB0342	Other	3H	672.9	ACACCAAAATACATGTCCATTGTC	CTCGAGGTCTGTATCAATTACACC
499	ICSB0307	Other	3H	673.8	GTACAGAAATTCTTGCACAGAGGA	CTTAAGCTTTTTCCTCACCAAGAG
500	ICSB0350	Other	3H	674.8	CATGCAAAGTGAAATTCGAAATAG	GGGATGCAACAAGTACTACGATT
501	ICSB0310	Other	3H	675.9	TAGCACCACAAAGTAGCCATCTTA	CCTCAGTGTAACGTGTACTGACAA
502	ICSB0589	Other	3H	676.0	GACTTAGGCATCCATGTTAGTTAGC	GAGTGGCAACCTCCCATTTA
503	ICSB0590	CoreSet	3H	679.4	GTTCGATGGAAGCCCTCATA	ATGAAGAGGCTGTCGAGCAC
504	ICSB1032	Other	3H	679.4	ATGACGCAAACTGCAACAAC	AGTGGCACAACTGTCGATGA
505	ICSB1253	Other	3H	680.2	CTATCCGATCCCGACTTTGA	GAGGGAGGGAGAGAGGAGAA
506	ICSB0209	Other	3H	681.7	GGCATTTGTACTCACAAGACAGAC	AGCCAGATTTGTAGTTGACAAACA
507	ICSB0314	Other	3H	681.7	GGCATTTGTACTCACAAGACAGAC	AGCCAGATTTGTAGTTGACAAACA
508	ICSB0339	Other	3H	683.0	ACAGAACACTGATTGCACATTACA	CAAGGAAGCTTTAGCCTTTAGGAG
509	ICSB0591	Other	3H	683.7	AGGAGGGTAACCTGGGAGTG	CTTGGGGTGAACGTCATTG
510	ICSB0305	Other	3H	685.0	TTACCATATCCATGTTTGTCAAGTT	TTCAAGGAGCAGGCTAAGAAAATA
511	ICSB1254	Other	3H	685.8	CGCTGTACAGTGAGGATGTCA	GGCCCGTTTGGCTATAAGA
512	ICSB0319	Other	3H	686.6	CCGGCTACACCAAGCATATACA	CGAGCATTGCTTAAGTACTCCATT
513	ICSB0302	CoreSet	3H	687.5	AGAAACTGGCGTTGAAGGACAC	GTAGCTGTAGACCATCTGCTCCCT
514	ICSB0592	CoreSet	3H	688.4	GAGAGTCATCTGGCTCCACTTC	GATGATGGACAATCTTTGTGGA
515	ICSB0318	Other	3H	688.7	CAAAACCTTCTTGAAGTGTGTGAC	CTTCTTGCCGTGGAAGTAGAAGT
516	ICSB0325	Other	3H	690.3	TCTCTGGTACTGAAACTTGTGGAG	TCACAGCCTGTCAAGTACAATCTTA
517	ICSB0210	Other	3H	692.3	GGCTTTCAGTCTCAGGTAGGTTAC	TGTGGAGAATGAGTAATTACAGGC
518	ICSB0316	Other	3H	692.3	GCTAAATTCTGTCGGGAAGATAAA	TTCACATCATTTGACAACTCATCC
519	ICSB0195	Other	3H	693.4	CTCTGCTCGGGTACATACACATT	GAATCACCATCGGTCGTTAATAC
520	ICSB0300	Other	3H	693.4	GAGTCCAGAGGTCATGGTTAGATT	ATAGAAAACGTGTGTGTCTGGAAA
521	ICSB0248	Other	3H	694.6	CACCGTCTACCTCCCATAATAACT	CTGCTCAGCTTCTGATGCTTCT
522	ICSB0335	Other	3H	694.6	CTGATGCTTTATTTTAGCCCAAAG	CTGGTTGTGTCTCAAGTTTTGATT
523	ICSB0243	Other	3H	696.0	AAATATTCTGCGTGACAACCATTA	AGAACAACCTGTAAAGAGCACCTG
524	ICSB0330	Other	3H	696.0	TTTCCATGTCTCACCATTATTAGC	CCGTCACCAATCTAATATTCCTCT
525	ICSB0251	Other	3H	696.3	AATGCTTTAGCAATGTGTAACAGG	CAAAGTCCATCTGAATTCTCAAGT
526	ICSB0321	Other	3H	697.6	TATTTGAGATTATTGACCGTGTGC	AGTTGGGTAGCAGAAATCAACACT
527	ICSB0317	Other	3H	698.5	CATGAATTTAATCTGATGGAACGA	TTTTTGTATGGACGACTATGATGG
528	ICSB1255	CoreSet	3H	699.1	TGGGTCAGTCATCATCCTTG	TGTCACCGCTCTCGTAGTAGAA
529	ICSB0297	Other	3H	699.2	AGGAGAAGAAATCGAGGAGGAG	ATCTTTGGAGGAGTTCCTGGCT
530	ICSB1033	CoreSet	4H	0.1	GGGCAACAACCTGTCAATGT	CAGAGGGCAGGTTTGAAGAG
531	ICSB0309	Other	4H	0.6	GGAGAGATGTGGATGGTAAATGAT	CCATTTCCTCAATCTTGGTATCTT
532	ICSB1034	Other	4H	0.6	TCACTGCAAGTGAAGGGAAA	TCGAGCAGTAGAGGAGAGCA
533	ICSB1256	CoreSet	4H	0.6	ACCAACTGTCCTGCCATCA	TTTTGGGCAGAGACGTCAG
534	ICSB1035	CoreSet	4H	1.1	ACAACGGTGAATCATGAACTT	TGTTTCCCCTCTACATTTTCC
535	ICSB0313	Other	4H	1.8	CATTCAAACATAAACAGCCGTAAC	TGACTGTACAAGTCCTGTTTCTTG
536	ICSB1036	CoreSet	4H	2.3	TGCCACAGAAAAATCAAAGAAA	GGTTCTTTGGTTCATGGGATT
537	ICSB1257	CoreSet	4H	2.3	TGGAGGAGGAAGAAGGAGGT	GTGTGTGTAGCCCGGAGACT
538	ICSB0322	Other	4H	2.8	ACGAAAGCTTATGGCAACAAAT	CTGTGTACCACATACTCTTCCTGC
539	ICSB1258	CoreSet	4H	2.9	GCGTGCGACGAGAGTATAGA	CCTTGTCGGCTTTCTAACCA
540	ICSB1037	CoreSet	4H	3.2	AGCTTGGCTACCTGAAGCTG	GGTGAGGCAGCATGTCAAC
541	ICSB0298	Other	4H	4.2	AGCTCGACAGTGAGATCAAGTCAT	ATAGCATACCTCATCCTTTTGACC
542	ICSB1259	CoreSet	4H	4.2	TTCGAAGCTCTACAGCAGCA	TACCGTGGCTGTGGACATAA
543	ICSB1038	CoreSet	4H	5.4	ACCGTGTGCTTAGGGTGTTT	CCCAATGACGTATATATCAGCAG
544	ICSB0306	Other	4H	6.2	GTGTGTGATCTCTCCTTGTTGTG	ACAAAGGCATGATTCTATGCTACA
545	ICSB0327	Other	4H	7.4	ATGATCGGACTGCAGAAGAGAG	TTTTTGCATGGTATCTTGTTGTCT
546	ICSB0345	Other	4H	8.1	CTACATGGCAGGGCTAGAGTATG	AGAAATAAAAATGACTGGCACGAT
547	ICSB0324	Other	4H	8.8	AGATTGTATTTGTTGATGGTTTTCC	AGCTAATACTCTCTGAAAAGAAGGC
548	ICSB0593	CoreSet	4H	9.1	TCCCAAGTGCAGCAAGATAG	CTTTGACAGGGAATGCTCGT
549	ICSB0227	Other	4H	9.7	CTGCTATCTTGATTTGGGCTTAAT	TAGACATGCTGTGGCATATAGGTT
550	ICSB0038	Other	4H	10.0	ACCTTATTCTATTGCATCGTTGTG	CGTCTTCAACTTCGTCTACTTCAT
551	ICSB0346	Other	4H	10.8	CGAAGCTTGTTTCTCTCTAGTTGA	GCAGGAGAATCCCTTAGGAAATAC
552	ICSB0323	Other	4H	11.9	GCCCTACTACCCAGAAGTGTTAAT	GCATTTTCTTGTAAGTTATGGGCT
553	ICSB1039	CoreSet	4H	13.7	AACTTGCACAGCATGGTCTT	TCCATTGCATCAAACTATCG
554	ICSB0347	Other	4H	13.8	TGCAACATTATCCACCTTAAAAGA	ATCCCATTGTACATAATCCTCTGG
555	ICSB0594	CoreSet	4H	13.8	AGTCCCTCCATCGTTCCTTC	GCTTGGATTGGAAGTTCTGC
556	ICSB0328	Other	4H	16.5	TTACTTTGCTTTCTCAAAACTTGG	CTTGCAAGGTACTGAATCGTCAT
557	ICSB0315	Other	4H	17.9	GCCAGTTGCTAATGAACGAGATA	CACATGTGAAAGAGAATGAAGAAAA
558	ICSB0311	Other	4H	19.0	TTTTCATGAGATTTTCTCCATTTT	TTTGGTTTCAAACAGAGTTTAAGAAA
559	ICSB0299	Other	4H	21.4	TTGCAACGGGATATAAACATTCTA	TCAAAATATAAGGTAAATCACGGG
560	ICSB0303	Other	4H	22.2	AGCAACAAGGGTTCCTCTTCTAC	AGGCTTCTGCTTTCTCTTATAGCA

Supplemental Table 1. List of amplicon sequencing primer sets for barley.

No.	Name	Type	Chr	Pos[Mb]	F_primer	R_primer
561	ICSB1040	CoreSet	4H	22.4	CCGGAAGTTCAATCTCTGGT	CCGCTCCTTCTCCTTAACCT
562	ICSB0340	Other	4H	23.6	AAGAAGCATCTTATCGAGCACTTC	TATATTTGGTGCTGATGAATTTGG
563	ICSB0344	Other	4H	25.9	GCAATCTACTAGTGCGAGAAGTGA	TGAAGACCCGTCTAGAACATGATA
564	ICSB0336	Other	4H	26.7	ATCATCTGACATGGCTTCTTCTTC	AAGAAAATGGTAGGGATCATGCTA
565	ICSB0039	CoreSet	4H	27.3	AGAAAAGACCTGCCAGTAAAGGAG	ATCATGCTCATCACGCTTCATT
566	ICSB0132	Other	4H	27.3	GTTTCCATGGTGACGATGTATCTA	AAGCATCATCCACGAATAAGATTT
567	ICSB0338	Other	4H	30.7	TATACATCAACCATAACCCAGCAC	TCAAAATACAACAACATGCTTCCT
568	ICSB0595	CoreSet	4H	31.2	CAGATGACACATGAACATACCAG	TCAAATGCAGGACTCCCTGT
569	ICSB0348	Other	4H	31.8	ATGAAGGACAAGCTATTCACCATC	TAAGCATCAGGTTGTTCTTATTGG
570	ICSB0320	Other	4H	33.0	GACTCACAAAAGGCTAAGGACATT	TTCTCTTGTTCCAAATCTTCATCA
571	ICSB0329	Other	4H	35.0	ATCTTTTCGTCGTCGGTTAAACT	CCACGACTCAATCCTCTGAAAC
572	ICSB0304	Other	4H	36.2	TATACCAAAGTCTACGACCAACCA	ATTGTGATGGTTGATTTTTCATTG
573	ICSB0596	CoreSet	4H	36.2	AGATCACGTCCGTCTCCTTG	TTCGAGTTTGAAGCCGAAG
574	ICSB0343	Other	4H	36.7	GTCTTCCGTTTCTTATCATCAACC	CTCGTTTGTGTCCATTATTGAAGT
575	ICSB0597	CoreSet	4H	43.2	GGGTGTTTGCTGCAGTTCTT	TCAGTTGGCAACATCCTGAA
576	ICSB0598	Other	4H	45.2	TTACCGTAGGGTGGAGGTTG	CCAGGAAGAGGCCCAAGT
577	ICSB0599	CoreSet	4H	49.4	TGGTGAGCAGTCAGAACCAG	TAACCGCCAGGTCACCTAAG
578	ICSB0341	Other	4H	53.7	CTCCTCAACTTAGCAGCACTTTTA	CACTGGGAGCATTCCTAGTTTAAT
579	ICSB0600	CoreSet	4H	53.7	AGCATTGACATGGGTGCTTA	TGGAAGTCGTAGGAAGGGATT
580	ICSB1041	CoreSet	4H	55.3	ACGTAGAAGCCCCCTCTGAT	ATTGTTTTTCCGTCGCAAGT
581	ICSB0337	Other	4H	56.1	AATACGAAGGGAGTATCTGCAAAC	AGAATATCCAAGAGCAAGAAGCAT
582	ICSB0250	Other	4H	57.1	TCACGGTTCGACTGTACTCTAAAA	AAACAGCAAACTTAGGAACTGGAT
583	ICSB0193	Other	4H	57.3	AATATCCCATGGTGATCGTTCT	ACGATCTGTGGCTTTGGTTAGTAT
584	ICSB0265	Other	4H	57.3	GAGGTTTCACATGGAAGTGATG	CAAATTTCCATTTGAGAAAAGAGG
585	ICSB0601	CoreSet	4H	57.3	CACACCGTGGGAAAAAGAGT	AGCAAATGTTGTTATGCTCTGG
586	ICSB0602	CoreSet	4H	68.3	GCCTGAGGGAATAAATTGAAAA	CAGATGCACAGATCCACAGG
587	ICSB0603	Other	4H	72.3	TCAAACTGGCATGCAAACAT	CGAGATGTTCCCATTATTCCA
588	ICSB1042	CoreSet	4H	73.9	TAATTCCTTCGGCCTCCAC	AGTAAGCAGTTGGCGTTGGA
589	ICSB0604	CoreSet	4H	89.4	TGGTACCTGGCCAACAGAAC	GTTTGCAGGTTAGCGCAGTT
590	ICSB0605	CoreSet	4H	94.6	TTCTAGGACAATGGGCTGGT	TAAAAGGTCTCCGCCCACAC
591	ICSB1043	Other	4H	96.0	TCTCCTCCATTCGTTCCCTA	GTGAGGGACAAGACCGAATC
592	ICSB0606	CoreSet	4H	98.9	TATCTTGAGGAGCGGTGCAA	CCCATCAAAATTAAGTGGGTGT
593	ICSB0607	CoreSet	4H	103.7	TTTCAAGTTAGCTGTGCGGTA	TTTTGCATGATGTGATGGTT
594	ICSB0608	CoreSet	4H	109.7	CACCTGCTTGTTTGTGCTTC	ACGCAACCCATCCAAGTTT
595	ICSB1044	CoreSet	4H	112.4	TCTCTCGGTGTTTAGGTCGT	AACGGCAAATAAAGTGGTTCA
596	ICSB0242	Other	4H	116.8	AAGAGCTCCCAACTTTATTAAACC	CATGTTCAAGCTAGGGAAACATAC
597	ICSB1260	CoreSet	4H	123.8	CAACACAAAAGAGGAGGTGGA	TGTGAGAGACAACGAGGAAGC
598	ICSB0609	CoreSet	4H	124.9	ACACGCACAGTACACACGAA	AGCCCAAGAGGAGGAGGTC
599	ICSB0610	CoreSet	4H	130.1	CAGTAATTCCTGAAGCAGGTAAAAA	CCTGCTTCTCTCAAGTGCCTA
600	ICSB1045	CoreSet	4H	130.8	ACCGGTTGAACTGGAAGCTA	ACACCAGAGGCTTGACTCGT
601	ICSB0611	Other	4H	133.1	CCTAGTAGCCCACGGTAAAGG	TGTTTGACTAAACTTGTATTGTAGAGG
602	ICSB0612	CoreSet	4H	148.7	TTCTAGTTGAGCAAGGGCAAA	CTCGTTACTGCGGGTGCT
603	ICSB1046	CoreSet	4H	150.9	CTGAGACCGACATCGAGACC	TCAATACCGACTTCGCCAGT
604	ICSB0613	Other	4H	154.4	TTCCCGATTCTTCTGTTTCTG	GGCAAAAGTCAGATTAGAGATTCC
605	ICSB0614	Other	4H	158.4	TGTTTCTCGTTTTGTGGAGAA	CAACAGTGGACCATCTGGATT
606	ICSB0615	CoreSet	4H	163.9	ATAGCGCGAATGACAGTGAA	GACACATTCGGGGTCGTC
607	ICSB0616	Other	4H	169.0	TCGGTACCACAAGCATTTCA	GCATTATGAGCTGCCCATGT
608	ICSB1047	CoreSet	4H	170.6	AGGAGCGACCTTCATTATCG	TGTGGTACGCAAATTTTTCTTTT
609	ICSB0617	CoreSet	4H	171.9	CGGAATCGTGTCACAAAGAA	CCCCAGAGACATGGTCCTTA
610	ICSB0618	CoreSet	4H	192.2	GCTTTAGTCGTGACTATGATTCCA	AAGCGGTCAATGACTTCCAG
611	ICSB1048	CoreSet	4H	194.8	TAAAAGGGTTGCTTGGTTGG	ATTGTGGTTGTTCCTGACGA
612	ICSB1049	Other	4H	210.2	GCCCAAGATCAAGTTTGACG	TCCAATGGTCAGGCACAATA
613	ICSB0619	CoreSet	4H	210.9	ATGAAAAAGCACCACAGACG	AGAATAACAGGGCAGGATCG
614	ICSB1261	CoreSet	4H	220.2	AGGGAGCTGCAAAGGTGATA	GGCAAAAGCTTGTGTTATCAGG
615	ICSB0620	CoreSet	4H	222.7	ACTCGTGCTTGGAGGATGTT	TGACCATAGCCGATTCAACTT
616	ICSB1050	Other	4H	230.9	GGCATCTAATGTGCATAATTCAA	TCATGCATGGGACCTAAACA
617	ICSB0621	CoreSet	4H	233.3	TCAACTATGCTGGCACGATAA	TTCACTGGTGCAATGTTGTG
618	ICSB1051	CoreSet	4H	248.2	AAATACACTGGCCGCTGAAC	AGAACCCATGCTCCTTTTCA
619	ICSB0622	Other	4H	256.3	GCGTACAACTCCAGCACGTA	ACTTGCACAGAAGGCTCCAT
620	ICSB0623	CoreSet	4H	260.5	ATGTGCAAGTCAGGATCACC	TCTGCTCTGGAAGTGGGTTT
621	ICSB1052	CoreSet	4H	262.0	TCGTTGATACTTGCATTGTGC	ACCATGCAAATTCCCATCTC
622	ICSB0624	Other	4H	272.6	TGGATACTACAAGTACAATGCCATAA	CGCTACCTTTCTCCCAGGAT
623	ICSB0625	Other	4H	274.5	GCATCATGCAATCAACTTGG	CGGTGAATGTTGAAGGTTTG
624	ICSB0626	CoreSet	4H	279.7	AAACCAGTGTCTCTTCACGACA	AGGTCGTACCCACAGTCACC
625	ICSB1053	Other	4H	286.2	ACTCGGTATGGCCAATTTGT	ATCCACCAAAAGCCTCAAAA
626	ICSB0627	CoreSet	4H	299.4	TGGTCTAACCACCACGTCAG	CCATCACACTCACCCTGTCT
627	ICSB1054	CoreSet	4H	299.5	GTTGCAGAAGTTCCCCAAAA	TTTTGCAAAGGAAAGCAAGC
628	ICSB0628	CoreSet	4H	307.0	ACACTGTAGGGGTCGAATGC	CCACAACTCTTTGTGCTCACC
629	ICSB1055	Other	4H	320.3	TTCTTCATATTCGGCGGTGT	GGCAGGTGTGCTCCTCAT
630	ICSB1262	CoreSet	4H	336.5	CTCCACCTTCGCTGTCACC	TTTTAACCTTGCGGGCATT

Supplemental Table 1. List of amplicon sequencing primer sets for barley.

No.	Name	Type	Chr	Pos[Mb]	F_primer	R_primer
631	ICSB1056	CoreSet	4H	339.7	TGACACATTTCGGACGTTTT	CGAAATGGACGAATTTTGAA
632	ICSB0040	CoreSet	4H	349.6	GAAGCTTCAGGCATACCATTTTC	GAGTAATGAAGCCACAATGAAGAG
633	ICSB0629	CoreSet	4H	352.0	TGGAGGTGCATCTTCAGGTA	TATCACAAGCGCGATCAGAG
634	ICSB1057	CoreSet	4H	366.7	TTGCCTTCCTAAACCACACA	TGGATGCATCATTTGAGCTA
635	ICSB0630	Other	4H	366.8	ATTTCAGGGGCAATCACAAT	AGTGTGGTTGTTTTGGCAAC
636	ICSB0631	CoreSet	4H	383.3	ATATTACTGGGCGTGGAGCA	CGCCATGGATTAAGTCCTGTA
637	ICSB1058	CoreSet	4H	385.8	TGAACTCCTCACTGACACAAAAC	CGTGTGCTTTCGATGTGAAT
638	ICSB0632	CoreSet	4H	401.6	TCCGACCTCTCTCGCTATTC	TCCCACCCACAAATATGTCTC
639	ICSB0633	CoreSet	4H	407.5	CTTGAGGTTAGGTCGGATGC	TCACATCCAGGTCCTGAACA
640	ICSB1059	Other	4H	410.0	GGAGGTTAACTTTGCTTTTGGA	AGTCAGATTTGGCCACACG
641	ICSB0634	CoreSet	4H	412.5	CGTTGGATGGCTAAGCTGAT	GCCGGGGAAGAGTGTCTAGT
642	ICSB0635	CoreSet	4H	414.9	AGCTCCCCGTCATTTAAGGT	TGTCCAAACCTCATGGTTCA
643	ICSB0636	CoreSet	4H	427.2	CATGGCCAATGTTGGTAATTT	TTGGGTCCCACACATAGTCA
644	ICSB0637	Other	4H	428.6	TGCAACTTCGTACCTGCATT	TGCACAACTTCTGATGAAACG
645	ICSB1060	Other	4H	429.9	ATTGCATTGGTTTTCCCTTG	GGACCTTGCTTGTTCCTCCT
646	ICSB0638	Other	4H	437.2	CCATCGTGTGTTGCAACTTT	TGCCACTCCAGTGCAGTAAA
647	ICSB0639	CoreSet	4H	440.3	CCGGAATTGTGTTTGTTTCC	GATTGCAGCGAGTTTGACG
648	ICSB0640	CoreSet	4H	446.4	TCTGCAGTATGGGTGCTTTTC	CACATTGAAGCACGAAGGTT
649	ICSB0641	CoreSet	4H	450.2	TGCCCGTTAGTTTACAGAGG	TTCTCTTCTGGTGTATGCCAAT
650	ICSB1061	CoreSet	4H	451.2	ACGCAGTTGCCTTTTTGTTT	TAATAATTGGGGGCCACCTT
651	ICSB0642	CoreSet	4H	455.3	CACGATTTAGCCCCGTAAAA	GGGAAGCTTCGGGCTAGT
652	ICSB1263	CoreSet	4H	464.1	GCACCAAGCCCTTTCCTT	CGAGGGGCATCATCGTAG
653	ICSB1062	CoreSet	4H	469.6	TTGAAGTTCGCCCTGAGACT	GGATACTCCACTTGATGATGACG
654	ICSB0241	Other	4H	472.3	AGTAAAGGGCAGTTGAAACAACAC	ACAAAAAGAATCACAGGTTAACGG
655	ICSB0643	CoreSet	4H	472.4	GGGGGATCAGAGATTCAGAG	CACGTCGTACGTCTCGTTGT
656	ICSB0644	CoreSet	4H	480.4	TGGGACAAGTACGCAAAGTG	CAGGTTGAGTCGAATGTGGA
657	ICSB0130	Other	4H	482.6	GAGTGTCAAAGAAGAAGAGCATGA	GCCATTTACTTGTTTGAATTCCTC
658	ICSB0131	Other	4H	482.6	ATACTGCAAATGCCATATCTGAAA	ATATGACTCCTCTGCTGGATTCTC
659	ICSB0218	Other	4H	485.4	AGCAGCTAATTCAAATTCAGACAT	CAACTACGATGCCACAAAATTAGA
660	ICSB1264	CoreSet	4H	485.4	AGAAGTGGGATCGTGGGAAG	ACGATCGCATCTCAACCAG
661	ICSB0645	CoreSet	4H	485.9	CTCGCCATTGCTCCCTTG	TCGACCACCGAAAGTTATCC
662	ICSB0234	Other	4H	488.1	CCCAGTATGGTTTATTCTATGCCT	GTCATTAATTTCAAGACACGCCT
663	ICSB0646	Other	4H	490.9	CCCTTTTTCCATGAGGAGTTC	TTTCGCACAACCCCACAAA
664	ICSB1063	Other	4H	491.0	GAACCTAGCCAGGTGGAACC	GGACAGTCTCTAGCCGGATG
665	ICSB0224	Other	4H	493.8	TTTATTTCTACCGCACCTGAAGTT	TTGTTTCCCACTGAGATAAGGATT
666	ICSB0041	CoreSet	4H	494.2	AAATTCCATATGCCCCTACAATC	GACAAGGTGGTGAACATTCCTAAC
667	ICSB0183	Other	4H	494.2	GATTCCTTAATGGACAGTAGCTCA	GAAGAGCAGGCCAAGGTTATAGT
668	ICSB0184	Other	4H	494.2	GTGATGAGAAGGTAGTTCACCAGA	ACTACTGCTCCAACCTGAAAAATC
669	ICSB0270	Other	4H	494.2	AAGAAAAACAACACGTAAACAGCA	ATGTTCATTGGCCTGGTTATCTAT
670	ICSB0647	CoreSet	4H	494.9	AGGACAGAGGGAGGAGGATT	GACACGGAGGCACTGGTATT
671	ICSB0260	Other	4H	496.4	ACTGTACACATGCTAGCTACTGCC	CAAACAAACATATCGAAGCAACAT
672	ICSB0648	CoreSet	4H	501.0	TGAAAAGACGCTGGAAGGAT	TCCAAGCATGGCATTTACTG
673	ICSB0649	CoreSet	4H	504.1	ATTCTGTTGGTGGCTTGGTT	GCTGCTGGCTAGTAGGATTGTT
674	ICSB0042	Other	4H	508.0	ATCTTGTTAGGATAGACCAGCGTC	CACTGCTGTCAACCCTCTGTTC
675	ICSB0110	Other	4H	508.0	ATGCCTCAGTTCCTCTGTCAGT	GAGTTTTTCAAAAGATTTCCCAAC
676	ICSB0111	CoreSet	4H	508.0	ATCTTGTTAGGATAGACCAGCGTC	CACTGCTGTCAACCCTCTGTTC
677	ICSB0650	CoreSet	4H	508.5	GGACATGGTGTTTCCTTGTG	GGTGACGGATTCAGAACTGC
678	ICSB0651	Other	4H	513.6	GGCCAGGTCAAAATGGTCTA	AAATGCGAAGCAGGATTATGA
679	ICSB1064	Other	4H	516.4	AGCTATACGCCCCACAGATT	TGTAATGAACATCGTGCGGTA
680	ICSB0652	CoreSet	4H	520.5	GATGAAGATGGCCGTCACTG	ATGTTTTCACATGCGCACAG
681	ICSB0653	CoreSet	4H	530.2	TTGAGCAATCGCAACATCAT	GGATTTCATGAGGTGCCAAA
682	ICSB0654	CoreSet	4H	536.0	CCAGTCTGCTAGGATTGGAAG	TGGGTGCTCATACAAGCAGT
683	ICSB0655	CoreSet	4H	540.1	TCCATCATCGGTTCTGTTCTT	GCGCCACTAGTAATTTACAGGAA
684	ICSB1065	Other	4H	541.5	AGGAGCGCTGATGGAAGTC	CTTTCGATGCCTTGATACCC
685	ICSB1265	Other	4H	548.7	TGCTAGAGCTGACTTGCAACA	GGAGTGCAAGTGCAACACAC
686	ICSB0043	CoreSet	4H	549.2	TAAAATAGCTGCATGAGAATCCAA	CTGGCTACTAATTCTACGCTCACA
687	ICSB0656	CoreSet	4H	550.1	GTCCTCCTCATCAGGCACTC	TTTTCCACGAGAAGGTTGCT
688	ICSB1066	CoreSet	4H	556.5	CCCTCCCATCTCCATGTTTA	CACGCCAAGACTCATTTGAA
689	ICSB0657	Other	4H	558.7	TGTTCATTGCTCCCATCTCA	TGCATGGTAACACAGTACTGAAAA
690	ICSB0044	CoreSet	4H	559.9	TTCAGAAGTTTTATTCCGAGACCT	GCTGTTATTCCATCTGAATTTGTG
691	ICSB0166	Other	4H	559.9	AAAGAGGTCAGTTCTTTCAGTATGC	TAACGGATGAATTGCTATCTCCTA
692	ICSB0658	CoreSet	4H	560.7	GATCCACCTGGTTCTGGTTG	ACGTTGCCAAAGTCATGAGG
693	ICSB0659	CoreSet	4H	567.2	CAGCAGGTCACCAAGCTCTA	ATGTCATGTCGGCCAATTCT
694	ICSB1067	CoreSet	4H	571.4	ACCTACGGACAAATCCTTCG	GCAATCAATGATGTAAGAGCACA
695	ICSB0660	CoreSet	4H	574.5	GCATCCATGCCGAAAGATAC	CACCGGTTAAGCCAAACCTA
696	ICSB0045	CoreSet	4H	580.6	CTTCATTATGCATGTTGAGGCAG	CTGGATGGTGTCGAAAGAAACT
697	ICSB0171	Other	4H	580.6	GTTCAACGAGGGTCTACGGCTA	CTGGTCCTCCAGGTAGTTCTTCAC
698	ICSB0172	CoreSet	4H	580.6	AGAATGAAATGATGGTCTCCAAGT	TGATCTTACAGATCTCAGGACCAG
699	ICSB0661	CoreSet	4H	590.6	AATTGGAGGCTTCTTCGATGT	CTCTTCGGACCCAACAAGG
700	ICSB0662	CoreSet	4H	594.1	TCATGCTGGAAATGGAAGTG	CTGGATTGTATGCTGGTGCT

Supplemental Table 1. List of amplicon sequencing primer sets for barley.

No.	Name	Type	Chr	Pos[Mb]	F_primer	R_primer
701	ICSB0663	Other	4H	604.1	TGCCTATGTCATCAGCGAAA	GGGATAGCCTGCAACTTTTG
702	ICSB1068	Other	4H	604.7	GGACAGTGGATGAACCAAGG	GGCCTTGAAATCTGAAACCA
703	ICSB1069	CoreSet	4H	605.6	GCTTAAGCAGTGCAAAAGGTC	AAAGAATACGGGGCACCAC
704	ICSB0664	Other	4H	608.1	GCGAACAACAGTTCTGACGA	TGGCCCTTCAAATTTCAGTT
705	ICSB0665	CoreSet	4H	613.6	TCAATGCGTGTCCAGAAAAG	GATGCCATCGACCAAAATTC
706	ICSB0118	Other	4H	620.0	ACAGGAGTATTCTACTGCGGTGC	TAAAAATTCTCCTTGTGGAACTCG
707	ICSB0666	CoreSet	4H	620.8	GCACCAAGGTACATTCTGTCA	TGCAATTTGTAACATTCGCAATA
708	ICSB0667	CoreSet	4H	625.1	CACAGACGCTGGCACATAAA	GGTGTGCGCACTGCATATAA
709	ICSB0668	CoreSet	4H	629.6	CTTCGCACCTACAGCAACAA	TGAAGCCAGCACCATTTTAG
710	ICSB0046	CoreSet	4H	635.0	AAGAAACGCAAAGAAAACTGTCTG	CTGCACTCTTCAATAAGGTTCTCA
711	ICSB0123	Other	4H	635.0	GTAGAGAATCTTGAGAGGGAGCTT	AGACCTCCCGGATAGCATCTACT
712	ICSB0124	Other	4H	635.0	GTTTGAAAAGGGAAAATTAAAGGC	TCTCCAAAGTCTCTTGCATATCAG
713	ICSB0669	Other	4H	635.1	GCCGATCCAACTATATGCAA	CGTACGTACAAAGTGCCCGTAT
714	ICSB1070	CoreSet	4H	641.3	TTGTACAGGAGACAAAGATCTGC	TATAACGCAACAGGGAATGC
715	ICSB1266	CoreSet	4H	642.1	TGATCGCACAAAGTACCTTCC	CACTTGTTTTGATGCAAGCAG
716	ICSB1071	CoreSet	4H	642.2	TTAGGCACCATTCAATCCATC	CATTTTCAAGGTCCCAAATGA
717	ICSB1072	Other	4H	642.2	TCTCAGCTCTCACGGAAGAA	TGGATTGTCAAAGGATGGTTC
718	ICSB0670	CoreSet	4H	643.5	AATCCCGTGCACATCATTTC	TGAATGGATGCGATGTTTTC
719	ICSB1267	CoreSet	4H	645.1	CAGGTAGGGCACCCAGAAG	GATGGCGAGGCCTATCATAA
720	ICSB0671	CoreSet	4H	645.8	CAAACCCTGTTTTGTTTGCAT	TTCAGCTTCCTTGGTCACAA
721	ICSB1268	CoreSet	4H	646.0	TCTCCTCCTTCTGCACGTCT	CTTTCCCTCTCCCCGATTC
722	ICSB0672	CoreSet	5H	0.4	TTGCTGGGAACTGAAATATGC	GGGGAAAGTTAATCAGCCTGT
723	ICSB0047	Other	5H	7.5	TTTGGAATATCAGTGAAAATGGTG	CACTCCTCAAATAGTGATGCATTT
724	ICSB0140	Other	5H	7.5	TGGGCAAGTAATACCTTTTAGCTC	GATCGTCAGCAACATAAATCTCAG
725	ICSB0141	Other	5H	7.5	AAGTCAATGCGACGAGTAAGAAG	TGTTCAACTAGGATTGTCTCAGGT
726	ICSB0142	Other	5H	7.5	CTCTGAAGTACCTGAAGTCCACTG	GTCTGTTGTTGCCCTTTTAACTTC
727	ICSB0143	Other	5H	7.5	CCCTTGATATAGATGCTATGTTTGG	TTACCTGTTACAGAGTCAAAGCGT
728	ICSB0144	Other	5H	7.5	CCCTTGATATAGATGCTATGTTTGG	TTACCTGTTACAGAGTCAAAGCGT
729	ICSB0196	Other	5H	9.3	GGCTCTTTCACCATAAGCAAAG	CACAGCTGAATATATACAACAGCCA
730	ICSB0673	CoreSet	5H	9.3	CCTCCTCGAGCTGCAATTT	TGCAACCTGTCACTGTTCAGA
731	ICSB1269	CoreSet	5H	10.7	AGAAAATTTGTCGCTTTAACCTT	TCACCAGATTTTCTCCCACTC
732	ICSB1270	Other	5H	12.3	CATGCAATTCCACAGACTGC	CCGACCTTATCTACTTTCATCTG
733	ICSB1271	CoreSet	5H	12.5	CCATCCTCGACATCCTTGG	AGCAACTTACTGGGATGCTG
734	ICSB0674	Other	5H	13.6	CACGGTGCTCATATTCGAGA	GTCCCATCTGACCAGACGAT
735	ICSB0675	CoreSet	5H	18.7	TCAGGGTGAGAGCAGCAGTA	CGTCCTACATCTGGGAGAGC
736	ICSB1073	CoreSet	5H	19.3	GCCTGACAACGTCAACACAA	CAACGGTCAACCAATTACAAGA
737	ICSB0676	CoreSet	5H	21.3	AGAAAGGATGGGAGGAGAGG	GCGTGCTAATGGGAATGAAA
738	ICSB1074	CoreSet	5H	22.5	AATTTTGCAAGTGGGACTTTT	TGTGGTCCAGACAAAAAGCA
739	ICSB1272	CoreSet	5H	38.6	CTCCGATAGGTCTGCGTCAT	GAATGGGTCCGCGAAGAT
740	ICSB0677	Other	5H	40.2	AGTTCTGCTGGGGATACCG	AACCCGACCCGAGGTTATAG
741	ICSB1075	Other	5H	40.4	CATGTCTGTGGGACCGGTAT	CATCTGTATGTAGTCCATAATGAAATC
742	ICSB0678	Other	5H	44.6	GCAAACTCACACGATACGACA	TTGGATAGATGCCAAGGTCA
743	ICSB0679	CoreSet	5H	54.4	CCCAAGCTGTTCTCCTCTCC	CGCCCGTCACTATCTACCAG
744	ICSB0680	CoreSet	5H	58.1	CCGGGGAATGTAGTCACACT	CCCGACCTTATTCTTGGACA
745	ICSB1076	CoreSet	5H	61.2	TCCATGGAGAATGGGAAGG	TCGGTACCATGACGACGTT
746	ICSB0048	CoreSet	5H	65.8	AGTTGAACTTGAAGGAGCATCAC	TTTTCGGTCCAGAAAGTCTACTG
747	ICSB0681	CoreSet	5H	65.8	CAAACCTGCAATGAATCAGC	TCCGGTCAAAAACCACTACC
748	ICSB0049	CoreSet	5H	72.3	TCTTTAAATTTGTCCTCCTGGAAC	CCTTCATTGACATGAATAATTTGC
749	ICSB0682	CoreSet	5H	72.9	TGCTGGAATAGCAGCGAAG	CGGATCTCGCTTTCTGTGA
750	ICSB0101	Other	5H	74.3	ATGACCTCACAGAGCTGCCATA	TCAACCTCTTCAGGCTGCATAC
751	ICSB0683	Other	5H	81.6	TTGCGTCTACCGAGCCTAAT	TGCAGGACGAAGAAACAAAA
752	ICSB1077	CoreSet	5H	82.0	CAAACACGGAAATGGACGTT	AGAGCAATTCCAACCACCTG
753	ICSB0684	Other	5H	91.0	AAAACAAGCTGATGCCAAGC	TTCACCAGCAGCACATCAGT
754	ICSB0685	Other	5H	95.2	CCGAGCTAAGAAGCGTTGAG	ACGCGTTGGATTGCTCTTAG
755	ICSB1078	Other	5H	98.2	CATCCTAAATTTTTACACACATACACA	CCATGGAGGCCGAATTTT
756	ICSB1185	CoreSet	5H	100.1	AAATAACCATAACACAGGCCAAA	TGCAATTTATGGCACTGACAC
757	ICSB1186	CoreSet	5H	100.2	ACCTTATCATCGGGGCAGA	TCTTTCTTTGCCGTCTGCTT
758	ICSB0686	Other	5H	103.2	TCGACTGTCAATGCATCACC	GCAAGGGCATGAACTCAAAT
759	ICSB1187	Other	5H	103.3	ATCATCTTGCGGACTCGTG	GGGTGGAGGGTTTTCGTAGT
760	ICSB1079	Other	5H	115.7	AACGTTGCATGGGAAACAA	TTCTTAACGCTTCCCGCTTA
761	ICSB1188	CoreSet	5H	116.2	ATGGAAGATGCTGACGAGGT	GAACCCCACAGCACAAACTT
762	ICSB0687	Other	5H	116.4	CCTCCAGCAAGCATCTACAG	ACTCCGATGCAAAAGAGTCG
763	ICSB0688	Other	5H	122.2	AGGCAAGCATAAAGGCGTAA	CCCGTTCTACATAGGCGTTC
764	ICSB1189	CoreSet	5H	122.2	CTCAACTCCCGAGCATGAAT	CATGGTGGTGAGATGGTTCA
765	ICSB0689	Other	5H	129.7	GGTGTCTTCGAAGTGGTGGA	CCTTTTGATTGCTCCATAGCC
766	ICSB1080	Other	5H	130.8	GAGCGTGAACCATGGACATA	AGCCCATTGATCCACCTAGT
767	ICSB0690	Other	5H	138.1	TGCAGATGGAATGGTCAAGA	TACCACCGCCAGTGATAACC
768	ICSB0691	Other	5H	139.0	CAATGGGTGGCTTTCTAACAA	CGTAACAATCCGATGCAAGA
769	ICSB1190	CoreSet	5H	139.1	ACCTGGACCAATGACACCAT	CATGTTGTCCCAACAATCAAA
770	ICSB1191	Other	5H	139.6	GGCCTGCCTCGGTAAACT	GCCGCAACTGCAAGTTTTT

Supplemental Table 1. List of amplicon sequencing primer sets for barley.

No.	Name	Type	Chr	Pos[Mb]	F_primer	R_primer
771	ICSB1192	CoreSet	5H	139.8	GGTCGAACAACTTGCTGAGA	TTCCTTTTGTTGTGGGGTCT
772	ICSB1193	Other	5H	141.2	CGAACAATGGTCCATCCTCT	TTTTGAAAAGGAGCGCTTGTA
773	ICSB0692	Other	5H	144.3	TGCTCAGGAATCTCTCCACA	ACCTGCTTCTTCCCAATTCA
774	ICSB0693	CoreSet	5H	157.5	AGATCAAAAGGAGCGCTGAG	GTAAGTTGGTGCAGCCAGTG
775	ICSB0050	Other	5H	158.9	GTAAATGTGGGACTTCCTATTTCG	GGGTACAAACTAAAAGAATGGATCA
776	ICSB1081	Other	5H	164.7	GCGTCCACTCGAACTTTTTG	TTAGAGGCTTCCTCGGACTC
777	ICSB0694	CoreSet	5H	173.2	AACCAGCGCACTGTCGTAG	GCTTCAAGTTACGCCCAAAC
778	ICSB1194	Other	5H	179.2	TGCGTCAGCTGAAGACACTT	TGTTCTACGGCGACACTCTT
779	ICSB0695	Other	5H	179.8	CCAAGGTGGGACCATATGAC	GGTCCAACTCCAGAGAATCC
780	ICSB1195	CoreSet	5H	179.9	CCCCTTGTCTCCGTCACTG	AAATTGTGCCTTACCGAAATG
781	ICSB1196	Other	5H	181.5	ACATTACCCAACCGGAGACC	ATAATATACCGGCGGACTGC
782	ICSB1197	CoreSet	5H	182.6	CGCTAAACCTGGGAGTTCTG	AATTCATCAGGGCCATATCC
783	ICSB1082	Other	5H	184.2	TGTCGGTCTCTCCCACCTTA	CCTATGCAAAGCCACAACAG
784	ICSB0696	Other	5H	196.6	CGGATCCGTGGGAGTAGTAA	CAAATTCTAAAACACCCAAGCAC
785	ICSB1198	Other	5H	200.1	CCGATGGAAGGCTAAGAGG	AGTTGTAGTCCCCGAGGATG
786	ICSB1199	CoreSet	5H	200.1	CCCGTACCCCATTAACCTCT	GCGAACCCACCAGTTGTAGT
787	ICSB1200	CoreSet	5H	200.2	CCCTCCTCTAGGAACCACACT	CTCACCAGAGGGAATTTTGC
788	ICSB1083	CoreSet	5H	200.4	ATTGACTCATCCACAGTTTGGT	TACAAGTCGCCTGTTTGAGC
789	ICSB1201	CoreSet	5H	200.5	CTCACCTCATGTCCGAGTCA	TGCAATGAAGCTGAGGACAC
790	ICSB0697	Other	5H	203.3	GCCATTCTGATGATTGAATTCTT	CACCATCTCTGGGGCTAGAA
791	ICSB0698	Other	5H	211.0	GTGCTGGTAATGGAATGCAA	GGCTTCTTGCAGCTCAGTTT
792	ICSB0699	Other	5H	214.9	TGTACTGGCACCAGTGATGG	CGTGGTGTACCACAAATTGAA
793	ICSB1084	Other	5H	215.2	GAGGGGAGCCAGTCTCGT	CTTAGTTCCTGGTCGGATGC
794	ICSB1202	CoreSet	5H	220.3	GGCATGCCTTTTATGAGATG	CCTTCCCAAGTCAGCACCTA
795	ICSB1203	CoreSet	5H	220.6	AGGCATAGACCTCAGACAGGA	CAGAGACGAGGTGGCAATG
796	ICSB1204	CoreSet	5H	220.7	ACGCAACCAAAAGACCAAAC	GTGTAGCTCCGCCACTGATA
797	ICSB1205	Other	5H	220.7	GCATACTGAGCATTGTGAATGA	TGAGGCTGCTAACGAGTTCA
798	ICSB0700	Other	5H	221.9	CGGTCGAACTTGGTTCAAA	GGTCAAGATTGAAACTGTATTTGC
799	ICSB0701	Other	5H	226.6	GGTGTACCAGCCACACTTCA	CCAGCATTTGATGTTGTTTACAT
800	ICSB1085	CoreSet	5H	229.3	TTGCCAAGCGTTAAAAACAA	GTTTTGGGGCAACAAGAGAA
801	ICSB0051	CoreSet	5H	230.2	TTTTCACAAACAAAACCACTTTTT	AATCTCGTGAACAAGTCTGGGT
802	ICSB0702	Other	5H	237.7	TTCAGCAGTGATGCAAAAAG	AAGCCACTGTCTTGTTTTGTGA
803	ICSB1086	Other	5H	239.5	GCAATTCTGGAACAAATTCCTC	GGCTAGCCATTCGTCTCGTA
804	ICSB0703	Other	5H	254.4	GATTCTCAAATGCAGGTCCAA	CATGTAGGGCGCGGATAC
805	ICSB1206	Other	5H	260.6	AGAGAGGTGGCGCAACAA	GCCTGGTCCCAAAGATAGGT
806	ICSB0704	CoreSet	5H	261.5	CTCCAAGGATAGGGCAATCA	CCGTCTCATCAATCATGGTG
807	ICSB1087	CoreSet	5H	261.6	TTCAAATTCCTCAGCCCAAC	AGAATTTCAGCTCCCCCATC
808	ICSB1207	CoreSet	5H	264.7	TTTTAGCAGTAGCACGGTTCA	GGGGACGGGCTACAAATAAT
809	ICSB1208	Other	5H	265.5	TCCAAAGTCACGAATGGACTC	TCACGTCCTCATGGGAAAA
810	ICSB1209	CoreSet	5H	266.1	GAGCGCTAGAGAAGCCAAAA	GCCTTCGTCGAGAGTTGG
811	ICSB0291	Other	5H	272.4	CGCTACTAGAAACTGGGTTAGCAG	AAGAAATACACGCGTTTAGTAGTGG
812	ICSB1088	CoreSet	5H	277.8	GGTCTGGGTACCCCTATTCC	GGACCCGTAGGTTTGTGATG
813	ICSB0290	Other	5H	280.6	TCGCTATTTTAGACATCCTTTGTG	AAACAAGAAGTGAAAGGCTGAAAG
814	ICSB0277	Other	5H	281.3	GTCGGGCAGATAAAACAAGAGTAA	GATTACACGTGCACACTGCTACTA
815	ICSB0279	Other	5H	283.4	GTAGACCAAGGGGACAAATGTAAA	GTAACTAAATACTCCTCCGTTCGC
816	ICSB0280	Other	5H	283.4	CGGAGGAGTATTTAGTTACAGAGG	ATATATGCTCTTTCGGATCGTAAT
817	ICSB0276	Other	5H	284.4	GTATACCTTCGAAGCCTGCCTCT	ATCGCCTAGTACCACTACTTCCAC
818	ICSB0282	Other	5H	287.4	AGTGACAGAACTGATGACAGAACG	AGTGGGACACAACTAGGGGTATAA
819	ICSB0705	CoreSet	5H	288.7	AGGTCCTGTTTGGCACTTTG	TTGTGACCAAAAATGCCTTG
820	ICSB0706	CoreSet	5H	293.3	CGTCCGTTTCTTGAGGGTAA	CCAACAAGCATCCACTAGCC
821	ICSB0287	Other	5H	293.9	GCACCCTAGTTAGGAAGGTTATGA	AATCAACCAAGAACGTTGTACTAGC
822	ICSB0292	Other	5H	295.6	AATAATTTAAGAGCGCAACGAACT	GTTTCATTGGTTTGTGTTTTGTGT
823	ICSB0281	Other	5H	296.7	ATGCTATCGGTACCAAACTCACAT	TGTTTCGAATTTTCCTTGAGATTT
824	ICSB0707	CoreSet	5H	296.7	GAAATCACGTGCACGAATCA	CTCTGCTGAACACGCACAAT
825	ICSB1210	CoreSet	5H	298.3	TGGACCAAAAGAGGAACTGG	AGGCATCAAGACACGCCTAC
826	ICSB1211	CoreSet	5H	298.5	TTTGCATGATCTTTGGATGG	GGGTGTGCAAACTCTTAATGC
827	ICSB1212	CoreSet	5H	299.6	GCTCCGTCATGAAGTGATGT	AATTGATGACCCATGCAACA
828	ICSB0708	CoreSet	5H	299.7	CTGTGCATCGCCATTCCTAT	AAGCTTGACGGCTTATTCCA
829	ICSB1213	Other	5H	299.9	AAACAACAAATTATGCAAGAAAAA	TCCTCATGGTGCCCTAATTT
830	ICSB1214	CoreSet	5H	300.0	TGTATGTCCCTGTATCTTGTGG	TTGCCTCTTCTTCCTCAACTTC
831	ICSB0273	Other	5H	300.5	ACAAATCACGAAAACACACCTATC	ACACTGTGAACAAGGTTTGTCATT
832	ICSB1089	CoreSet	5H	301.4	GTTTCCTTTCTGGCGCAAC	CCCTCTGTCCAAAGATGGTC
833	ICSB0283	Other	5H	305.1	TCTTGAAGGCGTCATTGTGAAG	AAGGGGAAACGACATCAAAGAT
834	ICSB0284	Other	5H	305.1	TCTCAACAATGATGGATGCTAAGT	CCCAGACCACATAGAAAGGAACTA
835	ICSB0285	Other	5H	305.1	TCTCAACAATGATGGATGCTAAGT	CCCAGACCACATAGAAAGGAACTA
836	ICSB0709	CoreSet	5H	305.5	CGTGTGACCCGTTCTTCTTT	AGATGGAAAGCCGTGGAAG
837	ICSB0710	CoreSet	5H	310.0	TGCACTAACGGCAACTACCA	TTTTAGAATTCATGACCCAGTATG
838	ICSB0294	Other	5H	311.1	AGGAGTCCAGCAATGCTAATTTT	AGACTGAATCTGGGTTATACTCCG
839	ICSB0711	CoreSet	5H	313.3	CCTAAACTAGTCAGGAACTAAACCA	CGCTGGCTCCTAGTAGTGTTC
840	ICSB1273	CoreSet	5H	317.7	GCACGGGTACGAGGATACAC	GTCGATGGTGCGTGTTCATA

Supplemental Table 1. List of amplicon sequencing primer sets for barley.

No.	Name	Type	Chr	Pos[Mb]	F_primer	R_primer
841	ICSB0712	CoreSet	5H	318.2	TGAACACCATAGATAGCACCTCA	TGAATGGGCAAGGTTAGAGC
842	ICSB1090	Other	5H	321.4	AGGATAGCCACACATCATTGG	TCCTCTATGCAGCGGTGTAA
843	ICSB0278	Other	5H	321.5	CCTACTACTACCGCTACAGTTCCG	ATGCATGCAGCATTAGCATTAC
844	ICSB0713	Other	5H	331.5	GCTCCTGCATAAGCACCCTA	CTTGCAAGTGCCAACACATT
845	ICSB0052	CoreSet	5H	335.8	ATCCAATATACCTTTCCCCGAC	CTCAAATGGTACCCTTACCTTGTC
846	ICSB0293	Other	5H	336.6	ACCACTTAAACTGTGAAATAGGGC	GGAATAGCAATTGAATCAAGGAAG
847	ICSB0288	Other	5H	341.9	TCACAATCAGGATTCATCTCCTAA	TTCGTCGATGCTTAGTACTACTGG
848	ICSB0274	Other	5H	343.1	AAGTGAGGGAGTAGTATCTGGGC	CAATGATCTACCTCATCAACTTCG
849	ICSB0275	Other	5H	343.1	TTGAGAATAAGGGAGAGAAATTGG	AATTGCGCATGAATGATGAAAC
850	ICSB1091	Other	5H	344.8	CCGCTTAGCGATCTACAAGG	ATATTGGGAACGTTGCATGG
851	ICSB0235	Other	5H	345.1	GCCGTGTTACCTGAAGATAAGTCT	CTGCAATCACAAAATGTTTTCAAC
852	ICSB0286	Other	5H	345.1	TTGTTGAAAACATTTTGTGATTGC	AATCGATATATTTCCTGGAACTGC
853	ICSB0714	Other	5H	347.8	GCGGTGGACGGCTTATACT	GAGCGGAAGAACTTGGACTG
854	ICSB0245	Other	5H	348.3	CGCCAATCACTAGAAGACGTAACT	CTCCAGTCGAATGTAGTGGAAACT
855	ICSB1274	CoreSet	5H	348.6	GCGACACTCCGAAAGAAAGA	TGGCAAGGTATACGGACACA
856	ICSB0053	Other	5H	349.7	GGATGCTGAGGAGTTAATTGATTT	GACTGACGTACAATATACGCATCAA
857	ICSB0289	Other	5H	354.5	GCCATTAGTCCTTGGTATGTCTGT	TTGTAACACCAGAAGTGGAATCTC
858	ICSB0715	Other	5H	354.5	TCAGGTGACTGGTGACTTGTTT	CGCTATCCTAATGGCTCCAA
859	ICSB1092	CoreSet	5H	359.7	TCATTAAAGTGTGGCAGGTCA	TTTTGCCACATTTGCTATGG
860	ICSB0295	Other	5H	362.6	GACAAGGAGAGAAGCTGATTCG	CCAAGTTTGTCTCCTCTCGCTA
861	ICSB0716	CoreSet	5H	364.9	TCGTCCAGATCCAGTTGACA	CTGTCCGGCATACAGTGAGA
862	ICSB1093	CoreSet	5H	370.1	AGATGGGAACAAGTCGTCGT	CAGGCACTAAGACTAACCCACA
863	ICSB1094	Other	5H	370.2	GACAGCAACAAGTGCCTGAA	TGGTCTGAAGATGTGCTTCG
864	ICSB0717	Other	5H	373.9	TCATGTGAGCCAAGGAAGTG	AATGGTGCTAGGCTGATTGC
865	ICSB0718	CoreSet	5H	384.7	TGAGGTATGACGTTGGACGA	GGGTCTCTCGAGCAAACAAA
866	ICSB0262	Other	5H	385.1	TCGTGATATCCACTGCGTAATACT	CAAAGAAGCTAATCTATTCCCTCG
867	ICSB0719	Other	5H	391.5	TCTTGCGTTCTGATTGATCC	CCAGGTATCCGTGCAACTTT
868	ICSB1095	CoreSet	5H	393.6	CGGAACCCAAATACCACTTC	TGTTGGTGACCCAATGTTGT
869	ICSB0054	Other	5H	399.7	TAAATGGAACTGCTTCAACAGAAA	TCTTTCTTCCTTCCATACAATGAC
870	ICSB0720	CoreSet	5H	400.6	CAACAGTCCTAACTTCCCACTC	TTTGTGTTGTTGTGTGCATGT
871	ICSB0721	CoreSet	5H	405.8	TCTGCAGACAATATCCCAAGC	CACAAGTTGTAACAGGGGTGA
872	ICSB0722	CoreSet	5H	410.1	TTTGGACGATTCATTCCTTTG	TGACTCACAATTGCCAAACC
873	ICSB0055	Other	5H	417.5	AATCTCAATTAGCAAAAGGAGGAA	GAAATGATGTTCCTTTTGTCACC
874	ICSB1275	CoreSet	5H	420.4	AATCGGGCGATACTTGACAG	CGACATGAACTGGGTAAGCA
875	ICSB0723	CoreSet	5H	424.4	TGCAACCATTGCTAGACGAC	ACTACCCAGGTGGAGCACTG
876	ICSB0724	Other	5H	426.6	TGGAGTGCTTTGACAACAAAA	CGTGTCTGGAGCATTGACC
877	ICSB0725	CoreSet	5H	433.6	TAGTTGACGGACTCCCCAAA	GCACAAGAAAGTGCTTGCAG
878	ICSB0726	CoreSet	5H	437.7	CCACCTGCTGATTTTCCATT	GCAGTAACTTGTCTTCCCTCAA
879	ICSB0727	CoreSet	5H	440.4	GGGGTGCACACAACTCATC	ACTGAAGCCGCAAAGAATGT
880	ICSB1096	CoreSet	5H	452.5	GCTCGGCTAGTCTCTAGCAA	ACCCCAGAGGAGATCACGTT
881	ICSB0728	Other	5H	456.8	CTTCGTCGGTTCCAATGTTT	GACATTGAATCGGGGAGAAA
882	ICSB0729	CoreSet	5H	459.6	AGCAACTCCTGCAAGTTGTG	ACGGTGTGTTCCCTACAGGA
883	ICSB0056	Other	5H	467.3	CACCTGGTCAAAATACATGACTTC	CTGGTTCCATAAGTAGTTTGGTGG
884	ICSB0167	Other	5H	467.3	GCCTGCATACTTTGACAAATACAA	CACAAAGATCACTCAAGCAAACTC
885	ICSB0168	CoreSet	5H	467.3	ATACCTCCTTTGGTTGCACATATT	GTGCATGTAGTGATCCTAACACAA
886	ICSB0730	CoreSet	5H	468.3	CGGTTGGTTTGGGTTTGTAA	GCAGCTTGTCTGGGTTCTTC
887	ICSB0731	CoreSet	5H	472.2	CAGCTCGTGGTTCGACACT	TTTGCATTGTTTGGTACATGC
888	ICSB1097	CoreSet	5H	474.1	CAACAAGGGCTTAGCTCGTC	ACACATGCCATCCAAATGAA
889	ICSB0732	CoreSet	5H	477.4	CGTCAAACTCGTTCATGTGG	TATGCAGCTTGAGGGAATGG
890	ICSB0733	CoreSet	5H	481.6	ACTAGGAGGAGGTCCGAGGT	TCCTTCTCCGTGAGCTCTGT
891	ICSB1276	CoreSet	5H	484.9	ACCCAGCAAAAATGTTGGAG	TTTTGCCGTTCTACGGTGAC
892	ICSB0734	CoreSet	5H	485.9	TAAAAGCTGGCGGTGGTTT	GCATGCCACGATCCACATA
893	ICSB0735	Other	5H	492.2	GGGTCCATTGACGGTAAGC	CATGCCCTTATTGAGCTACCA
894	ICSB0736	CoreSet	5H	495.3	GGAACAGGTTTGGCAAAAGA	TACTGTCCATCAGCCGAGGT
895	ICSB1098	CoreSet	5H	500.7	TGACAATGCAAACATTGTAAACC	CCAAATAATGCCACCACACA
896	ICSB0737	CoreSet	5H	513.0	TTCCGGAATACGGTATGACA	TGATAAGGCCACAAGGGAAT
897	ICSB0057	CoreSet	5H	516.5	GTCCATCTACTCCCTGACGTTC	ATGTTCATGGAGCTGAAATCCT
898	ICSB1099	Other	5H	520.4	CTTTTTCGGAGAGGGAGGAG	AGCTCCAGCGCTCTAGTCC
899	ICSB0738	Other	5H	526.7	TCCACACACACCTACAACAACA	AGAGCTGTGAATTGGGAGGT
900	ICSB1277	CoreSet	5H	529.4	CAGCAACAACAAGCAGAAGA	GGGCCAAATTGACCATGAAT
901	ICSB0739	Other	5H	529.6	GAAGGTGCTTGAAGGCAAAG	CCGGTCGAGAACTTGGTAAG
902	ICSB0740	CoreSet	5H	534.8	TGAGTGTGGATTTCCCTTCG	GCTGTAAATCAAGGGCAGGA
903	ICSB0741	Other	5H	542.6	GGGACTTGCATTCAGGAAAA	AGCTTGTCCCATGAATTGTG
904	ICSB1100	Other	5H	544.2	GCCTGCTGATTGCACCTTAT	TCACGGAAACGTGAGGTACT
905	ICSB0742	CoreSet	5H	544.6	ATCAGTAGCCAATGCCTGGT	GAAGGCAAAGCAAAAATCCA
906	ICSB1101	CoreSet	5H	544.8	CATTGAGTTTCATCCGTTGC	GGCTGTGCTTGAAAAGTTTG
907	ICSB1102	CoreSet	5H	546.0	AAGAATGGGAAAAGCCGAAT	AACTGACGCTCCAAGAAGCA
908	ICSB0058	CoreSet	5H	547.1	CCAGATAGTACATTTCACCATTGC	ATTACATCCCTGTTTGAGAAGGAA
909	ICSB0743	CoreSet	5H	551.8	CCCTCTGGAGTCAGAAGTCG	TGGCTTTGCTTCTAGGCTTC
910	ICSB0744	Other	5H	553.3	ATGTTGTTCCAGCCATCGTC	GCAACCACTGTGGAGCACTA

Supplemental Table 1. List of amplicon sequencing primer sets for barley.

No.	Name	Type	Chr	Pos[Mb]	F_primer	R_primer
911	ICSB1103	CoreSet	5H	553.4	CATTCATTTTAGTCCCTTAATGAAATC	ATGCCAAGCTGCTCCTCTC
912	ICSB1104	CoreSet	5H	553.6	ATCCAGGATACATCGCCTCA	CTCATTCGGAAGCCCTAGC
913	ICSB0745	CoreSet	5H	556.4	TGAAATTCTTGTCATGCCACT	GGCGCAATCAGGAACTAAAA
914	ICSB0746	Other	5H	563.3	CCAGACTCACATGTGTATTGTTGTA	GTGGTACATGGCGACCAAAT
915	ICSB0747	CoreSet	5H	567.4	GAGGGCCTCGACATCATCTA	CCGTTCTTGAGCTCCTTGAC
916	ICSB1278	Other	5H	568.4	TAAGAAGGTCGGTGCCAAAC	TGTAGTCTCATGCGGGGAAG
917	ICSB0748	CoreSet	5H	571.0	CTCCACGGGGAAGCTGTT	TCCCGGAAACACAAGACAAT
918	ICSB1105	CoreSet	5H	575.6	TTTTTGATAGACCTCAGTGTGC	ATTGCAATGGCAGCGATAAT
919	ICSB0749	Other	5H	577.2	ACTGCTCGTTTCTCCAGCTC	CAACTGTGATCTAAAACATGTGTCC
920	ICSB0750	CoreSet	5H	580.5	TTGACATTCGCAAGGAGCTA	TTCACTAAGCCTGTGCATGG
921	ICSB1106	Other	5H	585.4	GTCATGGCGTACGTGGACT	CACGTGCTACTCTCGCTGTC
922	ICSB0059	CoreSet	5H	585.7	CTATTGCTGATCATGCTCTCTGTC	TGATGTTATCTATGCAGAAGCAGC
923	ICSB0152	Other	5H	585.7	TCAAGGAGAAAACCATGTCAACTA	GAGAAGGAAGTCCTATCTGTTCCA
924	ICSB0751	Other	5H	585.9	CTCCCGTGGTAAGTCTTAAAAA	CCTGGAAGTGATGCGAAGAG
925	ICSB0752	CoreSet	5H	589.8	TGACGGTTGCATTAATTTGG	ATTTTAGCTGGCTTGGCTTG
926	ICSB0753	Other	5H	594.4	CAAGCTGTTCCGTGACTTGA	TTGTCCTTGGATCTGGATGG
927	ICSB0754	Other	5H	598.3	TGCAATTTCACCCACCAATA	TTCAGAAAATTCACAGGTTGTCA
928	ICSB0263	Other	5H	602.2	CTTTATTTCCTCATTTGCATGTTG	GAAACAACCTTCGACGAACAGTAT
929	ICSB0225	Other	5H	602.9	CCATCCGTAGGCTGTAGTAGAAAT	CCAAGTTTAGGATCCAAGTGGTA
930	ICSB0188	Other	5H	603.0	GGTGGTAAAGTTACATCAGACCAG	ATTTTGCCTTAGGTTGGTCTTTCT
931	ICSB0755	CoreSet	5H	603.5	CTTCACTACAACGCCGAGAA	ATCCGATAGGATCTGCGACA
932	ICSB0219	Other	5H	609.2	ATTGGTGGAAGGTTTAATGATGTT	TTGGATGGTGCTGTGTATTTACTT
933	ICSB0220	Other	5H	609.2	GAGTTGACACAGATTTTGCTTTTG	TATGTCTCCCACAGAGAGAAGTGA
934	ICSB0206	CoreSet	5H	609.5	CATTTCGAAACAAGATGGACATAG	AACTCGATTAGGTGGCCTGTTAT
935	ICSB0204	Other	5H	612.9	GTGCAGTTTCTGTTAGCTGATTTG	TGTCCACATGGTTTTATAGAATGG
936	ICSB0254	CoreSet	5H	613.0	AGTCATACGAGGAGCTATAAGGGA	AATTCTTTTGCTTACCCTCCATTC
937	ICSB0255	Other	5H	613.0	ACACCAGATACAACATGGCACA	ACAGTTATGCAACACAGCAGCTT
938	ICSB1107	CoreSet	5H	616.1	CCTCACAGGAGTTCGTCCAT	CCGAGATTTGCTTCAACCAT
939	ICSB0756	Other	5H	616.4	ACGCCGAGAAACACAAAAAG	TGGCTATAAGACCGATAAACCAA
940	ICSB0174	CoreSet	5H	616.6	GTCATCCTCATCATCGTCATCC	AGGATCATCAGAGACAGACAACAG
941	ICSB0173	Other	5H	616.7	CATCGAGGAGAATCAAGAAACC	CTCGAGAAGAGGTTGTTCATGG
942	ICSB0757	CoreSet	5H	620.2	GTACCGTGTCAAAGGCCAAG	TGTGCACATGGAACACAAAA
943	ICSB0237	Other	5H	623.9	CTTGGCTTCTTCCACCTACTTTT	CTTTGATTGGTATATTCATGGCAC
944	ICSB0216	Other	5H	626.1	CAAGCACACCGTTATAACAAGTAAA	TTTTTAAAATGCATCACCAATCAC
945	ICSB0758	CoreSet	5H	636.4	TGCCTGGTAGGCCTTTAGTG	CCGTGCATGACCATATATCAG
946	ICSB1108	Other	5H	638.2	GGGGGAAGAGGGGAAGAA	GTCGATGGCGTCCTCTTCA
947	ICSB1109	Other	5H	638.2	TGATGTTCATATTTGGGTTGG	GGGGAAGCTTGTTCTTGTCC
948	ICSB0060	CoreSet	5H	638.5	CTGTTCGTTCTGAAGCTGGATT	CAGATCCCCGTAGGTGTAGTTG
949	ICSB1110	CoreSet	5H	642.9	TGGGAAGGGAGTGAGAACAT	TCTGGCGAGTGTAGACCTCA
950	ICSB0759	Other	5H	643.0	TTCTATTAAGGCCAGTGGTTCA	TGACGCACACTATCGTTTCA
951	ICSB0102	Other	5H	644.5	TGCGTGCTTAATTAACAGAGATTC	CACTGTAGGACGTACGCGTTTA
952	ICSB1111	CoreSet	5H	647.6	TGTCCTCAAACCAAGCACAC	CCCATTGATCCCCGAAAT
953	ICSB0760	CoreSet	5H	648.5	CATTGTGCTGCAGACTGGTT	CGGTTTGGATATAAGCACCA
954	ICSB1112	Other	5H	650.9	TCCGTCCCAAAATTCTTGTC	TCCATCCCAAAATTCTTGTCTT
955	ICSB1279	Other	5H	653.9	CCACTGAACTTGCGTTTGAA	AAGTCGTTATGTATAGCTGGCTTC
956	ICSB0212	Other	5H	657.3	TATTCTGACCAAATGTCTTGATGC	AAATGCCCTGAGTTTGACCTTTTA
957	ICSB0761	CoreSet	5H	661.5	CGCTGTCACTGGTAACGTTGT	GTTTGTTAAGACGGCCGAAG
958	ICSB1280	Other	5H	662.2	ACCGTTGAAGAACTGGCTGT	GTGTGCCCTTCGAGATGC
959	ICSB1281	Other	5H	664.6	CGTTTGCTCACCTTGTGTTC	CTCCATGGGTGATGGAAGTT
960	ICSB1282	CoreSet	5H	665.6	GAAGGGCTCTGGAAACTTCA	CGAAACATCCGTCACCAGAT
961	ICSB1283	CoreSet	5H	666.0	TCGTTCTGGCATTACCACAG	GCTCGAGTGGACCAGGAG
962	ICSB1284	CoreSet	5H	667.6	TGAAGTTGCCCTTAGTTTCCA	GGCTCTGCCACCTCGATATT
963	ICSB0762	Other	5H	668.6	CATCCCAACTGTTTCATCCA	GCATCTGGACTCCACACAAG
964	ICSB0061	CoreSet	5H	669.6	GACCTTCCATTCTGAACGAATC	ACCGGACTGAGACTGAGGTATTAG
965	ICSB1113	Other	6H	0.3	GGGAACAATTTTAGAAATTCATGG	TCGGTTATTAGGCCCTTTGC
966	ICSB1114	CoreSet	6H	0.3	TGGAAGGAATGAAATGTCCA	TTCCATTGGAGGGATCAAAC
967	ICSB1115	CoreSet	6H	0.5	GAGTAGAGAGGAGGCTGAACGA	AAGGCCTCTTTTGATTCTCTACA
968	ICSB0062	CoreSet	6H	1.6	CTAGATGCTCCTCTACTCAAAGGC	AGATCCGCAAGACATTCAACAT
969	ICSB0097	Other	6H	1.6	ACTAAGAGTTGGTCATTCAGCACA	AATGACTTTACACCTGAGGAGGAG
970	ICSB1116	CoreSet	6H	6.2	TACGAGCTCATGCCTAATGG	GCCGATAGCTATGCTTGCTC
971	ICSB1285	Other	6H	6.2	CATACCGCCCAGTTTAGGAA	TGGTGATTGTGGAGAGAGGA
972	ICSB1286	Other	6H	6.2	CAATTAAATTTCACGCTGCAC	TTCCAAAACTGTAAACATCTCCA
973	ICSB1117	CoreSet	6H	6.3	CCTTCCAATGACCAGCTCAG	GCTGCTGCTGCTGAAACAT
974	ICSB1118	CoreSet	6H	6.5	AAAGAGCAAAAATAATGCTAGACC	CACATGGTCATGGAGATCGT
975	ICSB1119	CoreSet	6H	6.5	CGGTTTATCACGTCAGATCCT	TCAAGCAGGAAGCAGTAGCA
976	ICSB1120	CoreSet	6H	7.2	GCGATATACTCGTCGGCAAC	ATGCATCATGCCATTCAGG
977	ICSB1121	CoreSet	6H	7.9	GGGGAGTTCTACTGGAACAGTG	GACGGCATAGGACATGGAAG
978	ICSB1287	CoreSet	6H	8.8	CATGGCAACAAAACTTTGGA	TGGTGATGTACGCATTGTCA
979	ICSB1288	CoreSet	6H	11.5	TGGACCACCTAGAATCGTCA	TTTTTGTGAGGGCAGCTTAT
980	ICSB0763	CoreSet	6H	13.5	AAATGCAAGCCTCAATTGCT	TGAACCAAGATGATCTGCACTC

Supplemental Table 1. List of amplicon sequencing primer sets for barley.

No.	Name	Type	Chr	Pos[Mb]	F_primer	R_primer
981	ICSB1289	CoreSet	6H	13.5	CTGCAGGCCTCTGTATGTGA	CGACCTAGGAAGCCTCATTC
982	ICSB0063	CoreSet	6H	14.5	AACTTCTAGAAATGAAGCAGCCAT	ATTATGCTGGTGGTCTCTGGTC
983	ICSB1122	CoreSet	6H	14.8	AAGCAGCCCACATAGCGTAG	TGGCATGGTAACTTTGACCTC
984	ICSB1123	CoreSet	6H	16.4	TTGGATCACTGCACTTCAGC	CCCCCATGACAATCTAATCA
985	ICSB1124	CoreSet	6H	16.5	GAAGCACACAGGTTGGATCA	TCGTATGACACCCCTACACG
986	ICSB0764	CoreSet	6H	18.9	TTTTTGGGGGCTCATGATAG	TTGAGTGAAGCAAAAACATGAA
987	ICSB0765	CoreSet	6H	22.3	ACACCAGGCACAGACATGAA	TGTTCAAAACTGTGTATCCATTCC
988	ICSB0766	CoreSet	6H	30.3	AACGGCTGTAGCTTGCTCAT	GTTCCTGAGCCTGGTTCTTG
989	ICSB0767	CoreSet	6H	36.6	AGAAGCGACGACTGCAGATT	TCTTGCCTTGTGCAAACAAC
990	ICSB1125	CoreSet	6H	36.9	ATGCTGGCTCAGTCTTTTGG	TTCATCCTTATGAATGTACACACG
991	ICSB0247	Other	6H	39.7	AATATCACTACCTAACCACCGGG	CAGATTTATCACAAGCTTCTCCAG
992	ICSB0768	CoreSet	6H	40.5	TGTAGCCTTGCATGTGGTGT	GTCTGCACAATCGCCATATT
993	ICSB0769	CoreSet	6H	44.3	ATATGCCTCCGGAAATGTTG	GGTGTCCAGAAACAGGAAGC
994	ICSB0770	CoreSet	6H	48.7	GCTGTTGGTGGATTGAGAGC	TCACGCCTTGTTTCCTTCTC
995	ICSB1126	Other	6H	50.2	TGGCTCGGGTTGTTGATTAT	GATGTACTGTGTGAGCCAGCA
996	ICSB0268	Other	6H	59.1	TGTCTAAGATTTCAAGCAGAACAAA	AGAGCCTTGCTAGGAGAAATAGTG
997	ICSB0252	Other	6H	60.8	AGAACCAAGGGGTTCTCATCAC	TATCACCTTTGGGGAATTTAAAAC
998	ICSB0064	Other	6H	61.1	AGATCCAACCAGATTCTGAGTACC	AACGCTTCAGGTCTTCATAAGG
999	ICSB0771	CoreSet	6H	63.1	GAGCATGGAAATAGACGTGGA	TCGTGTAGCATTCGATTTGG
1000	ICSB0214	Other	6H	67.7	GAGGATGAGGAATTCGAGTGAG	TGTTTTTGTGCAAGGAAGAAATAA
1001	ICSB1127	CoreSet	6H	68.2	CGCACAATGATGAGAGGAAG	TGAGCTCCTCGGTGAAGTTT
1002	ICSB0772	CoreSet	6H	72.7	TGACGAGGAATTTGATGTCG	TTGACCCAAATTTAAAAGCTCAA
1003	ICSB0773	CoreSet	6H	74.5	GCTCCCGTTTTTGGTTATGA	TGGCAATTACATTCGGTGTG
1004	ICSB1290	CoreSet	6H	88.1	TGTGCAGGTTGTTTGGTTTG	AGAATGCAGGGGATACGAGA
1005	ICSB1128	Other	6H	90.3	GTCTTAGCGCCCTAACTCCA	CGCGATGTCATTATGGAACA
1006	ICSB0774	CoreSet	6H	91.4	CTATAGAGGGGCTATGGGATGA	CGGCATCATCTTATCGCTGT
1007	ICSB0775	CoreSet	6H	95.1	AACATACATGCCTCCTTGACG	TTTGGCAGCTTAAAAATTACCAG
1008	ICSB0065	CoreSet	6H	95.4	CTGTACACTAGTTGCTGCATGCTC	CAAACTTCACATACACATCGGTAA
1009	ICSB0776	CoreSet	6H	97.8	GGTGCCCTGTCTGTTCAAT	CCAGCAACAGTTTAAACCACA
1010	ICSB0777	CoreSet	6H	102.5	CCATGCCTGTTTGGACAATA	TGGCTATCTTTGCATTCCTG
1011	ICSB1129	CoreSet	6H	110.9	TCCTTCCAGTTCTCCTTTTTG	TGGATCCACCACCTATGACA
1012	ICSB0778	CoreSet	6H	114.4	TGTCTGTGTCTCCCCATTTG	TGAATAATGTCAAGGCAGTGG
1013	ICSB0194	Other	6H	115.8	TGGCCTATTTTTAGAATTTGTTACC	CATGATATGATAAAGCAGGCAAAA
1014	ICSB0066	CoreSet	6H	117.9	GGTTAAATCACATTGTTTGGCG	AAATTATGAATTTGGGAGGCGT
1015	ICSB0779	CoreSet	6H	121.8	ATCGTCTGCCTTCCGTTCT	GGAGGTAAGATTTCTGCTCCA
1016	ICSB0211	Other	6H	125.2	GATCTCTTTTCTTCTCGGTTCTTG	GAGAAATAGAGAAATCGGTGTCAAA
1017	ICSB0780	CoreSet	6H	134.1	CATGACCGCAAAATTGCTTA	AGCAGGAAGAAACTGCCAAA
1018	ICSB1291	CoreSet	6H	134.1	TAAATCAAGCATCGCCCATT	TGTGTCACTTGTTTTGGGACA
1019	ICSB1130	Other	6H	135.1	GATAGACAAGGCCGCTTCTT	GCGCTATCGTACGTCCATGT
1020	ICSB0781	CoreSet	6H	139.5	GAGCCTCTGCTCGTGGTG	CGCTAATTGGAATCCACCTG
1021	ICSB0782	CoreSet	6H	144.7	GCCCGTGAGTAGTCTTTGCT	GTTCGGGAACACGGACAC
1022	ICSB0191	Other	6H	148.6	TAGCTATTTCCTAGGGTTTTTGGA	ATTTTCAAGAAAAGAACCTAGCCC
1023	ICSB0067	CoreSet	6H	149.9	CTTCATCTCCTTCTCGCTCTTG	GAAGCAGTACTTCCTGGGTCTC
1024	ICSB1131	Other	6H	153.2	GCGGCAAATAAGCTTGTGG	TCCCCGTCAAAACTAACTCG
1025	ICSB0783	CoreSet	6H	157.6	TTGGAATCCTGTGACGAACA	TGTAATCAGACGACATTAGTACAACA
1026	ICSB0068	CoreSet	6H	165.7	GACTTGACTTTCTTCTCCAAGAGG	AACGGTTCGACGAGTACACAAC
1027	ICSB0105	Other	6H	165.7	ATAGGATGTAATCTTGGCCTGGT	GCTAGGCATTATCCCTGGTATTTT
1028	ICSB0106	Other	6H	165.7	GATGGTTGTCGACAGATTGAAA	AGGAAAAGAGGGATGAGACCAC
1029	ICSB0199	Other	6H	165.7	TAAAGAGAACACAAGATGATGGGA	CCTCTTGGAGAAGAAAGTCAAGTC
1030	ICSB0784	CoreSet	6H	165.7	CGATGGTTGTCGACAGATTG	GATGAGACCACGCCACTTCT
1031	ICSB1292	CoreSet	6H	166.2	CTGGCAAGCCTTTTCTGATT	CACCACTCAGGGTGTGTGC
1032	ICSB0785	CoreSet	6H	172.5	GCATGTAGTGAACGCTGCAA	ACTGCAGAGCGAAGGAATTT
1033	ICSB0198	Other	6H	172.6	TACTGCTTTTGTAGTTTGCTTTGC	ATGTCATCAGTAACAGCGTACATT
1034	ICSB0271	Other	6H	173.4	GAAAATCTGGATCTGGGCCTAC	CATCTTCAGCCACTTCTCGTAAC
1035	ICSB0786	CoreSet	6H	173.4	CGCGATACAGGTGAACAAAC	GAGCTGCAGAGATGGGTTCT
1036	ICSB1132	Other	6H	174.3	TTTGGATTCCATAAATTGTTCTC	TTGTTGACCGGTGGGTTATT
1037	ICSB0787	CoreSet	6H	179.1	GCAACACTCAGACAGATGCAA	TGCTGATGCTGCCTACTACC
1038	ICSB0788	CoreSet	6H	184.9	GTGGCTGTGTTGTGAACCTG	GGATCCATCGATCAACCTTC
1039	ICSB0789	CoreSet	6H	187.5	CGTGTCAACTGTTTGGCATAA	TTCCAAAGGTACAGTTCATCCA
1040	ICSB1133	Other	6H	197.3	CGCTCCAAGACTTTTAGTACCG	CCCATGGTAGCGCAAAGATA
1041	ICSB0069	CoreSet	6H	197.7	ATGTGGAGAAAACCATTCGATATT	GCTTGTAAGTTTCATGGAGTGTTG
1042	ICSB0122	Other	6H	197.7	AATATAAACTCCCGAGACAAATGG	ATCGAATGGTTTTCTCCACATTAT
1043	ICSB0790	CoreSet	6H	197.7	GGACCTGGATCAGGGTACAA	GCAGAGGATGAATAGCAGCA
1044	ICSB0791	CoreSet	6H	207.8	GAACAAGACCGCAGGATAGG	ACGGTGGAGAGACTGAGCAA
1045	ICSB0792	Other	6H	210.0	TGATTCTCGTCATGCAGCTC	TCCATGCCAGATCAAGTGAC
1046	ICSB0205	Other	6H	210.5	ATAAGTGTTCACCATGTTGTGCTT	AGAACAACTTGTACAGGACATGGA
1047	ICSB1134	CoreSet	6H	213.3	CATAGGAGCTGTCGGCAAAG	GCAGATCCAAGGAATCCTCA
1048	ICSB0793	CoreSet	6H	223.1	CAATGAGTTATCTGGGTGCACTT	TTTCCTCATTAATCACAAAACCTA
1049	ICSB0794	Other	6H	224.6	TTGCCGAACATCGATATGAA	TGCAGCTAAACTTCAGTGAAAA
1050	ICSB0795	CoreSet	6H	229.6	GGAGCTGGTGAAGGTACGAC	CGGATCCGGACTATCAACTT

Supplemental Table 1. List of amplicon sequencing primer sets for barley.

No.	Name	Type	Chr	Pos[Mb]	F_primer	R_primer
1051	ICSB0796	Other	6H	231.5	AGGACAGCGGCAAATAAAAA	TCCATCAACCTTTTGGCTTC
1052	ICSB1135	Other	6H	233.1	AACAGCCAAAGAAGCGATAAA	CTGACCGCCACAACGACT
1053	ICSB0070	CoreSet	6H	242.3	GATATTTGATGGAGAAAAATTGCC	TAAATAAATGCAATACCCCTTTTCA
1054	ICSB0797	CoreSet	6H	247.6	GGCATGCAAGAACAAAAACA	TACGAAGTACGGGGCATTTC
1055	ICSB1136	Other	6H	253.0	TGTGTGCTTTACAAATCTTTATGTCA	AATTATGAGTAGTAAAACCAGATTCGT
1056	ICSB0798	Other	6H	257.2	GTTTCCGCAACATCACCTTT	CCGTGGTGGCGTAATAATTT
1057	ICSB0799	CoreSet	6H	262.2	GCAAGACAATGATCTCAAACG	GCTTACTTCCATTGGGGATG
1058	ICSB0800	CoreSet	6H	273.6	AGCGGTCAGATGTTGATGGT	TCTCTTCACCGTCTGCAGTG
1059	ICSB1137	CoreSet	6H	274.1	GCCTTTTTATTCATTTTTCCCATA	TGGAGCAATTTGATTTCTAAAAGA
1060	ICSB0801	CoreSet	6H	276.3	AGAGCTCTGCCCACTCTCAA	AGGGTTTTTGAAGCTGGTTG
1061	ICSB0802	CoreSet	6H	280.3	CTCAGTACTGCCCGGTGAAT	AAATATTGGAATCAAGTGAAGGGTA
1062	ICSB0803	Other	6H	288.9	TAATCGTCCTCGGTTTCTGC	TCAGCGAGATAATACGGCAAT
1063	ICSB0804	CoreSet	6H	292.0	CTTTAGGCCAAGCAACATGA	TGCATGTTACAGACCGAGACA
1064	ICSB1138	CoreSet	6H	294.0	TCCTTTCCCAAGTGCTTACC	ATGCATGGTGTTGGAAACAA
1065	ICSB0805	Other	6H	298.9	CAATTATGATAGTATGCACAAATTCAA	AAAGGGCCAAAATTTCCAAT
1066	ICSB1139	CoreSet	6H	311.5	GATTCTTTGCCGTCTGTGGT	ACCTGTTGGGATGGAACCTA
1067	ICSB0202	Other	6H	322.1	TAGTTCAGATTGCAAATTCGTACC	AGATCAACGAGGTTGCATACAAGT
1068	ICSB1140	Other	6H	332.0	CCATGGCACACGACAAAG	CAAAGAACCTCTTCACCGAAA
1069	ICSB0806	CoreSet	6H	341.7	CCAGAAGCTATCCCGAGTCA	TCCTCTAGGAGGCTAGCTGAAA
1070	ICSB0071	CoreSet	6H	342.3	CTCTACCATACCAACCGGGAAC	CTAAAACTTTGCAAGGGTTGGT
1071	ICSB0807	CoreSet	6H	344.8	TCGATGAGGACCTTGTTTCC	TAGGCAATCAAAATGCACCA
1072	ICSB0808	CoreSet	6H	351.7	CACCACCCTGAGTGACATTG	CTTGGGGATCTGCAGAGTTT
1073	ICSB1141	CoreSet	6H	352.4	TCCACCTCCTCCTAAAAATGG	TCCTACATGATGCAACACGAC
1074	ICSB0809	CoreSet	6H	355.0	GCGCCATTATTTGTTGAGGT	TTCCTCTTGGCATCAGGTCT
1075	ICSB0810	CoreSet	6H	359.3	GCTTACGGTTACAGGGGAAA	CGGTTGCTGTCTCTTGGACT
1076	ICSB1293	CoreSet	6H	361.5	TGATTGGGGCTTTGAAGAAG	TGAGTAGGGTAGAAGGCCCTA
1077	ICSB0811	Other	6H	369.3	TCTGTCAAGCAAAGGCAACTT	GGTGGCATCTCTGTCTTTGTC
1078	ICSB0812	CoreSet	6H	373.4	GGTGGTGATGAAAGCTCGTT	CCTTCCTCCGAGTCACTGAA
1079	ICSB0072	CoreSet	6H	374.9	TACCAGCAGGATTGTACTGATACG	AAACATAAACACTGCTCAAGCTCA
1080	ICSB1142	Other	6H	375.9	GTGAAGGTGGGCATAGGAAA	ATAATGTGGCAGATGCGTTG
1081	ICSB0813	CoreSet	6H	379.3	TCCGATTAGAGACCGCACTT	AAACAGTAGCAGAGGCTTACTCG
1082	ICSB0814	CoreSet	6H	385.3	TGTGATGCCCTAGGACTGTG	CATGTTCATCCAACGTCAGC
1083	ICSB0815	CoreSet	6H	387.1	GATGTTGGGTTGCTCATGC	TCCACCACCACATTGAACAC
1084	ICSB1143	Other	6H	393.9	GGTTCCTCACGTATTTGCAT	CCCTTAGTAAAGATTGATATGGTTGA
1085	ICSB1294	Other	6H	394.1	GGATCGAGGAGCGACTGA	GGCGGAGAAGTTGGTGTG
1086	ICSB0816	CoreSet	6H	396.2	CCCTGTTCAGTTGCACACAT	CTGGGCTCAGATTCATCCAT
1087	ICSB0817	CoreSet	6H	399.4	CAAATGGATAACAACAGCTCCA	TTGCATTGTTTTGCTTCTGTG
1088	ICSB0818	CoreSet	6H	410.0	TGCTGCAAGGGAACTACCTC	GAACTTGTGCGGACACTTGA
1089	ICSB0073	CoreSet	6H	427.8	GTCTCTGAAACTGCTGAGTGATGT	GAAAGAGCATTATGAGGGGAACTA
1090	ICSB0819	CoreSet	6H	436.3	TCTTGTGTCAGCATGGTTCC	CAGAATTCAGAAAGCTGTTCCA
1091	ICSB0820	CoreSet	6H	440.8	CAATAACTCGCACAGCCATT	CAAGCGGCTGAAGTTCTTG
1092	ICSB0189	Other	6H	444.3	GCAACCTGAATATTTCAATTCCTC	CAAATCTGGACAGATGTTTGAATG
1093	ICSB0230	Other	6H	444.7	TATGTTATGGTTCCACCATTTGAC	CTAAATGAGAAAACAGGTGCCAAC
1094	ICSB0821	CoreSet	6H	444.8	ACAGGTGGTGTCATCGCATA	TCCTGAAGAAGTCCAGGAACA
1095	ICSB0822	CoreSet	6H	451.3	AACGGCCGCTGTATCAATAA	TGATTCGGTTCCTTCTCAGC
1096	ICSB1144	Other	6H	453.6	CTTCTGTCTCCGCAAGATCC	CATCATCACAGAGGCAATGG
1097	ICSB0823	CoreSet	6H	471.2	TGGACCCGGTATAGTTGTACG	TCATGTTTTGAGCGATTCTGA
1098	ICSB0824	Other	6H	474.0	GCAAAACTGTTCACACGACAA	AAAACGGACTTTCCAACAACA
1099	ICSB0825	CoreSet	6H	477.2	CCTTCTGTTCCCGATTGTTT	TGTTCTGTGTTGCCTTCGTC
1100	ICSB0826	CoreSet	6H	481.7	CAGTGGTATGCGGACAAAAA	GCAATGTCACGGTCTGCTAA
1101	ICSB0827	Other	6H	487.4	CCGACGAGTTAGCTGTTTGC	TAATCCTGTCCCTGCCTGGT
1102	ICSB0828	CoreSet	6H	491.0	GACGAGGTGTTTGTCCCAAG	GTGAGCTGCACGATGATGAG
1103	ICSB1145	CoreSet	6H	493.5	TTCAACTTTTCCCCCTTCTT	CAAGTAATATATCATAGCATGGGGACT
1104	ICSB0829	Other	6H	494.0	GGCCATGTAACCTCTGGTTC	ATGGATTTCGACCCCAAGAT
1105	ICSB1295	Other	6H	497.1	TCCTCCTCCTCCTCCTCTTC	GGCGGGGGAACTCTATAAAA
1106	ICSB0074	CoreSet	6H	498.6	GCAGATGATGGGATTTTGAAATTA	AACATTTTCCAACTGTTGCTGAT
1107	ICSB0830	CoreSet	6H	498.6	AGCAAGTTCCGGTTTTAGCA	AAGAGCGTGGTAAGCTGACAT
1108	ICSB0831	CoreSet	6H	503.9	TTTATCTTGCCCCAGCTGAT	CAAGCCCTTCAATGATGAGAA
1109	ICSB0832	Other	6H	506.2	AGCTGGCCTTGCTGATCATA	TGACCTTTTGCACATTCCTG
1110	ICSB1146	Other	6H	510.0	TGTTCTCCACCTTTGGTTCG	GCAACTACTCGCGAAAATGG
1111	ICSB0833	CoreSet	6H	517.3	GTGGTGCCTTTTCTGATCGT	ATGTTTCCAACAAGCACAGC
1112	ICSB0258	Other	6H	521.1	TTGCCTGATGAAAATCTCTTCC	AGAAGAGGAGGGAAACACATCAT
1113	ICSB0075	CoreSet	6H	521.6	CTCAATCTGAGGTTTCCTGTCCT	AAGCAAGCTTGTTAAGGTGAAGAT
1114	ICSB0834	CoreSet	6H	521.6	AAGATCCTTCGGGCACATCT	GATCCCGTTCTACCGAGTTG
1115	ICSB0835	Other	6H	526.5	GAATTACACGTGCGTTATACGTG	CAATTGTCCCCACAATGCTC
1116	ICSB1147	CoreSet	6H	529.9	GCCTGTCAACACCACTGTGA	TCCCGGAAGTCTGAGATATGA
1117	ICSB0836	Other	6H	530.3	TGATCCCAATTGACTTGCAC	TCTTTAGTCTGCTGCCGTTG
1118	ICSB0837	CoreSet	6H	534.7	AGTAAAACCGCACGCACAAG	TTTCTGAGACACTGGCTTGG
1119	ICSB0236	Other	6H	535.6	CAGTGTCCATGTCCATGTGTGT	AGAGCGGATCACTTGTGAGATAAG
1120	ICSB0838	CoreSet	6H	540.6	CAAGAGGCGATTATCGAAGC	GTCTCTCACGGCGCTCAT

Supplemental Table 1. List of amplicon sequencing primer sets for barley.

No.	Name	Type	Chr	Pos[Mb]	F_primer	R_primer
1121	ICSB1148	CoreSet	6H	543.9	CTTCAGCGGCACTGTAGATG	AGGGCATAATTCCAACAAGG
1122	ICSB0076	CoreSet	6H	544.5	GTGTCAAAACAGGAAGAGTGAATG	GTATGAGCTTCAGAGAGATCAGGG
1123	ICSB0116	Other	6H	544.5	AACTTTGACCTGCTTGATTCTGAT	ACCACATAGACCTTACCAACTTCC
1124	ICSB0117	Other	6H	544.5	GGAAGTTGGTAAGGTCTATGTGGT	TGATGAATCTCTCTCCAATGTGAT
1125	ICSB0839	CoreSet	6H	548.2	TGGCTCTCAAAGTTGGAGGT	CCCCAAACTTTATTTCACTCTCA
1126	ICSB0228	Other	6H	550.9	CTCTTGTTCAGAGTTTGTTCCATC	TATTAGATGTTGCACGGGACTAGA
1127	ICSB0840	CoreSet	6H	553.9	ACAGCTGTAGCTTCGCCATC	AACAATGCAGGTCTCGTGGT
1128	ICSB0841	CoreSet	6H	562.8	AGTCCATCAGCTACCTACCTTG	TTCCGCACTTCTGGCTAGTT
1129	ICSB1149	CoreSet	6H	566.4	GCGTACGCATACCCTACCAC	GCTGACTTATCGGCACCAC
1130	ICSB1150	Other	6H	566.4	TCCCTCTGCACCTAAATTACTG	CGTGGTTCCTTTTATTACCAGA
1131	ICSB0842	CoreSet	6H	571.4	AACACAGGACATCCGACACA	CGAGAGGCAGACGGTATGAT
1132	ICSB0843	Other	6H	576.4	TTACTGCAGAGAGGCGTCAA	TCCAAACGATGAGAGAGACAGA
1133	ICSB1296	CoreSet	6H	576.6	TCGGAAAATTTCGCTTCATT	TTGACAAGATCCCTGCCACT
1134	ICSB1297	Other	6H	580.1	CAAGTTTGCTCAGCTGGATG	CCGGGTATGTTTCCATCAAC
1135	ICSB1298	Other	6H	580.1	GCATTTTCGTTATAGCCCCTTT	TTGGTACAGGTGCGGTGTTA
1136	ICSB0844	CoreSet	6H	580.2	GGCGACGGAGACTATGACA	CCATCATCAACATTGCCATT
1137	ICSB0077	CoreSet	6H	581.6	ACTCCAGTTAAGGAGAAGATCGAA	CGATATGCATTAGCATCTGTCATAA
1138	ICSB0139	Other	6H	581.6	AGGCTGACCCTAGGGAAGATAC	CCACATGTGATTCTATATTTGGGA
1139	ICSB1151	Other	6H	582.2	CGAATCGACGGAAGAGGTC	CTCGTCGCCCTGGTCATC
1140	ICSB0845	CoreSet	6H	582.4	TCAGAAAGCATTTACCAAACGA	TTTACTGTGCCCCCTTTGTC
1141	ICSB1299	Other	6H	583.3	ACCGCATCCTCCTCCTCT	AGCTCAGCTCAGGGAATGAA
1142	ICSB1300	Other	7H	0.2	CGGCTTTTGAGGATCTCGTA	AAGCTATGGAGACGCTGCTG
1143	ICSB0078	CoreSet	7H	0.6	GCAAGGATAACAGCTGGTCTAAAT	TCGAGCTCATATGCTCACTTTTAC
1144	ICSB1301	CoreSet	7H	0.7	TATCGCAGGTACGTCGAGTG	CGCTCTCCTGATGCACTGT
1145	ICSB0261	Other	7H	0.8	TGTTTGTTTTCTCCCTTTACACAA	GATGGCGAGAAGTAGCTCTGTT
1146	ICSB1302	Other	7H	3.5	GACGGCATATTTGCTTCCAT	TTGTGCCTTACCCCTATTCG
1147	ICSB0846	Other	7H	4.4	TGAATACACCACAATGCTGAAA	CCTTGCTTGGGTGCTACTCT
1148	ICSB0847	Other	7H	8.9	TGTTTGGAACCAATTGAAAAGA	CACGTGTTCATGAAGTGTTGG
1149	ICSB0848	CoreSet	7H	13.4	GAATGCCTTTGCTTCAGCTC	ACCCTACCGAGGAAAGTGAC
1150	ICSB0849	CoreSet	7H	17.7	ACTTCACTCACGCCTTCGTT	GCGGAGAGTTAACGGAATCA
1151	ICSB1152	Other	7H	18.7	TGAACATCATCAATCATGACAA	TGCTGAGTTCGGAAACTTGA
1152	ICSB1153	Other	7H	18.7	TCAAGTTTCCGAACTCAGCA	TCTCCTGTGGTCATTATTTCCTG
1153	ICSB1154	Other	7H	18.7	AATGCAAGGACGAGTCTAACG	AATACGTCGGCCACTCGAC
1154	ICSB0850	Other	7H	21.2	CTTTAAGCTCCGCTGCCTCT	TGTTTCGGTCAAAGCAAAGA
1155	ICSB0851	CoreSet	7H	27.5	CTCGGACGATTTGGCAATAC	CCACAAAAATAATGCAACACCA
1156	ICSB0852	CoreSet	7H	30.2	CAGCTGCAACCACACAAGTT	CACCCTTACCCTTGACAGATG
1157	ICSB0853	Other	7H	36.9	CATTCGAGGCTCAGGAAAAC	ACCGTATCCTCCATGCAAAA
1158	ICSB0079	CoreSet	7H	41.8	AATGTACAGGGGTATCCTCCTTC	GACCAACCCTACGTCATGTAGAC
1159	ICSB0854	CoreSet	7H	45.2	TGGTAACCGCTGAAGGAGAC	GAAGGCGTTACCTGATGGAA
1160	ICSB0855	CoreSet	7H	49.5	GCTCAATGGCAACATCAAGA	CCTCGTCCTGTGTCCTCAAC
1161	ICSB1155	Other	7H	52.4	CTCCAGCAAAATGCACAAAA	TTTTTGTCTCCATCCGTAAGAAA
1162	ICSB0080	Other	7H	54.7	CACTTAGCGATGCAGTGAACTATT	TTTCTTCGGTTACTTGGAAACAAT
1163	ICSB0099	Other	7H	54.7	ATTGTTTCCAAGTAACCGAAGAAA	TCTAGATCAGTAAGGTTTGATCCG
1164	ICSB0100	Other	7H	54.7	ATTGTTTCCAAGTAACCGAAGAAA	TCTAGATCAGTAAGGTTTGATCCG
1165	ICSB0856	CoreSet	7H	59.1	GTAGTTGGCTCGGGTGGAC	ATGGCAGAGCAAATCCTCAT
1166	ICSB0857	Other	7H	68.4	CATAGAGTGAAACGGGTGGTG	CCAAAGTGAGCACATTCGAC
1167	ICSB0081	CoreSet	7H	75.9	GGCTTAAATTAAAAGGTATGCATGT	CAGTCAATTTCCACTACCAAATCA
1168	ICSB0186	Other	7H	75.9	CAGATAGGTCTTTGCAAAAGGG	TACTCAGATTCAGGTTGGAAACAG
1169	ICSB0858	CoreSet	7H	75.9	GAAACATGGGCCTTGTCG	GAGGAGCAACGAGGAGTCAG
1170	ICSB1303	CoreSet	7H	80.1	CCGTGACCTGGATCTGGAC	CCGGCAGTACCGTCTAGC
1171	ICSB1156	CoreSet	7H	85.2	TTCCCTTTATGCTGACGACAC	AGGATTAGACACCCGACGTG
1172	ICSB0859	CoreSet	7H	85.9	GTTGTGTTCGTCCTGGTGTG	GGCAAGACCACCATAAAGGA
1173	ICSB0860	CoreSet	7H	96.4	TGCCAGTATGGTACCCTCATC	GCTATTAAGGAGCACCCATGC
1174	ICSB0082	CoreSet	7H	98.7	GCTGCCTTAAAACGAGTATTGAAA	AGCTGATGTTGATCTCTGGTTTTT
1175	ICSB0133	Other	7H	98.7	CTAAAACAGAAGCTGTGTTGGTCA	CCAACAGAATGTGAAGTTTGAGAG
1176	ICSB0134	Other	7H	98.7	TCAGAGGCTAGCTATTTTAGTGGC	TCTCAGGGAACTACTCATATTCTGG
1177	ICSB0135	Other	7H	98.7	AATTTGAAGAAAGTTCCTTTGCAG	CTCATTTTCAAAAGAACCACACAC
1178	ICSB0136	Other	7H	98.7	TACCAAACAAAGGCTGTTCCTTAT	ATGTGCAGATGATTCATACAGAGG
1179	ICSB0137	Other	7H	98.7	GCCAGCTGTCCATCTGTAACTAT	AAATTTACAGCTGTCTTTGAAGGG
1180	ICSB0138	Other	7H	98.7	AAATAAACACATCCATCAAGCAAA	CTCTGAAGTCTCACTCCGTTATGA
1181	ICSB0861	CoreSet	7H	103.7	GAGGTAAAGGACACTGACACATTG	AGCTAAATGATCTCCGGTCA
1182	ICSB0862	CoreSet	7H	110.5	CATGTTCGAGACCCAGCAA	TCAGTTGTCCTATTTCTTTTTGG
1183	ICSB1157	CoreSet	7H	112.8	GGCTCATTCACAGGACACAA	ATGCGTGCTCCTTCTCCTAC
1184	ICSB0083	CoreSet	7H	114.7	GCAAAGTGAAGTTAATGTTGTTGG	AAGTCACTTGAAGATCCTTTGGG
1185	ICSB0153	Other	7H	114.7	ATATATCCTTTGTAGGTCCGGTCT	TCTAATTCCTTCAATACACCACCA
1186	ICSB0154	Other	7H	114.7	CCAGACTCCTCACCAGCTAAAC	ACTACCGAGTGTAGTTCCTGCTTC
1187	ICSB0155	Other	7H	114.7	AAAGACCATTCACAACGAAAATGT	CATGTGATGCTTTCAATATTCCAC
1188	ICSB0156	Other	7H	114.7	GATGGTTCTGAGGAAGCACTTAAT	AGGATCCTTCGTTACTTCACAAAC
1189	ICSB0157	Other	7H	114.7	GAAGACTCAGATGAGGACGATTCT	CATCTCCTGGTTCTGCTTCTCTA
1190	ICSB0863	CoreSet	7H	122.2	GACGCACATGTTGCATATACAGT	AGGTGGGCAATTGGTATCAC

Supplemental Table 1. List of amplicon sequencing primer sets for barley.

No.	Name	Type	Chr	Pos[Mb]	F_primer	R_primer
1191	ICSB1304	CoreSet	7H	122.2	GACCACCTTGCTCCCAGTC	TGGTGAATGTGTGGGCTATC
1192	ICSB0864	CoreSet	7H	126.3	AAGTTTTGTGGTCAGCCTCA	ATCTGGTTTTGGTCCTACCG
1193	ICSB1158	CoreSet	7H	132.6	TCTGAGCTAGAACGCAGGTC	GCTTACCACACCAACCCACT
1194	ICSB0084	CoreSet	7H	132.9	TGGGAAAGTATGGTCGTTTAATTT	CTTCAGAGTTTTACGAGATACCCA
1195	ICSB0865	CoreSet	7H	141.9	TCGGAAACAGTTCAGTGTGC	GAGGACCTGGAACAAAAGCA
1196	ICSB0866	Other	7H	147.4	GCATGCTTACCCTGACAGC	CTTTGGTAGCCTGGGAACAA
1197	ICSB1159	CoreSet	7H	149.9	CATTTGCACCCGAAGTTTTA	GAATTTGGGGTTCGAAAAGC
1198	ICSB0867	CoreSet	7H	154.2	TTGGCCAACTTTGCATGTTA	TGCATTAAGATCACCAATGTCAA
1199	ICSB0085	CoreSet	7H	156.1	TCATCACAGACCATGCAAACTACT	GAAGGAGACTTATAAGATGGAGCG
1200	ICSB0161	Other	7H	156.1	AAATGTTCAATCAGGGATTCAGAT	TTGAAATGGATTCCCTTGAAAT
1201	ICSB0162	Other	7H	156.1	CATCTACATTGTTGTCAGTCCCTG	AGAGAATGCAGAAGCAGAAACAG
1202	ICSB0868	CoreSet	7H	161.0	AACGGTGAAAATCTGGAAAGAA	TCCTGTAAGGCGAAAGAACG
1203	ICSB1160	CoreSet	7H	169.6	TCTCCAGGGAGGTTCATGTC	CTTGTTTTATCTAGGAACCGCATC
1204	ICSB1161	CoreSet	7H	192.7	CTTCCCCTTGCCATCTTCAT	CTGATGAGCATCTGGGATTG
1205	ICSB1305	Other	7H	200.2	TTCTCGTTGTCGAGGGAAGT	AGCGATTTGCTTGCTTCTTC
1206	ICSB0869	Other	7H	206.7	CCTCTCTCGAGCACTTCCTG	TCCCGTTACTGATGCATTGA
1207	ICSB1162	CoreSet	7H	210.5	TCTGCAACACTGCATGATTT	CTGGAGAAGTGGAGCCAATC
1208	ICSB0870	CoreSet	7H	215.0	ATGCTCTTTGGGCTCTCTCA	AGGAGGCCACACAGTTCAAG
1209	ICSB0871	CoreSet	7H	222.7	CATAGCAGTTCCCAGCCAAC	GTCCCAACAAGGCATGAAAT
1210	ICSB0185	Other	7H	225.3	AGCATAGCATGCAAACTCTACTTG	GATAGGTAGGAGCAGCAGATGAGT
1211	ICSB0872	CoreSet	7H	225.6	TTGTGCATTAGTGAAACATACGAA	CTCAACCTGCCAGGAAACTG
1212	ICSB0226	Other	7H	228.8	ATTACAACCACAACAGGCAAAT	AAAGTAGGGGACATGGTTGCAT
1213	ICSB0873	CoreSet	7H	229.6	AGGGTTTTCCATGACCCTTT	TTCAATCAGATGGCTGAGCA
1214	ICSB1163	Other	7H	229.6	GAACAAGTGTAGATTAGGGGAGTG	AAACAGATGCGACGGTTAGC
1215	ICSB0874	CoreSet	7H	243.1	GGTGCTCGCATGGATAATCT	GTTACTGCCACCGCTCAAAT
1216	ICSB1164	CoreSet	7H	250.2	TTCTGTTTTTATCGATGCCTCTC	CAACAATCAACTCCCCCAAT
1217	ICSB0086	CoreSet	7H	251.1	TGTTCGTTATTATATGGTGGGTTG	CGCACGAAAACTAGGAACTTAGA
1218	ICSB0875	CoreSet	7H	251.1	CCGCACGAAAACTAGGAACT	TGGTGGGTTGGTGATTTTCT
1219	ICSB0876	CoreSet	7H	255.3	CATCGGGGACTACAGATTCG	TCTCTTGTCAGGGCACCAAT
1220	ICSB0877	Other	7H	261.4	GCCTCTTGCTAGCTTTTAGAACA	TCATGTAGCAAGTGGGTGCT
1221	ICSB0878	CoreSet	7H	265.7	AAAGATTGAAGCACGCCAGT	CAAGACCTCCCTGTGAAACA
1222	ICSB1165	CoreSet	7H	270.1	CACCGATGTGGAAAACTCG	TCACAAAGTTTTTCTCCCCCTA
1223	ICSB0879	CoreSet	7H	273.2	GAAATCCACGTACTCCACACC	TGATCCAAAGGAGCTTGCAT
1224	ICSB0880	CoreSet	7H	281.0	GGATAGGAGTCAGACGTGGTG	CAGCACATCGACCCAAGTAG
1225	ICSB0881	CoreSet	7H	287.5	GTGTGGCATCAAACCCTCAT	AACAACATCCTTGTCCAGCA
1226	ICSB0882	CoreSet	7H	290.9	AGAGAAGGTTGGCGAGATGA	GCGACAAAGCACAAGGAGA
1227	ICSB1166	Other	7H	291.3	CTTACCAAAGGCGCTTCACT	CCTCATTGGGTTCGACACTT
1228	ICSB0883	Other	7H	297.8	TTCAAACCCATCAAGTAAGATTC	GGTTGCTAACTTTGGCCAGATA
1229	ICSB0884	CoreSet	7H	305.3	CCAGATGTGATTTTGGAGCA	CCAATATCTGAGGTGCATTCG
1230	ICSB1167	CoreSet	7H	309.6	TTCATCTTGTGTTGGCATCC	GCTTTGGAATTGGCTAGCTCT
1231	ICSB0885	Other	7H	317.7	TCCCAAGCTATCGTCACTCA	GGCAATTCCTAAGAGCCAGA
1232	ICSB0087	CoreSet	7H	323.7	TCAAAAGGCCTATGTCTTACATCC	GGCACGAATAAATCCTCTTTTCTA
1233	ICSB0165	Other	7H	323.7	AAATAGAACAGTTTAGTCCGCAGG	TTTATCCGGGATGTAAGACATAGG
1234	ICSB0886	CoreSet	7H	328.2	CCAACCTGGACACAGTGCTA	CGAGGCAAAGGATAGTGAGG
1235	ICSB1168	Other	7H	330.4	TGCTCAATCCTTCTGCTCTTC	TCCATCTTCAGATCCTGACATC
1236	ICSB0887	Other	7H	333.8	GGAGACATGGTCACAGCCTTA	GGCACGATTTCCAATTCTTT
1237	ICSB0888	CoreSet	7H	338.5	ATAGCTCCGGAGTGGAGAGC	GGTTCCGGTATCTCCAAGAA
1238	ICSB0889	CoreSet	7H	346.2	ATTGGCACCCAGCTGTATCT	TCAAAATTAGGCAGCTAAAGTGAA
1239	ICSB0890	CoreSet	7H	350.3	AGGGTTCTGGTCTGACTACCA	TCATGATTCGTCCGATACTGAG
1240	ICSB1169	Other	7H	350.5	ACCAGGAAAATTCTAGCAAGTCA	GGTTTTGGGGGTACTTTCGT
1241	ICSB0891	CoreSet	7H	354.9	TCCTCTTTGCCATTTTCCTG	CCTTGTATATGGTGGGGAAGA
1242	ICSB0892	CoreSet	7H	363.3	GCGTCACTGTCACAAGTCAAA	GGTCGCAGGTTTAGCAGAAG
1243	ICSB1170	CoreSet	7H	371.7	AAGCAAGCAACCCCTTGAAT	ACCCACCATAGTGGTCATCG
1244	ICSB0893	CoreSet	7H	386.9	TCAGAAAGCTCAAGGCAGAA	TCCTACAGCTGATTCAGAGCA
1245	ICSB1171	CoreSet	7H	391.2	CCAATTGGGTCACCATAAAC	AGACGGCAGGCCCTACTAAG
1246	ICSB0894	CoreSet	7H	393.6	TGGAGGTGCTCAGACTCAGAT	GTGTTGCTCATGCATACACATA
1247	ICSB0895	CoreSet	7H	398.2	ATGGAGTCGCTCAGCAGCTA	CCCCCTTCTCCAAAAGAAAC
1248	ICSB0896	CoreSet	7H	401.0	GCCGTGTGGGAGATATCAGA	GGATGCAGAAAGGGATGAAG
1249	ICSB1306	CoreSet	7H	401.4	CAGAGATAAGGGCGTTCATCA	GCCTTTTGTCCAGCAGCTTA
1250	ICSB0897	CoreSet	7H	407.3	CTTCACCAAAGAGCGACACA	CATAGAAGTGTTTGCCGATGC
1251	ICSB1172	CoreSet	7H	409.7	GGGGCTCTTCTTCAGACACA	TCTGTTTTCTGGCCCTTCAC
1252	ICSB0898	Other	7H	418.0	GGCAACCATACTGGCAGAAC	TGGCTTGCAGCTATTCAAGA
1253	ICSB0899	CoreSet	7H	427.0	AAAAACGTGATAACGAATAGACCA	TCTTGCAGACCCTCCAAATC
1254	ICSB1173	CoreSet	7H	433.6	AAACCGTCCAAAAGAACTGG	AGAATTCATCCTCATTTTTCTCG
1255	ICSB0900	CoreSet	7H	445.1	CAATGCATTCGCAGCAATAG	GCATTTTACCGAGGCCAGT
1256	ICSB0088	CoreSet	7H	453.5	CTTCACCACCTCTTGGAACTTG	AGGTATTGAACTTCAGCGACCAT
1257	ICSB0901	CoreSet	7H	455.7	GGGGAAAGAAAAACAGTCTCC	ACCGTGTACCATTATTTACAGTCA
1258	ICSB1174	Other	7H	457.9	TCACCAACACACCCAATACAA	CCTTGCTGTTTTTATCGTTCG
1259	ICSB0902	Other	7H	461.0	GCTCTTGTCTTCGTTCTCAGC	ACAGGAGAACTCATCGATCTTC
1260	ICSB1307	CoreSet	7H	462.6	ACCATCCCTTCTCCTCCTGT	ATAACCAGCGGCCGAAGTA

Supplemental Table 1. List of amplicon sequencing primer sets for barley.

No.	Name	Type	Chr	Pos[Mb]	F_primer	R_primer
1261	ICSB0903	CoreSet	7H	467.1	TGGGTCCCTGATGCCTAAAT	CAAAGGAGAAAGTGGCATCG
1262	ICSB1175	Other	7H	478.2	CCACTTTAGGTACCGTTGTGAA	TTTGATGAACCTATGAGTTGTATCG
1263	ICSB0089	Other	7H	486.2	AAAGAGTATTCCTTGAACAGAGGG	TACCAGACTTATGATCTCTCCACC
1264	ICSB0904	CoreSet	7H	487.9	ATCGATCAGGCCAGAAGAGA	TTGTTTCCTTGGATGTTGGAG
1265	ICSB0905	CoreSet	7H	489.5	AAGGATACACAAGCAGGGTGA	TATGGTGCCAGTCTGCGTAA
1266	ICSB0906	CoreSet	7H	497.3	TTCCAGATGCAGTTCTGGTG	GTATGCGCCGGTACTTGTCT
1267	ICSB1176	CoreSet	7H	499.2	TGGATGATGAGTGGGAACCT	CACAAAGCATTGCATGAAACA
1268	ICSB0907	CoreSet	7H	500.4	GTGTCCATGGAAGTCCCAGT	AAACGGTTGGAGATCGATTG
1269	ICSB0908	CoreSet	7H	504.5	GGAGACCTTCACGTCCAACT	ATTTGACAGCGGCTGGAT
1270	ICSB0909	CoreSet	7H	513.3	CGAGTGGTAATCCTCCCAAA	TGAGATGCCCGACAGAGG
1271	ICSB0910	CoreSet	7H	521.7	CACCAGACAACACAACGTCA	ACAGAGAAGGGAAGCTGCAA
1272	ICSB0911	CoreSet	7H	525.9	TGCTCTCGTAACCCCAAAAC	CCAGTTTCGCTTGGAGTTAAA
1273	ICSB1177	Other	7H	527.2	GAGGATGTTCCTCCTGTTAAAAA	TTTTGGATTGGCACAAAAA
1274	ICSB0912	CoreSet	7H	531.0	CAGCATAGACAGGACCAATCAG	TGCTGACTTATTTCAACAATCAAA
1275	ICSB0913	Other	7H	535.4	ACCCAACCCAGCTACTGATG	ATGGCCCCACTCTCAAAGTT
1276	ICSB0914	Other	7H	553.4	GGGTAACAACGTGCAGCAG	GGCACAAAAGGCCATCATAC
1277	ICSB1178	CoreSet	7H	558.0	TCAGGTGATAGTTTGCCCATT	CCATCTGACCGTTCTTGGAT
1278	ICSB0915	Other	7H	569.5	AGTCTGGCTCGAGGGAATAA	TCGCTAATGTTGTCTCTCCTCA
1279	ICSB1179	CoreSet	7H	574.2	TTGATCAGCCCCACAACATA	GGCTGCAAGATCCAAGAAGA
1280	ICSB0916	CoreSet	7H	575.8	GCGATTAAAAGCGGTACGAG	GGGAGAGAAAACCGCATAAA
1281	ICSB0917	CoreSet	7H	584.1	AAAATAGGAAACCCAAGCTATGC	CTTCAAGGCATCCAATTTCG
1282	ICSB0090	CoreSet	7H	592.4	TTTTGTGGATTCAGGAATTTATCC	GATCTATCTCACATTTCCCCTTGT
1283	ICSB0918	CoreSet	7H	607.8	TGACGGAAGGAAGTGAGGAC	CTGCCTCAACTTGGACCTGT
1284	ICSB0091	CoreSet	7H	609.6	CATGGGTACAAAACTGAGAAATTG	GTGTGAGTAGGAGGAGGAGGAG
1285	ICSB0919	CoreSet	7H	612.1	AAAGATCAGTCCTGATAACAAGTACA	TGTGCAATTCAGTTTATGCGTTA
1286	ICSB0920	CoreSet	7H	616.3	GTAGGAGCAAAGCAGGAGCA	ATTCTCCCCTAGCCGAATTG
1287	ICSB1180	CoreSet	7H	616.4	GCGCTCACCTTCTTCATCTT	AGCTGTCCAGGATGTCGTTC
1288	ICSB0092	CoreSet	7H	619.4	ATTTGAGCAGGGTATGCTTACTTC	GCGATAACTGACTCTGGGTATTCT
1289	ICSB0178	Other	7H	620.9	AATCAGGCTGTGTAAACTCTTGAA	ACTCATTCTTACAGCAATGGTTGA
1290	ICSB0921	CoreSet	7H	621.1	ATAATGGAGCCTTGCATTCG	AAGCGTGCACAACAAGAGG
1291	ICSB0231	Other	7H	623.7	GCAGCATTTTTACATAAATTTTCCT	GAAGTGGTACTTGTACCGGACCT
1292	ICSB0922	CoreSet	7H	624.6	GATTGCAGTCCAGCCAAAGT	ACCGTTCACTAATGGGATGC
1293	ICSB1181	Other	7H	627.5	AAATCGTGCTTGCACATGAC	CACCTTGCCAACGTATACCC
1294	ICSB0208	Other	7H	628.0	AAAAGTTGGACCTGTGTGATGATA	TCCAGTATGATTGTCTTGCTTGAT
1295	ICSB0923	CoreSet	7H	633.3	CTGCGAGAACGTTTGTCCTT	AAGGCAACCATAACTGAATCG
1296	ICSB0223	Other	7H	641.8	TCGACCCGTATAACGATATCCTAC	TTCCAGGGAAATTGTAAATTGAGT
1297	ICSB0093	CoreSet	7H	641.9	TTCTTCCCCTTCTTTCTACTATCG	GTGTGACATTTGCATTAGACCGTA
1298	ICSB0924	Other	7H	642.6	TGATTAACTGGCCAATTGTGT	GCAAAGGCTAACTATCACAATGC
1299	ICSB0272	Other	7H	645.5	TTAGTCCATGTAAATCAAGCAACA	CTAGGAGCAGGACAGCAACTCT
1300	ICSB1182	CoreSet	7H	648.2	TTGTTGTGAATCTCGTGTTCC	ATGTGCCAGCAATCAAGATG
1301	ICSB1183	CoreSet	7H	648.2	ACGCAAACTGTGAGCTGATG	CAAGGGCCTCGTCAATTTT
1302	ICSB1184	Other	7H	648.9	GCGCGTAAATTTTTATGGTCA	TTCGAGGAGCTCGTGAAAAT
1303	ICSB1308	Other	7H	649.4	GACACCTTCATAAAGTTGGCATC	AACAACCACTTGGCTTCAGG
1304	ICSB0925	CoreSet	7H	652.9	GTTTCCTTTCATCTCGAGTCC	TCCGAAGAGCATAGACAGCA
1305	ICSB1309	CoreSet	7H	654.0	AGCTGTGCGTGTATGGTTTG	GATGCCACCTGCACTTGTAA
1306	ICSB0094	CoreSet	7H	654.4	CATCCCTGCTGATTAAGGTGTAG	CACATGTTCACCTTCCTATTTGAC
1307	ICSB1310	CoreSet	7H	656.1	CGACGTACTTTTCACACTTCCA	CTCAAGCTTGGCAGTCATCA
1308	ICSB0926	Other	7H	656.7	TGTGCTCCTGCACTTCATGT	TGGCAGATTGGTCGGATATT
1309	ICSB0095	Other	Un	7.6	GATGTCATTTACTGCCACCATTAG	CACAATATCATCGGTTGTGTATCA
1310	ICSB0096	Other	Un	247.9	CTTCAAGTGGCTCAAGTCCAAG	GTAGTAGAGGTTCGGTTCATGGAG

Supplemental Table 2. List of barley accessions for evaluating amplicon sequencing markers.

No	Cultivar/Line	Source*
1	Shinjuboshi	TARC
2	Koharu Nijo	TARC
3	Tohoku kawa 46	TARC
4	Tohoku kawa 47	TARC
5	Tohoku kawa mochi 48	TARC
6	Tohoku kawa 49	TARC
7	Miyuki-omugi	TARC
8	Benkeimugi	TARC
9	Fiber Snow	CARC
10	Haneumamochi	CARC
11	Minorimugi	CARC
12	Hokuriku kawa 54	CARC
13	Yukimi Rokujo	CARC
14	Hokuriku kawa 59	CARC
15	Hokuriku kawa 60	CARC
16	Silkysnow	CARC
17	Hokuriku kawa 63	CARC
18	Shunrai	CARC
19	Hokuriku kawa mochi 64	CARC
20	Tosan hadaka 112	NAES
21	Tosan kawa 113	NAES
22	Tosan kawa 114	NAES
23	Tosan hadaka mochi 116	NAES
24	Tosan hadaka mochi 117	NAES
25	Tosan kawa 118	NAES
26	Sachiho Golden	TAES
27	Tochigi nijo mochi 50	TAES
28	Tochigi nijo mochi 53	TAES
29	Sukai Golden	TAES
30	Tochinoibuki	TAES
31	Tochigi nijo 49	TAES
32	Asuka Golden	TAES
33	New Sachiho Golden	TAES
34	Kashimamugi	NICS
35	Suzukaze	NICS
36	Sayakaze	NICS
37	Kashima Goal	NICS
38	White Fiber	NICS
39	Harushirane	NICS
40	Kanto kawa 98	NICS
41	Kihadamochi	NICS
42	Sachikaze	NICS
43	Kanto kawa 103	NICS
44	Kanto kawa mochi 104	NICS
45	Ichibanboshi	WARC
46	Toyonokaze	WARC
47	Haruhimeboshi	WARC
48	Daishimochi	WARC

Supplemental Table 2. List of barley accessions for evaluating amplicon sequencing markers.

No	Cultivar/Line	Source*
49	Shikoku hadaka mochi 129	WARC
50	Shikoku hadaka 132	WARC
51	Shikoku hadaka 133	WARC
52	Shikoku hadaka 134	WARC
53	Shikoku hadaka mochi 135	WARC
54	Shikoku hadaka mochi 136	WARC
55	Shikoku hadaka 138	WARC
56	Yumesakiboshi	WARC
57	Kirarimochi	WARC
58	Shikoku hadaka mochi 137	WARC
59	Mannenboshi	WARC
60	Houshun	FARC
61	Shunrei	FARC
62	Harumiyabi	FARC
63	Harushizuku	FARC
64	Nishinohoshi	KARC
65	Haruka Nijo	KARC
66	Kusumochi Nijo	KARC
67	Shiratae Nijo	KARC
68	Saikai kawa 76	KARC
69	Saikai kawa mochi 77	KARC
70	Nishinochikara	KARC

* Abbreviations are same with the Table 1.

Supplemental Table 3. List of amplicon sequencing primer sets for barley functional genes.

Gene	Marker_name	F_primer	R_primer	Reference
Ppd-H1	PpdH1_1	CCAACATGTTTCCTCTTGGAG	TAGGAACTCCTCCCAGCATC	Turner et al. (2005)
	PpdH1_3	TCACGAGGTGCAAAGCATAA	GATTGGAGACCGCACACATA	
	PpdH1_4	GCACTGCTCAGTCCAGTGTT	GTGCTCCCGTTATTGGTGTT	
GIGANTEA	GI	TGTGTTCCCACATGAGAAATG	AGCCAAGGCTTCAACTGCTA	Dunford et al. (2005)
Cxp1	Cxp1_1	GCAAATGCTTCCTGTGGTTT	CGCATCATCAAAACTAAATCG	Potokina et al. (2006)
	Cxp1_3	CTTTGATGCTTCTGCTGTGG	TGAAAGCTCTTCTCTCGTGATG	
HvEIF4E, HvPDIL5-1	Rym4/5_1	GGACCTTCTGGTTCGACAAC	GGGCTACCTCCAGAAGTCCT	Yang et al. (2017)
	Rym4/5_2	TCATCACCCTAGCAAGTTGAA	ATTTCCCTTTGCCACAACTG	
	Rym4/5_3	CTGGCATTGATTGGTGAACA	TTGGCAGCATTTTTAGTCCA	
	Rym4/5_5	GCTGTGGCTTGACTGACACT	CCCAGGATCAAACCGTGTAG	
fra	Fra	GGGCATGCATTACCTTTGTC	AGCATTCATCTTTGCCGTTC	Saito et al. (2018)
Lox-1	Lox-1_1	ATGTCCTGGAAGGAGTCGAA	CTTCCTGGGCTACTCCATCA	Mechelen et al. (1995)
	Lox-1_2	GAAGGTGAGGCCGAACTTG	GTACGTGGTGGTGCAGACAA	
CBF	CBF4	TTCTTCCAGTAGCGTGTCCA	CAAACCACTGCTCCTCGTC	Knox et al. (2010)
wax	WAXY_1	CCTGTCACTTTGCCTCGTTA	TACGACATCACCACCGTGAG	Domon et al. (2002)
	WAXY_2	GGACCAGAAAAGTAGGGGTTG	CGGCACAGACTATGAGGACA	
HvDIM	HvDIM_1_2	ACTTGCCCGATGAACCTCT	GAGCCCGTAGATGTGAGAGC	Dockter et al. (2014)
	HvDIM_3	AAGGGCGAGTTTGTGGAGTA	CCAAAGAGGAACCTGAACCA	
	HvDIM_4	GTGACGGAGCTGAACGAGA	GCCCCTTCTTGCTCTTGTAG	

Reference:

Dockter et al. (2014) Plant Physiology 166: 1912-1927
Domon et al. (2002) Genes Genet Syst 77: 351-359
Dunford et al. (2005) Theor Appl Genet 110: 925-931
Knox et al. (2010) Theor Appl Genet 121:21-35
Mechelen et al. (1995) Biochimica et Biophysica Acta 1254: 221-225
Potokina et al. (2006) Funct Integr Genomics (2006) 6: 25–35
Saito et al. (2018) Theor Appl Genet (2018) 131:353–364
Turner et al. (2005) Science 310:1031-1034
Yang et al. (2017) Theor Appl Genet. 130(2):331-344

Supplemental Table 4. List of amplicon sequencing primer sets for wheat.

No.	Name	Type	Chr	Pos[Mb]	F_primer	R_primer
1	snp1375	Other	1A	1.2	TTCTTCATGTCATGTAACCATTGATT	AGAGTATAATCAGAAGCTAGAACAGAGC
2	tarc0410	Other	1A	2.5	CAATTAAGTACATGACCAAATCATCG	TACATAGAGAAGGAGAACTCAAGATCTG
3	snp1603	CoreSet	1A	3.4	GGGGTATGTAGCGAGTTCGA	CTCAGAACACACACCCAAAACA
4	tarc0428	Other	1A	3.4	AGTTCCAACCTTCACACTAGGACA	AAGGCTATTTGAGCTTGACATCG
5	tarc1609	Other	1A	3.4	AAGCGTGCTCATATGTCTATGAATG	CAGAAGTTATGACCACAGGGAGTAG
6	tarc0427	CoreSet	1A	5.5	ACCGAGTCACACAACCCAGC	TTTTGTTTTACTCATTCGTGTTTGTC
7	tarc0445	CoreSet	1A	7.2	GGCTGGCTGCTTTGTAGTCA	TAGTTGGAGGATTTGAAAGGAAATAT
8	tarc1601	Other	1A	7.2	TCTCTCAGCTCAGCAATTACAACC	AACATCATCGGCTGCTTTTG
9	tarc1599	Other	1A	7.7	TTGAGCTTGGCAGACTAGACAATT	CATGCTAAAGACACAGTAACTGTAATAG
10	tarc0407	Other	1A	10.1	GTTTCATACCTGTGCTGTGACGA	CTAACACCCCAAATGAACCGT
11	tarc0412	CoreSet	1A	12.2	CATAAATCAAGTAATTAGCAACAGAGAC	CACAAGTGAGTGGTGCATTTACAGT
12	tarc1600	Other	1A	12.2	CAGATACTTCGCGTATCAGGTAGC	CATCGAAAGAAAACATGGAGATG
13	snp4678	Other	1A	13.1	CAAGGTTCCGACCGATGTTTC	GGATCACAAGACTTGCGGACA
14	tarc0399	Other	1A	13.1	CATGCTGATGAAGCATTGAGACA	CTCTCACAAACTCCTCATACACAGAA
15	snp2452	Other	1A	13.7	CTGATCTACATGCATATGTCCTTGT	CAGGTGAGCTTTGATCCTTGTG
16	tarc1597	Other	1A	14.1	ACAGTAATGAAGAGAAAGAACTATGCG	TTCTGATAAATCTGCAATTCTTGTGT
17	tarc1595	Other	1A	14.2	CGTCAAGCGACTTAGCAGCAG	GTTTCTTCTAGCCAAATCTACCCAG
18	tarc0438	CoreSet	1A	21.6	TATATATAGGGGCTGTCTATATTGTCGT	CTTTTGTATTCTTTGGCTTGGGT
19	tarc1598	Other	1A	21.6	CAGTATTCTCCGTCCAGCTTCAA	GACATGTTATTGGATACCCTTTTTAGT
20	tarc1604	Other	1A	24.0	GACAGATTTCTGCTTAAAATCATTCA	CTAGGTGTCAGAACCTTCATCCG
21	tarc1612	Other	1A	25.7	ATTGTATTAAGTATCGTTAGCTAGAATT	CAATAAACCTTGTAAGCAAATAGTGTG
22	tarc0443	CoreSet	1A	27.4	AAGGGAGCAGGCGTTCAAG	CAGCCAACTCCAAGGTAGCAC
23	tarc1607	CoreSet	1A	27.8	GTATCACTAGAGCAGATAAATGGCTG	GGACGCATCCCTGGAAGAAT
24	tarc1592	Other	1A	28.6	GTGTTCATTAAGGTGAATTGTTAGAATC	GTTATCACCAAAGTCTTTTGTGTCTG
25	tarc1605	Other	1A	30.8	GCAGAAGCAACCGTGACAAAC	CCTTCTGGGGAATGCATGATA
26	tarc0449	Other	1A	32.1	CACGGGCTTATCAACTAGGTCAC	TTCGTGTGTCCACAACATTTGTAT
27	tarc0408	CoreSet	1A	38.7	CCCCTGAGATGGTTGCGA	TATCAGTCTGCATCAGCCAGTTC
28	tarc1594	Other	1A	48.6	CCAAACAAATTTTATGCCACAATATAG	GCAAGATCTGAAACCAATCTAACAAT
29	tarc1603	Other	1A	49.2	CTGAGCGCCCTGATCCAA	ACTTGTTGTACTTACTGTGAGATAAAAC
30	tarc1593	CoreSet	1A	49.3	CATAAATATGCTCCAAATACAAAAACC	GCTACGTTATGTTTCTTTCTGCTCC
31	tarc1611	Other	1A	49.5	GACACTCAGGTCATTCTTCATTTCA	GCCCACTGTCTGACGCCG
32	tarc0421	Other	1A	62.6	TTTACAACGCTACTCCAGTACATGAC	GGGAGAAGAGGGACAAGCAAC
33	tarc0426	Other	1A	72.8	TGGTCAACCATATTTCTCTGATGATA	ACCGAAAGTAAGTCTCTCATCTCGT
34	tarc0423	Other	1A	76.5	TTCTTTACTGAGACATTACACATTATTT	GTAGACTAGATCTTCTGGAACACTATTA
35	tarc1591	Other	1A	76.5	TTTGAGTGAGAAATTATAGTTAGAAAAT	TCATACCAAACTTAGCGTCTTCG
36	tarc1606	Other	1A	91.4	TCATCAAAGCATACAAATACAGCATC	TTCCTGTGATAGTGTGATTGTGTTG
37	tarc0414	CoreSet	1A	91.5	CAAACAAAATAAAGAAGTGCAAACC	GCATTTTGACTTCTGAAGCTGTAGT
38	tarc1602	Other	1A	94.2	TTAACCGATTGATGTTGCAAAAC	AAACAATCATCAAATAATTAACCAAATA
39	tarc1610	CoreSet	1A	95.8	CAAAACAAAACAGCATAAAGATCTG	GCAGAAGTTTACTTGACTGAAAAGAGTT
40	tarc1596	Other	1A	95.9	CCAAGACACAGAGATGCAATCG	GCAGGGAGGAGACAGGAAAG
41	tarc2524	Other	1A	95.9	ATCAATTGAGCAGTCTTAAAAATACAAT	CAGCCGCTCTTGAAGGTTC
42	tarc0409	Other	1A	111.1	GTAAAGTTCTTGAGCTCTCTGAACTATT	TAGTTTTCCCAGTTATAAAGAGGAGC
43	tarc1608	CoreSet	1A	123.6	TACTGCTTACTATTCAATTAACATAACG	GGAGAAGAAGGCAAAGGCAA
44	snp8238	Other	1A	132.0	GAGGCATTGGTGAAACAATCAC	CGAAAGAGCCGCTGAAAGAAC
45	snp1803	CoreSet	1A	185.1	TACCAGCAGCAGGGGTCGG	GAAAACGGTCACCCGTATACTGC
46	snp8116	Other	1A	223.9	CTTCACGACGTTATCCAAATTGTT	TGACCAATCTGCGAGGCGA
47	tarc1577	Other	1A	250.2	GCCTTATCTTAGGACTGAAATATGCTA	AGTAAAACTAAGATTTGTATGACTTTCG
48	tarc0400	CoreSet	1A	251.0	TGTATAGGCCACTGCACTTTTGTA	GCAAGATTACCTCGTCGTACAGC
49	tarc1560	Other	1A	254.4	GTTAGTTCAAACAAGGCCCAAA	CCCTGCTTCTCTTCTATGAAAATGA
50	tarc1567	CoreSet	1A	260.8	GACTTGGAAGAATCGATGAAGAAAC	GCCGCCTAACCGCTGACT
51	tarc2523	Other	1A	268.9	TATCTGTGGAGACAACAAAAGGAGA	GAAATTGTGGCTACCAGAAGCA
52	tarc2517	CoreSet	1A	270.0	CCCTCATTGCATAACTATGTCTCG	TACAAACTAGCTTAGATTGTTACACCG
53	tarc1582	CoreSet	1A	288.5	CCTCGTGAGAATCAGTCGATACAA	TTGTGGTATGTAATAAACATTCTAAAGC
54	tarc0436	Other	1A	298.6	GCTTGTTACTTATAGTTGCCTAGCTATT	TGAATGGAGGGGAGTAGTAAACAA
55	tarc1588	Other	1A	301.1	TCAAGCCTTTATATTTTTAATATGATAA	AGCCAACTACTGTATAGAACATGAGACT
56	tarc1580	CoreSet	1A	301.8	GGAAAAGAGTGGAGAGCTGAAGTC	GCAAAAATGAAGGGGTAATACAAGA
57	tarc1549	Other	1A	308.8	TCAAATACTGCAAGTAGACATACTTTAA	CCAACGAACTTCTTTTCCACAT
58	tarc0411	Other	1A	313.9	AGTGTCTTCAGATAATTGGCGACT	GTCTTCTGTGTCTTCGATAGACTTCA
59	tarc0433	CoreSet	1A	320.9	ATGTTAAAGGTTTAGAGAAGGAAGCA	GCATATGAAGAAGTTAACGGCA
60	tarc1554	Other	1A	333.9	AATTTACAACGCGGGGCTT	TTGCTATTTGTTTCCAAAGAGACAT
61	snp3882	Other	1A	337.7	GCACCATTACATCAAGAATCGAT	GCGCTTTAGCGAGCTCGAC
62	snp3883	Other	1A	337.7	ACTCTTTTTTATTCAATGGGACTCAT	GACATTTGCCGCTGTGGAA
63	tarc1587	CoreSet	1A	343.7	GTTCTCCAAATCACCACAGCC	AGGAACGACTTCTCTGAAGAATCA
64	tarc1555	Other	1A	345.8	GATCAATGACTAGAGTTGATTTTTTCG	GCTATAATGGCACCATCACCG
65	tarc2518	CoreSet	1A	350.0	AATGATCTACTCCTGCAATTTCATAA	GAGTCGTTTGAAAATTCAATTTCTTAA
66	tarc0448	Other	1A	350.8	AGTTGCTTTCTCATAACCTGTTTTGT	AGTGAAATATCCTATGAAATGGGATTA
67	snp1806	Other	1A	352.1	TCAACTCCGTGTCCTGATTCTCT	GACCCAGCTGATTTCCTCGA
68	tarc0401	Other	1A	352.9	AATAAAGGATGATAGGAATCAACAGAG	GCTTTTCAAGTGTACCATAACTTGCT
69	snp2762	Other	1A	353.3	ATTACCATGCTGAGATTTAGTAATCTCTA	CATCAGGAAAATAAATTGTGATCG
70	tarc1566	Other	1A	353.3	AAAGATGGAGGTGAGTGGATGTT	CTATGATCTCTTCCAAAAGCAGGTA

Supplemental Table 4. List of amplicon sequencing primer sets for wheat.

No.	Name	Type	Chr	Pos[Mb]	F_primer	R_primer
71	tarc2515	CoreSet	1A	353.3	AGAGGCTTCATCTGCACCCA	TGATCACTTGCCATGCTAAAATT
72	tarc0452	Other	1A	353.6	CCTTTTTTATGGACAATTTTCTAACATT	GGCTGAAACCTGAATGGTACG
73	tarc0434	Other	1A	354.9	CAGCTTGTGATGAGTTTTTTTTTC	CCTCCCTTTTTACTTGTCTCGC
74	tarc1557	Other	1A	354.9	GCCGTAAGTCATACCCGCTC	TTCCAATCTTCTTGACCTGATAACA
75	tarc1562	Other	1A	355.0	GGGAGAGGGCGATGGACC	CATTGATGATCTGAAAACCAAGAGT
76	tarc0429	Other	1A	368.2	TGGAGCGGGTGATTTCAAAA	ACTTGTTCCCAACAGCGGA
77	tarc0441	CoreSet	1A	368.4	GTTTTTCCTTTTCTCTTGGCG	TAAATTTACTACTGTATTGTTTGGTCTT
78	snp3665	Other	1A	368.8	GAAGAACCAAAAGAGATATACGAATG	AGAATGGGAACCAGTGGAACTAC
79	tarc2522	Other	1A	368.8	GAAGGTGAACTGGAAAGAACCG	TGAACTTTAAAATTTAATTGCTAGAACG
80	tarc0397	Other	1A	369.5	ATTAGGAGTAGATGAGTTTGCTTCTTAC	CTTGGGCCAGTAGGAGCG
81	tarc1561	CoreSet	1A	372.6	GTACTAGAACACACACAATGCGG	GTGGGCCATCTATGGAGGTATAC
82	tarc0447	Other	1A	376.8	CACTTTACACGGGCTAATCAGG	TCTCTTTTTTTTATGATAGATGTGCAAG
83	tarc1564	CoreSet	1A	377.0	TGCAAGCATGGGCAACCG	AATTTGTACACTGACGAATAAATAAACA
84	tarc0420	Other	1A	381.3	ATTGACCTTGTAGATATTCGACCTGT	TACCTCCTTCCGTTCAATAAAGC
85	tarc1581	CoreSet	1A	381.3	CTGAAATCTTTCCAGCCCCT	ATTTGTGGTATTCTCCACTGCCT
86	tarc1584	Other	1A	381.3	TGTAACGAACACCACCCAGGA	AGGAACGAAAATTCTTGCTAACC
87	tarc0430	Other	1A	385.7	GTAGATCGATTTGGGTGCGG	GTGTAAGCAAGCCATATTTGTTTTT
88	tarc1568	CoreSet	1A	394.6	GGCTTCTGTGCTAATGAATGCT	TTTTGAAGCTGACATATTCTTTAGTAAA
89	tarc0431	Other	1A	396.4	TGAGTATTTGCCCCTATTTTTGC	GGCTTCCTTCTACAATATTAATGTAAAG
90	tarc0402	Other	1A	397.7	AAGGCAAAAGAAGGATACTGAGC	ACTGCAGGGAATCTTGTGCC
91	tarc0437	CoreSet	1A	397.8	AAGGAGCGGAGTGAGGCAG	CAGAGTCCGAAAAGTATCTTCCG
92	tarc0417	Other	1A	431.4	GAGAGGCATAAGCATGTGGGT	AGGTTGGGCAGCAGGTTTG
93	tarc0405	CoreSet	1A	440.8	AACATAACATATTGTAGCTCATCCAGTA	CACACATTTCCTGCTCCGAA
94	tarc0403	Other	1A	442.6	CCAGGGTTGAATCAATAGCTTCA	CCAATTTAGCTTTTTTGAGAATAGTCA
95	tarc0451	Other	1A	448.9	GGTCGTTACTTAACACTTGTATAATTTT	CATACTGTGGGTGAGACAGGAGC
96	tarc0435	Other	1A	458.6	CAAGGATCACAATGAGAGCATAGG	GATGAAAAAGTTTACCTTACTGACAACT
97	tarc0398	Other	1A	461.8	CATGTTCGGAAAATTGAACAGATT	GGCTTGGCCCAAGGATTG
98	snp7021	Other	1A	462.4	ACATAGGCAGCATGTATAGCCATA	TTTGCTACTTGATTTTTCAACCG
99	tarc1586	CoreSet	1A	462.7	AATATGCTTATTCTCTTGGTTGCG	CCTTGCTGTATGCTCACTTTGC
100	tarc0442	Other	1A	463.8	GCCTGCTGGTCCCAATCTAT	GCAGCTATCGAAAATATGTTAGTATGT
101	tarc0444	CoreSet	1A	474.2	TGGATTAATACCGGCAAGTCG	TGAGGACTCAGAGTCGGACTACG
102	tarc1553	CoreSet	1A	474.2	CCATTCTCATGCAAATGCAAGT	ACGGATTTTCTCGGGGCTA
103	tarc1559	Other	1A	480.5	CCTCTAGGTAATGAACTGGCCTCT	CTTTGTTCAAGATTTCTCACACCA
104	tarc1551	CoreSet	1A	480.8	ATTGCTTTGTAGTTCATCGAACAAT	GGAAATCTGTTAGCAGCTGGAAATA
105	tarc1571	Other	1A	481.9	AGTAAGTTCAAACTCTCAATTTTTTTAT	CGGAGCTCAAGAATGTACTCCG
106	tarc1570	CoreSet	1A	482.3	GGCGAGGAGAAGCAGTTTG	CAGCAGTGGGCTCGTTGATTA
107	snp2584	CoreSet	1A	491.1	CGATGGGAGTAATATACTGCCTGT	TGATCTGAGTGTCGGGTCAATC
108	tarc0440	Other	1A	491.1	CGAGGAGCCTGGAATATCTCA	CATGCCAGGGTACCCTTAGATG
109	tarc0432	Other	1A	497.5	AAAGTAATAATTGTTGTTTGCTGATGA	AGAAGACACCAAAGGGGGC
110	tarc1556	Other	1A	497.9	GATATTTCATTGCTGCCATACTCAT	TTCATTTTGTGGAACATGGCTTA
111	tarc0419	Other	1A	508.6	CAAGCTGTTGGTTGGTGGTG	AGAGACAACAGATACACTCCCAGC
112	snp601	CoreSet	1A	516.8	CCCCTCTGGTTAATATGTCCTTCT	CGGTATCCTCGCCTACTTGTAATA
113	tarc0439	Other	1A	517.4	CAAGCCATGACATGCGAGC	CATGAAAAATATACAAGAAAAATAAAGA
114	tarc1576	Other	1A	517.4	ATAAGAAGGGAATGCACTGGACT	AGCGTTCCTGAAGATTCAGATG
115	tarc0450	Other	1A	518.7	GCTAGTAAGCACAAAATTTGAACAGAA	TTATGAAGTCTCAAGGGACAACAGA
116	tarc0424	Other	1A	519.6	CCCATAATGCACTTTGATCTTCTC	TCCCTCAAGGTATTGAGTACACCA
117	tarc1589	Other	1A	519.6	TGGGTGCTGATAGAAATATGTGGT	ACAATATATTTCTTGAATTCTGTATTGT
118	snp6624	CoreSet	1A	528.7	GTAGGATATTGGTCTGTTGTGAAATATT	GGTTTTCATGGTTTAGTGAATGAAC
119	tarc0418	Other	1A	530.2	GCTGCATCTCCACCTTCACC	GGTACAGCCCAAATACCCCAT
120	snp4080	Other	1A	531.7	GTGGTTGTAAGTATAGAAAGGCTGG	GCTGTAACATAATATACTTTTCAACCA
121	tarc0422	Other	1A	531.7	GTCCGCAGGTTGGTCTTTTAA	GCATTGGATGAACAGAGAAAGATTA
122	snp6934	CoreSet	1A	532.9	CCAGGGAAACATAGAGGGCG	AAATACATCATTGCTATGGCTCGTA
123	tarc1583	CoreSet	1A	533.5	CAGGTAAAATACGCCATTTCACT	TCTGTTTGTTCTTGAGCAAATTACTTAT
124	tarc0404	Other	1A	534.3	AAATAGGCTTATCTTTTACTAATGAAGC	CCAATACTTGATTGCACATAATCTGTC
125	tarc0425	Other	1A	535.1	CTCAGGGAAGCTGTCAGCG	GTCCTGTCCACCCACTCCC
126	tarc2519	Other	1A	535.3	GTAGTACTAGCAGTGATCTAACCAGGTG	CGTCGCTGTCAGCTTCAGTC
127	tarc2514	Other	1A	537.0	TCCAATTTGAATATACCCTCCTCTAT	TAGATATACTTCTTTTGAATCAAGCCC
128	tarc1575	Other	1A	537.5	TTCTGCTTAGCGATATTACGTAGGT	TGGCAGTTTTGCGTCTTCAA
129	tarc2520	Other	1A	537.5	TACTCTGAACCAGGTGTAAGGGAA	CACATCTTGTAACCAGGATGACG
130	tarc0415	Other	1A	540.1	ATTCACCGATGATTACTGGTATGG	ATTTTTTGCACCAAGAGTCCG
131	tarc2509	Other	1A	542.0	GCAACCCTCCTGGCAGTG	CGCCGTCGATCATCCGTG
132	tarc2513	Other	1A	544.0	CGGAACAGACATGGTAACATGTG	ACGGGTTGTTCTTCTTGTTCTCTA
133	snp1195	Other	1A	544.1	CTCTAAATTCGTCTCATCTCCACAG	CGATCCAGTCATCATAAGGTAAACA
134	tarc1590	Other	1A	544.2	AGTTTATGCAAGAGGCAGGCA	GACTTTTACATGGATAATGAGTACAGGA
135	tarc2521	CoreSet	1A	544.4	CATGATGGATAGTCCGATTCTGG	CCCGAGACTCGTCGCGTC
136	tarc1572	Other	1A	544.6	CAAGTCCTGGCACACATTTGATA	GGTGCTGGAAATGAGAAAAAGA
137	tarc1585	Other	1A	544.6	GAAAGGTTGCATGTGCGCTAC	ATTCAGGATCACCATAACAGTCCA
138	tarc2510	Other	1A	547.3	CTTGCAATAATAAGGGGGAAAAG	AACTGTATTTATGTGTTTGCTAGGTAGT
139	tarc1565	Other	1A	549.4	ACACAAACTCAGTTCTTGAATCAGC	GAGATCCTCATCGAGGAGTCGA
140	snp2784	CoreSet	1A	549.8	TGAACGAATTAACATGAAGTCCATATA	TGGAACAGTCTGGTGCCTCC

Supplemental Table 4. List of amplicon sequencing primer sets for wheat.

No.	Name	Type	Chr	Pos[Mb]	F_primer	R_primer
141	tarc1573	Other	1A	551.3	GAATGCAGTGTGAGCATGTGTC	AGCTACACAATATAAACAATACAACTTT
142	tarc2512	Other	1A	551.5	AGACATGGGTCGTAAAGCTGTCT	GGAATGAAACCAATTTGTGAAACTAC
143	tarc1552	Other	1A	554.3	CTAAGTCTCTTCTTTTTTTGTACAGTTC	AGTGGCTGGACGTGGAGTTC
144	tarc1558	CoreSet	1A	554.3	GCATCACTTGTATTTGCGGATAGT	TCGTGATCGCCTGACTGGTA
145	tarc1563	Other	1A	557.0	GCATAAAATTCTCTTATCAGCTCAGATT	CACCAGTTCAAGGTGAGCAATG
146	tarc2516	Other	1A	557.5	CTGAGTCTGGCCTTGATATTGTG	CTCGGAAGCAGAAGGCTCAA
147	tarc2511	Other	1A	560.5	CTTCAAAAGCTCAGGAAACATTATG	AAAGATACAAAATGCACATCATCAGT
148	tarc1579	Other	1A	573.2	AAGCTCTCGGTTAGCCAGTAGC	AATCAATTTAAGCAGAGAGTTTGGAT
149	tarc0446	CoreSet	1A	578.5	ATGATGGCTTCATCAAGGTCAA	CTATAGCAAATGATGCTGGCG
150	tarc0413	CoreSet	1A	580.2	ATCTAGTGGTTCTTGTGGTGCG	CGATGTGATTGTTGAGTCTGGTAT
151	tarc1569	Other	1A	580.2	ACTAAATAGTACTCAGAGATCATGGTTT	CTAATCTTGGTCTATATCAGCTTTATAC
152	tarc1550	Other	1A	580.5	CAAATTTCTGACAGACAAACAACCT	ACTAACCTGCAAGACCCAGCAT
153	snp1560	Other	1A	581.6	AATGTTTTTGATCCTTGCGTGT	AAGTGCTGTACTGCCATGTGACT
154	snp2035	CoreSet	1A	581.8	TCCACCAAACTGTCAGGCATAG	GCCCGCCTTAAAAAGATCATTAC
155	tarc1574	Other	1A	581.8	GCATTTGGGAGGTACAATTCTAAA	GCCCGCCTTAAAAAGATCATTAC
156	snp6312	Other	1A	582.4	CAAAGTTACTCAACATCATGAGAAAGA	AATGAAACCAAGTCTGAAAAGGAC
157	tarc0406	CoreSet	1A	588.3	GTGAGCCTACTGGCATTTGTTC	CCGAAGAAGCTGTTTTCCCTT
158	snp4271	Other	1A	589.1	TTCTATCATGGCTTCCAGGGA	CTTGTTGATGATGTGCCTTCCTA
159	snp1306	Other	1A	589.4	TGGGAGGCGAGACAGAGTCTC	CCCAGTAATACAACACTGCACAAGA
160	tarc0416	CoreSet	1A	591.2	CGATTCTCAAGTATCAACAGTCACAAA	ACTTATTGCCTTGCATATAAATAACTGA
161	snp3977	Other	1A	592.3	CTACGGTGTGAACTAACCGCTG	AGTGACAGTGAAAAGTGCAGAGAATAT
162	tarc1578	Other	1A	592.3	GGAAGGTATGCTTCATTCTCTCGT	TGTTTCTGACGTTCAGACGACAG
163	tarc0816	Other	1B	1.4	ATGCCAGGACCTCCAACCT	TCTGCCCTCGGACTGATGAA
164	tarc2013	Other	1B	3.7	GGAAGAAACCCTCATAGATGTTCG	CGCCGAGCCATGACGATG
165	tarc2010	CoreSet	1B	4.3	CGGCCAGTCTGCTGATGC	CCAGGAAAATGGCACTTGG
166	tarc2011	Other	1B	4.3	CTCAATCCTGTAGACTCCATCATACC	TTTCCTCAATACATCAGCATTTCA
167	tarc2001	Other	1B	5.2	ACAAAAAGGAGAAATTGGTCATATTC	CAGTCCAAAGTTACAAACAAGGTCT
168	snp61	Other	1B	9.1	TGTATCGAAACCTTGCACAAATT	CTTATGTGCTTTACATAAAATTGCTATTT
169	tarc2005	Other	1B	9.1	GGATATATTCTCTCTTTGTCAAGCCC	CTTTTCAATCTGTTCCACATAAGTTG
170	tarc2008	CoreSet	1B	9.1	GCTTAGCAAGCATGTAGAGTCTAGG	CGTGGAGCTGTCTTAATTTAGTTAGT
171	tarc0827	CoreSet	1B	10.1	CATAAGAGGAGATCTACTAACTGTCATA	CATTCAGGGACTACCATGCTCTG
172	tarc0812	Other	1B	15.1	AGCTGTGCCGTTTGAAGACA	ACAGGTGGCGATTAGTTGACATT
173	tarc0817	Other	1B	15.2	GATTAAACCAAGCAGATATCAGGG	GTCAAACAGAGTTAACATTGTGGGT
174	tarc2002	CoreSet	1B	17.8	CTGGACCTCTCAGAAGGCTTGT	GGACACAGCTCTTACTGCAACG
175	tarc0844	Other	1B	22.0	CAAGCACAGCCCTTCCCTAT	AAGACTGAACAGATAAAGTTCGTAAAAA
176	tarc0843	CoreSet	1B	26.2	GCAACAAAGAGTTCTGTGTCCG	TTATTCTGCTCTCTTTTACTCTGAGTAT
177	tarc2006	CoreSet	1B	40.2	CATTTCGTATGCATAGTTCTAGCTAATG	GTACTGTTTGGGGTGTTTCGC
178	tarc2007	Other	1B	41.1	CACATACGCTCTGCATTATTTCG	ACTTGCAGGAATAGACGACGC
179	tarc0834	CoreSet	1B	42.3	ATAGAAAAGGTAAAGTTTGGGATGAC	TTGGCGTCTCCGCTACACA
180	tarc0839	CoreSet	1B	45.7	GAGTTCTTTTGGTTAGGCTGGA	ACGAAAGCAGAGCTTACAGAAATAGT
181	tarc0823	Other	1B	52.9	TGAAGACACAGAAGGAGAGAGTGTTA	GCGTGCTCGGTGCTGCTAT
182	tarc2009	Other	1B	58.6	AGCAAGAGCAAATCGCAACA	GTTTTAGCAACATGTATGAACCTTTACT
183	snp2578	Other	1B	59.6	ACGTACTCGGTAGGAGTACTACAAAAA	GATGCCACCGACTAGGTGTC
184	tarc2004	CoreSet	1B	59.8	GCGCTAGATAATCAGTAGTAGCTATTGT	CGCCTCTGTCTGCTCTGCTT
185	tarc2012	Other	1B	61.9	GCTTCATTTGTCTAGCCCACC	ACTGCTCTCGCCGCCTGC
186	tarc2016	Other	1B	63.2	GGACCAAGTGTATTTCACAACGA	ATCTTGGGTAATAACAGTTTGCCT
187	tarc2000	Other	1B	65.5	GCTAAGAAATTCACCAAGAGGCTT	ATTAAAGATTAAACGTGCTTGCGT
188	tarc0822	CoreSet	1B	68.1	GCACATGCACAAGTGTTGACAT	CTTTTTCTTGCCAATCTTGCG
189	tarc0847	Other	1B	99.6	TGATGTTCTAGATAGCGAGCCAA	TTTCTTGATTGTTTTTCCTTGGG
190	tarc0815	CoreSet	1B	118.5	GGGAGAAAAGAGAAACTATCACAAAGA	TTAATAAGTTATGTACACCAATGCCAGT
191	tarc0850	Other	1B	132.1	CCATTAACAGCATCAGGCACTG	GCAAAGTTGAATTGCTAATACGATAC
192	tarc2003	CoreSet	1B	151.5	CTGCCCCAGGTTGCTTCC	TGGCATACATAAGCAGTGAACGA
193	tarc2014	CoreSet	1B	156.6	AAATTTAGAGATGCAGTACAGGGTGT	GGAGGTGGTCGTTTTCTCAGG
194	tarc1999	CoreSet	1B	199.6	CCACCATCAGGTCCACCAT	GTATCACTAAGAAAAAGGCTCTTGAGA
195	tarc2015	CoreSet	1B	217.3	AAGCGGCGTTAAGAGGGC	GTTCTGGAACTTATTGGTTTGATG
196	tarc0809	CoreSet	1B	299.4	ATTACCATTAAAAGAACCGACAGC	AGGCATCGAGATGCTTGGG
197	tarc0810	Other	1B	300.6	ATTAGGGAGTGTGGACAATGTGAA	TGTGTTGGTGTATTATGCAACTGTG
198	tarc1986	CoreSet	1B	329.0	CAGATCAAAATGCACCATGCC	GTCATGAGGAATTGAGAACTGCA
199	tarc1974	Other	1B	332.1	GCTGGGTTTTGGGGTGTC	CATCCAAGGGTTGGCTTGTT
200	tarc0845	CoreSet	1B	378.4	TACCATCTGATTTGTTAGGAGGTCA	CCAGAGAAAGAGACGAATAACACG
201	tarc0811	CoreSet	1B	380.9	CAGCAAAAACCCGCAACAT	ACCGTTTCCATTCCTCATGTATT
202	tarc0841	CoreSet	1B	385.2	TGATCTTTTCAGTTCAAATAATATCCG	GAAAAGCAAAGAATTGATGGTGTT
203	tarc0819	Other	1B	411.8	CTATATTTTGCACAGGCCACGA	GATGTCAACTCAATGAGCCCG
204	tarc0826	CoreSet	1B	415.1	CTGGGAGATAATGGGTGTATTCTC	AGATCATGAGATGCTGTAAATGAACA
205	tarc0838	Other	1B	428.1	CATAACAGTGATTGTTGTTAGAAAAAAG	TGTGTGCTGGGCTTCCGT
206	tarc0821	CoreSet	1B	444.8	CAAACAACGAGCGTGTCTATCCT	GGCGAAGATTTCTCGTGTGA
207	tarc0818	Other	1B	449.7	AAAGAGAGGCTGACAATCCCG	AAGCAATCCAAACAATAAAAAAAAAC
208	tarc0824	CoreSet	1B	453.2	TCAACTCTCAGAAGCACATCATCAT	GCAAAGATATTATTATCACATCTCCAA
209	tarc1960	Other	1B	468.5	CCAGAGTCATTCTTAAAGCGACATC	GAACATATATCTCATCCGCCTAGAATA
210	tarc0836	CoreSet	1B	488.2	TGCTGCTGCTGCTGCTGC	GGAGTCGCTAGATCGCTTTAGAT

Supplemental Table 4. List of amplicon sequencing primer sets for wheat.

No.	Name	Type	Chr	Pos[Mb]	F_primer	R_primer
211	tarc1979	Other	1B	492.8	CCCCTTTGTAAACAACCTTCCC	CAAGAGGTTATGAAATCTGTAATATGTT
212	tarc1998	CoreSet	1B	496.1	CGGAACCAAAAGCCCATAGTG	GTGCAGGTCTTTTGAGAACAATG
213	snp270	CoreSet	1B	510.8	GGAGGTGACAGGGTACATACAGAT	ACCAGAAGGAGGTAGTTTTCATCTC
214	tarc0808	Other	1B	518.3	TGATAAACTGTGGATTTATTGGATGA	TCCTTCATCAGAGGTATTATTCTGTTAG
215	tarc1970	Other	1B	531.2	GCAGCAGCAGTAACAACAACAAG	GCGAGCGAGCGAGCGATA
216	tarc0849	Other	1B	532.2	CATCGCTCCATTGTCTTTCTTTAT	GCAAGGATAAAGAGAAGCCCAT
217	tarc1971	CoreSet	1B	544.1	CATGATATCAACCAGCTTAAAAGG	GAATCGCCTGCTGAAGGTAAGT
218	tarc1973	Other	1B	545.8	CCTCAAGCCCTTGCTCTCC	CAGCTCATGTTTCTAGTAAAAGAGGTG
219	tarc0848	Other	1B	551.2	GCACAGATGTTAGTGGAATTCACG	CCCTGTCATATCAGCCGCC
220	tarc1961	Other	1B	551.9	AGCAAAAGAGATTGAAGAATCAGC	GGTTCAAGCTCAGTCCTTACTTTG
221	tarc1982	Other	1B	553.0	CAGCCCATATATTTTGATTTCTGACT	GATTGGAGCGTGCCCATCT
222	snp3076	CoreSet	1B	565.7	CAAATTATGATTTCACAGTTCCTGC	AATAGGGTTGTCCTCCTGATACATAT
223	tarc0832	Other	1B	569.0	CTGTGGCCTTCCGCTTTG	AGGTGGAGTGCTTCAATGGTC
224	tarc1990	Other	1B	572.5	TGTGGTCATCGCTATGTGCCT	CAGGACAGCATATAACCAGAAACG
225	tarc1993	CoreSet	1B	573.4	CAGATCAATGTCATGTTCACTTACTG	TTTGTAGTCTCACAAACTTGACTATACA
226	tarc1965	Other	1B	575.9	TGAACTGTGAATCTTGTACATCCTTT	TTAAAAAGATCCGCCAACCAA
227	tarc0829	Other	1B	583.1	CAAACAGCATAACTAGAAAAGGCAT	CTTATGTCAAAATTGTAAGTTCCTTCA
228	tarc1964	CoreSet	1B	583.1	TGAGTTTGCACGGAAATTAATACA	ATGACTAGGGATACCAATCCATACC
229	tarc1980	Other	1B	583.4	TCTAAACCTGTCGTACATTGGGG	TGGAAGTAACTGAATGTCTGAAACTGTA
230	tarc1987	Other	1B	583.7	TACTTGATGATGACGATGATGACG	AGAAGACAGGGCAACCAAGTAGT
231	tarc1975	Other	1B	585.9	AACCCGGAGGTGAGAAGTTTC	TGTTGAAGCTAGGCTCCAGAGA
232	tarc1972	Other	1B	586.3	TCAAAGCCAAGGGGAGCATA	CCCAGTCCATTATTTTAAATCCTCTC
233	tarc1988	Other	1B	591.2	TGTGCATTTTATGTTTATCTGCTATGT	AAAACAAAGCATCTAACTCCAAGGT
234	tarc1995	Other	1B	614.3	AAATTATGTATGTATCCACATGATGTCT	CGACCTTTCGACAGACAAACAA
235	tarc0813	CoreSet	1B	614.5	AAGTTTGTTTTGTCCTAGCCATTC	GCATTCAGGGCAGTATAGGCT
236	tarc1978	CoreSet	1B	614.5	GGCAGTATAGGCTGGTGCTCT	AAGTTTGTTTTGTCCTAGCCATTC
237	tarc1966	Other	1B	615.1	CAGCACCATGTTTTTTATGATAAGTAG	TGATTTAATGTTGTTTGTACTGGCAG
238	tarc1967	Other	1B	615.1	CTGCAGCGAGCAGTGCCT	CGTCCATAAGCCCCTCCAATA
239	tarc1968	Other	1B	615.1	CTCTGTTTTGGCAATTGATGC	CGCAGCCAAAAGATTCATATTGT
240	snp4154	CoreSet	1B	627.9	CTGGGTCCTTATCAAACTTAACATATT	CTATCTGGTTTCAAGATTACAACACG
241	tarc1969	Other	1B	628.0	AACAGCTCTTATCTCAACCAGCAT	CGTCCCAAAATAAGAGTCTCAAAA
242	snp5915	Other	1B	630.8	TGTGCTCCATATTGTGTTGTTGG	GCGTTGCATAATGATTATGTACGT
243	tarc1992	Other	1B	630.8	TGTAGCTGCTTCTTATGGTTTGC	GGGGCTCGTTCATTTGGC
244	tarc1994	CoreSet	1B	631.2	TTTTCTTTGCGGGGAACACTAT	GCTAAAGCTGGAATGTCTTTCTCAG
245	tarc0835	CoreSet	1B	637.8	CGTCAGAAAAACAAAACAGAAGG	GGATCAACTAGGGGGTGTGCT
246	tarc1976	Other	1B	637.8	TTCATGTTATTGATTTCCATAACGC	TCGCTCTCAACTCTATCCCACA
247	tarc0837	Other	1B	642.4	ACACCAACAAGCCATCATAAAAGTA	TTAGGGCAACTACGGGCG
248	tarc1962	Other	1B	642.5	GGGTCAAAAGACTGCAATTAGGT	GCCCATCATGTAACCCTTCAAG
249	tarc1984	Other	1B	643.1	AAGGACTGCTTAAATGAAACCATG	CTAGATTCGTGAGTTTGAAGAAGAGA
250	tarc0828	CoreSet	1B	643.6	GCTTTGTTGACAGCGTCTCCTC	CCTTTGCCAAATAGTTTTGTTCG
251	tarc1997	Other	1B	658.5	TTACTTGTTCTGTTTCTCTCATACAAAC	GGATCCATTCCTCCAAAAATAATCT
252	tarc0833	CoreSet	1B	658.9	TAGCTTGTGCATTTCCTTCTTGA	CCCTGATAAACCAAATCGTCG
253	tarc0840	Other	1B	661.5	CGCTGGCGCAGACTAATACA	TGGCACCACACGTTCAGAGAT
254	snp7992	CoreSet	1B	664.8	GCAAACTTGAAGATATCATGGGG	CTTCCTCAACTCACAGTGAGCTC
255	snp8332	Other	1B	664.8	GAAAGGTAACGAGTGCCTCCAC	GAACAACTCAAAAAACCCTCCAA
256	tarc0830	Other	1B	664.8	AGCTGATGGTCCAGATATAGTCGT	CCACCTATGGAGCCAAACAATAA
257	tarc1963	Other	1B	667.2	AGTTTGTTAAGTTAACATGAGCAGTCC	CCGTGCTTTTGCAATCTGAAG
258	tarc1985	Other	1B	668.1	ATTGGCTTTGATCTGAAATAATCG	TTATTACGGGAGTTGTAAACATAGAGG
259	tarc1996	Other	1B	668.8	GAGAACGGGCAAGTGGCAT	ATGCTTACCTACACATATGATATTTTCC
260	tarc0825	CoreSet	1B	670.4	TGAATGTGGAGATTAACTGTGTGC	ACTGGTAACTGTTATGTATGGGGC
261	tarc1977	Other	1B	674.5	TCTATGCCATCTTGCAATGTAAGTAT	AGGCAGAGGTTCGGGCTAG
262	tarc1989	Other	1B	675.6	GGATCACATCGTTGGATAGCAA	CTGATACTATTTCCAAGCTCCTGTAGA
263	tarc1983	CoreSet	1B	676.1	CAAATCACTTAAGGTAAATGACGG	CAGCATGGACAGCCAGCA
264	tarc1991	Other	1B	676.2	CCAGTCAACGATATCATGTGAAGA	AGGTGTGCCTGATTATACTAATTGTTAT
265	tarc0820	Other	1B	680.9	TGGCTCATTATACTGTGCCTTCAT	CGTAGAGTTAGCATGACATTAATTTGTT
266	tarc0814	Other	1B	683.6	TTTTTGGCTGACCAATACACATAA	TTTTTTTGTTCAGCTACGCTGTTAC
267	tarc0842	Other	1B	686.8	TGATTCATCAGTCGGTTGGTTAG	GCCTGTCGATGATTGTGTGCT
268	tarc0846	CoreSet	1B	686.8	AAGTAAAACCTAATTTATCGTTACAGAA	CGTCGTGTTCCATGAGTTGAGT
269	tarc1981	CoreSet	1B	686.8	CCCCTGCCCCCTGTAAGT	CCACCTGCACGCACACGG
270	snp6512	Other	1B	687.4	AATTTGCTTCTTTTTTATGTCCATTC	GGACAAGCATTAGGCATAGGTTA
271	snp198	Other	1B	687.7	TGGACTTCTGAAAGACGTCTGC	TTCGATCCCAACTTGTGCTTC
272	tarc0831	CoreSet	1B	687.7	CGAAGAACTCTTCACCGTTGC	GCAATCAACTGACAGTTCATTCTAATC
273	tarc1364	Other	1D	0.6	TATGTTGGTTGTGTGCATCCTAAC	ATATCAAACATGGGATTGGGAATA
274	Inf22056	Other	1D	0.6	ATCCAGCGCCAACTGGGG	TGGTGCTTATCCTATGGATCAGC
275	Inf15585	CoreSet	1D	0.9	GGCGAACATCTATGAGGGTTAC	ATGAGGGTTTCGGACACGC
276	tarc1268	CoreSet	1D	2.1	TCTATTTCTTCCGGTACTAATATGATGT	CTCAATGTCCTGGATGGGC
277	Inf43259	Other	1D	2.1	GAATCAGTGGGTCGAGAAGAATAG	CTTAGCGGATATTGACCTAACGA
278	Inf44021	Other	1D	5.5	GCCCCGGGTGAATGAAGT	GAAACCAAGGCGATTCTGTCT
279	tarc1372	CoreSet	1D	6.3	ATCGGCGTATCCTATGATTGTG	ATGAAGAAAGGCATTTCCCACT
280	tarc1283	CoreSet	1D	6.8	GCCGCTAAGCCATCCCTC	TCGGGGGAGAAGGGGGAC

Supplemental Table 4. List of amplicon sequencing primer sets for wheat.

No.	Name	Type	Chr	Pos[Mb]	F_primer	R_primer
281	tarc0083	CoreSet	1D	7.9	GAAGACCCTGGTGGTAACAACAT	CAGATATATTACATAAATGGACCATCTTA
282	tarc2358	Other	1D	7.9	ATAGTGATGATGACTTGTTTGGGG	AAGAGTTAACGGCACAACACAATAA
283	tarc2360	Other	1D	7.9	TTATCTGTATCAAGAATTAACTGTTGGA	TGTATCATGGAGTGATGCACTTATCT
284	tarc2365	Other	1D	7.9	GCTCAACGAGATCGCTCGG	GCCATCTCCAGCAGCAGC
285	tarc2366	Other	1D	7.9	CGCGCAATCTAATTCTACCATGA	GCAAGGGTATGTATGACGATGATA
286	snp7797	Other	1D	8.6	ATCCCCAAACAGCTTGTGTC	CCCTTGTTGCTTGGATTTGT
287	Inf28957	Other	1D	8.6	CCGGAAGGTGCCCACACC	ACTTGGAGTAGCAGGAATCACAGA
288	tarc1297	Other	1D	9.8	CCAATTACTCTTTGTATCTGTAGGCG	ATGTATGATTCAAGTTTTGTGGGAA
289	tarc0076	Other	1D	9.9	AATTGAGTGCAAACTCGCCAT	AAATTACAGTAAAGTGAAAGTCACCAAT
290	tarc0066	Other	1D	10.2	CTTCACCATCTCCATCTTCTATCG	AAGCCTACAAAATAATAAATCAAACACA
291	snp5372	Other	1D	10.3	ATAATGTCCTAAGGTAGCGGATGTT	TTCAGAAAATCAAGGTAAAAAGGAAC
292	tarc0070	Other	1D	10.4	CGATGACGATGACGACGATG	CATTCACCAGACCAAAAAACAAC
293	tarc0072	Other	1D	10.4	CAGGTGCTCTGCTCCAGCTT	CACACCAGATTATTTTATTCAAAGCAT
294	tarc1271	Other	1D	10.4	GCATTGTTTTGTGGATTATTCATCT	CTCCAGACCGAAAAAGCAGAA
295	snp4716	CoreSet	1D	10.7	CTTGTGGATTACATATGATACTCCCA	CAGCTCAAACACCGTCATGTAGTA
296	snp7533	Other	1D	10.7	GAATATCTTATCAAATACGCTAGAGTCTAAC	GTAAGGTTAGATGGGGAAGATAACAAC
297	tarc0065	Other	1D	10.7	TGGTATCTGGGGATTTGACTTTG	CTTAAATGGGGTGCCTGCAC
298	tarc0067	CoreSet	1D	10.7	GGACATAAATTAAAAATTAGTTTGTTGC	CCAGGTCTCCAATCTTTACTTCAC
299	tarc0069	Other	1D	10.7	TCATAAACATCAGCTTTGGCG	GTGATGAGGACCTTGTTGGGA
300	tarc0082	Other	1D	10.8	ACCAGCTTAGATGTTGCAGCA	ATATGCTTTGAGGTTCATAATGTTATT
301	tarc0075	Other	1D	11.5	CACATGTATCATCAAATGACTCAAGTT	TGTCGCATGATCCTTCTCTGC
302	tarc0081	Other	1D	12.3	GATATGGGACCATTATGATAACAGG	GGATAAAAGACTAGAAACCCAGTGGT
303	tarc2357	CoreSet	1D	12.3	GGAGAAAATTCCCCACCAAAA	CGTGATTTCGAGCGAGCAGT
304	tarc1274	Other	1D	14.5	GGGATTGGTCGGTCCCTATAT	CCTTGCTAAATTCTGTCTACTCGG
305	tarc1453	Other	1D	14.7	GCCGTCTTCTCACCGCCG	GTACATCCTCAAGGACGCCG
306	tarc1328	Other	1D	15.3	ACGCGTGAAAAATACTGCCC	ATCAACTGATTCTCCCTCTCTCTCTA
307	tarc2364	Other	1D	17.0	CCATGCAAATTGCTTACGATGT	TAATAAAATTGTTGGAGAGTGAAAGTTT
308	snp8551	Other	1D	18.1	CAGAAAGCTTCGTCTATGATGCC	GATGTAGGATCTGCGTCAGCG
309	tarc2361	Other	1D	18.1	GTTGGCACCTCAACGAGTGA	GACAATGATGATACGAGACCAAAAG
310	tarc2362	Other	1D	18.1	GGTACATTTGCTTGCCCCC	CCTTTAAAAATGGAGTCAAAACCAG
311	tarc1429	Other	1D	18.4	TTTGGACAAGCCAATCAACG	TCATGTCCATCCCACTTCCC
312	tarc1383	Other	1D	18.9	CCCTGTAACCAAACACCCTCAT	ATGCACGCAGGGTCAACTAA
313	tarc0071	Other	1D	19.3	CCTGAGTTATGTTATACCTAGCTGATCTAT	CCAATGACCAGTCCTTCAAAGAA
314	tarc1316	Other	1D	19.9	ATGGTTATTTTCCCCACATCG	TTTCTAGTACAATATGGTAAGGCTTACG
315	Inf44231	Other	1D	20.1	TGGTTTTCTTTCTTTGGAACGG	TTTTGAAAGTCCAGCCTTACAAAG
316	tarc0064	Other	1D	21.5	TGCAAGCTATTGCGCTTCAG	AGGGACGAGGAGTTCAACCC
317	tarc1423	CoreSet	1D	25.7	CGTCGCCCAAAACACTACATT	GGCTTTAGCCCCTTCAATGG
318	tarc1321	Other	1D	26.0	CGTGGAAGTGCTCCTGCAA	GAAAAAAAGTACGATTATGTGGCTG
319	Inf12311	Other	1D	27.7	CGGTGACGGCGAACAGAC	TCTCCTCTCCCCTTGAAGCAT
320	tarc1280	Other	1D	27.9	GGCCGGAGGAAGAACCTC	CTGCGGGAGGGAGCGAG
321	tarc2363	CoreSet	1D	27.9	CCTCTATCATAATATAAGACGTTTTTTG	GCTTCAGTTTGTGATTGATTTTTTAC
322	tarc0077	Other	1D	28.2	TCCCTGAATCCCTGCGTG	TTGAGAAGATGCTTCTTGACCG
323	tarc1469	Other	1D	29.5	ATGATGCTGGTGGAAATGGAA	ATTAAAAAAGAAAAGAAAAAGAAAGACA
324	tarc0068	Other	1D	34.6	ATGATTTTCGTGATTGTAGAAACG	GTAAACACATAAATAACAGCAGTCCA
325	tarc1494	Other	1D	52.7	ATCTTCTGGATATAGCATTATTTGTGTA	ATGCAATGAAACCTAGTAACCGA
326	Inf36154	Other	1D	55.7	GGGCTAACGAAAACGCGT	ACGATCACGCAGGATGCTG
327	tarc1490	Other	1D	58.5	CCTCCGTTCATCAACGTAAAAG	TCTCTGAATATTTGACAAAGCTAACACT
328	tarc2359	Other	1D	74.4	CAGAAAACACTGATGTATATAACACACT	TAGCCTTTGTAATTTCTTGTTAGAATTA
329	tarc0078	CoreSet	1D	86.9	CGAACACAGGGACAGTACAACTG	CCGAGTAAATTAGGGACTCACTCG
330	tarc0080	Other	1D	93.1	GACACAATTCTTGACGGCGT	ACACGAGGCTTGCCATCG
331	tarc0073	Other	1D	98.1	CAGAAACAATCATCAAAGGTAAAAGAA	AGTTTTTGTCGTGTCAAGTCGTTT
332	tarc0084	Other	1D	98.8	CGGACCTGAGGCGAACAG	GCAGCCGTGGATGTAATCAA
333	tarc0079	CoreSet	1D	120.5	AAAATGGAGACAGATGCCGC	TGAATGGCGAACCACTGGT
334	tarc0058	Other	1D	176.4	AACACTATGCCTTCAGAATGTGGT	ATCTCCCGAGTTAACATGGTATGTA
335	tarc2343	Other	1D	185.1	CGACAAAGTAATACTGCTTCCTTTTTA	TTGTATCACGACAGATCCAAAGATC
336	tarc1365	Other	1D	198.3	ACCATCAACTTCAGCTATCTCCTCTA	TTATATTTTTTCTCTGCATCCTTTTTA
337	tarc0037	Other	1D	200.9	TCACTGTTTTGTATGCGATGCTC	GTGTGGACATTGTTTGTACTTCTCAC
338	tarc0035	Other	1D	202.6	TTGATTGTGGACTCTACAAGAGGTG	GGGTTTGTAAGTCCCTACCAGTG
339	tarc2342	Other	1D	204.6	TGTCTTCTGTCACCAGGTTTTGTA	AAAGGGATATAGTGGACTGCTGTGT
340	tarc1472	Other	1D	221.1	ATAGATGATTAATCGGGTTTTGGAT	CATTGGTATTCTTGCGCTGGT
341	tarc2349	Other	1D	227.1	TCTTAACATTCCGGTTATTCTTTACAG	GCTTAGGTATAGCCTGACTCGATG
342	tarc0034	Other	1D	230.7	GTATCCAATTTGGACAAAAGAAGTG	TCTGAAACTTAAAGAAGCAGGATCAT
343	tarc2354	Other	1D	230.7	GTAGTAGTTAGAAAAAAAGACATGGACA	CACATCAGCACCACATTATACAGAGA
344	tarc0060	CoreSet	1D	253.2	ATTGGTGAAGATTCTTCAGGCTAG	TCTAAACATGAACAGGCGTGACT
345	tarc0043	CoreSet	1D	280.2	ACTTTGCACCAACCCAAACTACT	TCTATACTGACCTGATCACCATCC
346	tarc2351	CoreSet	1D	307.2	TGGCAATACTGTCAGTTGAGCC	TTGTGTAATGGACATTATAAACACTCTC
347	snp3481	CoreSet	1D	320.4	GTCAGGATGAATTTTCAGTGACAA	CTAGCATCTGGAGATAGACAGCGA
348	Inf61426	Other	1D	326.0	AAGAAGCTCATTCGACATTGCTT	GAGGATTGTGATGAGACTGGGG
349	tarc1298	CoreSet	1D	328.1	AAGAGCACCTCTGGCGGAA	GAATGTGTCAGACTGGCTGACG
350	tarc2355	Other	1D	330.6	TTGATGCTACTGGCTTAAGACTTCT	CAGTTCAATTGTTAAAGATTGTTTTTT

Supplemental Table 4. List of amplicon sequencing primer sets for wheat.

No.	Name	Type	Chr	Pos[Mb]	F_primer	R_primer
351	tarc2352	Other	1D	345.7	CCTTCAACAGATTTTCAATTAAGGTAA	GAAATTATATGCATGAATTTTAACTTCT
352	snp7154	CoreSet	1D	351.4	GTATTACTCTTAGGCTTTGGTATGTCA	GAAATCAAGGCTTCGTGCG
353	tarc1366	Other	1D	356.9	TTCTTCAGCATATGGGGATACATAA	AAACGTCCAACAAAGTTGAACTACT
354	tarc2356	CoreSet	1D	359.8	ATGTGTGCTTTCTACAGTAATATATACG	CAGTTAAGCTTAGTATTATTTTTCTCTT
355	tarc1392	CoreSet	1D	370.6	ATGTATTTTTAATTTAATTCAGATGAGC	GAGCTTATTTACTGACAAGGAGGG
356	tarc0049	Other	1D	372.9	AGAATTTTCGTAACTGACACTCTAATTTA	CAACACACATTTCAGTCAGATGCTA
357	tarc2338	Other	1D	385.0	GGAAGGGCAGGTACGTTACG	CGCCGTCCATGTGCTCGT
358	tarc0050	CoreSet	1D	386.1	GCGCTTTAATGTCTTGATGTAACA	GCAGCCAGCGTCAGTATCTG
359	tarc0042	Other	1D	387.2	AAGTTCTGGGCAAGTCCTATAAGTG	CGGCTCATTCAGTAGCCCCT
360	tarc0048	CoreSet	1D	391.3	GTCCGAGTACATCTACCAGCGA	TTGTAGGATATACATGTTGGCGTT
361	tarc0040	CoreSet	1D	402.3	CTCTTCTGTTATTAAGCTGCTTCCT	ATTTCGGTTAAAAAATTAACCTAGC
362	tarc0038	Other	1D	403.7	CGTATAATATTTTGGCAACATGGTACT	CCTTTGTTCCACCAATGCCTT
363	tarc2344	Other	1D	405.8	CACGGAGATTGCGTGCTTG	TCGGTCTGGGGTCCAGTTAG
364	tarc0052	Other	1D	409.8	AGTACATCTGTACATGTTAGGGATTTAAA	GCTGGCTTTGGAGTTTGGA
365	tarc1399	Other	1D	412.2	ATTCCTAGCAGAGTGTTTACGCA	CTTAACCGCTATGACCTACGTATG
366	tarc2353	Other	1D	413.2	GGCAACAACCTATTGAGGTAAGATT	TATATAGATTCCAGATGCTACTACTGTG
367	snp6146	Other	1D	416.8	GTTTTAGCGATTGCATGCGAT	GCCAGATGAATCAACTAACGACC
368	tarc1477	CoreSet	1D	420.7	CATGAGGCCGTCCACTTTTT	CCGCCGCCTCTCTCTTGT
369	Inf23980	Other	1D	423.3	TTAGTCATCATCCTGATCTGATATCGT	CAACCAGAATTAGAAGACCAAGAAGT
370	tarc1357	CoreSet	1D	423.6	ATGCTGACTACATTTGTAGGTAAATTAT	CATTTTTTAATGCCAAATGCAATT
371	tarc0056	CoreSet	1D	431.8	GCTGATCAATCCATAACAAATTCAT	AGATATTCAGTGTTTCACCAAGACC
372	tarc1545	Other	1D	432.0	GTCTCCATAACACAGAGACAAACAGA	CCAAATTACATTACAGAGATGACCAA
373	tarc0045	Other	1D	435.3	TCATGCTAGTAATGTGCGAGTTATTAG	CTGTGGGGCGATTTGCTC
374	tarc0046	CoreSet	1D	435.3	GGTAATCATGTCGTTATCTACCGA	TTGGCAAGTGTAATTTGGATGTT
375	tarc0055	CoreSet	1D	435.7	AATCGAGAATGTGCCACAACTG	TGGACTCGATATGATTAAGGGCT
376	snp3547	CoreSet	1D	436.2	GGCAGTTGGAGACGTTGCTAC	TTTTCGGATCGGGCTCTTG
377	tarc0033	Other	1D	439.0	ATTCCACACCAGATATAATCTGTACG	CCAGCAAGTACGGTTCTTCCTC
378	tarc0044	Other	1D	451.1	CTCATTTGGCACAAGCTCGA	TCTACAAAGTCGGCTTGGTGTT
379	tarc0063	Other	1D	455.8	CAGCGAAACTATGGTAGGTCACTC	AGCCTGAAAATCAAATCGCA
380	tarc2350	CoreSet	1D	455.8	GCGAAACTATGGTAGGTCACTCC	TCATACACAAAACAGTGAAATTATCATT
381	tarc0059	CoreSet	1D	458.3	CAAAACAGGAAGTAAACTCAGTAGACA	TAAAACTTGTTCACTGGAACCACA
382	tarc0036	Other	1D	458.9	AACGCACACACAATATGCTCATTA	CTTTGGACACCCTCCTTTAGGT
383	tarc0057	Other	1D	459.0	GCTGACAAAAGCAACCGTTAAG	GCAATACTTACCATTGTCTACTTATCG
384	tarc1440	Other	1D	459.9	ATGGATTATGAGTCCAAGGACTAGTTA	GAGTAATCAAGCTCCAACACAAAA
385	tarc2341	Other	1D	462.5	CGAGGGTAAGCCCTCTCTTTT	GGAGTACTGATTCTTGTGTTTACTTGTA
386	tarc0061	Other	1D	467.5	TCTTCAGGTAACCTCATTTGTGC	AACTTCGAATCAAAGCCCTACACA
387	tarc0041	Other	1D	467.9	GATTTCTCGTGATCTGAACAGATTATAT	AGTGATGTACCAGTGCTAAAAGAGAC
388	tarc0047	CoreSet	1D	467.9	ATAACATAAACAAGGTTACCAAATCC	ACAAGAAATTCGGGAGGCTG
389	tarc1498	Other	1D	472.5	TCTGCAAGAGGCAAAAGAGTGA	TTGACGCCGAGGTGGTGA
390	tarc0062	CoreSet	1D	473.3	GACAGGTTACATCTTGTGAACTCG	GATTATATTGAACACCTGAAGGGTC
391	tarc0039	Other	1D	473.6	AATGAGAAAACAGGATCACTGATCA	AGTTTAAATGATACTCTGCTGGGC
392	tarc0031	Other	1D	473.9	ATATGATAAAACCCGGAGAAATTG	ATCCATAGTACTAACTGCTTCAGAAATT
393	tarc0032	CoreSet	1D	476.4	GGTAAAAATGTGCTGACCTCTACA	ATTTATCCGTGCATGATTCATCT
394	tarc1418	Other	1D	476.9	GCAATTTGAAATATGGTATGTTAGGTAG	TGATAGGCAAAGACGTGGTGG
395	tarc0051	CoreSet	1D	480.3	GGGAGGACTGGATTGCTTTGT	CAAGTGGCAGCTTCCTGTGAT
396	tarc1314	Other	1D	480.9	AATTTGATATGTGCTCTGAAGTTTGT	GTTTAACTGTTCTTTAGAAGGGGGT
397	tarc2346	Other	1D	484.4	GTAAGATGTTGCATACTGTAATAGTTGA	CCTTAATTATGCTAGTACATGCTTTGTC
398	tarc0053	CoreSet	1D	484.6	CAGGAAGTCCTTCCCTTCTATCA	CATCGAGGAAACGCAGGC
399	tarc2348	CoreSet	1D	485.5	TGTTGCTCTGACTTTCCTCGG	GTGTACAATCAACTATGGCTTGACA
400	Inf25377	Other	1D	485.8	GCACCTGTGGTATAAAAGCCAGT	CTTCCACCGAATGTGTCATCAG
401	tarc2339	Other	1D	486.0	CTGTGGGTACCATATACTCTACTAGTCT	CCTCGAAGAGCTTGTTCACGA
402	Inf17607	Other	1D	486.1	CCTGCTTTTCTAATGCACATCTTTAT	CGAGCATACGGTGCGAAAC
403	Inf46801	Other	1D	488.0	AGAAAGATATTGAACTCTAAGCATTTGT	GCTTGAGGTGAGAAAACCCG
404	Inf16510	Other	1D	488.6	CAGGTGGGTATATCTTCATGCG	AAGTGGACCGGTTTATCAACAAT
405	tarc2347	Other	1D	489.7	AAATCCCGTGCATGTGCATAT	GGTAATGATGTGAATTTCTGTGGTG
406	tarc0054	Other	1D	491.6	GACAGCAGGCTAAATAAATACTCAAG	GAAGGTAACTAACTCTGCTCCTCTGT
407	tarc2340	Other	1D	493.8	GCGGAAGTCGAGGCTGTTCT	GTCGAATTTCAAGTAGAAGAAGAAAGA
408	Inf6300	Other	1D	494.1	ATCAAATCAGGGACCCCGA	CCACCCCTGGAACTGGAGT
409	tarc1388	CoreSet	1D	494.2	CGTCTGTAACTTCCCGACGTT	TGATATATAGCATAATGCTTAATTTTCA
410	tarc2345	Other	1D	494.5	GTTTGAGGGGCCTAGTTTTGAC	CTGCGGTAAGCGGGATATG
411	tarc0465	Other	2A	0.3	TTCAGTTTTCTTAATGTATATACTTCGA	TGCACTACTGATTTCACAATTTGC
412	tarc0461	CoreSet	2A	2.5	GAGGTAAATTATGCTTGTGCATTG	GCATCTTTAAATGAGAAGCCATATAAT
413	snp2428	CoreSet	2A	2.6	CGGTGTTGGATTTTCACCTG	CCTTGCACTTTTCCCTCGC
414	tarc1664	Other	2A	3.8	TATCTGCCAGCAGGACTAAGAAGT	AGTATGACAGGCCAAGTAGAGGTG
415	snp1562	Other	2A	4.2	CTGACCATGCCTGTTTCTGTGT	TGTAGTAGCGCCCACTCGC
416	tarc0453	Other	2A	4.3	CAGAAACCCACATTTTGCAGTCT	CAAGATAGTGTACTGCCGGACAG
417	snp6922	CoreSet	2A	4.8	GACCAAGAGCAGATTCTACATTTACA	ATACCAAACTTGATTTCATGATGTG
418	tarc1686	Other	2A	4.8	GCTTTAGAATATTTTATTTTCACTTATG	CAAACCGATTCTAGCCAGCATT
419	snp5762	Other	2A	29.9	GCTGCTTGCACAAAGGCTG	GAAAGCTACTCCAGTAACAAAACTTACA
420	snp1242	CoreSet	2A	30.0	GCAGTGCCAAATAAAGAACCTTATT	CTATGTATGAAGGTCTTTTGTGAACG

Supplemental Table 4. List of amplicon sequencing primer sets for wheat.

No.	Name	Type	Chr	Pos[Mb]	F_primer	R_primer
421	snp4441	CoreSet	2A	30.5	CAAATTGCGGTGCGGTTT	CCCATTCAGTGCTTGCTGC
422	tarc0475	Other	2A	30.5	AAAAAAATATAAAGATCAATAAGCAAAA	GCCATGTGGATTTCTAATTGGTTA
423	tarc0485	Other	2A	31.1	ACACAATGAAAAATATGAAAATACAATA	GAAAGTGCACATGTCTAGGGTCC
424	snp7410	Other	2A	32.1	CCAACCAGAACTAACATTGCAA	GTGCCAGTCAAGAGTTACCCTAAC
425	snp5087	Other	2A	33.0	TTTCCATGATTACACCAGATCACA	CACCGTCGACGACTCTATGG
426	tarc0464	CoreSet	2A	33.0	ATCATCTTAAGTAAAGTTTCGCCAA	GAGATTCAGTTTGCCCCCG
427	tarc0497	Other	2A	35.5	TTATGCAGTTTTACCATGTTAGCG	GGTAGTGGAGACAGACACCAATAAA
428	snp4212	Other	2A	41.2	GGGACAGCTAAGTTTGTTGCAAG	AGATGCGAGGAACCTACAGACC
429	snp4213	CoreSet	2A	41.2	CTGTACCATTTTGAACGTCATCTTTAT	GGTACAATTTGAATCATAAAATGCAT
430	snp4215	Other	2A	41.2	GAACTTTTGTTTCTTCTCTTGGGT	AAGATAAGGGAATAAATCCATCAGTT
431	snp6477	Other	2A	45.1	GGCGGGTACTATCATTCCTTG	TTAAGAAACTATCTGTTATCAGATGTCCA
432	tarc0493	Other	2A	48.9	CCTCATGTAGTGTTTGCCCCT	AATAGAGGAACCAGATCCTTTATACG
433	tarc1685	Other	2A	48.9	AAGAGCCTGCACACGGTGTAG	GGTCGTCTATTCAGTGGTAAATTGTC
434	snp6250	CoreSet	2A	51.6	AAAATATACTACAAACGGGCGC	GGCCAAATGAGCACCCAA
435	snp6566	Other	2A	61.3	GATAAGAAGCTTTATCTTTCTGGTAATATC	GCAACAAGGATCAGCTTTCCTC
436	tarc2541	Other	2A	61.5	TCCAGCCAGCATGAAAAAAAG	AAAGTTTGTGTCCTATTTGCTCAAC
437	tarc2549	Other	2A	61.8	AAATGGCATCTACGGTGGCT	CCGCGTCTGCACAGCATA
438	tarc0478	Other	2A	62.3	ACCCTAATTATCTTTGTTTAATCTCTAC	AACAGAAGTATTTGTGCAGGCG
439	tarc0473	Other	2A	67.5	TTCTAGTTCTAGACTAGAAGCGATTTTA	CAAAGAAGACCGCAAAGTTGG
440	tarc0502	CoreSet	2A	69.2	GTGAGGATGAGCCATTTTCATCT	CTTCTAATCCAAAACCCATTTTAATT
441	tarc0496	Other	2A	69.4	GCTACCTCCGTCTCCCCA	GCTACCACAATAATACTACATACACAGG
442	tarc2540	Other	2A	69.4	CCATTCTACATGAACGGCAAAT	GATGGCGAGAAGAAGAAAAAAA
443	snp5022	CoreSet	2A	71.0	ACAGTTCATTTGACACTGATCACAC	CTGCAAAAACAACTTCATTCCAA
444	tarc1675	Other	2A	71.6	TCTTTGGTATGTCATTTTTTTGTACTTA	AGGAGACATTTACTAGATTAATTTTTGG
445	tarc2538	Other	2A	73.2	CCAGTATTACACTCTAATTTTTTAGATG	AAATGCTGCTGAAGAATGAAGTTTA
446	tarc2539	Other	2A	73.2	AGAAGGATAGTGAATTAATAACAGTAGG	GCTATAAATTGGGATTGATTTGACA
447	tarc1665	Other	2A	73.5	TTTGGTTCAGGTGCAAGGC	ACCTTTCCTCAGTTCATTCCATTA
448	tarc1666	Other	2A	73.5	AGACTACGTACAAATCAGTTCACATTAC	AGCTCAATTATTCTTGTCTGACAATTAT
449	tarc1689	CoreSet	2A	74.1	CTTTGAGAGTTTGAGCAATCGGT	GCAAGAAGGTCAAACGCACA
450	tarc1657	Other	2A	74.8	TTAGCTAAAAATGAATATGCTACATCAG	TTTTAGTTATCAACACAGAGCTCCTTTA
451	tarc1678	Other	2A	74.9	GGCCGTGTGGATACAACCG	GTGCTGTAGACATTACCAATTGTTTG
452	tarc1683	Other	2A	75.1	GGCTGAACGGTTGCTGATG	CTGTTCATTTGTGCAAAGAAGTTTC
453	tarc1662	CoreSet	2A	75.3	GATACTGTATGTTGATCTTCCACTTCA	TCATGCAATATTAGCACATAAATAGCT
454	tarc2537	Other	2A	75.3	AAATATATCAGTTCTCTATGATCACAGA	GTTTCCATGATACAGTCGAAATCTAT
455	tarc2546	Other	2A	77.3	AAGGCTCATTTTTCATGGAAGATA	TTCATTGAGAACTGCAGTCAGTCA
456	tarc0483	Other	2A	77.9	TGTATTACAGATTACTCGGTTAGGGG	TCGACAGGAAGAAGGGAACG
457	tarc0486	Other	2A	78.8	CATAATTTCCATCTTTTAAACTTGATCT	AAAAACTGAAAGTATGATAACTGCTGAC
458	tarc0482	Other	2A	99.2	ATGTTCCCTCCGACATCTTTGT	GGGGTAGTATTTTCTTCAACAACATC
459	tarc1674	CoreSet	2A	101.9	GCCGCCAAACTCTCCTGA	TGTAGTGTCTCTGGTTGAACTGTGC
460	tarc0488	Other	2A	102.7	ACAGAACTATAAAGTTGTTTATGAAAGT	TATTATAAAGGTGAGAGTCAGAATCCAT
461	tarc1659	Other	2A	104.9	AAGCAAATTGTGTTATGTACTTAAACCT	AGCACTTTCTCTGCATCCCG
462	tarc0491	Other	2A	117.4	GCAGGAAAGTCTGCCCGAA	TGATTTGTTTTTTCTTCCTTTTTTCT
463	tarc2544	CoreSet	2A	119.1	TCAGTGCCGCTTCGTCAAC	AAAGAATGTATAGGTCCTGGCATTTA
464	tarc2543	CoreSet	2A	126.6	GCCATCATTACCTTTTAGTTTTTTTTAA	AAAACACAGTGCATAAAGTTCCATG
465	tarc0498	Other	2A	134.0	GGTACTGCACTGCCTGTTTTTTG	GCATTTTTTTAGTGTCTCGGGA
466	tarc0456	CoreSet	2A	141.3	TCATTTTTCTAGATACCAAATTTAAAGC	TTTTCCCTGCCGTGATTTTT
467	tarc0468	Other	2A	141.3	CCAATGAGACCACAAAATAACCG	TAAATAAGGTTGATTTTTGTAGAGTTGT
468	tarc0501	Other	2A	141.5	AACTTTCAGCTTTGAATTGGAACA	AGACCTGAGGAGGAACGTATGC
469	tarc0455	Other	2A	142.7	GCAAGCCCAAAATCAGCAA	CCCCCTTCCGTACAACTTCG
470	tarc0463	Other	2A	145.2	TGTTCTGGAACGCAGTGGTG	TATGAGTGCAAAACAGTTCAGTGC
471	tarc1684	Other	2A	149.2	CAAAGAAATATGCTCCCCATTGA	ACTACTTGTTGATATTTTCCTTTTTCAA
472	tarc2547	Other	2A	151.3	ATTCAAAATTTAAACAAACAAACCTTC	GCTGTTGTTCACCAATCCGA
473	tarc1688	Other	2A	154.4	GTTAATGGCTTCTTAAATGTGCTATAA	ATTCCTCGAACCACCGCC
474	tarc2548	CoreSet	2A	165.9	CTTGGAGAAGCACATGTTCTTTG	GGCCAAGCAGCCTCAATTT
475	tarc1676	Other	2A	179.7	CTTTTTCCGACTCCACTCTCAC	GCTAAGCTAATCCACTGAAACCG
476	tarc1677	Other	2A	179.7	GGCGACAGAGCTTTGATCC	TGCGCAAAAAGAGCTTAGAAGTC
477	tarc2542	CoreSet	2A	181.8	TCAACTGACGATGCTGATCTCG	CCAATGACTGATCATGAACGC
478	tarc2545	Other	2A	182.6	AAGCCCTGCAGTGAAATTCCT	AGATTGACGGCGACGCCG
479	tarc1670	Other	2A	193.7	CATGGACAGAATATCAATTGGGC	ATACAACAAACAGGCAGAGAATCAC
480	tarc1682	CoreSet	2A	205.2	ACAGAATCAGTAGCATGTGCTTCAC	AATGCTTACGTGTTACACTAACCGA
481	tarc1671	Other	2A	205.9	CGTCGGACTACAACAGCTTATTTCT	GGGACTCTGGTTCCCTTCG
482	tarc1690	Other	2A	211.9	ATGACGTGGTTCGAGTTACGC	GCTATTCCGAACAGCTATTCCAA
483	tarc1660	CoreSet	2A	212.6	TGGGTGGTGTACGTTCGATTATT	TGAGATTGACCACGGGCTG
484	tarc1667	Other	2A	214.9	AGAGTCACCAAGATAGGCATCAAA	GCAATACAATGAAATCTGCTTCGA
485	tarc1653	Other	2A	233.5	GCGGAATTCAGCTAAAACAATTT	TGAGGTATAGTTTTTCTCACCCTGTT
486	tarc1656	Other	2A	235.7	ATTATCTATGTAAAAGTCTTGCAGTTTA	CGTTACTGCTGCGAGCATGAG
487	tarc1672	Other	2A	239.4	GGATTAATTGGCATGGGAATATC	GCATTGGGAAGACCAACAAGT
488	tarc1661	CoreSet	2A	240.5	CATGATAGACTGCCAAGAAGGAG	TCTGCAGTTCATGCAATTCTACG
489	tarc1668	Other	2A	242.0	CAACAACTGTAGCTGAAAGCACTG	TTCAAAATTGGCGCATGGA
490	tarc1669	Other	2A	257.9	GGCAAAGGAGAGACTCAATATAGATA	TGGTGAACCATATGAAACAGGC

Supplemental Table 4. List of amplicon sequencing primer sets for wheat.

No.	Name	Type	Chr	Pos[Mb]	F_primer	R_primer
491	tarc1663	Other	2A	267.9	AGCATAAGTTCGATAGTCATTATACCG	TTGATTTGCTGTATGGCAAAATTA
492	tarc1673	CoreSet	2A	271.9	CTTAAGTCTGTACAAGCAGAAAGACAG	CTCATTGTTACCTACAGTACAATTTTAG
493	tarc1654	Other	2A	273.3	GAAGTTTGAGCAAAGCAATCAGAT	GGGATGTTGAACAAGACAGCCT
494	tarc1655	CoreSet	2A	275.9	CTGCTGATCATTTTGCTTACCAT	GAGAACCAAAAGTCAAACAAAGTCT
495	tarc1658	Other	2A	277.1	GCTTTTAATTCCCTACAGTGAACAAGT	CTCACTCACTCTTTGGATTTTGCT
496	tarc1679	Other	2A	277.6	AGAAAACTGCTATTAGCTGAATCACAT	AGAAATTATTGAGGAAAAAGGCG
497	tarc1680	CoreSet	2A	277.6	GGATGTGGAACTGATAATTATAGTTAAG	CTTCCTCAGCCTCTTCAACCA
498	tarc1681	Other	2A	277.6	ACCACGTAATTTTCACATGCTCA	TGCAATGTACCCTTGTTACACACTT
499	tarc1687	CoreSet	2A	285.6	CAAGATATTAATGTATGCGTACCTTTG	CTTTTGCATATTCACATTTACAAATGT
500	tarc1628	CoreSet	2A	341.9	GGTCTTCAGTAAAGGCACCACAC	CTTGGTCCGGCGGTATTTT
501	tarc1630	CoreSet	2A	360.4	GGAGTACTTCATAGCAGGAAGTGTGT	CGTGTGGCGCTATTAAATGGT
502	tarc1638	Other	2A	360.7	GTGTTGTTCTCACAAAAATTAGTCATAC	ACTGGCATATCTGAAACTAAACAACTAA
503	tarc0489	Other	2A	468.5	TGACTAATAAGCATTGAAAAGTATACAG	ACCACTTTTTGCATAAAGGCAA
504	tarc2527	CoreSet	2A	470.2	TGAGCCTCATCAGAAACCTAAAAC	TCTACTGACCAAGTTCTGAAAATAGAGA
505	tarc0467	Other	2A	498.1	GGAAAATGTATGCAAGTTTGGAATT	GGTAGGTAACCACCAAATTCATTCT
506	tarc0474	Other	2A	503.0	CTGCGGTTAAGGAGTAAGTGTTTT	TGGTGCGTGGAGAGAAAAGG
507	tarc0479	CoreSet	2A	504.3	GCCGTTGTTTTCCCTTCAA	CAACTGGCACACAGGAACTCTC
508	tarc1618	Other	2A	507.4	GGGTACACCGGTCCTCTCG	GTAAGCGGCGTTTGGTTGA
509	tarc0471	Other	2A	514.9	TTCTTTGACCATTGCCCAGC	GAAGCACCTTGTGAAGTGAAACATA
510	tarc0481	CoreSet	2A	523.9	CAATCTATTAAAGTCTCAAGTAGCACAA	TCCTTTCATAGTGTTATAAGTGTGTGTG
511	tarc0462	Other	2A	525.6	TGTGCCAACCAAGAGAAGGG	GCACCTATTGCTTGACCTGAAAG
512	tarc1646	Other	2A	530.0	ACAGAAAAGGAAATGAATCAGGC	GCGTTTTTCCTCTCCTTCCC
513	tarc0477	Other	2A	530.7	TGTAGGAACCTTTAAGAACCATCAG	TTAATAGTGCTTAGCATCATATTCTTTA
514	tarc0499	CoreSet	2A	542.7	CAGCTTCAGACAGGTCATCCAG	TTGGTTTCTCGGCGGTGG
515	snp3151	Other	2A	558.5	ATTCGATCCAGCAACTCACGT	TTAGTCAGGAGCTGAACCCTCTT
516	tarc0495	CoreSet	2A	587.4	ATACTGTGTAAAATGGGGTGTGGT	CGTGAAAGATTCGTCATCCG
517	snp488	Other	2A	602.8	GGCTTGACTTGTTCTTTGTTTACG	GGCATCAGTGTACTGTTATTTGTCTATA
518	snp1174	Other	2A	603.5	TAACTGGAACCGCTCGTACAAT	GTGCAATGGTGAAGATGGATGTA
519	tarc0470	CoreSet	2A	612.8	TACAAAATAATCAAAATGTGTGGTCC	TGGCTAAGCTAAAACTTCCCGT
520	tarc1626	Other	2A	638.0	CTCGGACTCCACCGACGC	CTTAATCTCTTCTTGGCTTCTTGC
521	tarc1632	CoreSet	2A	663.3	AGCAGTAGTAACTGCAGCCTGC	CAGCGGTTGCGTGCTACAC
522	tarc2532	Other	2A	663.3	AAGTATAAATTTGTAACCAAAATAAAAG	GCTAAACATGTAAGTTTTCTGGTCACTA
523	tarc1643	Other	2A	669.4	CGCTGAGAGCAGAAGCAGGA	GAGCCGTGTTGTTCGCCG
524	snp7547	CoreSet	2A	675.9	CCGACCCGTAGCCGTGTT	ATGGCGTGAGTGATGGGC
525	tarc2529	Other	2A	677.0	GAACATCTTAAGCTTCCATTTCCA	CTGACTTTGAATGCATAACCTTCC
526	tarc1631	Other	2A	678.2	AAAATGGGGGGAAAGAAGGA	ATGGCAAGTAATTTTACTTGGCAC
527	tarc2531	Other	2A	678.2	TCATTGGCCAGTGCAGGTAATA	CAGGGGCCTGTTTCAGACAA
528	tarc1625	Other	2A	678.4	TTCTGGGATGTGGTTGGCA	AATATGTACCCACATTCTATACATTTGT
529	tarc2536	Other	2A	678.7	TCCTCTCGCTGCTGCTCG	TCCCTGATTTGGCCAGCA
530	tarc1636	Other	2A	679.0	GGTGTCGGCACACAAAGAAAG	GTACAGCTTGTCCTCCATGAATATG
531	tarc2018	Other	2A	681.1	ATCATTTAGTGAGACCTTTTTCTATCG	AGTTAAACGCAAACAAAAAAAAAAG
532	tarc1652	Other	2A	686.0	TAGCATGTACTTGTTTGAGATATGGG	TGACTATTAATGGGGTATTATTATTTCG
533	tarc0476	Other	2A	686.9	AGTCAAAAAAGGGAAATGGGAGT	TCGGGTCACACAGATTTTTTTC
534	tarc1621	Other	2A	686.9	GGAGCCAGCCCACTTGCC	AAAACACAAATATACACTTCCATGAGTT
535	tarc1642	Other	2A	688.6	ATGTGAAAATCCAGAAGGACCTACT	TTAAATTTGCAGAATTATAATCCTATGG
536	tarc2535	Other	2A	688.6	GCCAACAGCTGCCAGCAA	CTAGCACAATCAGAAAATTCATAATCAT
537	tarc2528	Other	2A	689.2	CCTCCCTTGGAGTAGGAATCAA	CAGGAGGGCCAAATGGGT
538	tarc1637	Other	2A	689.9	GTTGAGCATTCAAACGTGACCT	CAGGAAGGGTACTCTCAAGCAAT
539	tarc2530	Other	2A	690.0	CGAATGGAATCATGTTGAAGTAATAAA	CCTGTCGACTCTGCTGCACTAT
540	tarc1647	CoreSet	2A	690.9	TGCAGGGCGTGCACTCCA	AACATAGGGCCTTGCACTCG
541	tarc1648	Other	2A	690.9	GCTCCCAAAACTGGTGCCT	TGAAGAGAAACATCAACACTTCTGAA
542	tarc1649	Other	2A	690.9	TAATCCTCACATCATTGTTCACGA	AGATCTGGGATTCCAGCTCAA
543	snp1275	Other	2A	692.9	GATAGGCAAGTGCTTCTGCTGT	AGTTCCCTTGCTGGTGTATGAA
544	snp3597	CoreSet	2A	694.5	ATCGGTGGAGTGGATTTATCATATAT	GGGATTGTACGGTCTTGGG
545	tarc0457	Other	2A	694.6	ATGGCTCATCTAGCTCAGTGTTTT	AACTCCTGCTATTATAGTTCCACAACT
546	tarc2533	Other	2A	695.0	TGTAAAACGTCTTATATTTTGATACAGA	TATAGAAGGCGTAATTCATATTCCAGT
547	tarc1623	Other	2A	695.2	GTGGTGGTCGTGGTGGTTG	TGCGTTCATCGCTTTCCTTT
548	tarc0469	CoreSet	2A	696.9	ACAAAAAAATGATGGCAACTGAC	GTAGATCATCCACATTTTATGATATTAT
549	tarc1619	Other	2A	696.9	CAGTTGAACCTATATAAACGCCTGA	AACGTCATAAACACGGACCTGA
550	tarc1614	Other	2A	704.8	GCACAAGCTTCAATATACTCAATATTCT	GGTTTGAAGTTCTGCTGTGCATAG
551	tarc1615	Other	2A	704.8	GCATATTGCTCATATTAGTACTCCCTC	ATAGTACTTTGGCTGTTCCATTCG
552	tarc1651	CoreSet	2A	716.2	CAGGTTTGTACTTCTAAAATGAATTTAA	ACATTTACTATGATAAACTTGAAGGGTA
553	tarc1635	Other	2A	716.5	AAGATGGCGCTCGGGATAC	AATCGTGCCCGGAATCGC
554	tarc0466	Other	2A	716.9	GCATCCAAAGAACATAAGAAAAAAC	GGCGTATGTGCTGGGATTT
555	tarc2525	Other	2A	716.9	CATCATAGCAGCCCCTGTTCA	AGGATATAGAGATGGGCTCAACG
556	tarc2526	Other	2A	716.9	ATACACTACTAGGTTTGACATTTTCGC	AATTTCTCTCTCCTGTGAGCTCATAG
557	tarc2534	Other	2A	717.1	CCACAACGTTATGTTCACCCAT	CAGAAAAAACTTCCCAGTGATACAG
558	tarc1645	Other	2A	717.8	ACATTCTGTTGTGCCGCTGTA	CCACTGAAGTCAGAATGTCACAAC
559	tarc0484	Other	2A	718.1	TTCTCAACAAACAAGAATTACTCAGC	GAAGATTCAGAGACGCAAAACAAC
560	tarc1650	Other	2A	718.1	CACGCCCTGCATATATATACACG	AAGATTTAGACTCAACTTTCTTCAGAGA

Supplemental Table 4. List of amplicon sequencing primer sets for wheat.

No.	Name	Type	Chr	Pos[Mb]	F_primer	R_primer
561	tarc0458	Other	2A	718.2	GACTCATTTATTATGCCACTTGCAT	AGGGAGTACATTATTGATGGTTGAA
562	tarc0460	CoreSet	2A	718.2	GTCAGCAAAAGGATTTACGATACAA	ATACAGACTTGAAGCACAACCTTAAGT
563	tarc1613	Other	2A	722.7	CCATGTGTGTGTATTGGCTGC	ATGCAAACACAAGGGATTTAACAT
564	tarc1633	Other	2A	727.2	GGCACTGTATGGTATGTTACATGTGTT	CATAACCTGCTGCTGGCTCC
565	tarc1634	Other	2A	727.2	CACGCCAATACTTCATCTTAATCAA	TTTAACAGCCTTATTAAGTATTTTCTTT
566	tarc0490	CoreSet	2A	729.2	GATTAAAAGTTTAAGCAAAGGGTACTG	GATGCTATAAAAAACAAAATTATATGAT
567	tarc1622	Other	2A	729.3	TATATATAGCGGTTGCTTCCAAATAA	GTATAATAACTACACAGTAACAGCCCCA
568	tarc0480	CoreSet	2A	734.3	CTCAGATTGCACTGAAAACCGA	TGACGATAATAATGATCCGACGA
569	tarc1616	Other	2A	734.9	ATCGATCTCCCTGCTGCGT	CCGGTAAATATCTGTTCCCTTGA
570	tarc1617	CoreSet	2A	734.9	TGCAAATAATTAACTGTACGTATGTACG	CGCTGACCGACAGGCCAC
571	tarc0500	Other	2A	740.4	GCTCTAAAGTAAGTGTTGTTATAGGCAT	TAGAAATGAAAACAGGTGGATGGT
572	snp4336	CoreSet	2A	740.8	GTAACCATGATGATTAGTCATACAGGTC	GTCAAAAGAAGTTGGAGAAGGCT
573	snp6548	Other	2A	741.4	TGCTGTGCTGAAACCATCTTG	TTCCACCGGGATGGTTTTC
574	tarc0454	Other	2A	742.2	CTGAGGGCACATATCATCGC	CCATTTTCATTGACAGGGTCTGT
575	snp7727	Other	2A	743.1	AGAGGCTCTTAGACCTAGGACCA	ACTGACCCCATCGATGTTTTG
576	snp3897	CoreSet	2A	744.4	AGACAATGCTGTCAAGGGGATAC	AGACCATCATAGCTCCCGCA
577	tarc1644	Other	2A	746.6	TGCTGATTATGGTCAGGTATGCT	GTACCTTCAACAATGGCACGG
578	tarc1641	Other	2A	747.0	GGCTGCTACTTGAACAGCATCTA	AAAATTGAGCAACTATGAATAGATAGAA
579	tarc1640	Other	2A	747.6	TTGTGGGAAACTGTCCTCGG	TGAATGTCGTAGAACTTGTGTGCT
580	tarc1627	Other	2A	752.3	GTGTCGTTGCTCCATTCATTGT	CCGCAAGCTCAAGTTATTCACA
581	tarc1629	Other	2A	753.3	CGTACTGAACCTGTATTCCCTGG	TCAACCGATGTAAGATACGCTCA
582	tarc1620	Other	2A	753.6	GAGCAGACAATGAGACAAAAGGTG	AGATGGGGTCGTTGAGAAAGTT
583	tarc1624	CoreSet	2A	756.8	GAGGAAATTCCTCTGAATCCTCTG	GGTTACCTCACGACGTTTCTCTT
584	tarc0472	Other	2A	757.8	AGGAGAAAGGCGGGCACA	TGCTTATATTTATTTGAAATTTTCTTCA
585	snp6797	CoreSet	2A	757.9	CCTACAGGCTGTTATACTGACCG	TGGGGAAAACAAAACACGGA
586	tarc0487	CoreSet	2A	760.0	GGGTGATTGCTTTGACATTTGT	CAAACGGTTGTCTAGCACGCT
587	tarc1639	Other	2A	760.0	TTAGGATCTATTTTTTGGAGGTTTCA	CATGCATAATCCTTTCTCCTGG
588	snp1347	CoreSet	2A	760.6	GTCTTGCAAGGAAAGAGTAGTCTG	TCTAGAACTGAAGGCGCAGACA
589	snp5161	CoreSet	2A	771.2	TGTTCCATTTTGGTTATTATTTAACATAC	TGATAATTACTGGGCCAAGCG
590	tarc0492	Other	2A	773.4	GACTTTGAAAGTAAAGTCTGACCATTT	TATGGCCTTAACTAGATTTTTGTGG
591	tarc0494	CoreSet	2A	778.4	GCGACACTCCGGGTATTTTT	CCACCGACCTCTCCCGTA
592	snp6839	Other	2A	779.7	GCAGATTCCAACCTTCTGCCTA	AAGAACTAGCTCAGGAGCAGAGC
593	tarc0861	CoreSet	2B	4.6	ATGCCATTTTACTCCCTCCGT	TGTTGAATAACTAAACCATTTGCG
594	tarc0905	Other	2B	4.6	GTGCATTTCAAAATTCATTTTCTCT	CATCCTCTTCGTCGTCCGC
595	tarc2075	CoreSet	2B	4.6	GGCGTTGAAATAACAAGACACAT	GGATAGAATACACACAATAGTTGGGC
596	tarc0877	Other	2B	6.3	TAAATATCCAAGGCTGCCAGG	TAACATTTTTTGTAATGCTTTTACTACC
597	tarc0907	Other	2B	6.3	GCGTCTCAGGACCGAAAATAATAC	CGAGCTCACCGCTTCAGAGA
598	tarc2087	Other	2B	7.4	TTCAATTGTTGCAAGTTCAATTAGATA	GATGCTTGAAATACTTTCATGAGGT
599	tarc2078	Other	2B	10.8	ATATCTGGTGTACTGAAATCAAATGGT	AGGTTTGAAGTGTCACTGTTTCG
600	snp4953	CoreSet	2B	11.1	CTCGTCTTTAGACCCTGCCAG	ATTTTCATGATGGATCAGCTCATA
601	tarc0872	Other	2B	11.1	GACATCGATCAAATAAGATATACAATGG	ACCGTTGATGTCTTCTTGTTCTTAG
602	tarc0855	CoreSet	2B	25.0	TTGGCTCAGTGAAAAGAACAGG	ACACATTCTTACCGTATTCTTCTACAGT
603	tarc0860	Other	2B	25.6	TTTGCACATACATAAACTCAAACAGAC	GTGGATTGTGATAATCCCGCT
604	tarc2081	Other	2B	26.6	AGCACTGAGATGACTGATGCTATCT	AGCAAAACAGAGTGCTGCTTGT
605	snp2110	Other	2B	29.0	CATCTCGCTGCTGTGTTGGA	ATCACAGCTGGTACGTCCAATC
606	tarc0890	CoreSet	2B	30.2	TATCAAATAGCAGTTTCACATTTTTTG	GTCCTCAGCTACAATAAACAGCG
607	tarc0898	Other	2B	42.3	GACTACAATAAACTCGCTGTATTCTAGT	CTCACCGATGTTTCTTTCTCCA
608	tarc2094	Other	2B	44.4	CTTCCGTATATTCCTTTCAGGTGA	GGTAGGCATCATACTGATAACAACTTT
609	tarc0864	CoreSet	2B	49.4	CATTACCATAGCAACCATGTATCG	TGATAAACATAAGCAATTTGCCACT
610	tarc0896	CoreSet	2B	56.5	TTTTCGTTCCATTTCTCCCAA	CCCGTCAAGCCAACATCAC
611	snp2572	CoreSet	2B	59.0	GGTAAGCAAAGTCGAGCTAGAGC	AAATCTCACCTACTTGCGGAAAA
612	tarc0884	CoreSet	2B	72.6	CACGCATGTCAAAGGTTCG	GATAACTAAATCCTGTCTTACAAATGAA
613	tarc0876	CoreSet	2B	90.4	GATCGAACAGTGTCTGATGTCTCTTAT	CACAAAATACTTCATGTAGCCCAATA
614	tarc2092	Other	2B	95.8	GAAATTGTTAAGACGAGGCCG	ATTGCCTTTTCACTGTTTCGTG
615	tarc2085	Other	2B	98.0	GTTTTCCTCCTGAGCCTTCTCTA	GTGTTTCCAACTGCATTGACTGT
616	snp4135	Other	2B	103.0	TGAGGCTGATGTGGGGTATGT	TCCATCTACGAGCAGGAGTCTG
617	tarc0909	Other	2B	110.5	TACCGGCACTAATGTTTTCTAAGTC	TGTTATAGCTCATGACATTGAATTTTAC
618	tarc0887	Other	2B	116.5	GCACCACAACCAATCAGTTTCAT	AGCAATAACCCGATTGACGC
619	tarc0868	CoreSet	2B	118.6	GCAGTTTCATGGGGACTTCTTCT	CACCGTTCCATTTAGCGAGC
620	tarc2089	Other	2B	118.6	GATGTGGACTTGGTAGGTCACTTTT	TGTGACAATGAGGTAGGCAATGAT
621	tarc2086	CoreSet	2B	135.1	TGTAAGGGTGGACAGTTAACAAAATAG	GGCAGTTCTTAGCTGTTTGATTATG
622	tarc0917	Other	2B	136.2	AAACATATGGATACCCTAATAATACAGT	ATTGAACTGCCACTTCACCGT
623	tarc2083	Other	2B	146.6	TAGAAAAGGCATCCTTAAGCTGAG	TGAAACTGTTAATTTTGACTCCGAGT
624	tarc2095	Other	2B	148.4	ATAATGCCAAGGTGGTATTTGGT	TCTGATAATCCAGATTCCAGAAGTATC
625	tarc2088	Other	2B	149.0	CCAGATGTAATTGTATAATCATTTTAAT	CTATGTACATGAAAAGGACATAGTATGA
626	tarc2084	Other	2B	149.8	AATTAACTATCAAATGGAACCAAAACA	GAAAAGTAGCGACTCACCTCGTACT
627	snp1763	CoreSet	2B	150.7	CGTGTTGGCTCCTGAAGCTG	CAGTATCTACACACAGCATGACAGG
628	tarc2079	Other	2B	150.7	TACTATCTGCAAAACACAAACACGA	ATCTTAATGTAGTTCCTGAGCTTTACG
629	tarc2080	Other	2B	150.7	CCCAAGTGGGCATAAGGAGT	CAGTATCTACACACAGCATGACAGG
630	tarc0888	Other	2B	151.6	CACAGCTTCATTCACCATTACTACA	GCACTTCCACAGAAGCCCAAC

Supplemental Table 4. List of amplicon sequencing primer sets for wheat.

No.	Name	Type	Chr	Pos[Mb]	F_primer	R_primer
631	tarc0879	Other	2B	151.7	CTACAGGGTATCATTCGCAGGA	TGTCTCTTTTAAATGCTCAATTTTTCT
632	tarc2082	Other	2B	151.7	AACTTCCTCCGCTATTGATAAACA	TTAATCAGTTGCCACAGAATTATATTG
633	tarc2090	CoreSet	2B	153.6	AACATGGAGTTCTCTGCCCAGTA	GTCTGCGATTTGGTAGGTACAAGTA
634	tarc0919	Other	2B	154.3	ACAGTACAGTTCAAGCAGAGACACAA	ATAAACCTTGTGCTCGTCGATAGA
635	tarc0870	Other	2B	159.4	CAGGTGAGTTTCTTGCATTTGGT	GTCGGTAAGGTCTGCGTGTGT
636	tarc2074	Other	2B	159.9	TTTGGAATGAAAAGAGAATCATAACC	AATCATAATATTACCGGAGTATTAAAGT
637	tarc0851	CoreSet	2B	171.8	CAGTTTGCGGACCGAATGTA	CAAGTTCTTCCAATACTAGCACGC
638	tarc0920	Other	2B	172.2	GAACCTAACTCCTAAGAGATATTGTGG	CGAACATAATACACTAAACACTGGATTA
639	tarc0904	CoreSet	2B	191.5	TTTCATATTAAAACATGAAGTAATGGAT	AAATGTCAGACGCACAATTACCA
640	tarc0873	Other	2B	191.9	CAAATGTATTTTTGCAGTTCAATCTG	AAGTGACAGCGTATCGGACAGT
641	tarc2091	Other	2B	207.6	AGAACATTAGGGCGTACCGTAAC	CCCTCCTTCTGTCAATGCAACT
642	tarc0913	Other	2B	210.2	ATAGCGAATTTTTTTACAGCAACA	CTATGGCCGTGAGTTCCTGTC
643	tarc0862	CoreSet	2B	210.3	GAAAGTTAAGAACATTTTCAAAAGAGC	GAGGGAGTACTACATATCATTTATCCG
644	tarc0882	Other	2B	212.2	AGTGTGGAGTCAATAAAATATAAAACAT	AGTTTCTTTAGTGAAAACCAGGCTC
645	tarc0856	Other	2B	225.3	CCAAAGTGCTATTAAGGAGAACGA	GGAATGCCCGTCTCCAAGTAG
646	tarc2077	Other	2B	244.6	CCATGGTGCTTAGCGAGTAGAGT	GGTAACCAAAATCAACCAGTTCAAC
647	tarc2076	CoreSet	2B	250.5	CACTAGGTACAATACAGACATTCCACT	CCACAGGTCGAAACTATGCTCA
648	tarc0891	Other	2B	254.6	CAATAAAGGGAGTACAAACATCGTG	CTATTTTGCGTTTCTCCTGTTGTAT
649	tarc0863	Other	2B	259.8	CATCCTCTGCAACCCTTCTGT	AGGGAAAAGAAGAAGAAGAATGTATTAG
650	tarc0894	Other	2B	260.6	GCAAACCACAACATCCACAAAGT	CAGGATCAAGACGATCTACCCAG
651	tarc0881	CoreSet	2B	260.8	CATGCAACCAGAATTCATAGTTCTATA	CAACTAGACCTTTTCACTCCAGATTG
652	tarc0914	Other	2B	263.8	CAACGGCACAAACATAGAAGCA	GGAAATCTCTTCTGTGACAACGG
653	tarc0875	Other	2B	280.5	TCTTTTTGTGCCTACCGCCT	CCGGAGTCCCGAGTTATATAGTTAT
654	tarc2093	CoreSet	2B	280.5	AAGGTACACACCTTTGTACTTGATGAT	CCCATTGTTAATTTAATGTCAAGATAA
655	tarc0895	Other	2B	373.3	CGCCATGAGATTTGAATCTTCAA	CTGCTATCTTGCAGGAGGACG
656	tarc0903	Other	2B	377.5	TTGAGTCTACTTAATCGGATAACCAGT	GCAGGCACATCAAGCACACTA
657	tarc2561	Other	2B	378.4	GCCCCCACCATCAGCGTT	GAAGGAAGGAAGAGGACTGGC
658	tarc2030	Other	2B	384.0	AGTTAAAAGTCAACAAAAAAGCTAAATA	CTTTGCTATAATAGGAATGCCCG
659	tarc0878	Other	2B	388.1	TCATTCTTTCCCTCCACCGA	GAGGAAAATAAAGTGAATGAAGACTTC
660	tarc2020	Other	2B	390.1	ATGGTACAGGGAAGGAAGTACTAGC	TGAAGTGATATGAGAAGTTGAACGAA
661	tarc0889	Other	2B	392.9	CGTATGCCAGTCGTACATACAAAC	AATCCACAATCTTTGCTTTGATTTAT
662	tarc0867	Other	2B	396.9	GCACAAACGTATAACCTCCCTGA	GCGTATCTTATTGGCTGGAATC
663	tarc2027	CoreSet	2B	397.4	ATTGGTTTGAGTTTTTTCCCTTCT	AGATGGTAACATGCCATTGAGC
664	tarc2052	Other	2B	402.7	GAACTGGAAATTTTAGTATCAATTAACA	GCACTTCATTGCAGACCATCTATATAC
665	tarc2028	CoreSet	2B	411.1	GTTGTGTCTAATAACTGGGTTCGC	CAATTTCGTTGGAATTGTACGG
666	tarc2044	Other	2B	415.1	CGCCGCCAGTGCTGCTAC	CCGCACATCGACCAAGACAG
667	tarc2033	Other	2B	416.8	GCGTCATGGACATGACCAATA	TGATGTTCATAACAGAAAACGCTG
668	tarc2040	Other	2B	417.1	AAGGAGCAGGATTTGCAGGTAT	CAAGAAGCGGTGCAGGCG
669	tarc0918	Other	2B	417.6	TGCTTTCTCTACTTTCCAAGAAGAA	AGGTAAAACATCATATTTTAATGAATAG
670	tarc2070	Other	2B	419.6	CATCAATTCTGTCTCTTCCTGTATGA	CAATAAAATTGGTGACCCAGTGA
671	tarc2037	Other	2B	421.1	TACTCGTCGTCCATTTTCTTGC	GAGAAGCTGCTTACCCCTGTTG
672	tarc2025	Other	2B	421.7	CAAAATGTTTGTAAATGTATGCTGCT	GATGCTTGTAGTTTTGGGATTTGT
673	tarc2062	CoreSet	2B	424.3	TCATCATCTTTTTGGTGGGCA	CATGTAGGGAGAGAAACTCTTCTATTT
674	tarc2034	Other	2B	429.4	CTCGACATTGAAGCCGACAC	CGAGAACTGCAAACATATGACCAA
675	tarc2071	Other	2B	429.5	GTGTATGTCTGTGCAATCACGG	TTTAGGAGATATGCCTCAGAAGATG
676	tarc2038	CoreSet	2B	432.1	TTCCGGGATAGGGTTGTCG	ACCAAGCTGAGATAACGAAATAAGA
677	tarc2059	Other	2B	439.9	GGTGTACAATGGCTTATTCATACG	TCTATCCTTAATTGCAGAAACACAAG
678	tarc0853	Other	2B	441.1	AGCTTCTGAAAGTATGTCGGTTGA	AACGTCTGCATTGACTGGTTCA
679	tarc0858	Other	2B	442.8	CCCACCAACTCAGCCAGATAAA	AGCATCGGTGACTATAGCACAAGTA
680	tarc2047	Other	2B	449.1	GGGGTAACAAGAACAGCTTATAATAGG	GGTGGGCGGCAAGCAGAC
681	tarc2024	Other	2B	451.5	GCATGAACCAACGCTCTAGTAGTAGA	GTTTGTGGTAGATCCCTTGATTTACTA
682	tarc2050	CoreSet	2B	451.5	CCGCAACTTGTCCTGTTTCTC	TATGAGTCACTCGGTAAGTCGGTAG
683	tarc2060	CoreSet	2B	451.8	GAAAGTATGGGTGAAGCCTCG	CATAAATATCAAGACATACATAGCACAG
684	tarc2042	Other	2B	464.4	ACATTTAATCTCGAAGCACCCTAC	GTATGCCCAAGTATGTCTGCGTA
685	tarc2055	CoreSet	2B	478.1	CTTGTGTTATTACAATGGTTGTGTTACT	CATTGCATTCACATAAATTGTTACG
686	tarc2058	Other	2B	478.2	GACCCCATCACCGTCAGG	GCGCTGAAGAAGGCGTTC
687	tarc2026	CoreSet	2B	480.1	GAAGAACTTCAAATAGCCCAAAGAG	CTTCGGCCTCGGCCTTAA
688	tarc2031	Other	2B	482.7	TGCAACAAATTTACATCTTGAACCTA	TTTAGTACAAAGTTTTACTAAAGCTGTG
689	tarc0857	Other	2B	485.9	GGATCGTCAGAATAGCAGTGAATT	AAATATAGGAAGCAATTCTGCGG
690	tarc2036	Other	2B	488.2	TTAATCATAGGAAGTATGTCATACTGAT	GACGCTTGATTTCGGCATTAT
691	tarc0874	CoreSet	2B	524.0	ACTGATCATCTATCTGTACTGCACTCTA	AATCTATCAGCACTGGCTGAAAAA
692	tarc0893	Other	2B	529.3	CCGATACATCATTCTGAGAAGGTATA	CCCGAAAAAAAGGTACTACATTTTAA
693	tarc0912	Other	2B	541.1	TGGACCCTGGCTGACATCTAA	TGCATAATCGCTTCCTCTCTTG
694	tarc2041	CoreSet	2B	541.1	CCATTGGATTATTTATTCCGCA	TACAAAAAAGTAAAGGTAGGAATATGAT
695	tarc2068	Other	2B	545.3	ATGGACAGAAGGCTAATAATCTACTACA	TGGGTGGACCAGGTTCAAAT
696	tarc2023	Other	2B	549.0	TTCAGTGCAGATGCTGACCG	CTATCTGTTCACATCATCGCTCACT
697	tarc0885	Other	2B	551.6	TACTTTACTATGCTTATTCAGGCTATAC	TTGGTTGATACCCTTTAAAAACACTA
698	tarc2048	CoreSet	2B	555.3	CAATAACTGGCAAAAGAGGAGAATC	GTTAGTCCACATTATGTCAAAGAACTTA
699	tarc2049	Other	2B	561.2	CTATGCCTCATCAAAAGAGTAATACG	CTGGCTGATTTCTGAACTCGC
700	tarc2056	Other	2B	563.3	GCTAAAGTCGAGAAGCCCAGTT	CTTTGCAGATTCTGTTACATATAATGC

Supplemental Table 4. List of amplicon sequencing primer sets for wheat.

No.	Name	Type	Chr	Pos[Mb]	F_primer	R_primer
701	tarc2043	CoreSet	2B	563.9	CTTCAAAGAAAATTGGAAGTAAATGTT	TAGAAATTCCGAGACAATTTAATCTGT
702	tarc2065	Other	2B	572.6	GCAGTTCATTCAAGCCCAGTAA	AAATTCACTTAGAAGCTCAACATATACC
703	tarc2063	Other	2B	576.1	TTGTTCTCTTAATTATGTCCACATATTT	ATTCCCTAGAAACAGCAATTCCA
704	tarc2064	CoreSet	2B	576.1	CAACAGGGAATGTAGCTAAGGACA	TATAAAAGGATCGGAGTGAGGAAA
705	tarc0886	Other	2B	579.4	AATAAAGTAGGCAGCTCTTTTCCAC	GTAGCATTTTGTATGGAGTATACTAAAT
706	tarc0883	Other	2B	582.6	CCACCAAGGCTGGAGTCTCAG	ATGGTAGAAACAAATTAAGCAACACA
707	tarc2032	CoreSet	2B	583.8	CAATGTGCCAGCCTGAGAAA	CGCCGGTGCTCCAAGAAC
708	tarc2054	Other	2B	587.2	ATAGGACATTTCTACGGGAAACTG	CCCGCAGGTAGTGCTTCTG
709	tarc0869	Other	2B	594.8	CGCCAGATGCTCTTTGACG	CCTCAGGGGCCACACTTGT
710	tarc2069	Other	2B	595.1	GCCTCTTCACCTCCCCGT	CCCTTCATGGTCTTGAGCGAC
711	snp1393	Other	2B	599.0	TGGGACAAAATGGTGAGTGATT	GTTTCGACATCTGTTAAGCTATGCT
712	tarc2017	CoreSet	2B	601.2	GGTACTGCAACTCTGAACCTCTTTG	GATTTTAGGAGATCAGTTAGAAATGCTC
713	tarc0899	Other	2B	612.7	GCTGGCAGATCCCACAAGTT	GAGACTACCAAATCTAGGCAGGCT
714	tarc0908	Other	2B	621.7	CATTCACTCTGAAATGCTGGGA	CCTCATAATTTCCAAGCAAGTCCT
715	tarc2061	Other	2B	636.8	CGACACCTGACCAAGGAACA	TGCTTGCCTTGCCTTTGC
716	tarc2045	CoreSet	2B	639.2	GGTCAATCAGTATGTGCATCCG	GCTCTCCTCCGTGTCTTCCC
717	tarc2019	Other	2B	646.2	TGAACCCTTCCTGAAGTCGG	TACCTTTGCACAATTTTGACTACG
718	tarc0871	Other	2B	651.7	TTTCTGAAATAATAAATAGCAAGTATGG	TCGAAAAGAAAAAAGAGTGGGTC
719	tarc0852	CoreSet	2B	652.4	TCAGTTAACAGCCAGTAGTACCACC	TGTAATCTATTTCATCTATCCATTTTTT
720	tarc2046	Other	2B	653.3	CCAATGGGCAAAGAAGGTTACT	CATTTTGTACATAATTTAGACTTGAATT
721	tarc2057	CoreSet	2B	655.0	GCTTGAAATATGGTTCTTCCTTTTAGTA	CGTTCGAGCAAAGGGATAGAA
722	tarc2066	Other	2B	655.0	TATTGGTTTTGTCATCTCGCTGT	AATGTAATGCTGATGGTTTCCTATG
723	tarc2029	Other	2B	659.1	TTGAAAAAGTTTTCCGTGATACATT	AAGAACCGGCAGTTTAAATTGG
724	snp1389	Other	2B	660.5	TTTTCTGTTTTAAGCAGTTCACGA	TCAGGTAAGTTGGCATCTGTCTACT
725	tarc0897	Other	2B	660.5	TCTCTTCTTTTCTCTTTAAGCAGTTCA	TCAGGTAAGTTGGCATCTGTCTACT
726	tarc2072	CoreSet	2B	664.2	ATTTGGATCACTCTACTCTTTGATTCA	AAGCAAGAAACTTTACCCTCACAG
727	snp1708	CoreSet	2B	690.0	GTACAAATTGGTATAATGCTCCTACAGA	ACTTTACTCCCGCAAAAAAAAAA
728	tarc0900	Other	2B	709.0	TGAAATGCCCATCTACAACACAG	CATTATCATATGTTGTGCTTATGTTTCT
729	tarc2560	CoreSet	2B	709.0	AAGTTCAGAGTACCATATAACATAAACG	AAAAGGTATATTGTAATCTTTGAATCCC
730	tarc2552	Other	2B	713.7	AGGATGATGCAAATATTAATGCTCA	GATCTGTCAAATGTTCAAACCAAA
731	tarc2039	CoreSet	2B	715.0	CTGAATACCCCAGTGAGGATGTT	CCCTGTGAACGGGGAGTGT
732	tarc2555	Other	2B	715.9	GGACTGCCTAGAAGTTCTTTCCTTA	CGGGAGTTTTTGAGGGCG
733	tarc2051	Other	2B	716.6	GTTTGAGCGTAGTAGTATTGTTGTGG	CAAATGAGTGCAGTGAAATAATAAAAAT
734	tarc0910	Other	2B	722.6	TTTCTGTTAATATTTGTTCATCTGGTG	ATTAAGGTGATGCTCTATCGGGTA
735	snp7909	CoreSet	2B	724.7	TCCATAAGAGCAATTCTTTCATTGTA	TAGGAGTGAGGATTAAGTATTCTGCAT
736	snp8406	CoreSet	2B	740.8	TTAAAGAACAAAAGGGGCTTCAG	CATGTCTTTGTCATATATCCTAAGTGATA
737	tarc0901	CoreSet	2B	742.5	GTCTGATTGGTAAAAAAAAGTGATACAA	CTTTTCATATTTTTCTCAACAAAACTAC
738	snp8534	Other	2B	743.7	TTTTTTTTTATGATTTGTCGTTTTGA	CTAGCGAGGAGCCATGAAAGA
739	tarc0906	Other	2B	743.7	ATCTGCCATCTAGTAAGATCAATAGGT	GGTTAGTTTTCATACAATTCGCATT
740	tarc2554	Other	2B	745.7	CACATAGCCACTAGTTTCACCAGC	ACACGCTACACGCTTATTCTCG
741	snp5810	Other	2B	746.0	AGGACATATCCGACAGCGATG	GGACACTGGACGTCTAATGTTTTG
742	tarc2067	Other	2B	746.0	CCTCCTCGACCCTGTGCC	CCAGATGTGTGTCTTGATGCTATTTA
743	tarc0854	Other	2B	747.2	TCCCTTTTGGAGTACTATCAGTTTCTA	ACAATGCGTCCTTGCTCAGTATAT
744	tarc2558	Other	2B	754.7	TGGCTTGATGGAGTCATAGGGT	TGACTGACAACCGACTGGCTT
745	tarc0915	CoreSet	2B	759.3	TACAAAAAGGAAAACAATTGAACTACA	GAAATGCAAGAGAATTGGTGGA
746	tarc2551	Other	2B	760.9	CAGCGTCGATTCTGGTGACC	CATGTAACCATATCTGTTATGTACACTG
747	tarc2559	Other	2B	767.2	GACACATGACGAGTTCCACCG	GAACATAATCAAGTTCGAGCCAG
748	tarc2035	Other	2B	769.1	ACTGCCATGATCAACCTCATCA	CTTGAGAACGGTAGGGAAAACATA
749	tarc2553	CoreSet	2B	775.0	AAGCAAGTGTAAGTGAAAATAGAAGGT	GTCTGTCAGATGCCACAATATTCA
750	snp3596	Other	2B	775.2	TCTTTCTAATTCTGCTCTGGAATGA	TTGGTTCAATGCTGACCTAATACTATT
751	tarc0859	Other	2B	776.8	GATGGGTTCTTGTTGGTAGGTGT	CATCCTGAGGAAGTTTCTTGTAGC
752	tarc2557	Other	2B	776.8	TGTTTCTGCATGTTAGTTAAGTAATAAG	GGTTTAAGCAAGCGTGTCGGT
753	snp3474	CoreSet	2B	777.5	CTCCACTGTATGCGATGCTTAAC	TTGAGGCCCTTACAGCAGAAC
754	tarc2556	Other	2B	779.4	GCAACATGATGGACTGGAATGTAT	CTTGATAAAGTAACTGCAAGAGAGAGA
755	tarc2021	CoreSet	2B	779.8	CTTAAGCAATATAATCTGGCGGA	GACGGCACAGTTTTCTTTCAATA
756	tarc2022	Other	2B	786.0	GGTACAAGGATGATGAGGGAACTG	CATATGGAAAGATGTCTTCACTATTAAA
757	tarc2550	Other	2B	786.0	GCATAGGAGTAATAAGATTATCTGTTTT	AGAGAGCACGCCCCTTGC
758	tarc0892	CoreSet	2B	788.7	AAATTCTGCTATATCAGCAAACAGTGT	GAAACTGCACTAGACTTTATCAGAGAA
759	tarc0866	CoreSet	2B	789.9	GGGAGTTGGTCACTTCAAAGGTAA	TCCAGTCGTCCACTATTGACAGAT
760	tarc0902	Other	2B	790.6	TGTTGTCTGTCTATGGGATCTCTGA	CTGGTATATTCTGGTTGTCACTTTGA
761	tarc0865	Other	2B	795.1	AGTCGACGAACAAAACCATGAGT	TACAGGTCCCTGAACTCTTCTTTG
762	tarc0880	Other	2B	797.3	ATGCTGGACCACAAAGAACGT	AGAGATCTACTTCTGCAATGCTGTG
763	tarc0916	Other	2B	797.3	CAGCGAGGCCTTTTCGTTAC	CAACCCAGGAATCAACCAATC
764	tarc1340	Other	2D	0.0	CTCTTCTTGAGTTGACACCTCTCTTT	CTAATCATGTGTGCCTTTGTTTCTT
765	tarc1430	CoreSet	2D	0.4	CCTCTGGAGGGATACACGGAT	AGGCAATGAAGCAGACAAAAAA
766	tarc1346	Other	2D	5.7	AACGAACAAAAGAAAATTGGTCAG	TGAGGTTCCTGTCCAAAAAATG
767	tarc0144	Other	2D	8.3	ACAACATCACGAGCATTATTAAAGC	AGCATACAAAATATTACCACTCAGAGAA
768	tarc1373	Other	2D	8.3	CTAATTTAGTGAGTAATTTATTGTTGGG	GGCAATTCAAGGATGGCAGT
769	tarc1334	CoreSet	2D	8.7	TGATCAGGAGATGCTGCAACTT	ACACTTTGTTTAAATGCGATTG
770	tarc1426	Other	2D	8.9	TGATGCCGACAGCGACGA	CATGGATGCTCAGAACAGGGTA

Supplemental Table 4. List of amplicon sequencing primer sets for wheat.

No.	Name	Type	Chr	Pos[Mb]	F_primer	R_primer
771	tarc1463	Other	2D	10.2	TGTTTCATAGCATGTTTTTTCTAATAAT	GCATACACAATGAACAGGAAACTAAA
772	Inf17420	Other	2D	11.2	AGAAGATTTAGTGTCTAATCGTAACCAA	TGGAAAGACTACTCCACATTCAATAGTA
773	tarc1511	Other	2D	11.4	GAATCCAGACAATTACTTTTACTAGCAA	CACGCGATAATCCCCACTATT
774	Inf65191	Other	2D	12.7	GGTGAAAGCCTGTAATGGTCAA	GTTGTGACAGCACTTAAATGACTAGTG
775	tarc2386	Other	2D	12.7	TTGCTTGTACTTTTGCGACCA	TTTATTCTAGTGCTCGGCGTAGA
776	tarc2395	Other	2D	12.8	CTAACTCCCTATGGAAACAAATAAGC	GGAGGTGCACTGGCAATCG
777	tarc2385	Other	2D	12.9	TTAAGCAAAATATGTCACATCAATCTG	CCATGACACACCGACAGAAGC
778	tarc1359	Other	2D	13.2	GGCGTTCCCTAGTACCCAGC	AGAGGAGGTTGATGACACTGAGC
779	tarc1333	Other	2D	13.4	TGCTCGAAGAGGACCCGAG	ATGCACATGGCCTCAGTTGA
780	tarc2397	Other	2D	13.5	TTCACCCAGGACCTAGATCCTTAT	CGACTGCCATTATCCAATCTACTAC
781	tarc1495	CoreSet	2D	13.6	ACGTGCATTTTCCTATTTCCTTTA	TCACTCCCCTCCCCACAAC
782	tarc2389	Other	2D	13.6	TCCTGATGCTTAAGACACGAGACT	CCTACCCTGGCTGCAACG
783	snp1601	Other	2D	13.9	TTCTGATATTTGTGTTTGTGTATCTTCC	TGGACTGCCCAAATGTTTCC
784	tarc2384	Other	2D	13.9	TCATTGGTCAATGAGGAGTCCAT	TTTATGATAAGAAAACTAAAGACTGAGA
785	tarc1317	Other	2D	14.1	GCAATAAATTGTTGTTGGCGG	TGTCGATCCATCATCTATGGCTT
786	snp4746	Other	2D	14.4	CTTCTGGACCCTCATTATGAACC	GTTTGCTGTGTCTATCCTGAGTCA
787	tarc2394	Other	2D	14.4	TGCGCCCGATCTCCTTTG	GCAGAGAGCATAACGCATTGAA
788	tarc2396	Other	2D	14.4	CTCAAAAACATGCAGGGAAAAT	AGGTTTAAAAGGCACATAGACTCGT
789	tarc2399	Other	2D	14.4	GACATACTATTGATATTGCATACTGATG	AATAAAAATGCTAGGGAAGAGATGACT
790	tarc1374	CoreSet	2D	14.6	AGTTATATCCATAACTGAACAAAAGAAA	CGGGTCATACGGAGCGAAG
791	snp4354	Other	2D	14.8	CCTTTGTTTAATTCAGCGCCA	GAATAGTGCAAACAAAGATGGAGT
792	tarc2387	Other	2D	14.8	CTGGAAGAAGCTCTTCCATATGG	AATCCAATACCCAAATCCCG
793	Inf8540	Other	2D	17.6	GGGTTGGTTTATTATTGTTGATTGG	GAATAAGTTCATAATTGTCGCCGT
794	Inf54376	Other	2D	17.8	TCGCCTTAATGCCTGACTCAG	ATTTTCCTGGAGATAAATCTGATCTTAC
795	tarc1506	Other	2D	22.9	TCTAAAACCCTGCTTTCTATACCTGA	CACTAGGTTGATGGCTATCTAATGTTG
796	tarc1446	CoreSet	2D	23.0	AGATGACGGGTTTCGGCG	GTTGTCCTTGATCCCATTCTTTG
797	tarc1367	Other	2D	25.0	CCTCAAGGTTTCTGAGGAAGATTG	ACTCCCACTGCAAATAATGATGAC
798	Inf17749	Other	2D	25.0	TTCTTTAGAGAAACTTTAGTGTCATCCT	AGAACCGGTTGATGGCGG
799	Inf6081	Other	2D	26.6	GGCAACCTAACTTTGCAGGTTAA	ACACTATTGTCACATGAGCGCAG
800	tarc0147	Other	2D	26.8	AACAATAATTGCCAGCAAATAAGATAT	GATGGAGTAGATTAGGTTCTTATGTCG
801	tarc1327	Other	2D	27.8	CACAAAACTGCATGAATTACCGA	CTGCCACCAAGGCTCCCG
802	tarc0167	Other	2D	27.9	CAGCAGAGTGGTGGACAAAAGTTA	CGAGTTCAGCAAGGTTGGCT
803	tarc1326	Other	2D	27.9	TTGCCCGGAAAAAAATGGTAA	ATAATAGTGGTGCTTTTGTTTTTGC
804	Inf11824	Other	2D	27.9	ATGCAAGGCCTTTTGCACA	CGGACGAGCTGGACGCTG
805	tarc0170	CoreSet	2D	28.6	AGTGCTCTGACATCATCGTCGT	TCTTTGTACTGGACACATTTTATAACAG
806	tarc1384	Other	2D	28.6	CCCTTGAATAATCAAGAGTGCCT	CAAGAACATAAAATTAGACGTGTGACA
807	tarc0151	Other	2D	29.2	GGCCGAAAATCTGGTTAACAAA	AACCAGTTATGTGATTGAACTTCAAGA
808	snp989	Other	2D	32.8	GTGCCCGATTCTCAAGGAAAT	AAGACAGCAGCTCGAGAATCG
809	snp760	Other	2D	34.2	GGGAGAGTAAATCCATGCCTATCTA	AGGAACTACATAACACTTAGCAGTTCAG
810	snp927	CoreSet	2D	35.7	CATGAAGAGCGACCACTACGTT	GCCCCTTGAATCAGACAAGTC
811	tarc2398	Other	2D	39.4	CGTCTTGATATAGTAACCGGCAGTAT	AGCAACGACATCAGCGCG
812	tarc0158	Other	2D	44.7	ATGGCACAATGCGAATGATG	CCTCTTCTCCAAACGGTCCAC
813	tarc1416	CoreSet	2D	48.3	CCATACGTCCAGATACGAGGG	GTCTAGTGGGAGTGATTGAGGAAC
814	snp3248	Other	2D	62.3	CTTGTGTTGACTTATTGTTGATTGGTAT	CCAGGTTATATTTCCAGCATTACAC
815	tarc0163	Other	2D	62.4	CTGGCCGCTTAAAGAGGTACA	TGCCTGAAGGGTCTAACAACACT
816	tarc2392	Other	2D	62.4	TGTCCTAGTACTTTTTCCAACAACAA	CAGAAACTTATCCCCTTCTTTATAATG
817	tarc0164	Other	2D	63.0	CACCGTTTAGGGTCTCGGATAG	GCTCTTCTTCGTTCCCTCAACA
818	tarc2393	CoreSet	2D	63.0	CAACTTTACATTGTGAATGAAAAGAGT	ACCAAATCCTTAAATAAAAGTTCGC
819	tarc0169	Other	2D	63.6	GTTCAATGGCGTCGGCAG	ACACACTCTGGGTGCAGTATCAA
820	tarc0143	Other	2D	63.7	TTTAGTGTTGGACATGCTTGCG	GCTAAACCCTACATTCTTATTATCCG
821	tarc0160	Other	2D	65.6	CGTAAGTCACATCTATTTTCCTCTTAAGTT	GATCCGAGAACATAGCACCGT
822	tarc2391	CoreSet	2D	65.6	CCAGTGGTTTTTCATACTGGGATA	AACTTTTGGTTGCATGTAGCACTT
823	snp6374	Other	2D	67.2	CCTGCATATATTAATTGCAATTTAGC	CTGAGAGGTGTGAGCTTATGCTTC
824	tarc0166	Other	2D	67.6	CCATCCTGGAGGACCACTGA	GCTTAGAAGTAGCAAATGGCAGC
825	tarc0137	CoreSet	2D	69.5	TGCTGCAAAATCACAATATTATACAATA	CATGTTAAGGGTGCTTACCTGGA
826	tarc0141	Other	2D	71.8	CACACCAATCCATTAGGAGATTGT	CAGGAGACACTCTGAACTGTAACATG
827	tarc2388	Other	2D	71.8	GTCAGACGCCTGCCTGCTA	AGCTTCAGAAGAAGATGAAGTCTCAG
828	tarc0161	Other	2D	72.2	TGTGTATGTGATCTGCAATCAAGC	AGCTGAATGCCATGTTACCG
829	tarc0140	Other	2D	73.0	TCGCAGCATCTAATCCTTCG	AGAAAAGCACGTTTTGAGTATCTCA
830	tarc0145	Other	2D	76.5	GGCAAATGGTAACAGGTCGC	CCGTTTCTTCGAGGCACAG
831	tarc0162	Other	2D	76.5	ACAGCATACAAACAAGCACAAGC	TTACAAAAAGCCACAGTGGTCAG
832	snp209	CoreSet	2D	76.6	TCGCAGGAAGGGAAATTCCT	CGAATAATAACAAAATGGTGATTTT
833	tarc0168	Other	2D	76.8	CAAAACAGGAGATTTGCCTTGA	GGGATAGCCACAGGAAAGTCC
834	tarc0152	Other	2D	76.9	CTTTTCTTAGAGCTCCGTCGC	TTCAGCACCACAAAAAGAACTGA
835	tarc0159	Other	2D	76.9	TGTTACATCCATTTGAACTTTTCGA	AATCCAAACATTTCAAAGATGTACAA
836	tarc0149	Other	2D	77.1	TGATGTGATTTCACTATTCGAGCA	TACTTATGGAATTCTACTTCTAACAAACG
837	tarc0146	Other	2D	78.1	CTATTTAGTCTAGGAGATCAATCTAAAACG	TCTGCCATACGAAGTTCTGAGC
838	tarc0153	Other	2D	78.4	AGAGAGAGAATTATACATCTGCCGAA	TAAAAATTAACAGTAAGTTGAAGAAGTTCA
839	tarc0138	Other	2D	78.6	CATACTAAGAATTGAAGCCACGTTTA	CCTTGAAAAGGATTGGTGCG
840	tarc2390	CoreSet	2D	83.2	TCCACTGTACTTTCAGCCATTGTA	AGCTAATCCCTGCGACACGA

Supplemental Table 4. List of amplicon sequencing primer sets for wheat.

No.	Name	Type	Chr	Pos[Mb]	F_primer	R_primer
841	tarc1532	CoreSet	2D	93.1	CAAATAAACAAGTAGAACAAACTGGC	GAGTTGGCAGCAGGGGTCT
842	tarc0156	Other	2D	97.6	CAATCTTTCCCTTTCAGCACG	GAGGTTAAAAACAAAGAAACTGAAACTAA
843	tarc0142	Other	2D	101.4	CATGAACAAATATATGTTCAGGTGATAG	AAGGTTTCAGCTATAAACTGGGAA
844	tarc0155	Other	2D	118.0	CCTGACCATTCAAGTAGATAAGGAGT	GGAGAAATTGTTGTAGACTCCGTCT
845	tarc0157	CoreSet	2D	119.1	GTAAAATCTTCTCTCTGAATCCACATG	CCTTGACAGCTTTAAGATCATGAAGTA
846	snp2722	Other	2D	121.8	TCTTTGTTTTACTCTTCTTCTGATGC	CAGGTACTACACATCAGCAGGGAC
847	tarc1509	Other	2D	127.1	TCAGGGTCGGATCTGCCA	GCCGTCAAATCATGGTTGATAG
848	tarc0139	Other	2D	170.0	AAATGTAGAGTTCAGTAATAATGGGGA	CCGCTAGTAGGCTTTCTTCAATT
849	tarc0150	Other	2D	201.2	GGACTCACTATGGTTGGTTTGCT	GGTGATGCAAAGTTTGGAACTT
850	tarc0154	Other	2D	205.9	CTAATACTCCAATATTTGACATTGTTGTAT	GGATACACCATGCCGCTGT
851	tarc0165	CoreSet	2D	206.7	CAGTTAAGGAATTGACAGAGAGACG	TTTAAATTTTCTCATCATCCAACACT
852	tarc0148	CoreSet	2D	236.1	CAGCAGTGACGAAAATGGTTACAT	TTGTCCTCGGTTCCATCCAC
853	tarc0111	CoreSet	2D	270.8	CCTGTCACATTGTACAGAAAGACATG	ATAAAAAAAATGCAAGGTTTATGCC
854	tarc0115	Other	2D	293.1	AAGCACTGTGAGGTAATACATCGG	CCTCTGCATCGCTATCAATGA
855	tarc0112	CoreSet	2D	302.4	CAATGGCTTTCCAGCCTAGC	GCTTGTAGAGATTATCAGTGGCAG
856	tarc0099	Other	2D	314.1	GATTTACATTTTTGCGGGCTTT	GCTAAGTGACCAGGAGATGGCA
857	tarc0132	Other	2D	316.7	GTTGCGAAGTTTCAATCAATCTG	ATGTTTGGAGTTACAATGGTTGC
858	snp2961	CoreSet	2D	320.5	AGTCAATGAGCATATTCAACCACTG	CATAATAAGTTTCACTGGCTTGATTATT
859	tarc0127	Other	2D	320.5	GTTCATCTATACTCTCAACATCATCCATA	AGTCAATGAGCATATTCAACCACTG
860	tarc0092	CoreSet	2D	324.6	GCAAATGAGGGGCACAATG	CCCTTGGCTTATGAGTTCCTTTTA
861	tarc0126	Other	2D	330.5	AAAAGTTCAATAAAAACACTCTGACACA	TGGACTGCACTCTTCCTTGGT
862	snp5542	Other	2D	344.3	CTCTATGTTCTTTCTGCTCTCATTCT	CTTCAAAAGATAGTGCACAAACATTAC
863	tarc0118	Other	2D	347.8	AATGATGCCAATTCTTGCCAT	CAACTTAGAGCTTTTTGTATAATGACACA
864	tarc0096	Other	2D	349.0	TGAAAAATCTTTTTTGTGTAATTCGA	AACAATCAATACAAGGGCCTATACA
865	tarc0107	CoreSet	2D	352.2	TGAGCTATGATGCTTTATGTCGTAA	ACCTTGACCAACATTGGTATACATTA
866	tarc0103	Other	2D	354.7	TGATAGGAAATAAAAAGTGAAGCTCTT	ATCTGCTTGAATGGATAGATCATGA
867	tarc0113	CoreSet	2D	354.7	GGCAATCCAAGTCGTGCAG	AAAGAACAACTCTAGCTCAGAAAGCT
868	tarc0086	Other	2D	356.0	AGAGGTGAATGAAGACGTGGTG	ATGGCGCGATAAACTCCAA
869	tarc1393	Other	2D	364.9	TATTTAGTTGAATTGAAAATGTGCTCTA	CCCACAAGATCCTCGATCCTG
870	tarc0133	Other	2D	370.6	GGAGACCGATCAAGCAGGTTA	ATCAAGAGCTTGCATAATGCTTATT
871	tarc2380	Other	2D	378.1	GATTGAACTTGTCTGTCCAACGTT	AACTCACATATATCATCCCAAGGTCT
872	tarc2379	CoreSet	2D	386.2	GGGTCAAAAGCTGCACTTCG	TGTACGCCGGCGTTCCAA
873	tarc2369	CoreSet	2D	402.2	CTACTACATTTTGCACATTTGAACTACT	GGGCAGTAAAATATTGCTCACATT
874	tarc1307	Other	2D	412.5	CCCTGATCATTTCATCAGTGGAA	GTGCAGAAGACTTGAAGAAGTGG
875	tarc0125	CoreSet	2D	421.8	TTTCAGTGGAACAGTGCAAGACT	TTTGTCTACCATTGACCCCATATT
876	tarc2372	CoreSet	2D	428.7	TATCATGTTCCTTAAAACTAAGGCAA	GATTATGTCTGTGAAGGTATGGTGG
877	tarc1518	Other	2D	432.0	CTTGCTGTATTGGGTGGTTGTT	TTTTTTTCTTAAGCCTTTTACGGTT
878	Inf20112	Other	2D	435.0	TGCTACAAAAGGTGTTCAAATTTAAA	GGCAGCAATTTTTTTAGGGG
879	tarc2368	CoreSet	2D	437.9	GGAGCAAAGCTAAGCCCCAT	GGGAAATTCCAATATCCATGAAC
880	tarc1533	CoreSet	2D	449.1	TAGAGTCCATGACAGTATAAAAAACCC	TCACAGATCCATGTTTGCCG
881	tarc2371	CoreSet	2D	450.5	AAGTGTATTTGTTACGTCGTTATTGC	GTCATTCCTTTGCCTATCTATTGTT
882	tarc0087	Other	2D	473.2	TGCTGGGGCCTAGTCGTG	ACTTTCTGCTTGCAGTGTGACAG
883	tarc2382	Other	2D	473.3	TAGAGTGCAGACAATCAAATTAATACTT	ACCTTGTTTACCCTGACCCTTT
884	tarc2375	Other	2D	474.5	CACGTACTGTTAATTCTGAATCACATTT	CAACAATTACCTAAAGAAGCAAAATT
885	tarc2377	CoreSet	2D	478.7	AGCAGTATTTATCTTCCTATAACTCGTC	ATTTTACCTAGTGATTTCATATCCTTCC
886	tarc1523	Other	2D	485.0	AACAGCGGCAACTCTCACAG	CAGTCCACCTCAAAGGACAGC
887	tarc0117	Other	2D	492.7	ACAGGAAAAATAAAAGTATAGGAGCAG	TTACCCACAAAATGGCCTCAT
888	tarc1265	Other	2D	498.8	CTTTTTTTGGTTTGTTATTGGCA	CATGAAAGTATGTAGAAGAAGTACCTGT
889	tarc1305	Other	2D	513.0	CCTTTGGTATAATCCACAGATTTGAA	ACCCATTTTCCCTTTGCTTG
890	tarc0110	CoreSet	2D	520.6	ATTGACTCGGTGATTCACATATCC	TTTCCTAAAAGAAAGAAATACAAGGG
891	tarc1503	Other	2D	523.2	CCAGTGATGATGTCTTTGTCACATG	CTGCAAGTACCATGCTTGTATCAA
892	tarc0116	Other	2D	542.1	TTTCGTCAGATTCACGATAGTCTGT	CGGCTGGCAATAGTTCGAGA
893	tarc0136	Other	2D	544.9	GTAGATACAGAACTAGGAGAGAAAAATACA	CATATCATTCACCATAAGGATCTTGAT
894	tarc1397	Other	2D	556.1	CTTTCCCAAGATTGGGCAATA	GTTGAATTGTGTGACATGCAAAAA
895	tarc1360	CoreSet	2D	556.9	CCTCCTCCTGAAACAATGGTGA	CGAATACATTTTTCTTTCTTCCTGAGT
896	tarc1289	Other	2D	571.0	TTATTTATCTTATCCTGTTCTTTTGATA	AAAGTTTGGTTATTCGGCGG
897	tarc0100	CoreSet	2D	573.8	TTAGATCAAACAAATCATGGGCA	TATGCCTTTACTTTAGCAGTTTTACAA
898	tarc1324	Other	2D	578.6	CAGTAGAAGCTCAGCCAGTCAGTC	TTGTTAATGAATTTCCTTTATTTACTGT
899	tarc0101	CoreSet	2D	582.0	GCTAAGCACTTCTAAATTGGAGTTCA	GATGGTGCTGAAACTCGTAAGG
900	snp2164	CoreSet	2D	587.3	GAAAACATTTTTGCTTGTGTAATGC	GCTAAAACAACTTCATGTTTAGAGTAGTA
901	snp3940	Other	2D	587.3	GGTTGACCGCATGAGTGTTAG	CATAGAACTAGAAGAAACGACAGCCT
902	tarc1335	Other	2D	589.9	GGAGGTTGCATCGAGTAATCCTAC	GTGTTCCCGATCCCGTGT
903	tarc0106	CoreSet	2D	594.5	TGTCATTATCAAATTAGAAAAGCATGT	ATATGTACTGGAAAGGCTCAAGTGA
904	tarc2374	CoreSet	2D	600.3	TGCAACCTAAGATTTACAAGCATGT	GCTACAACATCTTATGGGGTTTCC
905	tarc2381	Other	2D	601.0	GCTGGAGGCTTAACAGCAGTG	ATTATTAGGTCTGGAATGTTGCGA
906	tarc2370	CoreSet	2D	601.2	GAAAGGAAATTTGTTTTCCCCT	TAGAAGTGGATGAGTGATCGCTCT
907	tarc2383	CoreSet	2D	601.6	CATACTTACTCGTCTGATATTCCGC	GTTATTTGCCATCATGTTGCATAA
908	tarc2378	Other	2D	602.5	CTTCCTAAGGAATTTGAGATGTTCAG	TTACCCAGTCACGCTTATATATCTATGT
909	tarc0114	CoreSet	2D	607.4	GTCGTCTATCTGGAGCACTTCG	ATTTTAGAAGTACAGCTTTGTAGCAACTA
910	tarc1475	Other	2D	607.7	CAAAACTGTGAGTGCTGCCCT	AGGTCCGAAAGGATCACGC

Supplemental Table 4. List of amplicon sequencing primer sets for wheat.

No.	Name	Type	Chr	Pos[Mb]	F_primer	R_primer
911	tarc1486	Other	2D	607.7	GACAAAATCACGATAATTGAGACCA	CCAATGTTTACCGTGTTGCCT
912	tarc0119	Other	2D	607.9	TGGTGACAGTTATGTCCATCGAC	GATAGTGACCATACAAAAAAAATAGTCC
913	tarc0097	Other	2D	608.2	AAACACCAGCCAAAGGGAATC	CTATGGTTGATGGAGAATGTTGATG
914	tarc0091	Other	2D	608.5	GAGTGTCGATACCTTGGAACTCATAC	GTTACTCTCTGTATGCTCGACTGCTA
915	tarc0095	Other	2D	608.5	CAGCAGGTTATCTTTAGTACATGCTACT	GTTTTAATCGAGTGTTCTCAGTTCAAT
916	tarc0123	Other	2D	608.6	GAATGGTTCACATAAATGGTTCAGT	GTGTATTTATCTTTTGATTTGGTATTGA
917	tarc0130	Other	2D	608.8	AAGTACCCTTGTCTTGTGCTTCG	GGCATATTGAAAAATTGGGCA
918	tarc0134	Other	2D	608.8	CAAAAAAATTATTGGAAAAGGGG	ACGTAACCAAGACCGCCTCA
919	tarc0102	Other	2D	608.9	GCCACCTTAATCAAGCATAAACAT	ATCAATACTTGGGCAAGGCG
920	tarc0120	Other	2D	609.4	CTTCGGTAGATTTCAAAACTATGAGT	ATACATAAGTATAAACTTACCGGCAAGA
921	tarc0085	CoreSet	2D	609.5	TCCACTCTTTGATACTGATGTAGTACTGA	ATACATGATAATCATTAGGAACACGG
922	tarc0105	Other	2D	609.5	GTGCTGTCCCCAATTTTTCTC	AATTGACAATTCATCCATGCTTTC
923	tarc0089	Other	2D	610.3	TGGTGATTGAAGTAGTTTAATGAACAC	GCCAGCCAGGAATTAGTTAAGGT
924	tarc0090	Other	2D	610.3	GATGCTATTTTTGTAGCTCACCG	CGAGGAGGGAAACAAGTGCA
925	tarc2376	Other	2D	610.3	TTCCACCTGTATCACTCCCCA	TTAAACTTAACCTATAGTTCCCTCCG
926	tarc0108	Other	2D	611.2	ATATGAACTCAGGGGAGCGGT	TGGACAAAGAGATGCTCTCGATT
927	tarc0093	Other	2D	612.3	GTCTCTGACGTTTCTGATACTCTGG	GCAAGAGGTTTTGCTTAGGCT
928	tarc0104	Other	2D	612.3	GGAAAACAGAAATAATTGAATAAGCA	AGAACTCATGAAGATCATTTTCAGAAG
929	tarc0121	Other	2D	613.1	GCAGGGCGATTACTCAGGC	CCAATAGCTTGCTGTGAGTGCT
930	tarc0122	CoreSet	2D	613.4	TGCAAACCCTCGATAAATTATTTTA	GGAAAGCCTCTCAAATGTAACACAC
931	tarc0129	Other	2D	613.4	CCATTGGTTGTCATCTTTCTTTTT	CACCAATATGATGTTTTTGCTCAC
932	tarc0094	Other	2D	614.9	ACAGGAGGCCCTTCGCAT	AGAATGAAAGCCAGCCAGTCA
933	tarc0098	Other	2D	615.1	CAAAGTGAGAAGGATATGTATAGCAGAT	GAAATGTAATGAACAACTCAGTAAGACC
934	snp229	CoreSet	2D	619.6	ACTACCTCAAAGTTGAAGCATGTAAAC	CATTAAAGCAAATCTTATACATTGACTTC
935	snp3849	Other	2D	619.6	CCCGCTGTTGGATCTTTGTAT	AGTTGGGCAGATTATTTCGG
936	tarc2373	Other	2D	620.0	TATGAGGTTCTTATCTCTTGATTTAAAC	TTTCAAGTTCCATGGTTATTTATTCA
937	tarc0088	Other	2D	625.5	GGTGAGGGGAGGAAGGTCT	TTGAGGTTGAGCAGCGTGAT
938	tarc0131	CoreSet	2D	626.6	CGGTGCAGCTATGCAGTGCT	CTAGCTCGAAGCTAATAATCGGTACT
939	tarc2367	Other	2D	631.9	AAGACAAGTTGTCAAGCATGGG	GAGAGCAAATAAGTTGTACTATGCTATT
940	tarc0128	CoreSet	2D	633.2	TCACCATGCAAAATCCGATAGT	CAACAACTCTGCTGCTCTAATTTT
941	Inf11457	Other	2D	636.0	GAAAATCGATGGTCAGCGC	TGTGTGGCGTCGTCACCA
942	Inf6763	Other	2D	637.7	CAGTAAGAACTAAATCTTTTGATCCTTG	GGGGATCCTCTTGACCTCGA
943	tarc0109	Other	2D	638.1	GGTATGTTCACTAGGATTTCAACTGT	CCAGGAAATAACATCAAATTAACGT
944	tarc0135	Other	2D	638.1	TTTAGCTGAGGTCACTGGGCTA	GAAAATAAACCATAACTTCTACAAGCAT
945	tarc0124	Other	2D	638.4	CTATAGCACCAATCGATAAAACAGC	GCACAGATAACTTAAAAGCTGGAGT
946	tarc1540	CoreSet	2D	641.1	AAGGAGTCCGACAGCTTCGTC	CCGTATGCCTGCGTGAAATAA
947	tarc1431	CoreSet	2D	644.7	CACAACGTCCGTCCCCTC	GCGTGTGATGCGTGCTTACT
948	Inf22476	Other	2D	644.7	AGGATGAGCACGCTTTCTCG	TATCAATGTGATACTTATGCTTTCTGAG
949	Inf64731	Other	2D	646.0	ATCGTTATTGGGATTTCCAAGTT	GCATAGTGCTGCTGTTGTTGAGAT
950	Inf64107	Other	2D	648.3	GAATGTAGTTTTCCCTGTCCCAT	ATCTCTTACATTTAAGGTAAGGCAGG
951	Inf64223	CoreSet	2D	648.5	GGAAAAATCACGACGAAAGCAG	TCTCCACCATGGGCACGA
952	tarc0548	Other	3A	8.6	TCAATAAAGAGAATGGTCATTATACAAA	ACCATGTTTCCTTTTTTTTCCC
953	tarc1758	Other	3A	12.5	CGATCTCTCTCCCCCTATCTCTT	GCTTGGGCAATTTGGCGA
954	tarc1754	Other	3A	13.0	CCAGTTCACTTATTAATCTTATGAGTAA	GTGTTCTTCACAGGTGGCATTG
955	tarc1757	Other	3A	13.0	TGGAGACTCGTGGTTGGCAC	GGCAACTCTAATAAACCAAACAAGTTAC
956	tarc1753	Other	3A	13.5	ATGCACAGGTAACATCAGAGGGT	CATAGCTGTGGTTTAACACTTTGGA
957	tarc1760	CoreSet	3A	13.9	GACCCTTCTCATGTACTTCACGA	GAACAGGTCTGTGACGCCATC
958	tarc1755	Other	3A	14.0	CTACTAGTTCACTTGGAAAGTTACTATC	GAATCACTTATATTTTCTACCTGTATGT
959	tarc0513	CoreSet	3A	19.9	ATAGAACCCATTGGTTTCAGTCG	CTTGTTGAACACTCGTTGTCTCTTT
960	tarc0533	Other	3A	20.3	CTCTCCCTCTTCTTCCTCCTCC	AACACCTCCCTGAATGAACGA
961	tarc0528	CoreSet	3A	20.8	CGCCTAGATAGTTTTCCCATTACA	CTTTTTTATCTGGATGGTGGAGATA
962	snp3939	CoreSet	3A	23.7	TCTATACAGAACAAAGACGCTCTCC	CCCTCCAGATTTACTCTTGCG
963	tarc0532	Other	3A	23.8	GTGAGCTTATGTGCACGACTTGT	GATATATGCAGGGATGTGCTGATAG
964	tarc1756	CoreSet	3A	25.9	CAAGCCATCCTTGAGCAAAGA	AGAACCATGAAGTTGTTAAGCTGAT
965	tarc0543	Other	3A	32.1	GTCTGCCTGCCCTACCCAT	AGATGAACAGTTACAGCCGGAGTA
966	tarc0527	CoreSet	3A	32.2	CATATATTTTCTTGTTTGCCTTTCGT	CCTGAAAAATGACTTGTTTAGATGCT
967	tarc0537	CoreSet	3A	45.6	AAATAGGATTCACTATTTCATATTGTTC	CGACAATCTGACCTCCTTCGA
968	tarc0517	CoreSet	3A	59.8	AATCCAAGGCAATCGAAACC	AGTAAGACCACGAGCACGGC
969	tarc0524	Other	3A	61.2	CGTTTGCCACCAACCCTAA	CTTTGCTAGACAGATGATGCGA
970	tarc1759	Other	3A	72.7	ATGTTTAAATCTTCATGATTCATTCAGT	GCAACAAGCCAGAACAAACAAG
971	snp5151	CoreSet	3A	95.0	TCGCTCAGGATTCACCGTAAC	GTCCATGTGGAAGCACATGCT
972	tarc0531	Other	3A	140.0	TCTAAAGCCCAACGAGTTGACA	GTGGCTGCCCTTGTGGTCT
973	tarc1752	Other	3A	147.8	ATTCCTTTTAATTAGAATATACTCCCAA	CAAAACAAAAGCATAGATAGAGACCA
974	snp1308	CoreSet	3A	176.6	AGATGAGTAGCACTCATCGTATCCT	GTTGATCGACATATACAACATTTATGG
975	tarc0512	Other	3A	204.9	TGAGCTACCATATAAAAGATGTAGGAGA	CAGGCTTGGGCATGGATACTAT
976	tarc0515	CoreSet	3A	211.5	GATAGAAATTACCAATCCAAAATTAAAA	GAACCGATTCATCCGTTTCTAATAT
977	tarc0520	CoreSet	3A	227.9	CCTTGATCAGTTGATACTGACCCTC	ACACAAATAAGATATTAAAATTGTCCCA
978	tarc0535	Other	3A	262.5	GCATACCTGAGAAGTAAACGACATG	GAGGGCTTGAATTCCTCCATG
979	tarc0529	CoreSet	3A	343.2	GTGCAGCATACTTGCGGACA	TCCTCGTCATCTGCACCCA
980	tarc1716	CoreSet	3A	379.8	TTCTCTCATGTGTATCTTTTCTTAGTAC	AGCTGCAATTATGTTCTATCATTTTC

Supplemental Table 4. List of amplicon sequencing primer sets for wheat.

No.	Name	Type	Chr	Pos[Mb]	F_primer	R_primer
981	tarc1697	Other	3A	477.7	ATTCGAACTGAGCAAAAACAAAAT	GTTTGCAGCTGTCGAAGAGC
982	tarc0536	Other	3A	478.7	TGTTATTTTCAAAGTTTAATACTGGCA	GTCTGAAGAACTTCTATCTTTGCGAT
983	snp5005	Other	3A	480.1	TCAAAGATGGTTCGGTTGCC	CAAGCCAAAACAAGAAATACAGGT
984	snp5786	Other	3A	480.1	GCCACATATGGCATCTCAGGT	TTCTTCTTCCTGGAACCTAGTCC
985	tarc0514	Other	3A	481.0	CAAGGTTTTATACTAAGGTGAAAATGC	GATAAGCAATGAAAATGGCGAA
986	tarc1717	Other	3A	485.6	CATACTAGGATAAAAAAAATCAAACTCG	AATTTAAGATTTTATTGTGCAACTATGT
987	snp143	CoreSet	3A	487.4	TCACTCATTTCGGTCCATATGTACT	CTCCTGGCCTGTCTGAAGATATT
988	tarc0506	Other	3A	487.5	CCTTTTGATGGGCTACCGA	AAAGGAATATCAGTTCCACACGC
989	snp6256	CoreSet	3A	501.0	GCATTATCATCACATGGTGTGG	TGGTGCACTGACCCCGACT
990	tarc0507	Other	3A	501.2	CAGTTGTCTTACTCTTTATCACTCTTTA	CATCACCTTTGATATGATGGGG
991	tarc0539	Other	3A	501.2	GGTATATATCCTGGTGCATATGAAGAAC	AGAAATGATGTTAGTTATTAAACTCCTT
992	tarc1702	CoreSet	3A	502.4	TGTGCAACAACAATAAAACAACCTT	CGCGATTGATTGTATCCAGAATG
993	tarc0540	Other	3A	505.2	CGTACTACTGACATTCTGTTTATTGATG	GTGAGAGGGGGAAGGAGAGAC
994	tarc0542	Other	3A	513.9	CAGCACCACACCTCTCCGT	GGTCTCGCCCCGTGCTTA
995	snp1378	CoreSet	3A	514.1	CACGTTGAATATGAAGTAACACATTCT	CAATTTGTGCCTTTGTAGGTTCTT
996	tarc1719	Other	3A	514.3	GGCCAGATAAGATAAGTCTTTGTATTTT	ATCTTTACCTGGTGTCCGTCG
997	tarc1724	CoreSet	3A	514.6	ATTTATTGCGAATTCACAGTAAACTATT	GCATTGCCTCATTGCTCCTAT
998	tarc1734	Other	3A	515.2	ATATTAGTACACATACATTGCATGAAAG	AGATGAACCTTCTCATTTACTGTTGAA
999	tarc1698	Other	3A	516.6	TGTTGCACTATAAAACATACTTCTTGCT	AAAAGAGAACTGTATTAAGCTTTTGGTC
1000	tarc1718	Other	3A	516.8	TTAGGGTAGCATATACTGCTTTTGAAT	CCACGTTGTTGCTTTTGAAGG
1001	tarc1714	Other	3A	517.5	TGGCAACATGCACACAAATAGA	AAACATTAGGACGATGGCAGC
1002	tarc1701	Other	3A	521.3	TGTACTGGCTCTGCTATCACATCC	GAAATCTACAAACAAATTTATCACTAGA
1003	tarc1732	CoreSet	3A	521.3	CAATGCACCTAAGGTCTCTCCC	GGTGTCTGAAATTCAAACAGCAA
1004	tarc1743	Other	3A	522.1	CTACGAATGGCACCTCTATGAAT	ATCTGGTCCACCTGCGATG
1005	tarc1725	Other	3A	523.6	AAAGTACATCCTCTTTGGCATAACTAT	TGCTGGAAATTTCATAGCAATTTC
1006	tarc0545	Other	3A	526.5	GCTGCATTTTCAGCAGTACATGT	CAAAGTATATGCAATATTCACAGAGGTC
1007	tarc1723	CoreSet	3A	526.5	TTGGGCTTAAATTTGTGGATACA	TTGAAAATGTTAGAAGTGCGTGTG
1008	tarc1696	CoreSet	3A	528.5	TTAATTTATCAAATATATGGCATGGAAT	AGTAAATATTAGAAGAGAGCATGCTAGC
1009	tarc1750	Other	3A	532.3	CTAATATCAGATTCTCCATTGCCA	CAAAAGGTGTAGCTGGAGTTTGAG
1010	tarc1703	Other	3A	532.5	TTTTTTCAAAATACCATGCTAAAGACT	AACCTCAATTTTACAATAGTCTAACAGA
1011	tarc1712	Other	3A	532.8	CTAGCTGCAATTTTTAAAAGGGAA	CCTATGTTGTGAACGGTAGCTTAA
1012	tarc0522	Other	3A	532.9	CTGGTGTGACGTCTGCTTGTG	ATTAAAATGATGGGGGAGGTTG
1013	tarc1708	Other	3A	532.9	AAATTCTACTTAACAAATATACAGTTGG	TCATGGGGTACCTCTACACGG
1014	tarc1727	CoreSet	3A	533.6	ACAATCTTAGAGCCCAAAGAACAG	TTCACAGGAGAGGGTTTCTGAAT
1015	tarc1748	Other	3A	533.6	TGACGTTGAGATAATCATTTGTCAGT	CCAAGGTGATATATAATGACCGCA
1016	snp1998	Other	3A	533.7	CTGTAGTATCCTATCATCATCACTCCA	GCCCAACCTCGACTCCCTAG
1017	tarc1728	Other	3A	535.0	GAATTCCAGTACAACTGTTTTACACG	TTATGTTATTTTCCAAGTGGTGCC
1018	tarc1740	Other	3A	535.0	TAAATTTACACTAGCATCAATAGAGCTT	TTATGATATGTAACTTGATTTATCCAGA
1019	tarc1737	Other	3A	535.2	ACATTTTAGTTTTTAAGACTGCTATTGG	CGAGTGATTGGAGTATGATGCC
1020	snp132	CoreSet	3A	536.6	ATGGAATGCAAGAAAACACATGA	TTTATCAACCACAGAGCTATATGCTATAT
1021	tarc1705	Other	3A	536.6	AAAAATCAAGAAATAATAGGTTCACAAG	GTATTTTCAACGGAATGCCAGA
1022	tarc1730	Other	3A	538.0	TTTCAGCGCGATACCAGTTG	CTGCAGATACCGATTTGCACAT
1023	tarc0518	Other	3A	538.8	ATCAGTGTTTTGAGAACTTGTTAGATAA	TTTATGAGTTCCCAAGATATGACAGAT
1024	tarc1695	Other	3A	541.0	GAAGTAGCTGTCAGTTTCTTCTCACC	CTCTGTATTACCACAAGTCCACATAGTC
1025	tarc1745	Other	3A	541.3	GATAACATTGCAACCCCCTTC	GCATCACAACTGCAGCTTATTCAG
1026	tarc1739	Other	3A	541.6	CCACAAAGGAGGATGCCGA	CCATTTCTTCTATGCAATTTAGATGT
1027	tarc1720	CoreSet	3A	541.7	GAAACGGAAAGTACAAAAAAAAGGT	GTTTCCTTCCTCCGCCTG
1028	tarc1741	Other	3A	542.2	AACAATCTGCGCGGAGATTT	TTATTTAATATTTACCTCCAACAAAAAA
1029	tarc0544	Other	3A	549.3	GCACTGTGGCACATAAACCATATTA	GGCGACAACAAGACAGGTGAAT
1030	tarc0526	CoreSet	3A	550.7	AAAGGGTAATTTGGTGTTGCGT	TACAAAATGATAGGTTAACAGAAGTACC
1031	tarc1735	Other	3A	556.5	TCATCCACAATTTTAAGAGCAGC	CCTGATGCACACAAAGGTTCG
1032	tarc0530	Other	3A	558.0	ACGCATGAAAACAACTAGTGGTG	GGCCCCGAACATATGCTTTAC
1033	snp1983	Other	3A	562.4	TTTAGTACATCGATCATATACATGTACAGC	GAGTTCTGCAAATACTGCTAGATCTTC
1034	tarc1722	CoreSet	3A	581.3	CAAATAATTGAATCATTTAGAGGATTAT	CGCTAAAGACAATGGGGTGAA
1035	tarc0541	CoreSet	3A	595.9	AAATTTCGCAGATAAAGGATCAGTT	AAAGAAATAAGTTTTCTCTAAATGGCA
1036	tarc0521	Other	3A	619.6	GTTAGCAGACATAACAACAAACACATTA	CACCTACATTGTACACTTTTAACTCTTG
1037	tarc0525	Other	3A	624.4	CCACGGGAGCAACTCTCTG	GGGGTTTGTTTAGAGACCTGAGT
1038	tarc0546	CoreSet	3A	627.8	CGGTGAGATGAGAGGAGCTTTTAG	CACGTTCCCAGGTCATAGTTGT
1039	tarc1691	Other	3A	628.0	TTTATTATTAAATGTATCATAAAGTGCG	CAACCATAAACAAAAAATCACACC
1040	tarc1699	Other	3A	633.9	AATAAAGTAAAGCAAACCCAGAAAGT	ATTTTAGTCCTAAAGTTTATGCCTACG
1041	tarc1749	CoreSet	3A	635.2	CAAAAACGCGACAAAACATACAA	TCAGGCTGTTCACACAAAATGG
1042	tarc0547	Other	3A	638.3	CTAACCTACGGTCACAAGATCACAC	GATTTCTCAGATGATGGTTATTGTTGT
1043	tarc1747	Other	3A	639.2	GCCCTCCTTGAGGATGTCAA	GCAATTCATCTTAATACTGATGGTGG
1044	tarc1713	Other	3A	642.0	TGCTACCACCTAATGAATCATTTCTT	CATTATTGCTCCTTCCCCGA
1045	tarc1731	Other	3A	642.2	CTAGGGCAGCCGCATAAATT	AAGCAATTGTCGGCGAACC
1046	tarc0509	Other	3A	645.1	GGTTCTACCGAGCCACGC	TACTGTGTGTCATTCCTGGTGGTA
1047	tarc0538	Other	3A	648.9	GCATACTGCATGATTGATGTTCC	ACCATCTTTGAGCAAATTAGGCT
1048	snp4296	Other	3A	649.0	GGCGAATGATGATCCTCGTT	GTACTTAGGCAACACGCCGA
1049	tarc0503	Other	3A	649.0	CTGAGAAACAAAAATATCATGCTCTAT	TGTGTGCAAAGGACACTCCAGT
1050	tarc0516	Other	3A	649.0	CCAAATAAGAAATGAGAAAGGAAGA	CACATGCGCCTGCTCCAA

Supplemental Table 4. List of amplicon sequencing primer sets for wheat.

No.	Name	Type	Chr	Pos[Mb]	F_primer	R_primer
1051	tarc1706	CoreSet	3A	649.0	TTGTAACAAAATACAAGATGATATTGAC	CCTATTCCACATCCAGCACCAG
1052	tarc1744	CoreSet	3A	649.7	CCGGCGGAAGCTGGTCTT	GTTGGGATACTCTTGCTGGTATTC
1053	tarc1736	Other	3A	650.4	CTGCGGAGCCTCCGACCT	AGAAACTTTAACCCAGTTCTGATGAC
1054	tarc1726	Other	3A	651.5	GCATGCTCTCTCTACAGGGGT	AGCTCAAGTGCAATAGCTGACG
1055	tarc1709	CoreSet	3A	651.6	TCGAACAATTATAGTGCCTGTTTCT	CGATGGACACATTCAATGTTGC
1056	tarc1704	Other	3A	652.0	CTTCTTCTGGTTTGCACATGG	GGATAGAATGAAAGAATGCGGA
1057	tarc1746	Other	3A	653.5	CATCTCATTGGAGGTGAGTTTCAC	CAAGTATATCCCTAAATTCACTCCTGT
1058	snp1891	Other	3A	659.2	GGAGGTAATTTCCGGTCAAGG	CTAATGTATACAGATAAAACTTCATCGC
1059	tarc0534	Other	3A	661.6	ATCAGGCAAGTCAAAATAACTCTAAAT	TCATTGACTCGTTGTGCATCTGT
1060	tarc0510	CoreSet	3A	667.8	CCGTCGTGAGACCACCGAT	AAGATCTCCGGGTTCGCAC
1061	tarc1693	Other	3A	682.4	ATTTGTTTGTATTCCCCTCCTTTT	CCCCATTTGAAGAGTAAATTCATAA
1062	tarc1707	CoreSet	3A	683.0	CGTATGTCGATCTACATGTCCGC	TCGAGAGGGGGATCCTGC
1063	tarc1715	Other	3A	683.0	GAAGCCTCAAGCCCGTCAA	GCTGAGCTCTGGCTTTGCC
1064	tarc0523	Other	3A	684.3	CCAGCACCTTTTCAGGACGA	TCTTATATTTAACTCATTTGTGTTCATC
1065	tarc1692	Other	3A	684.3	AGCAAGGTAAGTCTGCACTTTTATACT	AAGTTGGTACAAGAGAAGACGGGT
1066	tarc0505	CoreSet	3A	684.7	CACCAAATTATGCCACGCC	GTGATGATGACGACGACGATG
1067	tarc1700	Other	3A	685.1	GAAGACTTTGTGTATGGAGTGTATGATG	GAGTGTACCTTGGAATCCCTGTG
1068	tarc1738	Other	3A	686.1	GGCAAAACTATGGAAAATTTCAA	TACAAGACTCATGGCTAAGAGGCT
1069	tarc1729	Other	3A	688.6	ATAGCTAATAGTAAGGTTTTTTTACCAA	ATCGATAGTGCTTATACTATTTTGTGCT
1070	tarc0504	CoreSet	3A	698.9	GAAGGCCACACTGGAACCA	CTGGATCAAACTGATGCCTAAACT
1071	tarc1751	Other	3A	700.8	CCCTTCTTTTTTCTGATGATGGT	CATCCAATTGAAAATAAAAAGCGA
1072	tarc0549	CoreSet	3A	712.1	TGTTCATTTCCAACTCATGTGAATC	GCATGGCATCGAGAAAAGAGA
1073	tarc1742	Other	3A	716.1	GCATTGATAGGGAAAGTTACTCACT	GATACAAGGTAGTAAAAAAAAGCATAGC
1074	tarc1711	Other	3A	716.4	GCTTTTCCGATCATAACAATAGTTTA	GGGCCGTTTGATGCTCCG
1075	tarc0511	Other	3A	719.3	CAAAGCAAGCCAGGTGGTG	ATGGGCCGAATGGAAGCC
1076	tarc0519	CoreSet	3A	726.1	ATAGCAGACAACTACAGCTCGGTAG	CTGTCTTATTTCTCAACGTCGGA
1077	tarc0508	CoreSet	3A	727.1	CATAGTTTGTTTCTCCAAGATACCAG	ACAATTTAAGACCTAGTTCTTACCATCA
1078	tarc1721	Other	3A	739.1	TGATTGGCTTGTTGAGGTTTGTAT	TCCAACGAGGTCGGATGTGTA
1079	tarc1694	CoreSet	3A	739.2	GTCGATCTCACCAACATAGTACTCAT	GATGTCTTGCTGTCTTCAACCG
1080	tarc1710	Other	3A	742.5	AGCTCGCTCATTTTATAATGTCAAA	CGAGACATCGGTGATCGCTT
1081	snp2870	CoreSet	3A	743.8	TGTTGCCTTCTTACTCTACTGCTTC	AAACATAGGATTCAATATAGTCGATTCT
1082	tarc0952	CoreSet	3B	0.2	GCACTATGCTAATGTTCTTGGGC	TGTGTTTTAACTTTTTAAATTTTGGTTC
1083	tarc0959	Other	3B	2.3	GACAGAATCAGCTTGATTGCGT	AATATGAGAACCTACGACCGCC
1084	tarc2140	Other	3B	3.2	TCATGCTGTGTTTCATACGGG	AATAAACTTTCTTCATACTGATTCTTAC
1085	tarc0955	Other	3B	6.7	TATATATAATTTTACCCTTAAAATGCCA	GGATAGTTACCGCACTGGGAAAT
1086	snp4800	CoreSet	3B	7.2	AATTATGTCTAGAGGAGATGCGAAG	AGTCATCTAAAATTGGTGAAGACAAG
1087	tarc0957	Other	3B	8.3	GTAAAAGAATAGTATGTTCATGGAACGA	CAGTGCTCGAGATACTGAGAAATAACT
1088	tarc2142	Other	3B	8.8	CTCAAACCTGTCCTGCTTTGC	TGCCCTTATTTGTTAGTAACTGCTCT
1089	snp289	Other	3B	16.0	GCAGGCATTTCAAGTTCTGC	AGCTGGAGTTCTTGCTGGGTAT
1090	tarc0962	Other	3B	16.4	TACTTAGGGCGAGACAAACTGACT	TTGCAGAGCCAACCACCG
1091	tarc0958	Other	3B	16.5	GATTCTGAAGGTAGCATCACTGATG	ATCTGAGGAAGAAGCAACCATGT
1092	snp2493	CoreSet	3B	17.1	CTCTTATATAGCAAACTTGATCACATGA	GAGCCGTAGGTAGACACCAACA
1093	tarc0968	CoreSet	3B	24.0	AATGCAGATAAACAGCTGATTAATTG	TGCTATGTAAACCTAACTCACAAAAAA
1094	tarc0939	Other	3B	40.2	ACTGAGCAAGAGATTCACCACCTA	CCTTCATTGCATATTAAATTGACTCC
1095	tarc0940	CoreSet	3B	43.3	CAACTCTAACATCACCCGGTCAT	GGGAGTACCTGTTTTGGCTGA
1096	tarc2128	Other	3B	43.9	GACCTGGTGATGCCATAATGC	CCAAGCCCTGTCTGAATTCTCA
1097	snp6632	Other	3B	52.7	CTGAATTGTATGATTTTGATGGTGAC	CAAGTCAAAGTTAGGTAAATACAAGTAAAC
1098	snp6192	CoreSet	3B	60.3	GATGTGCAAATTCACACTTGGC	TCGAACAACATCAACACTGCCT
1099	tarc0937	Other	3B	62.4	AGCATCTGGAGGACGACGAA	CAATTATCGGTTCTTCTGTAAGACATC
1100	snp347	CoreSet	3B	68.9	CGTTATGTAGACCAGTTAGACTCGG	AACCAGATGCTCCATTATCACATG
1101	tarc0970	Other	3B	70.9	AAGGCACGAGCTGATGGTAAG	TGCCATTTGGATGGGCGT
1102	tarc0961	Other	3B	117.9	AAAAATTAACCACATGCAGAAACTATT	TAATCTTGGTAACTTTAAATTTCTGTAC
1103	tarc0927	CoreSet	3B	120.6	GCCCACAGAGACCAGCAGT	AAACAAAATGGAAGGACGATGAC
1104	snp5925	CoreSet	3B	123.5	GCAACGTCGCAGGATTGATAA	AAGAGAGAGAGCTCAACTTGCTACAT
1105	tarc0960	Other	3B	126.6	GCCTTATCACTAATGTCTTGTCGTT	CAGTTTTGTTAGGAATTAATCGTCG
1106	tarc0984	Other	3B	129.4	AAGAATAATCAGAAACTATTGGACACTA	CTTGCTCACTTTTATTATTTACTACACT
1107	tarc0983	Other	3B	139.8	GCGTTTGCAGGAAGGTGG	CTACGGTAAGCATTTCTGCACTTAC
1108	snp7294	CoreSet	3B	141.1	CTGTATCAGGTGTCAAAGATGGCT	GCTCTGCTTCCTCTTCGGAAC
1109	tarc2156	Other	3B	142.7	AGGAGCTACAGGTATATTTTGTTTTCA	GAGTATTTTCGGACGGAGGGA
1110	snp5977	Other	3B	145.4	TGGTACAGTTGCTTAACAGATAACAGTAG	TGTACCTAGCATTCAGTCATTTTAGAGT
1111	tarc2572	Other	3B	166.1	GCTTCACCGGGGTCTTTG	ATTGAGAGCCCATTTCACACG
1112	tarc2144	CoreSet	3B	167.7	CTCAGTATACAAAAACGCTACAGTTCT	TAAATGTAGAGCATAGGTTATCATTCTT
1113	tarc2141	Other	3B	168.3	CTCTTGTATAAACATTTATAGTATTGGC	GCTCGATGCTACATAAATATGCTTAAA
1114	tarc2120	CoreSet	3B	172.7	CCTCCAGCCCGACACCTATT	GCTCGCCTGCATCAGTACA
1115	tarc0944	CoreSet	3B	194.3	ACAGTAGGAAGTAGCCACCTTGAAT	AGAAATTACCTGATAAGATTGCTAAAAT
1116	tarc0943	Other	3B	202.6	GCGCCTAAACCTGTCCGTC	ATCAGGACCAAAATAAATTGCACT
1117	tarc2145	Other	3B	204.3	GGACTGGGGATGGCGGCT	ACATCTAAGTCTTATATCAAATCGTCAG
1118	tarc0951	Other	3B	238.3	TTCAAATTAAAGTTGAGTACACTTTTAC	TGGTTATCATCTATTTCATTTTCCCTA
1119	tarc0982	Other	3B	245.5	CCCTCTGCTCAGCATGACTTG	TATTTCTCTGAATCTAGAGTTATTGTCG
1120	tarc0928	Other	3B	250.1	TGTGTTTGCTAATCAAACGCCT	AACCATTGAGAAGCTATCTCGAACTA

Supplemental Table 4. List of amplicon sequencing primer sets for wheat.

No.	Name	Type	Chr	Pos[Mb]	F_primer	R_primer
1121	tarc2122	CoreSet	3B	250.6	TGACCTGTGAGATCCTTTCTACTGTA	GGAATGGCATCGAAAATTGTATTAT
1122	tarc0936	Other	3B	253.5	GTAACATGAGTCATTTGATTGTTGATT	TGCCCACATCTGTAGAAGTAAAGTT
1123	tarc0973	Other	3B	253.5	GTAGAAAAGACTGTGCTTCGTAAACA	GGTTTTGTTGGTGAGTTTTCCTAA
1124	tarc0941	CoreSet	3B	256.8	GAGCCAGCTCCAGCAATGT	CATGCTTTAGGCCAAAAGAATAA
1125	tarc0974	Other	3B	269.3	GGATGGCTCTCTGCAAATTAGTA	GCTTCGTGCCCTAAGTTCTCTTT
1126	tarc0947	CoreSet	3B	284.7	CCTAATTCACCCAAAAAAAAAAGTC	GTTGTTGTACGTAGTTTTGTGCATAGT
1127	tarc0924	CoreSet	3B	305.5	TTAGAATATATGTCGTCTCAGCCACA	TTCTTCCAAGATCTACTGAGCTTTTT
1128	tarc0979	Other	3B	322.3	TGAACTGCACACGGAAACCAC	AATCTCTCTTCAAGAAGGACCCTTA
1129	tarc0972	Other	3B	327.1	GAAACAATAAGTATGGATAGTTGGAATG	CTTATTTAATGGTATACGCTGTAAAGC
1130	tarc0930	Other	3B	333.5	TAGTAATTCTTATGCCTAAGTCATCTTG	TCCACAAACTGGCTGCTACG
1131	tarc0956	Other	3B	340.8	CGGTAGAATAATTTTTAGTTGGAACAC	AATCAGCACAGGGCATTCAA
1132	tarc0953	CoreSet	3B	347.8	CCTTGTATGTAGTTCCTACATGTGATTC	ATGTCTACTGTGTCTGTGTAACACAAAT
1133	tarc0934	Other	3B	360.1	GGTGCATGTCTGCATATAGTTTCA	TGCACAGATGAAGCTGTCAAAA
1134	tarc2109	Other	3B	366.2	CCACTTGACCCGATTTTTTTGT	CAGTGGCATGTTCTTTACGGAA
1135	tarc2155	Other	3B	367.4	CGTCAGAATCATCCGAATCGTA	GACTGAAAGAGGAGGAGGCG
1136	tarc0938	CoreSet	3B	370.2	TTCAACACATAGATGATAGATGTCTTTC	TGACTATCTACAGTCATGCTTGCG
1137	tarc0969	Other	3B	370.9	AAACTCTAGTTTATTTTTCTTGCCAATA	GCTTAACTGTGGCGACAATGTA
1138	tarc0935	Other	3B	372.2	GACCACGACTTCTCCGCAG	TATTCTGTATATATTCATTTTACATTGC
1139	tarc0942	CoreSet	3B	373.9	GCATTGACTATGAGTTTTCTGGTG	CATTTGGCCGCCCCTCAT
1140	tarc0963	Other	3B	379.2	ATGGATTCGGCAATAGCTTACTAG	CAATGTTTCAACATGTGCTGGA
1141	tarc0978	CoreSet	3B	401.0	CTGGGTTAGACACCGCAAAAA	ATGGTGCTAACCAGATCAAGCA
1142	tarc2126	Other	3B	412.6	CCGATGCCACTGAAACTCAC	AGCTAATATAGCATTGTTTTTTGTAGGT
1143	snp210	CoreSet	3B	414.0	GGAAGCAAAGTTCATAAATCTACCG	CAAAGAAAAAGAAAACCATTCACTG
1144	tarc2562	Other	3B	416.1	AGTATTTCACTCACCGATTCGC	ATGGCTAAAACCTAATGTGTGGG
1145	tarc2139	Other	3B	421.2	CAGCCGGAGGTCAAGAAGTT	CCGAAGAAGGCGATGAAGG
1146	snp6814	Other	3B	431.6	CATCATAGGTTGGTAACACCATTTCT	GGTGATGCCACCTGGCTAATA
1147	snp3000	CoreSet	3B	457.0	GGAACAGGTAGATGAATGTCTGATGT	GAAATCTGGAAACATAGTTATCCAAATA
1148	tarc0975	Other	3B	469.6	CCTCCGTTCGGAATTACTCG	AGAAAATTAAATTTCAGGGATCAGTCT
1149	snp4452	CoreSet	3B	470.0	ACCCTTTCGCGGTGTCG	ATTCCATTTCCATTTGCTGCT
1150	tarc0945	Other	3B	480.0	CAGAAAATACCAATTTACTGTTCTGCT	CATAACATTATTTGGTTTGTTTGTGTT
1151	tarc2568	CoreSet	3B	499.2	GACCAAATGTGTGCAGGAGC	CGCTAAGAACTCTGCTCCATTGA
1152	tarc2583	Other	3B	505.3	GGCTTACTCCTTTCAAGAGGAAA	ATAAGATTTTCTGCACATAAACAAAGAG
1153	tarc2115	Other	3B	506.6	GCACGGAAGATTAGGAAGGG	TAATTGTTTTTATCATTGCAGGCA
1154	tarc2136	Other	3B	507.1	CCCACTGATGAAACAGGCTTTT	TCTGTTTGAGTTGTGCTGGTGTAA
1155	tarc2587	CoreSet	3B	507.3	GCAGAATAAAGTGAGACATAACATAAAA	GATTGTTCTTGGTCCATGTGTCA
1156	tarc2124	Other	3B	507.4	TTGTTGGATGTGTATCGCTCG	AGTTGGGAGAAACTACAGAGGGAG
1157	tarc0925	Other	3B	508.5	TTATTGTACTTTTTTTTACCTTGGGC	AAGCATCAGCATTCAGCATCTAA
1158	tarc2117	CoreSet	3B	514.0	CCTCTGGATCAAACCTATGATCG	AAATACTAACATGGCTACACTGCAGAT
1159	tarc2152	Other	3B	528.2	CGTCTCAACCGGGACGCC	GCCGGACATCGTAGGGCT
1160	tarc2578	Other	3B	544.1	ACTGTCCCCTTTCACTTACATTCA	GAAGATGATATATACTTTGCTGGACTAG
1161	tarc2588	CoreSet	3B	544.6	CCTAGAATAGGTAAAAGATCGGTCTAA	ATGGAACAAGTTATGATTGATCTCAG
1162	tarc0921	Other	3B	545.1	GAAACAAGCCATGATTGACTTGA	TTCTCAGGCGTCACATACTCTCTTA
1163	tarc2116	Other	3B	545.1	GCCATGCCACTAAATTCATCG	CTAAAGATTTCTGGACTTCCTATGC
1164	tarc2563	Other	3B	556.9	TCAGTGAAAGAAAAATTCAACGC	AGTTTTGAATTCGAACTCAAACAATAC
1165	tarc2564	Other	3B	556.9	CGCCCCAGAATCATTCGA	ACTGCCAGTAGAGGCGCATT
1166	tarc0977	Other	3B	559.3	CACATTAAGTCAGTGACTACCCCAA	GGTTCTTATGAATCATCAGTATAGTGGT
1167	tarc2097	CoreSet	3B	559.3	AAACAACTACTAGCAGAGTGGTGAAAG	TATGTTTTGCCGAAACTGTTGTC
1168	tarc2098	Other	3B	559.3	ACTAGATGTGCAAAACTTTGGAAGTAG	TTGCTACTGTAGATAAGGATTATTGTCT
1169	tarc2103	Other	3B	564.2	AGAAAAGACAAATGTGGTATTGAGTG	TTTATATAATGATTTTGACAGTGGCG
1170	tarc2102	Other	3B	564.8	GAGACTCAAGATCGTCGATTGTCTA	TTTATTTTCTTTGAAACGCAGAGATA
1171	snp2022	Other	3B	565.6	GTATCAGTTCGGACAAGATAAGTGTTA	GGAAACTAATGGCTGGGTAAACC
1172	tarc2580	Other	3B	565.7	TACCTGCATTAAAAAATTATTGTCATAT	TACAAACGAGCATCCAGCCTAG
1173	tarc2118	Other	3B	566.3	GTTGTAAACAGAACAAGCATCGG	ATACAAACATCTTCCCTGTCTTACTAAC
1174	tarc2132	CoreSet	3B	566.8	CCTTGTTTTTTCTGTTGTCGGTG	GTGCAAAACGCAAGCATAACTC
1175	snp7510	CoreSet	3B	572.3	CTGGCAGAAACGTCGGGA	GCTCATGGTTTTAACATGTTGC
1176	snp238	Other	3B	572.7	CATTCATCTTTTAATGGAGTTGTGAT	CATTGCAGAGCCAGTTGTTTATAA
1177	tarc2566	Other	3B	572.7	CGCTGTATCACTTTTGATAATCGT	CCCTGATGATCTGTGGATGC
1178	tarc2579	CoreSet	3B	576.4	GACAAAGTCGATGTGCATTGTTG	TTTCTTGCCATAGTTCTGAATATCAA
1179	tarc2129	Other	3B	576.5	CTATTTCACCATTTGGCTTTGAC	TACTTAAAGAATATTACCATTTCCACTC
1180	tarc2569	Other	3B	578.4	GAAGACCGTAATGAGAAAAGACTAGTG	TCTTCACTGTCGCTTCTTCTTACC
1181	tarc2111	Other	3B	585.8	ATGCTATAACAATTAGCCATTTCAGAT	GACAAATCATCACAAGACAACAAGG
1182	tarc2575	CoreSet	3B	586.5	TCTAGGATCAAAGGTAAAAATTGTTAAT	CATAGTGTCAAATTAGTTTCCAAGGTA
1183	snp4218	Other	3B	586.7	CATGGAGGTTACAGACATGAGCT	TACTAGTGACCTATTTAACCTGATCCTTA
1184	tarc2133	Other	3B	586.7	AAAAGAAACGGATTCCTAAAAAATG	GGGAGTGAGGGAGTCTGGC
1185	tarc0932	Other	3B	588.5	CCTCAGCGTTATGGAATCACAG	CACACAATATATATAGTCACAGAGCCAG
1186	tarc2148	Other	3B	591.4	TCCATGGCTGAACTGTGATGTAA	GCCTGGATCCTTAGCTTCTCTG
1187	snp5711	CoreSet	3B	591.5	CAAAATATTACTTACAGAAGGTGCAGAT	GTTTATATTGTAATTTGTAGCTGGGAA
1188	tarc2565	CoreSet	3B	591.5	GCATGATATAGCAATAGCATCAAAC	ATAAGTCAGATGGAGCTGCTGATT
1189	tarc2123	Other	3B	592.7	GCCCCTAGCCCTAAATCTAAAT	GGAATAGGCTCCCGCTTAGTC
1190	tarc2581	Other	3B	594.4	CACACGATTTTTTCTACCCGAC	TGGTACAAAAGAAGTTCATCTGCTG

Supplemental Table 4. List of amplicon sequencing primer sets for wheat.

No.	Name	Type	Chr	Pos[Mb]	F_primer	R_primer
1191	tarc2138	Other	3B	597.5	ATGTGAAAAATGTGAGGAAGAAAATA	AAGGAGAGTGAAATGTTCGACTGT
1192	tarc0971	CoreSet	3B	617.7	AAACCTCGAAACCATCTGCG	AGCCTCCGACCTGCCCTC
1193	tarc2105	Other	3B	622.4	CAATCTGGGTGTGACACTTATGAA	TCCTTTTGGCATTTACACATCG
1194	tarc2110	Other	3B	623.8	CAGGCAACCTCTGTTATTATTTTTT	TCCTACACATGAAGTCAAATGTTATACC
1195	tarc2137	Other	3B	648.7	CTCATGGACTACTCACCCATTGTT	GAATTGGTATGATGTCTATCCTATTTTG
1196	tarc0929	CoreSet	3B	651.3	TGGGTGAGTGATGGTGAATGTC	TCGCAATCTCCCTGTCACCT
1197	tarc0946	Other	3B	657.6	AGGTCTTGAACATTGCCATCG	AGCTCACTAGTAGTACATTCTAATCTCC
1198	tarc2104	Other	3B	663.1	GGAATAGTTATTTCCTGACACGAGC	AACGCATTTCCTTCAAAGACTG
1199	snp3244	CoreSet	3B	665.3	CCAGGATCATAGCAGAATATGGTC	GAAACCAAGTTGCAGTGTTGTAGTA
1200	tarc2121	Other	3B	666.8	GGATGTTACTGGATCTAACATCTCTGT	GTATGATTACGCATATCCATCATACTAC
1201	tarc2100	CoreSet	3B	669.3	TATGCACAAGGACCTGAACGAT	GGTAGTCCCATATTCAGTTGGTCA
1202	tarc2151	Other	3B	671.7	ATCACCTTAAGATTAAAGATAAAGAGGA	CTGAACTTCACTTCTTTTAGGTTAGAGA
1203	tarc2154	Other	3B	671.8	AAGGAAATGTTCAGTTAGTTTAAGTAAT	CCTTTTTCTCAGTAAGCCTATCTTTGT
1204	snp4841	Other	3B	674.4	GAGCTACCAGTGTAGCAACCGTAG	CGTTGGACATACTGACATATAGTGGG
1205	tarc0933	CoreSet	3B	679.2	ATTCTCGGTATTCGACTCCTTGA	GAGACTGGGCAATCTGGCG
1206	tarc2157	Other	3B	681.1	GCTAATGCAACCCAAGTCAGTG	GAAGTTGCTGGAAAATGGAGAG
1207	tarc2099	Other	3B	686.7	TTCGGTCACAGACTCACTCCC	ATGTGATGTGAATCTATTAATTCGTAGA
1208	tarc0966	Other	3B	692.2	TCCCATCCATCAGATCAGATAAGA	TTTGTTCATCCAGCCATCCA
1209	tarc2150	CoreSet	3B	693.1	CCGTCACTCTTTCGCCCAC	TTTATGTTATTGCGGCATGTTGT
1210	tarc0923	Other	3B	694.0	GCACATAGAGTTGGAAATAACAAATC	GTTCATGTTGTGGACAGAGGCT
1211	tarc2146	Other	3B	695.8	CCACCTGCATGAAACATCAGT	CTTGGGCACCACCATTAGC
1212	tarc2147	Other	3B	695.8	CCCTTCCAGTTGGTGCTTATAGTC	GAGTTACGCAACCGTTTGGC
1213	tarc2149	Other	3B	696.7	CCCCATCCCCAGTTTTGTTT	TATTTGATCGTGATTTGTCTTTCG
1214	tarc0926	Other	3B	701.4	GGGAGGGACGACAGCGAG	GTAAACTGCAAGTTCGACCACC
1215	tarc2125	Other	3B	701.4	TTTATCCAATGAAAATGGAAAAGAA	ATACGCAATTAGTAGAAGGGAAACTT
1216	tarc2113	Other	3B	705.7	GTTTCTATATTGGTTCGCATACTCCT	GTTTTAGATAGCTTCCAATTTCTTCTC
1217	tarc2114	Other	3B	705.7	CTGTGCCTATAGCCTGACGCT	AAAAATCACCCTGCAAAATCG
1218	tarc0922	Other	3B	708.9	CTGCACTGGCTTTGTCTCACC	AAGTGACTCAAAGAATGATGTCGG
1219	tarc2135	Other	3B	709.0	GCAAAAATGTTAGTGCCTGTTAGAA	CCAGGAAGGATGATGCGG
1220	snp2063	Other	3B	719.1	CTGAAGGGACTGCAGAAGATCA	ATAGGGAGCAACACAATGATATGT
1221	tarc2108	Other	3B	723.2	CCCGTGGGAGCAGATCAG	GGCGTTCCAGTCGAGCAC
1222	tarc2577	CoreSet	3B	723.4	GCGGTTCAGTTCATCTCATCAC	CGAATGAACAGTTGTCGAAGCC
1223	tarc2127	Other	3B	724.0	CCTTTCCTTCATCGTACCATCAT	TGAGTAGCTTACATAGCAAGAATTCCTA
1224	tarc2130	Other	3B	724.7	GCATGAGGTGAGGTATCGGA	ATTCAAGCAGGGGGCAGC
1225	tarc0965	Other	3B	724.9	CAAATAGATCAAAATCCATGTCACG	CATGAGCTTGAGGTAATGATACACTTA
1226	tarc2119	Other	3B	724.9	GACAGGTATCTGCCAATAGCTAGTCT	AGTAATGTGAATTGTTGATTTTTTTTCT
1227	tarc2567	Other	3B	724.9	GCAGAAGTCCCATGCCATTG	CAAACCAACAAAAGCTGCCAG
1228	tarc0976	CoreSet	3B	725.4	CAATCATATTGAGCAATTCAGTTCAG	GTTGAGGTCACTGGTAAGGGAA
1229	tarc2112	Other	3B	725.4	TGCCAGCGACTCGAAGCA	GATGACGGTCTCTCAGCGATAG
1230	tarc2576	Other	3B	725.7	GTGGAGATGTTCAAGGAAGTGCT	AAACTGTTACATGGCTGGACAAAT
1231	tarc2585	Other	3B	726.4	GGTCACTTGGGAAAAAGAAGAGG	CAGATTTTTGTCTGGATATCTCGG
1232	tarc2101	Other	3B	728.9	ATTATGTAACGGGAGACCTCAAGA	GGTGAGTAGAAAACCAGGGCAA
1233	tarc0981	Other	3B	729.2	AATTGTCTAAGTGGAATAAATGGAAAGT	GTCTTGAAAATGGATATTGTGTCTTTAT
1234	tarc2584	Other	3B	729.2	CTATGCAGACATTGTCATATCTTACCT	TTCTCTTAAATAGCCTTCACCGC
1235	tarc0980	Other	3B	731.3	GACTGGCTTTCGGCCTTTCT	GACGATTCAACTTATTCTACACACTTCT
1236	tarc2586	Other	3B	732.4	ATTATGTCTTGCATCCCAGTATGAA	CTTGTACTGTCTGCGATGAATAGAA
1237	snp2360	CoreSet	3B	734.4	TGTTATCATGCAACTTCAACTTCTGTA	GGTATGTATGCGTTGTCTACTTATGGTA
1238	snp3667	Other	3B	737.8	ACAGCTGCATTCCTGCACATC	TTTCGAGTCGAGGGCACAA
1239	snp5014	CoreSet	3B	739.1	ACGTCCAATACATTCTCTGTGGC	CAGTAAACTTCATGCCGCCA
1240	snp2462	Other	3B	739.2	GCTCGACAAGGAGGTTCGG	GTCTCAAAGCAGAAATTGCTTCA
1241	tarc2134	CoreSet	3B	750.1	ACTTTCCAAGAGAGGTATGCCC	GAAGGACAGGAACACAGACGC
1242	tarc2573	Other	3B	763.1	GTATGTCTTGCAGTTGGAATATGTACTT	ATACAGGTTATTAAGGAAAGCACAAGT
1243	tarc0967	Other	3B	767.0	CTCTTTGCTGGCACTCTGTCC	CATGAGTTTCAACCCTCTGATCA
1244	tarc2106	Other	3B	775.5	ACAACGTGATACAAGAAAGCCAA	TGATGAGGTCTGCCCACTATATTT
1245	tarc2574	Other	3B	775.8	GATGATTGCACCTTTTATCATTGC	ACATCGTTAGAACAAAAATCAATCCT
1246	tarc0964	CoreSet	3B	779.6	CAGGTCGGTACATTTTAGAAATCATT	TTTGACACTTACACAGACCTCTTGAA
1247	tarc2096	Other	3B	783.5	GCCAGCATATAATCCAAAGACAAA	CATCAAGCCCAGCGTTCA
1248	tarc0931	CoreSet	3B	784.7	GCATTGCTATGGACATAAATTCTACA	TAACACTTATCTGCGTATTATCTCTTCT
1249	tarc2570	Other	3B	802.8	GCTGATGTTAGATTGCAGGAGTGT	TCAGCGCAATAAAATGACTGTGTA
1250	tarc2571	Other	3B	802.8	AGGTCTTAAAATTGTTTTTTATCATAGC	TGAATCATGCACTTTTGCTGTGT
1251	tarc2107	Other	3B	806.2	AGTTAGGACATCACTGACACCTTGA	AAGACTCCCTCTGCACACGC
1252	tarc2143	Other	3B	807.3	GAGCAAGTTTCTTCAAACACCG	GAGTTCAACTTCATCGAGTCCATAGT
1253	tarc0985	Other	3B	810.5	GGAATGTGTGTGCAACTTTACTTTCT	CATGCAAGTATTCCATGCAAATAA
1254	snp2148	Other	3B	817.8	CGTGTGCTGCCTGAAGACTAAAC	GACAGAAAATTAAAAGGGCTAACAGT
1255	snp2147	CoreSet	3B	817.8	GTGTGCTGCCTGAAGACTAAACC	ACAGAAAATTAAAAGGGCTAACAGTT
1256	tarc2158	CoreSet	3B	817.8	TGTGCTGCCTGAAGACTAAACC	TTCTCTTTTCCCTCTCTCCCTCT
1257	tarc0954	Other	3B	818.8	GGTCTCCATTTCACTCTAACATTTTTT	GCTGGGCTCTTTTCTTTTTCAA
1258	tarc2153	Other	3B	818.8	CATTGCGAATATGGGAATGTGA	CACCGGATGTGGCTTCTTG
1259	snp1756	Other	3B	822.2	GCTGCTGGTCTTTCAACATATACTTAT	TGCGCAACTTCGAACATGAT
1260	tarc0949	CoreSet	3B	822.2	CGCTTCTGCCGTGCTTGT	ACGGGAAAGGCGAGCACA

Supplemental Table 4. List of amplicon sequencing primer sets for wheat.

No.	Name	Type	Chr	Pos[Mb]	F_primer	R_primer
1261	tarc0950	Other	3B	822.2	TGTAGCCAATTTTATTTCTAAACATAAT	GGTTTCCAATGTGCACTCCA
1262	Inf23510	Other	3D	0.8	CCATGGAAGATGTGCAGCCTA	TTTGTACATGACATACATAAATTACATG
1263	Inf42979	Other	3D	1.3	TACCCCTCGCAGTACACACAAC	GAAGATGGATGAGTGAAGAGTTGG
1264	Inf11113	Other	3D	2.6	GATATTCCAAGTAATGACATTCCCC	GGTTAGACCTGGTCAGGTGGAAC
1265	Inf48159	Other	3D	3.2	CTGCTTGAAAGAGCGGCC	CTCTTGGAAAAGTATCGGTAACTCC
1266	tarc0212	CoreSet	3D	5.0	ATGTAGAGGCACTAGTTGGCATTC	ACACATAGTAAGTTGCACCGCTG
1267	Inf16020	Other	3D	6.3	GATAGTAAGATACTGTAGGCACCAACA	AAAGCTTCATTATTTTCTGGGTTTAG
1268	Inf18637	Other	3D	7.3	CTATATTAGGCACATCATGGGGAT	GGAGTAATTTTTAAAAAGATGTCTACGA
1269	tarc1308	CoreSet	3D	11.8	ATGAATAGTCTGATGTGAGGAAACG	TTCCATGTCTAGAAGAACTTGTCGT
1270	Inf15686	Other	3D	14.4	TTGCTGAAGTTTTTTCTAACAGGAA	AGATCCCAACAGCATTCGTAATT
1271	tarc1450	Other	3D	17.7	CATCTCCTCTCTCATACACATATCTCAA	TGGGGTAGGAGTACCTTCAGTTCT
1272	Inf11832	Other	3D	22.0	GTGCATGGGTCGAGAGCG	CAGACCACGATGCAGGAGC
1273	tarc1306	Other	3D	23.1	AACTCATACTCCCTCCGTCCG	TTTCATTTCAAACTGTTCCTTTCG
1274	Inf16507	Other	3D	23.1	GGCTTCTGATGATCGTGGTTC	GCGGCGATCTAATGGACTTAT
1275	tarc0221	CoreSet	3D	27.9	CTACAGAGACGAACCGCTTCTATAT	CAAAGAAAAGATGAGAAAGATGACTACT
1276	tarc0226	Other	3D	28.3	GCTGTCATTTCCAAAGAAACGA	TTTTGCTAGACTACAACTTCAATCCTA
1277	tarc0216	CoreSet	3D	30.2	GTGGGACATTCAAAAACACCTAGA	AGGTATGTTTTGTCCGTTCAGAAA
1278	tarc0224	Other	3D	30.3	AAAATATTGCTTTTGGCAGGTGT	TGCAATAAGTCATCCTTATTGACAG
1279	tarc0217	CoreSet	3D	31.7	GACTTGCTGAATTTGCTGGTT	GTACCCAAACTTGGAAAGGTCC
1280	tarc0218	Other	3D	31.7	CTAATCATTTCTATTTGGTGCTGGA	ATTCCATTCCAGAACTACTTGACG
1281	tarc1315	Other	3D	33.2	CCTAAATTTTATTCCCTATACGATACCT	CACGCAGCACACACACATACA
1282	tarc1508	Other	3D	44.0	ACCATCAATTGCAGCCACTG	TGATCTTCACAGTGGAAAAAGTAGG
1283	tarc1483	CoreSet	3D	45.0	CCAATAAATAATTCCCAGGATCATACT	TTTCCAGCTTGGTTCTTGTTGT
1284	tarc1535	Other	3D	45.4	TGCTATAACAGAATGTGTGATGGCT	TACGAAGACTGTGGAGGTCAACTAC
1285	tarc2429	Other	3D	46.1	ACATTACTTCATCTTCTGCTAAGTGCTA	GCATGAACATTAGCCCCAGC
1286	tarc0223	Other	3D	57.2	CAGACAACATTTTCTGAATATGCTTAA	TTGCTGGCCCGATATTTAATG
1287	tarc0228	CoreSet	3D	58.1	TGTGACTCAGCAAAACTGACAGC	ATTGTTGAAAGCCATCTTGTTCA
1288	tarc2426	Other	3D	72.0	CACATGGAAGGGTGTAAATAAAAA	GAGCTAAAACACTGCTACAGACAGAT
1289	tarc0214	CoreSet	3D	74.4	ATAAGTAAATCTGGTTATACCCTTTTTC	CAACCCATATAAAGACCTTATCCGT
1290	snp7672	CoreSet	3D	80.9	GATTTGATGGAATTTCAGAAAATAAT	AGGAGAGAAATACTGTAAGTTAGATCTGAT
1291	snp6119	Other	3D	84.6	GTAAAGGTGTCCACCGCTGA	CACAATAGAAAACTAGGGTTGGCA
1292	tarc0220	Other	3D	87.9	TTTGTTCTTCAATGACCTGAGGTTA	AAAAGTTTTGACCTTGTTACATTAATTA
1293	Inf35063	Other	3D	88.1	CCAAGTCAAATTCATCTCCATCAG	GGGATGTTAAAGGATGGACTGTTG
1294	tarc2427	CoreSet	3D	120.6	GATGCTCAAAGCATCTAAAAGATCA	CTCAGGTCACCGCCCGAC
1295	tarc1309	Other	3D	130.5	TTAATATAGAACAAAACAAATAATCTGA	CATCCATCAACTTTTACTTCACACG
1296	tarc2428	CoreSet	3D	130.5	TTAATATAGAACAAAACAAATAATCTGA	CATCCATCAACTTTTACTTCACACG
1297	tarc0227	Other	3D	130.9	AGCCTGCAACCAGAATGACAG	TGTGAAACAATAGTTCCACTCGC
1298	tarc1484	Other	3D	144.3	TTGATTACAATTCATGACACAGGC	ACGTTAGTGTATATGTTCCATTTGTTTC
1299	tarc0219	CoreSet	3D	158.1	TGGCAAATAAATACAACATCCAGG	CGTATGTCAATTTAACACTGTACATTGA
1300	tarc1536	Other	3D	175.5	TTCGTATGTCATTATTTATGCCCTT	ACTTTGTATATCTGGAAACCTTAGCC
1301	Inf15270	Other	3D	179.2	CCCACTAGTTGATTCACAGCAAA	GATTGTTGTTTTGGTGGGGGT
1302	tarc2425	CoreSet	3D	181.7	TCTGATGCGTCATCTAAGGTGG	CACACATAGCAACACACACAGAAAACTC
1303	tarc0215	Other	3D	186.6	TTGTTGGTTAGTATTGTCCCCTGTA	TTTTCATAATCTAACTGAATTTTCCGT
1304	tarc0222	CoreSet	3D	189.5	TGGTAGTTATTTCAACACCATTATGAAC	TCAGCATATACAGGATCACTGAACC
1305	tarc0213	Other	3D	190.3	GGTATCTTTTTCCCTGAACTTCTTATAT	ACTTGCTGACCCGTTGCTAAA
1306	tarc0225	Other	3D	238.6	TCTATGTGCTCGCATACGTGG	ATGCTCCTGAAATTTGTAATGCC
1307	tarc1302	Other	3D	263.3	TTCTGGAAGCAATGGACTGTATG	GCTACATGGGCTTCTTAGCCTG
1308	tarc1299	Other	3D	266.6	GAAGGTCAACTTTATTTTGTACTCTCC	ATATTCATCAAATATGAACTTTAAAAAC
1309	tarc0191	Other	3D	268.1	CGTATTTCAAGTTAAATAATTGGAACAA	GTGGGTGCTGTTGTCAGTTCAG
1310	tarc0188	CoreSet	3D	273.5	CTATTCAATTCAGCGAGGTCACTC	AAGTCTTCTTCAGAACTGTCCTACCT
1311	tarc2415	Other	3D	274.5	CCTCCCAGGAGGGAGGTATG	ATGTTTGGTTACAAAGAAGAAGGGT
1312	tarc2404	CoreSet	3D	276.5	TATGCTTTACAGATTAATATTTGGTGTG	CCTGTCAGTAGTTCAATGGCG
1313	tarc0210	Other	3D	290.9	GTTCCTAGTATAGCAGCAGCTCTAATG	CCAAATCTGACAAGAGTTAATTGTCC
1314	tarc1443	Other	3D	313.3	TCATATCAAAATATCCAAAACATCTTTT	GACAAATATTCTACGGCGTATATGG
1315	tarc0174	Other	3D	313.6	AACCTCTCTTAAACTCCACTTACCTG	AAGCTGTGGTTCTTCTTGGCA
1316	tarc0185	Other	3D	314.6	GGAACATGCCATTATGCCATA	TTGTCATGTGAACTTCATCTTAACATT
1317	tarc2412	Other	3D	329.4	AGCCGCAAAGATGAAGCAAC	CTCAGGGGTCAGACGTACGAT
1318	tarc2413	CoreSet	3D	329.4	CATTTGGATGGAGATGCAGGT	TGGTAAGCTAAAGTTGCAGATTTACA
1319	tarc2410	CoreSet	3D	330.3	TTTCCTTTACCTACAAGCATAACAGA	TTTTCTAGGTAATGGATGCCTTCA
1320	tarc0179	Other	3D	332.5	AATGATCGAGTTTTCTTCATTGTCA	TCCAAATATCTGATGCAAGTAGTACG
1321	tarc2411	Other	3D	332.6	CAAATCTCTTACAGTAGCATATTACAAA	AGGGAGCAGGACATAATCAGGTA
1322	tarc2423	Other	3D	336.6	GCCGAGTTCCGCTGCATC	AAATCGCATTGAACATCTCTCTCT
1323	tarc2424	CoreSet	3D	336.6	CTGTTCATCATAGTACTGTGGCTGT	TCGAGAGATCTAGTCTCTTCCAAGG
1324	tarc0200	Other	3D	337.5	TTAGAAATTGCACCCTTTTTCAGT	AAATATTGTGCACTTATCCTTGTGTT
1325	tarc0175	Other	3D	339.3	TGTTGACAGAACAGTTGATAGAGACC	TGCTCCACTGGCTTTTGAGA
1326	snp7468	Other	3D	340.7	TGTGGACAATCATTAAGTTTTTTCTTC	GAGCAAAAATCCTAAACCGCA
1327	tarc0178	Other	3D	351.1	TTTCACTCTTTATTCAAAAGTCCATAATAC	TCTACAGTCGTGATGCCTCCC
1328	tarc0190	Other	3D	353.0	CCAACGAATTGATTCGAGTTATGAT	TCTACGAGCGATGTATGCCGT
1329	tarc1510	CoreSet	3D	354.6	AATCTTAAGCAGCATTTCTACACTACAT	TCTTCTGTAGGGACTGTGTTATGGA
1330	tarc0187	Other	3D	356.9	TGTGCCTTCTGGTCCGAGAG	CCAAAAAAACTCCAAAGCAACGA

Supplemental Table 4. List of amplicon sequencing primer sets for wheat.

No.	Name	Type	Chr	Pos[Mb]	F_primer	R_primer
1331	tarc2408	CoreSet	3D	369.0	GTTCTTTTCAGTTTTCACCTCATCA	ACTCTGGTATCATTTTGGTACGTTCT
1332	tarc0195	Other	3D	381.6	ACATTCCATACCAACTTAATACTTATTTG	CTTAGTAAATAAACAACTCGCTGGG
1333	tarc2419	CoreSet	3D	382.5	CCAAGTGTGCAGAATACTGAGAAA	CCAAAGAGTGAATTAGTGTTGAGGAT
1334	tarc0209	Other	3D	386.4	GGTGATTGTATGCACCACTTCC	CTGCTATTAATGGCCTTTCTGC
1335	tarc2414	Other	3D	386.7	TGAGGCGACAAGTTGAGGGT	CCGCTCACTAAATATCGATGGA
1336	tarc1329	Other	3D	401.9	ACTCTCTGGTACACGTTGACTGGA	TATAAATCTGAAGCCTAGAGAGTAGAAT
1337	tarc2400	Other	3D	405.8	ACGGATAATATCCCTGAAAGGAG	TATTCAGAATATCTACTCTTCTTGAACA
1338	tarc0184	Other	3D	407.2	ATTAAGTTGACTATGTTTCATGGACG	CAAATCGTGGCTCTTGGAAAA
1339	Inf24347	Other	3D	412.1	CAGTTCAGTCAGAAGCCCAGTATA	ATATGGGTGAAGTCCTAAGCTATACG
1340	tarc1336	CoreSet	3D	422.7	GCTAGTTATGCTTCTTTCAGTTCAAA	TTGTGCAAATGCTACAAAGAGAAA
1341	tarc2402	CoreSet	3D	424.7	AGTCGATTAGTAACTTCCAGAAAGAAAT	ATTGGAGAGAGTTATACAGCAGTCAA
1342	tarc1294	Other	3D	427.4	ACATTGTCAAAATTTACAAAGCTCG	GACCAACCGAAACAATAGGCA
1343	tarc0194	Other	3D	427.8	GACGAGCTAATATTCCATTCATCG	GGGTGTCTCCCCTTAGTCCG
1344	tarc1358	CoreSet	3D	433.3	GCCCCAGCAGAGAAGTTCAGT	TGTCAATAAATATTTTGATGCCCTACT
1345	tarc0171	Other	3D	433.4	TCATTTTATGACTTCATATAGAGATGGAA	TGCCTTACCTCAAAGTCATGGTAT
1346	tarc1451	CoreSet	3D	449.7	GGAGTTTTATGTTTTTGTGCTTATTTAA	TGCTGGAATACTGGATTCCTGTT
1347	tarc0183	Other	3D	449.8	TAATTCTGTTCACTACTGCTTATGTACTGT	AAATGTCTGAAAAGGCATATCTATTAA
1348	tarc0201	CoreSet	3D	450.9	TATTGGATATCTCAAACTTAGCTTACC	CAACTGTAACACGGTACTGAAATGG
1349	tarc1278	Other	3D	460.4	GCTATGTTTGGTCCACTATCTGG	CCTACTCACTTGAGTATCGTATGCG
1350	tarc0193	Other	3D	468.8	TCTGCTCCTTTGAGAATAGCCTG	CTGAAGTGTTTGAATGAGTTCCTGA
1351	tarc0211	CoreSet	3D	473.4	AACAGCAAAGCTATTATCGAGATCTA	CATGGCACCGGGATAGCAA
1352	Inf17604	Other	3D	480.4	CACCAAGCGATTCTTCTACAGC	TTTAGATCACTACTTAGTGATCTAATGG
1353	tarc0196	Other	3D	502.9	TTGTTATTTTGTCGTACTCAATCGA	TGGAGTATTTCTCAACTGATGACCT
1354	tarc0202	CoreSet	3D	503.9	AGACCTCAAGCTCCCCACTGT	GGAAAGTCTTCGGCGTGTTG
1355	tarc1480	Other	3D	504.1	GCCAGTAAAGCGTCAGTGTTTCTAT	CAACTGAGCCTGCTTCAGCTATC
1356	tarc0208	Other	3D	511.3	ACAATCTGACCAACTGCTCCTG	GTGGTGCAGTGGGTCCGT
1357	tarc0203	CoreSet	3D	514.7	TGACAGCACCAACTAGGCAACTA	CGGGTGAGACTGTGTTTTCGA
1358	tarc2405	Other	3D	518.2	GCTCCCACTCCAGCCCTG	TGTTCGGTGCCAACCCCAC
1359	tarc0189	Other	3D	519.3	CCCAGTTCGACAACTTCAACAAG	GCAGCTACTTTGTCCAGTCTCTTG
1360	tarc2407	Other	3D	527.0	CATGCAAATTTCATAAAATCTTATCAA	GGACTATGGATGGCTAGTGTGGA
1361	Inf63677	Other	3D	528.6	ACAATCAAAATACAGCAGGAACGT	TTATCATATAGAGAGCCAATTAAAAATA
1362	tarc0206	Other	3D	532.7	TATTCAGGTAGGCCGTCTAAAATG	GATGCTGCATCACCGAGGT
1363	tarc0207	Other	3D	536.4	GACGTGACCATGTGATTCCAA	AGTAAATGCTAGGCTGAGACATAAATC
1364	tarc0199	CoreSet	3D	543.9	AAGGGGAGAAATACAGCAGCA	GCAAACTGAATTTCACATTCCTAATAT
1365	tarc2409	Other	3D	546.2	CCAATTTATCATACATCTTCTTATGGTC	ATAAATGTTGTGTACCGATCATTGTATA
1366	tarc2418	CoreSet	3D	547.3	CTCAGCCCTCCCTCTTTCCT	ACCGCACACCACCACCAG
1367	tarc2406	Other	3D	549.5	CTTCAGTTCCATCTAATGCTGACTAA	CATTTAAAATTCTTGCGCTTGTG
1368	tarc0177	CoreSet	3D	553.0	TTCCATTTCCTCCTTACAGCG	TCTCCAAAAATGAGGATCACATG
1369	Inf59538	Other	3D	562.7	GCACCACGCACGCAACTT	TTTGTCAGTCATGCGCGC
1370	tarc0181	CoreSet	3D	581.7	TTCTATTTTGGTCTGTCCAGATCATAT	CACAAGTGACAAAAAGTATCAAAACA
1371	tarc0204	CoreSet	3D	585.7	ACCTCTGGTGTGGCATAGTTTAAA	AATTAAATTGTCCTTTGAAACATAAAGATA
1372	tarc0176	Other	3D	590.9	TTTCAGAATGAAACAAACAGGAGG	TCCAACCCTTGAGAAAAATCTAACT
1373	tarc2403	Other	3D	590.9	ACTTCGTCCTGGAGAACCTCAT	TCCCAAAATGGTTAATCCATACC
1374	Inf6798	Other	3D	596.9	GTCAAATTTGGTTGTAACAGGATG	CCCATAATTGCACTTCAAAATTAAG
1375	tarc0198	Other	3D	600.1	CATAAGCAAAAACTAGTCAGGCGA	TTAACTCTCCCATAACTTTAGAAACACA
1376	tarc0172	Other	3D	601.9	TGTGTTCCTGCTCCTTCAAGG	GTTGAAGTTCAGATTTACGACTTGAG
1377	tarc0180	CoreSet	3D	601.9	AACAATCTGGTCGTTAGCTTAGACA	GCCACCAGGATTGGATCAA
1378	tarc0186	Other	3D	602.3	TCAGTGTCAGATGCTTCTATGCC	TCATTCTTTGCATCATCAACATCTA
1379	tarc0182	Other	3D	603.4	GTTTATTTTGCCTTGACCATTCTT	TTGGAGGTTGAACACGACGA
1380	tarc0173	Other	3D	604.4	CTCAGGAGGATAAACTGATGAATAATT	TTCAACCTAATCTCCATACCTTTCC
1381	tarc1419	CoreSet	3D	604.4	AGGAGAATGAGCCGCCGC	TTATGTTCTAGCTTGAAATTTTACTCGT
1382	tarc0192	Other	3D	604.5	CATATATCAACATGTACACCTCAATAAA	CGGGTGTGTGTACCTCATGCT
1383	tarc1269	Other	3D	604.7	CTGTCCTGATTTTGAGATTCTGTTACT	TCCCTATGCAGCACTGAGCC
1384	tarc0197	Other	3D	604.9	GTACCAACACTGGCTATACATCTCG	ATGAAAGAATGTTACAATGGATAAACC
1385	Inf19228	Other	3D	606.3	CCTCTAGGTACCAGTTTCTCTCTTCTAA	ACTAATGGCTGAACTTTTGATCTTCTA
1386	tarc2416	Other	3D	606.9	GCTCTTCCCTTTTACAAAAGAACA	GGCCACAGAACTTAAACCTACGA
1387	tarc0205	Other	3D	609.2	CCTTCAGCACATAATCACAGTCTTAT	CTTGAACGAACCTTAGATGAATACG
1388	tarc2420	CoreSet	3D	609.6	CAGGGGTTCGAAGTGCTCA	CCACAACATCACAAACTCATGTTAT
1389	tarc2401	Other	3D	609.7	TGCAAGGAATACAGAGTATTAAGTAATT	GCTGTTTGATATGTGTCCTGCC
1390	snp3177	Other	3D	610.3	GCACTGACATTTATTTGCGCT	ACAAAATCTGTTAAGGACCCCG
1391	snp6725	Other	3D	611.3	CACGGGATGTAACTGCATTCA	CGCACAGGCCCTTTTGCT
1392	snp4683	CoreSet	3D	611.3	GCACAAATGGTTTTTCGTTTCA	AAAATGTATCAATACAACAAGAATATGGTAT
1393	tarc2417	Other	3D	612.3	CAGTCTTCTCAAGGCCTAAATTCTC	CATCAGGTTTTCTCTCAAAGATGG
1394	snp7274	Other	3D	612.9	CAGGTCCCCTTCTATAGGTTGTCT	CGCTTACTCACATTATGAGCTTCA
1395	snp643	CoreSet	3D	613.4	AATGCATGCACTTGGTCGGT	TCGACGTCGGACGTGTCC
1396	tarc2421	Other	3D	613.4	ATGAGTTTGTATTTTTGATTATTAGTTT	AGGGTATGGAAAAGTATACAGGGG
1397	snp4081	Other	3D	613.7	GCTGTTGTCGTTGAACAAATCG	ATGTACAGAGTTGATGACAGGCC
1398	snp7526	Other	3D	613.7	GTATTCCTTGATAGTCTGGTATCTGATAC	CCATATGACATTATTAAAGTGTCATTGC
1399	Inf50460	Other	3D	613.7	CTGTTGTCGTTGAACAAATCGA	TACTGGAATTTACAGGGATGCG
1400	tarc2422	Other	3D	614.2	AAATGCATAAATTTCATTGAAAAAAA	AATTATAGGAGCATGCATGAGACG

Supplemental Table 4. List of amplicon sequencing primer sets for wheat.

No.	Name	Type	Chr	Pos[Mb]	F_primer	R_primer
1401	tarc0571	CoreSet	4A	2.9	CCTCCTCCTCGTCCTCCG	GTACAAACCAAGGCCGAACA
1402	tarc1804	Other	4A	3.1	ATTGCATGCGGGTTAACCTTA	GGTTCTTTGTCCTAAGCTATTACGAT
1403	tarc0562	CoreSet	4A	3.2	TCCTGGGCCTTGAGTTCCT	CCCTCGCAATCTGCTGAGA
1404	snp6137	CoreSet	4A	3.4	GCAGCCGAGACGACAAAGTA	TCAACATTGAAGGGCTTGCA
1405	tarc1814	Other	4A	3.9	TTGGTATTTTTTTCTCTAAAGGTGGA	GAGGAATGAATGCAGATGATGGT
1406	tarc0581	Other	4A	4.2	GGGTTCGTGGAGAGCGTG	GGCGATCCAGTTGCCCTTAC
1407	tarc1805	CoreSet	4A	4.6	CCTTGAGTTTCCTGTCAATACGA	CAGCCTCTTCAAAGAATTTATAACAA
1408	tarc1813	Other	4A	5.2	CCTTTAACACGCAGCAGCATAA	TGCAGTGAAGTAGCAGGGACTG
1409	tarc1794	Other	4A	7.6	TAGAAATGAATTTGGTTCACACACTC	AGAACATGCCGCCATCCG
1410	tarc0622	Other	4A	11.4	TTGATGCCTTGAAATCACGGT	TGGTTATTGCTCAGATGGTGACA
1411	snp7632	Other	4A	12.7	TCTGTACAACATATTCTGGTCATATTGAC	AAGGTCATCTTGATGCTCTTCTTG
1412	tarc0618	CoreSet	4A	17.3	TCATTTGGTCAATTTGTTTGGATAA	TGCATATGAGCAACCTCCTTTTT
1413	tarc0557	Other	4A	24.9	TCACAACCACTAAGATCAAAACCAC	TTAGAAATCCTGAAGAATCTTGCCT
1414	tarc1792	Other	4A	28.2	TGATCTCAGCAACCTCATCAACAT	ATCGAGAAGGTACCAGTAATTTCTATTA
1415	tarc0625	Other	4A	28.3	CATTTGCTTCCTGACTCCACCTA	GGATGATCTACTGTCTACAATCTGCTAT
1416	tarc0585	CoreSet	4A	29.0	AGGACAGTTCTAGCAAGCCTCTG	AATTTCATGATTTAAGACAAACGACA
1417	tarc0559	Other	4A	31.4	GCAGAAAGAGTGGAGAGGCATG	TCTTCCATATTCAGGAATCTCACTG
1418	tarc1808	CoreSet	4A	35.6	CAATAGTGCCAGAAGCATGAAAA	GACGATGATGTTGTGCCATCAG
1419	tarc0572	Other	4A	37.3	CGGATCCTCAAATGGCAGG	GCTCAAATTCAGCTCTGGATGT
1420	tarc0555	Other	4A	37.8	GGCAATCAGTTACTGATTCTTCGT	GTTATGAATTGTGATATGTTGGCAA
1421	tarc0567	CoreSet	4A	38.4	TGTTGCCTTGGTAAGTGTTCATAGT	GCTTTGTGTTCTGCCAGGTAAGT
1422	tarc0592	Other	4A	45.3	CGAAGCAGTGAACCCAATGAG	GGTGGTACATCCATAATTTGAACAG
1423	tarc0558	Other	4A	45.7	AACTAATGCTTAGAACTACAAAAGATGT	CCCACCTACTGTGAATTTTGTGT
1424	snp1992	CoreSet	4A	46.1	GTTGGTCATTCTTTTGGATAGGAG	CAATCTTCTCCAACCTCTACCAAA
1425	tarc0560	Other	4A	46.1	GCCTAGTGAAGCCTTGATCTCGT	GACTTCATGATTCAGTTGTAGCCG
1426	tarc0573	Other	4A	46.6	TTACCATGCCCAACTGAAGAAA	GCCTGCAACTGCGATGAA
1427	tarc0583	CoreSet	4A	60.0	AGTGATGTTGCCTAAATGTGGTG	ACTTTATGACGTGACTCTTGTTACTTAG
1428	tarc0607	Other	4A	60.7	TAGTTTATATAGACCCCATGTTCACTGT	CACCCCACCAAAAATTGCC
1429	tarc0609	Other	4A	64.1	CACATGCATTACTTCTGCAAACG	ACTATCAGGCGCAATATTGATTG
1430	tarc0551	Other	4A	77.4	TGTGTGTGTGTGTTTTCTTCCGA	TTGGACCTCTTGCTGAGCCT
1431	tarc1818	Other	4A	95.0	GCCCGAACACCTCGTTGAC	TTTCGTCCGCATGCAAACTA
1432	tarc0556	Other	4A	101.0	ATAGAAACTAATCGAGGAGTAAGGCA	TGTCCTCTTTTCTTAATAATAGTATGGT
1433	snp5652	Other	4A	105.2	CAATGATTTCAATGGGTCTGAAAC	TGTCTCAATACAATACAGGAACCGA
1434	tarc1793	CoreSet	4A	105.2	TGTAATGGGAGATTAAAGATCAGCA	TCCGGTGTCGTGTTGGAACT
1435	tarc1809	Other	4A	111.2	ATGTACAAGGAATACATCAACCCAG	CCACAGCTGCCACCATGAA
1436	tarc1807	Other	4A	111.3	ATATTGCTAGATTTGCACCTTGCT	AGTTAGTTGGTATACTTTATTCATGGCT
1437	tarc1806	Other	4A	112.6	CTTGTCGCCTTGTGCAGTG	TCGTGAAGCCGGTGTGGG
1438	tarc1811	Other	4A	112.9	CAACGAATCCTATATGCAAGTAAATAA	TGTCTTCATCTACTGAAGGGTATAGTCA
1439	tarc1799	Other	4A	113.9	CCGAGAGGAACCCAACCC	TGAATTCCTGGTGATTTGATTACG
1440	tarc1800	CoreSet	4A	113.9	GCTAGGATCAGAGCTCCACTCA	CGTTCACCACCGAAGGGG
1441	tarc1803	Other	4A	114.5	ACATGCCCAAGTCCTGCTG	CCATGGGGGACAGGTTGTTC
1442	tarc1797	Other	4A	115.6	CATAATCTCCTTGCCTCTGCG	TTAACATTATGCTACAGGCCACTATC
1443	tarc1810	Other	4A	115.6	CGATCAAAGGGACTACCACCA	GGCGGACCTATAATTCACGG
1444	tarc1801	Other	4A	117.1	CTGTTGAATTGATAGATTGTTAACGATA	ATCCCGCTCCCTCTTCTTTT
1445	tarc0598	CoreSet	4A	119.7	AAGGTCCAACTAGAGAATCAGCACT	CACTGTTTCCAGCAGTTGACAAC
1446	tarc1795	CoreSet	4A	125.0	ACCATGTTGGAATCTATTGCATCT	AAGTCATGGCTGGTCCAAAGA
1447	tarc1817	Other	4A	125.4	ATCGGACATGGCCTTATGTTT	CATAATCTAAGCCTTATACACATCGG
1448	tarc1796	Other	4A	126.4	AACATTAACTGAAGCTCCAATACTAGTT	GCCTTTGAAAATCTGGGTGTTT
1449	tarc1798	CoreSet	4A	135.3	GCCATGTCAGCTAACAATTACTTAAATA	TCTTCTCAAAACTTTCTAGCAGGG
1450	tarc1816	Other	4A	135.6	CAAGCACGCCTGCATCACT	CACACACACACAAATAGCTGGG
1451	tarc1812	Other	4A	136.6	GTTATCATCAAGCCATTGAAAAACT	AGCAGTGTCAGCTTTCAACACAA
1452	tarc1802	Other	4A	137.5	ACTGGATTAAACTACATTTGTTTCTACT	TGCGAAACTTTGCTATGGACAG
1453	tarc1815	CoreSet	4A	137.5	CCCGCATTATGAGTCTAAACTTTTC	CATGGGGATACGTACATAGAGACTAATA
1454	tarc0603	Other	4A	157.7	CGGGGACTCCAAGATCCG	CGATGGAATCTTGGCCGT
1455	tarc0606	Other	4A	175.9	AGTCCGTCGCAGACGTGG	CCAGGCAGCCGATCGAAA
1456	tarc0570	Other	4A	181.6	CCCCAGCATACAGATGGCTTA	CAATGATAGCGGCGAAGAAGAT
1457	tarc0586	CoreSet	4A	184.0	TGTGTACATGCAAACCCAAATGA	TGCAGGTTCTGTTTCTAAGTATGATTT
1458	tarc0604	Other	4A	215.1	CCTTGAGATAACATAAACATTATTCGC	ACTCTCAGTTGACCTTCAAGTTCAGT
1459	tarc0602	Other	4A	252.7	TTTTTGCTAGGATTAATTGAACTGTG	AATTTACCTAATAGCATCTGAACTAACT
1460	tarc0610	Other	4A	353.9	CCAATTGATCGTTCTATAGCACTCTT	ACGGAGATGATGATTACATATGAATAAA
1461	tarc0568	CoreSet	4A	441.8	TAAAGATTGTAAAAGGAAAATAATGGAC	CGTTACGCTGGTGCTTCTTTGT
1462	tarc0550	Other	4A	465.9	CATAGTCACAGGAATACCTGTGGTAC	AATCTTTATTGGCTGTGGTACTAGCT
1463	tarc0597	CoreSet	4A	490.6	CGTGACAAGGAGGACAGCAAG	CCATCCTTCTTGTCCTCTTCGAT
1464	tarc1782	Other	4A	492.5	TCATACTCCCATATACCAGTCTGAGTT	TGTTACATTCATGTTTTATATCATCAGA
1465	tarc1784	Other	4A	497.0	TCTTGTACTTGATGTAATCAGCCCT	GTGTGGACATTGGCCAAGTAC
1466	tarc0595	CoreSet	4A	513.3	CGCATATGAAAGAAAAGCTGACA	GCACTTGCTCATTCCATTCTAAA
1467	tarc0596	Other	4A	520.1	GGAATTTTCGATGAGCAATATGA	CACTCACGCCCTATCAACTTTCT
1468	tarc0594	Other	4A	523.5	TTTGTCTTATGCCTATTTTTGTTGTT	CCAAACTTTCTTCTAGGCGACA
1469	tarc0619	CoreSet	4A	540.4	CAAAGCGGTAAGTTGAGTAGTTGAC	TGGAATGCATCTCTGAACATCG
1470	tarc0574	Other	4A	541.7	GGTGCACATCAGCTCTGAAA	AGCATTCCAGCTTTGCATTT

Supplemental Table 4. List of amplicon sequencing primer sets for wheat.

No.	Name	Type	Chr	Pos[Mb]	F_primer	R_primer
1471	tarc1764	Other	4A	544.2	GCAATAGGAAGTAAATGAGGATGAAGT	CTGCTTGCCTACTCCCCACT
1472	tarc1791	CoreSet	4A	556.8	ATATCGTATGTGTTTCTTAAGGACTGAA	TAGGTACCAAACTGAAGGACTTCG
1473	tarc1789	Other	4A	559.0	CAAGGGCGGCAGCCATTT	GTTGCGCCGATTTGCTTT
1474	tarc0578	Other	4A	560.4	ACTAGGGTGATTATTTATTTATGTGCG	CATCCCATAAAAAAAAGCGATAA
1475	tarc1785	Other	4A	562.3	CAAACAAAGACAAGTTTACCCCCT	CAAACTGGCGACCACGGC
1476	tarc1776	CoreSet	4A	567.7	CACCTCTGTATTAAGGATTACCAGCT	TTGCTACAGAACCCCAGTTGC
1477	tarc1779	Other	4A	567.7	AGAGGAACTTGGAGGCAAGTTT	GCATGCAGTCCAATAGATAGATAAGA
1478	tarc0613	Other	4A	569.9	AAATTGGCTTGTTCAATGTCTCA	ATTAACAGTTTCCAAAAGAGTTCTGC
1479	tarc1787	Other	4A	569.9	AAGGAACAACTTAAATCAAAATGGTG	GTGGTTTAATTTGAGCTTGCACA
1480	tarc1772	CoreSet	4A	570.5	GGCACCAGGTTGGTCTGAA	CCCAACATACACCAACCTCAATA
1481	tarc2589	Other	4A	570.5	AGGATGCAGCCAACAACTTTG	TGGAGACCAGTGCAGTTGTCTG
1482	tarc1769	Other	4A	572.8	GCCCACAATTTAGCCAGGTC	ATGGGTGCTGACGGTGCG
1483	tarc0552	CoreSet	4A	576.2	ATAATTACTTGTGTTTTCAATTCTGGTC	GCCAGTTCCTCTTTACCAGTTAGC
1484	tarc0553	Other	4A	576.6	TGGAATTGAAATTTGAGGAGGTATC	CAGCACTGGGAATGAAAAACG
1485	tarc0554	Other	4A	576.9	CCTCCTGCGCTGAGGTCTG	CAGGTCTATGCCGCATCAGTAA
1486	tarc0593	Other	4A	580.3	ACAGGGAGAAAATGAGAAGTGCT	CTTTTGTGGTGAGTGAACTTACAATT
1487	tarc0623	Other	4A	581.9	TGAATGCGCCAAGAAACGA	ATGTCGTTGTGATGCAGTGTAATATT
1488	tarc0621	Other	4A	583.9	CGCCATGTAGGTCGCTGTGT	AGCTGCTACCAACTCACCCG
1489	tarc0624	Other	4A	584.7	ACCTGATGTAATACGATTGGAACG	GCAGCCGCACCTTGTACG
1490	snp2606	CoreSet	4A	591.7	TCTGATACAATTTCGCTGCCC	TTCGGCATCCTAACTTGTCGTA
1491	tarc0582	Other	4A	591.7	GAAGCAGATGGGAGGCGAT	GCTGCCTGCTCCTAAAGTTCG
1492	tarc0584	Other	4A	591.9	ACAGTTCCCGACGACTAGAAGTAA	CCGTGTTGTTCTCCAGGTACG
1493	tarc0601	Other	4A	593.2	GCAATGTTGTTCCAGTTTCTGC	AAAGCAACTCTCTGCAACACACA
1494	tarc0608	Other	4A	595.8	TTGTCACAAACAAGTTAGCAAATATCTA	TGGACTTTTGTTTCATTGCTCAGT
1495	tarc0563	Other	4A	595.9	TGCCTGATGTTTGATCATGTCTG	TGATCTTTGTAGCCAAAATAGAAGTGT
1496	tarc1770	Other	4A	596.8	TGATTGTATATGTCTCGGAAAGCAG	TAGGAAAACAACTCATTTATTAGGTCAT
1497	tarc1777	CoreSet	4A	596.8	GCCAGGTCAGTTGATCTCCTATT	GGTCACAACATAGACTCAATTCATTGA
1498	tarc0620	Other	4A	597.7	TTTGTATGGTGTAGAAAGATGGCAT	ATTGAGAAGTAGGTGCTGTGGC
1499	tarc1767	CoreSet	4A	597.8	CCCATCATGTAGCTGCTATGAGG	GATTAGACATGAACCAAGCAAGGA
1500	tarc0600	Other	4A	597.9	TTTGAATTTCTTGCAGAGCTCATAT	AGCAAGCACTCCATTTACTCCAT
1501	tarc0617	Other	4A	598.1	AGGAATATATGACATTTACTTGTTCATA	CGGACAATCAAAAATCAGTGC
1502	tarc0569	CoreSet	4A	598.7	TACTGTTGTTTCCTGAGTGGTAACTTA	GTTCCTGGTATTGCTTTCACACA
1503	tarc0580	Other	4A	598.7	GGATGTACAAGGATGCTGCG	TTGCCTCCATGCCATCGT
1504	tarc0590	Other	4A	600.9	CGTCTGTAACATTTACTGGCTGTGA	ATGCCCCTGTAGCTAGACACCTA
1505	tarc0576	CoreSet	4A	602.1	CAGGGATCCTAAATTTGAAGAAAATAT	TGAAATGTTTGGCCTAAGATGC
1506	tarc1765	Other	4A	605.7	TCCAACTTCCGCCTTGTCA	GCGTATCCACCAGTACGCGT
1507	tarc1778	Other	4A	606.5	CCTGCAAAACCCTCGTCG	GGGCTCATATGGGTTGCTCA
1508	tarc0566	Other	4A	607.1	CTCAAACTATGACAACAAAATGCG	CATGATGTTTATAGTATTATAGGGTGTT
1509	tarc0614	CoreSet	4A	610.5	CACCAAGAAGCAAAGTTGACATT	TGTGCCATATCCCATATCTACTAATACA
1510	tarc1781	Other	4A	614.4	GCAAACATAGGATGTTGACTTTAGAGA	CAAGCTGAAGCATCTGCGTCT
1511	tarc0565	Other	4A	614.9	CTTGACGATCCAGGCACAGAC	GCTTATTTTGCTGGTGCTCTTCT
1512	tarc0589	Other	4A	617.2	GATACCTTGCTTATTTGTTTGATTTG	GGAGGTTTCCTGAGTGTGAGAC
1513	tarc1788	Other	4A	617.2	ATACAACCGTAAGGATTAGAATAAAGTA	ATGTCAATTTTCTCTGTTTCCCAG
1514	tarc0587	CoreSet	4A	617.9	AAATGACATGGTAAGACTTCAAATAAAT	TTTCAAGGTATAACTATCACAAATGATT
1515	tarc0605	CoreSet	4A	619.2	CTGATAGCTAGAAGCAGCATAAGAGATA	CTGTAATTATTTTTGCCCATTGTGT
1516	tarc0611	CoreSet	4A	627.8	CGAAGATGAATGAAGTAACAAACAGTG	CCAAAGACAAACTCTAGCATTTACAA
1517	tarc0612	Other	4A	628.5	GTCCATCCCCCAGTTCTTCT	ACGTCCAGTCCCATACAAGG
1518	tarc0599	Other	4A	628.9	GTCCAAAGGTATATGTCAGATAGTCAC	GAGAGGTAACAAGAAAAGAACCTAAGTT
1519	tarc1786	Other	4A	633.4	GCTGAGGAAGATGTGCTTTTGTA	ATGATCTTATAGGTCATGATTTGCCT
1520	tarc1783	CoreSet	4A	634.8	GCAAGAAGCAATGGTAACTCCC	ATGCTCTCGTTTTTCGTCGG
1521	tarc0561	CoreSet	4A	636.6	AATGCTTGGGCCTATTTCTCAG	CAACCAGTGTGGATCTTATTTTCTC
1522	tarc0564	Other	4A	640.2	GGACAGTGTCGTGGTAGAAATCAT	GCATTGTAGGGTGTCCTGGTGT
1523	tarc0588	Other	4A	640.9	TGGTGGCTCATCATCCTTGG	GAGGTGTTCAAACTCGCATCTATT
1524	tarc1762	CoreSet	4A	649.2	GCACCATTTTTTTTCACGGG	CCATGCATGGTGTAAGCTGGA
1525	tarc0575	Other	4A	664.5	GGCAATTGGCAAGCATCAGT	AGTTAGCTGCTGAGAAAGCATACC
1526	tarc1768	Other	4A	673.4	GGAAAATGACAAACCACACGA	AGAAGAATCTTGGGATCAAGTTCAC
1527	tarc0591	CoreSet	4A	675.9	AACCTCTTGGTGAGGCATTTGT	GATGGTGGTGTGTCAGCTCG
1528	tarc0616	Other	4A	676.5	CCCAGTCTTGTTTCTGCCCTC	CCTGCCTAGTAAAGCTATGGTGAAC
1529	tarc0626	Other	4A	680.0	TGACAGCAAAGCTTCAAATGAAA	TGTTTGAGATTATCTGATGTAACTGGAT
1530	tarc0577	CoreSet	4A	683.9	ACCTGAGCGCCAACTCCC	ATCTCGCCGGACAGGTCG
1531	tarc0615	Other	4A	684.6	GGGTGGAATTAGAAAAGGTGTGG	TCCCTCTGTCTGGTGTATCCG
1532	tarc0579	Other	4A	685.0	GAACGAAAAGAAAACGAACATAGAC	AGAAAGGGAGATACCTGGAGACA
1533	tarc1775	Other	4A	693.0	GGTGGCTGTGGTCGCTTTAT	GGGAAATCCGATGCTGAGTG
1534	tarc1761	Other	4A	693.3	AGACTTTGCTACTGAGAAGAACATCC	AATCTGGCAATCTTGGCGG
1535	tarc1763	CoreSet	4A	697.9	GAAATGGGGATACAACACACAGA	AATTAGGCCATGTTTGACAGCA
1536	snp1066	CoreSet	4A	712.9	AAAGCAAAGTAAACATGCCTGG	AGATGATCACGTCGAATGAGGTC
1537	snp2170	CoreSet	4A	713.5	AGGATTTTTCCAAAGGTCCACA	TTACGGGCATTAAGTCCCGT
1538	tarc1766	Other	4A	717.7	TGCCGTCCGTTCCTCATCT	CATGCTCATACGAGCAGAGACATAC
1539	tarc1780	Other	4A	723.8	ACCATTTGTAAAGTTCTTAGTGTAGCTA	CACACTGCATAAGGAAATCCCT
1540	tarc1771	CoreSet	4A	724.8	GTGGGTGCATTCCGCTGC	AAGGCCTGGTTGAACAGGATAC

Supplemental Table 4. List of amplicon sequencing primer sets for wheat.

No.	Name	Type	Chr	Pos[Mb]	F_primer	R_primer
1541	tarc1773	Other	4A	725.7	ATGCATTGAGGATGAACTCGG	GCTACCTGAAATCAAAAGCAAACA
1542	tarc1774	CoreSet	4A	725.7	GTGCATGGTAACATTAGAATGGAA	GCGGCATCACCAGTCGGA
1543	snp1674	Other	4A	732.5	TTTACTCACCACCACGGTCTTCT	CAACGATGGTTAAGTTTTAGAAGATT
1544	snp1836	CoreSet	4A	737.4	TCTGAACCAGTGGACGATGCT	GAGGCGTGGTTATTGTGGTGT
1545	snp1410	CoreSet	4A	742.4	GTGCATACATATCAGTGCATTTTAGT	CTTCTGCCATCAGATTGGTCA
1546	tarc1032	Other	4B	1.3	ACCGCCAACCATCCTTCTC	ACGAGAAAATGCTGCTGTGG
1547	tarc1041	CoreSet	4B	1.8	CAGAAGTAAATGAAGTGTGGCATG	CATATATCAGCGTTAATTTAGACTACGT
1548	tarc0995	CoreSet	4B	9.7	CACAGCACCTAAGTAACACGCATA	ACCTTCTTAACGCTTCTCATATAACAG
1549	tarc1053	CoreSet	4B	10.6	GCGTATAAAATGCACACGGTT	CAAAGAAGCAAGAGTAGCCTGG
1550	tarc1054	Other	4B	11.7	TTGGATGAACGGCCTTGG	ATGGACAACCAGCTATCGGC
1551	tarc1031	Other	4B	11.9	AGCAAAAAGATGCAGACGGC	GTTAATATGGTTGATGCTTGGGT
1552	tarc1040	Other	4B	11.9	TCGTTTATTAGTCATGTATATATTCTCA	CCTAGTGAGAAGTCCGAGAAAATAAGT
1553	tarc2178	Other	4B	13.1	GGCTGTTGCTGAGTTTTCAACA	TGAAAATGAGCTGAATGTGCTCA
1554	tarc2179	Other	4B	13.3	AGTGGGAGCAAGATAGAATTTGACT	CCTCCCACTAAACTTGGTCAAA
1555	snp7266	CoreSet	4B	14.0	GGAAGTACAACGCCAACTTGC	TACACAGATTGCAGGATTGGAAG
1556	tarc1062	CoreSet	4B	14.0	CATCGCCAAGAAGAACCTGAA	GCGGAGCTTGTTCTTAGATACTGT
1557	tarc2184	Other	4B	14.0	GATGTTCAGAGAGTATGCAACGG	ATCTCTTATTCATACTAAATTTTGATGA
1558	tarc1045	Other	4B	15.4	GCGTCTGCGTACTGCCGC	CTTGGAGTTCAGGTTCCTCTAGAAC
1559	tarc2182	Other	4B	15.4	TCGAGAGCCTGGAATCTCTCA	CGAGAATAAAAGGCTAAAGTAATTAGGT
1560	tarc1003	Other	4B	16.1	GTTTCTCTTTGTTGTCCTTCCTTTTA	TTCTTCATCCTCGCTCAGCAT
1561	tarc0990	Other	4B	17.1	AACCAGGCAAAAGTGCAGTTT	TGCCGTTTTCCGTATTCCC
1562	tarc0998	CoreSet	4B	19.7	ATGGCCCATGTGAGAGTTCAC	CGTTGACAAGTGGCAGTCCTAA
1563	tarc2180	Other	4B	21.3	ATACTGGCCTTCATTGCAGTTAGA	TTATGTCATCCTGCATAAATTGATTT
1564	tarc1056	Other	4B	21.9	ATCTCAAGATCACGTATCCCACC	CCTTTGAGCAGAAAACCACCAA
1565	tarc1069	Other	4B	21.9	GACAAGCCAAGTAAGTGCATGTTC	GGTTTCCATGTAACTTGCTGCA
1566	tarc2175	Other	4B	25.8	ACTATGAATCATATCAAGAAACATTCGT	CGCTGCTCATACAAAACCACG
1567	tarc2176	Other	4B	25.8	TCAATGAGCAAACATCTTGTTAGGT	AAGCTGGACTGTCAAAAATGAATC
1568	tarc1043	Other	4B	28.4	CATTCCTTTTCCTCTTTCAGGAGA	CGGACTATCCAGGAGGAAGATAA
1569	snp500	CoreSet	4B	29.0	GTGTACCGTAACACCTTAAACTTCAC	AACAACCATTGGATGATGACTGA
1570	tarc2172	Other	4B	32.5	AATGACATCATTAACAGCTCCTGTG	GGTCAATAACGAAATTCTGTCAAAC
1571	tarc2181	CoreSet	4B	34.0	AATGGCAGCAGATCCCGA	GAGGTTGGCCCCCTTCCC
1572	tarc1020	Other	4B	37.9	CTGTCAACAAAACTGAATGGTATCC	GATTCTTTGTCTGCTTGGATTCAT
1573	tarc0991	Other	4B	38.0	GTGCAGCGAGAAGGGATATG	ACATCCCACTCGGGGTGTT
1574	tarc1002	Other	4B	40.2	TTCCTTTTTCCTCTCTTCTTATTTCA	CATCCAATCCCTCAAGTGTTCA
1575	tarc2177	Other	4B	47.0	AACAAAAAAGAACATCCAGCACA	CAGACGACCACCAGACCCG
1576	tarc2173	CoreSet	4B	47.6	GATGTCTTCCAGCCAACAACAA	TATGCCATATAAATGTTGTTTTAGATCA
1577	tarc2174	Other	4B	49.5	GGCAACCTACCAACCAAAACA	TTTAGGACTACTTTTGTGATCTAGACAA
1578	tarc1063	Other	4B	54.3	GTCAGTGCAATTAAAACGGCAT	CACTCTGTTCATCTTGTGTTTCCAT
1579	tarc2183	CoreSet	4B	57.0	AAGGTGAAAATGGCCCCG	CATCAACAGAAAATAGAATCTTCAGTG
1580	tarc1015	Other	4B	61.3	CAAGTCCTAGAAAAATAACAAAGTTCAG	AGAAGCACGGGTTTCTCTCAAG
1581	tarc1064	CoreSet	4B	71.6	GCCGCTGCAACAAGACGC	GTGCAACTCCACTGGCACAT
1582	tarc2185	Other	4B	131.7	AATTCTTCAGCCTTTTCAAGGTAGT	CAGATTCTTGATGAGTTAAGTGGACAG
1583	tarc1018	Other	4B	135.1	CCCTAAAGCCTCAAGGATATCGT	GCCAGCACTGTTCATCCAAA
1584	tarc0996	Other	4B	136.5	AGTGACTGTGTGATCTTTACTTCTACTT	TTGCATCATCAAGAGAATCGTTG
1585	tarc1037	Other	4B	139.4	AGGCCCCTCTTCTGGACAA	AATGACAAAAGAGTAGCTGCAAATC
1586	tarc1004	CoreSet	4B	140.7	CCGTTGGGGAGAAGGATTC	GCTTCAAAGTTATTGGGCTTATGT
1587	tarc1067	Other	4B	144.7	CATGGTCGTGGCACTGTAACTATA	CAAGTGGTCACAGTAACTTGACGA
1588	tarc1055	Other	4B	149.7	TGCTCAACATCCATTTTATTACCTAAG	CCACCGGAACAAATTAATTAACTACT
1589	tarc1059	CoreSet	4B	171.1	CACCGTAGGTATTCCTGTGACAA	GAATTTTGGTAAAGTACTGCAAGGT
1590	tarc1024	Other	4B	171.6	GCCAATTTGTACTGAAATACATAAACC	CTGACAACAGGGCTATCCACC
1591	tarc1001	Other	4B	307.6	GCGTCTTTAATTTATCTGAGGTGAAT	TGGGTTTGGATTGACTTCATAGTG
1592	tarc1013	Other	4B	317.0	AAATTATTAACGATAGTTTCACATGGTG	GAAAGAAAATGTACAACAAGAAATGGA
1593	tarc2162	CoreSet	4B	351.9	CTCCCTCGAATGATTTAAAACAAC	GGTAAATTTGGTCTGTCAAGTTGC
1594	tarc1044	Other	4B	375.1	CAGTGAACACTACTTGGATTTCTCAGT	CTGTTGATCCATAATCCCTTTGC
1595	tarc1038	Other	4B	400.4	TGCCGTTTCATCTGTGCG	CCACCAAGTTGCGGTTATTCTT
1596	tarc1022	Other	4B	411.5	ATTCAAATAGGAGTAACAAATAAGGAGT	TGAACCAAACCCGCCCAT
1597	tarc1039	Other	4B	412.8	ATCTGGCTGCAAATACAGCACTA	TCCTCTCCCGCCTTTTGTC
1598	tarc0992	Other	4B	413.2	TTATGGCTGAGCTGAATCCGA	GCCATCCTGCTCTCCCGA
1599	tarc1061	CoreSet	4B	413.4	ACCTACGGATCACAATACAACAGTAATA	TTAGTCTCTATGTACGTATCCCCATCT
1600	tarc1035	Other	4B	414.2	GAATAAACCATCACTGCTTATATTCCT	TTTTTATTGCTGTCAGATAATTGCTC
1601	tarc0994	Other	4B	414.9	TGATCTTGTCAAGTGACCTGCATT	CCTGCAAATAAGAGGCACGGT
1602	tarc1068	Other	4B	415.1	AGCCTTCTGACAATTTAGCCTTC	ATCCAACTTGTGAATTGCTAATTCT
1603	tarc1065	Other	4B	416.4	CAGTTGTCGGCAACTGGAGA	GCCACAGGATGTTGTACTGAACTAC
1604	tarc1009	Other	4B	422.5	AATACAATCAGGCAAGGGGTAGA	GGCAAGCAGAGTCCTCCAAT
1605	snp58	CoreSet	4B	423.1	CAGCGCACTGTTCTAGCACAA	ATTTCTCCTAGATTCCTGGTTTCAC
1606	tarc1014	Other	4B	423.4	TGTACAATCTTCAGGTGCAAATGTT	GATCACAAGTTTCTCTTTTCCTAACTTC
1607	tarc0993	Other	4B	423.8	GAGACCTCCTTGACAATTTCTTCAT	TGCTGTGCTAGTTGATTGTTCTGAT
1608	tarc1028	CoreSet	4B	427.5	GGCAGTTTGACTAGTTGGTTCG	ATCGTATGCTAACGGTATCCTTCA
1609	tarc0986	Other	4B	427.8	CAGTACGGGAAGTGAGGGGAA	CGCTGACTGTGGAGGATGTGTT
1610	tarc1071	Other	4B	440.3	GCTTTTCTCTTCCCCCTGTCTAT	GGATTGGTCCCTATACTACTTGAGC

Supplemental Table 4. List of amplicon sequencing primer sets for wheat.

No.	Name	Type	Chr	Pos[Mb]	F_primer	R_primer
1611	tarc1049	Other	4B	441.7	TCCCTTCCTTGCCAGTGTCTA	TCCTCTGGAACATATTTTTTGGG
1612	tarc2164	CoreSet	4B	441.7	CAAAAATTATGCAGTAAGCCTTCG	GGCAGTTCAGAGTCTGCCTTGA
1613	tarc1052	Other	4B	449.1	CAGGATAGACTCTAACGTCCAGACT	AGGAAGTCCCGAGATCTGTAATTT
1614	tarc0987	CoreSet	4B	469.0	TAATTGACCTAAATAAATAAGGTGTTCT	GCTCAAGCAAGCAAACACGC
1615	tarc1016	CoreSet	4B	480.9	GCCAAATGATGATAAGCTCCAAT	CATTCAATTTTTTTCACAAATAGCAC
1616	tarc1034	Other	4B	483.8	CATTAAGCTAGAACACTGATGAAACAA	GGTCTTTGATCCTTATGTTTGTCG
1617	tarc1051	Other	4B	483.8	ACCCGACCCGCTTGTCAA	GTATTGTTTCTGTAGGCAACTCTTACC
1618	tarc1027	Other	4B	495.0	CTGTTTCTGGTTGCTTATTTTTTTAT	CCGTCAGATACTGGAGCTTCACA
1619	tarc0988	CoreSet	4B	499.8	TCCGCTTCAGCAGCAGTGT	AATAACAAATACAGTACAAAATTGTTCT
1620	tarc1076	Other	4B	514.6	TGCTGTTTGTGTTAGCATTTTTTT	CAGAACTGGGAGATTGTTGACG
1621	tarc1070	CoreSet	4B	517.0	GGGCACCGCTCTCCCAAC	TTATTTTATTGCAGGGGACAACC
1622	tarc1000	Other	4B	527.0	GTTATCCAAGAAGCAACTCCTACG	TGCTCAAATTCAGCTCTGGATACT
1623	tarc1042	Other	4B	532.4	GTTGTCATTGGCTCATTTGCAT	TGCAGCTTCCAAGAATTTACGTT
1624	tarc1017	CoreSet	4B	533.5	CACACAGTATATTCTCTTCTTGGTCG	GCATTTAAAGTTTCATTGAGTCACTT
1625	tarc1033	Other	4B	542.8	TATGATGATTATTATTCTTTTAGCAGTG	AACTGTGTCATCAATAGGAAGCG
1626	tarc1030	Other	4B	547.6	TGACCAGAACATTACTAGACTATTGACA	CACCACTGAGGTCATTTTATTAAGAC
1627	tarc1058	CoreSet	4B	551.0	AAGGAAATACAGATCCAAACCAAA	GATAGTGCTTCCATTCTGAACCG
1628	tarc1026	Other	4B	552.0	AGCACATCGTCCTAGAGAACAATG	GCGGTTCTCATAGAAGGCGA
1629	tarc1072	Other	4B	552.8	GCAAATGGAAAAAAATCCAGAGG	CCTTCCTGGTATGGCTTATGTGA
1630	tarc1048	CoreSet	4B	553.9	CGGGGTTATCCCCTTTTTG	CAAGATATCACAAAGCTTTAGGTACTCT
1631	tarc1012	Other	4B	560.0	TGAATGAATTAAAATGACTGAACTCTG	ATGATCAATGAAAGGACTCTCGG
1632	tarc1060	Other	4B	562.9	TGGAACTATGCACCAGAATTACTTG	ACTTACGAGACCAACATAAGCAGC
1633	tarc2168	CoreSet	4B	562.9	GCTAATCAATGTGCACTATCACATGT	GTACAGTAAATGGTAGCAAATTAAAAAT
1634	tarc2170	Other	4B	562.9	TGAATCCTTTCCACCCTCACAC	AACAAGCGCCCATGACAGTC
1635	tarc2161	CoreSet	4B	579.4	ACTATAAGGGGAATATGTTTGTAATTCT	CAGTTTTTTGTATGCTTTTCTCTCA
1636	tarc1066	Other	4B	588.2	CCATCTTCTGATGTCTGGTTGTTTA	GGAACACCTAGAGGAAGTTTCCAT
1637	tarc1075	CoreSet	4B	589.2	CGTAATCCCTATGACTGCAAATGT	AAACATAAATCATCTGACATTGGGA
1638	tarc1008	Other	4B	590.9	CAGTTAAAGGTAGGCTCGTTGGT	AGCAATACAGCATTAGGCACATTT
1639	tarc2167	CoreSet	4B	594.6	GATCTGTTATGGGTTGTCTCATCTAAA	TTCCTGCTTATACTTAGTATAGTTGAGA
1640	tarc0999	Other	4B	595.3	TTATTCAGGTTATGAACCTAAGTTGGA	GTCATTTCATTCCATGATGACAGAT
1641	tarc0989	Other	4B	595.5	TGACCCAAATAGTCAGGATAACGA	CTATGAAAGGGGCAACCAAAT
1642	tarc1029	Other	4B	604.9	GCTGAGCTACGGTCTGCGA	CATCATATACAGGAGAACGCATCAA
1643	tarc0997	Other	4B	611.0	AAACAAAAAGAGCAAGTGTAAACCA	GCCGTTTTCTCTACAGATCGC
1644	snp4041	CoreSet	4B	613.2	CGAATCTCTTCTAGAGTACCTTAGCG	CACTAATCTGGAACAATAACCACTTAA
1645	tarc1050	Other	4B	619.1	GCAAGTGCTTTAGCCTTTCAAAT	TGTTGTCTCATATGGTTAAGGATGAA
1646	tarc1006	Other	4B	630.5	CAGATACCACTTGAAACATTTAAAATAG	AATTTCTTACACCAGAGGATGCAA
1647	tarc2159	Other	4B	642.2	GCATTTTCCAATATATGGCCGT	TTAGATAAAAAACCCTGTTCCGC
1648	tarc2163	Other	4B	642.3	CAACAACTTATAACAGCGAGAAGGA	ACATTTCAAGCAATAAAATGAGGATT
1649	tarc1005	CoreSet	4B	643.3	TTTTCTCCGGGCTATCTTTTTC	CCACTAATAAGCTTGCACTGTCG
1650	tarc2160	Other	4B	643.7	GTCGGAACCCTAGTCTGCG	AATTTTGCATGAAATACCTGTACACA
1651	tarc2169	Other	4B	644.8	CGAAAGCCTTCGCCTCCG	GGATTACCATCTAGGGCTGACG
1652	tarc1019	CoreSet	4B	645.4	GCCATCTTCAAGATGAACCCTG	AGAGATTCTGATCATATACCTGAGAGC
1653	tarc1046	Other	4B	646.7	TGCTCAACCACCTGGGAGTAAT	GTCAATAGGATTCTGAAAGTCGAAG
1654	tarc2165	CoreSet	4B	649.5	GGTGCTCTTGCTTCTGTCATATCTC	ACCGAGTCTGACACTGCAACTT
1655	tarc2171	Other	4B	649.5	ATGACATGTTTCTACAAACTTCCAATA	AATAGTACGGAAAAAAATGTTCCATAA
1656	snp2031	CoreSet	4B	650.9	AGAAGTTCCGAAAAAGCCAGG	TCATCATTTTCCTGCTGTTGTAGAT
1657	tarc1025	Other	4B	650.9	GTGCTGTGGTCTGGATGTTACG	AAATGGATTGCACTACAATATGACA
1658	tarc1036	Other	4B	651.0	CCCCATAACACTTCAACCCATTA	TCAGGAGGAAGGTGCCCG
1659	snp2595	CoreSet	4B	652.0	CCATGACCAGCAACCATGGT	CGCCTGAATTTCGGTGCAC
1660	tarc1021	Other	4B	652.8	GTCTATCTTGAAAAGCAAAATGAGC	TTGATCCAGCCAACACACAATAA
1661	tarc1074	Other	4B	656.8	CGGTTAGCCCGGCAACAA	GTGAAGCAGGTGGTGCGAA
1662	tarc1023	CoreSet	4B	657.2	CAACCTGTAAGTACTGTGTGTGCGT	GTGCATGTGTGGCTTTCTCCT
1663	tarc1007	CoreSet	4B	658.0	GCGCAAAACATGCTTTGACA	GTTCAAGTGTGAGAGCAGCAGC
1664	tarc1011	CoreSet	4B	660.7	GCCACATCCTCCTGCTCATA	GAAATTGACCAATTCCTAGCTTGA
1665	tarc1057	Other	4B	660.7	GCCGAGTTCAGCGTAACCG	CAAACCACAACATCCACAAATTAC
1666	tarc1010	Other	4B	662.4	TCCTGCATGGTGACAGCTATCT	ATGTACCATTCCTTGGTGCTTTC
1667	tarc1047	CoreSet	4B	665.4	CGCTGATAAGGCCAATAGCC	TGTATGTTCATAGTTTTGCTAAATGGAT
1668	tarc2166	CoreSet	4B	665.4	TTAACACGACCTGAAATTTAGTTATCA	GCCACCTGTTAATTTCTCAACG
1669	snp2178	CoreSet	4B	670.4	ACCTGCAACTCACTGTATTCAGC	ATGGGGAAGAGATAACTACACAGAA
1670	tarc1073	Other	4B	671.8	GCGTCTCATTGATTTTCTTTGGT	ATGCTAGTAAAATGAACATTGCGA
1671	tarc1513	Other	4D	0.5	GCGGGTTCCTGGTGGAGT	GCATGGCCCAGTCGAGGC
1672	tarc1409	CoreSet	4D	1.3	CAAGTATCTTTTTCCCGTCCG	ACAACTCCCTCAGCCCCG
1673	tarc1448	Other	4D	1.4	ACCTCAACTATCAAGCGAGAAGTG	TGAATCGGTTGGCAGGGG
1674	tarc2439	CoreSet	4D	3.6	TCATAGACCTCAACAGAACAGTTAGAAA	GGAACCGAGGGAGTAATATAAAGG
1675	tarc2436	CoreSet	4D	3.7	TCTCCCGTGCCAGAGTGC	AAAAACTACGACAAGTAGACATATTTAA
1676	tarc1491	CoreSet	4D	4.1	AAACTTGGCATTCATATTTAGACCC	GTGAAATTCAAGTTCAAAAAACAGAG
1677	tarc1470	Other	4D	4.4	ATATAAGTCTTTGTAGAGATTGCACTGA	GCGATTTTGAGTTGCGGTG
1678	tarc1481	CoreSet	4D	5.6	GCCAAAGAGAAACAACAATACAAGA	AGGGTTAGGTCAACAGCCGA
1679	tarc1405	Other	4D	5.7	AGTGACCAGGTGCACACTTTTCT	CCTCAGGCAGTACGGAATCAA
1680	tarc1290	Other	4D	6.5	GAAGATACCTAAAAAGTGTGCATACTGT	ATATGGTCGAGACAAAGTGGAGG

Supplemental Table 4. List of amplicon sequencing primer sets for wheat.

No.	Name	Type	Chr	Pos[Mb]	F_primer	R_primer
1681	tarc1454	Other	4D	7.7	CTACGGAAATGGAACGAACG	AGAGTGAGAAAGGAAGTACCAAGAATA
1682	tarc1277	CoreSet	4D	7.8	CTCGTGCGGTTCAATCGTG	AGGAGGAGGAGTAGGTGACGG
1683	tarc1519	Other	4D	9.2	CGCTCCCAGCATTTGAAGACT	GAGAGATAAGGCTAGTGAGTTACCGA
1684	tarc1402	CoreSet	4D	11.0	AGTCTGACAGAAATTGCAGTGAGC	CCATATAGGTTTCCCTCTGTTTGTC
1685	tarc0027	Other	4D	11.1	CTACATGATAATAATCAACACCAACTAA	TTCCACTCTTTTCAGCGGTATG
1686	tarc0025	CoreSet	4D	12.4	TCCTCTTGATGTTTGCTCGGT	CATGTCATATTAAGCATTTATACACCG
1687	tarc0029	CoreSet	4D	12.8	ATGGAATATTCTAGAAGGATCAGTACAA	ATCAAAATAATGTGATCTTTCCTCAT
1688	tarc0018	Other	4D	15.4	TTCAAGGTGGAAGAAAGAACTATATGT	AGGTAAACCAAGAAAGCAAGGG
1689	tarc1520	Other	4D	15.6	CCCAGATCAGATCTCTCGTTTCA	TACGCTTGCTGGTTGCTTTG
1690	tarc1341	Other	4D	16.1	GACGGCAGGGGCAGGATG	ACAGCGCCACACGAGGCA
1691	tarc1517	CoreSet	4D	16.6	CTGCTGCCATGTTGCTGC	ACGACGCATCAAGGGTGG
1692	tarc1452	CoreSet	4D	22.0	GTAACATAACGCAATTCAAGAAAAAA	TTTGCCCTCACGCACGCT
1693	tarc1473	CoreSet	4D	27.2	CCTCCATCCTCACCAGTTGC	CTCAGTACAGGTTTTTCATGAGAAGT
1694	tarc2437	Other	4D	30.5	GTCCATCCAGTGAAGTCTATCAGC	CAGGTAAGAACTCAACATGTTCCTAG
1695	tarc1464	CoreSet	4D	39.3	CTCAAACACGAGAGCAGAGACG	CAGTCTCTGACGCTTTATCCTGG
1696	tarc0024	Other	4D	43.1	CAGACTGAAAGATTCTGCACAGAT	CCTGAGATTTGCATCACTGTTATTT
1697	tarc1543	Other	4D	46.4	CTACAGAGCATCATTGTATGTTCAGTG	GCTAGATAGCTCATTTTAATTACTTCAT
1698	tarc0020	CoreSet	4D	48.0	CTTTTGGACACATTACTTTTTCACTG	TAATATGCAGAAACTTCAACCCTTC
1699	tarc0030	Other	4D	48.7	TTTTTGCGTATCTTCACCTCG	CACTAATACCTATCCTATAACTTCCATT
1700	snp7344	CoreSet	4D	50.2	GAGTATCTGGCAACTCCGCA	CATCAGGGAGCAAGGTTTTGT
1701	tarc0021	Other	4D	50.2	ATCAGGGAGCAAGGTTTTGTT	GAGTATCTGGCAACTCCGCAT
1702	tarc0019	Other	4D	53.2	TATGGACTTGAACCTCCATCGT	GGTGGCACTGCGTAAATCGA
1703	tarc1521	CoreSet	4D	55.1	CGTCCACCAGGCTCTTGATTC	CCTGGCTACGTCGGGTACA
1704	tarc0026	Other	4D	57.1	CACAATACAGGTGGCTCACCAT	GATTGTTTCACCAGTTTTTTTGTG
1705	tarc0023	Other	4D	60.9	TGTTGGCAGTGTACTGCACCA	GAACTGAAAAAGTTCGAGGTAATTT
1706	snp5381	CoreSet	4D	62.5	TAATAAAAGATGAGATCCATGTATGTAGC	TAGTGCTTTGTACGAACCAAGCT
1707	tarc1318	CoreSet	4D	75.2	CAGGCGTCTCCAAGCGAAC	AGATTTTGCGAGGGGGGG
1708	tarc2438	Other	4D	85.0	TGCCGAGCAGTTCTTGAAGC	CCTTTGTACTGCTAGTTGCCG
1709	Inf34360	Other	4D	89.5	CCATGGCCCAGAATCCTTA	AATTCTGATGGAAAACTAATTGCAT
1710	snp6277	CoreSet	4D	94.6	TGACTACCAGAAGAGCCGGTTT	GATCTTAGCCTTGTCACCAAGG
1711	tarc0028	Other	4D	98.5	CCTAGTTCCTCCCAAACATGTATC	TCCTCTATGTTCACCCTTCAGGT
1712	tarc0022	Other	4D	121.8	GGAGAATGTTCTAACCGTAGAAGGTAC	CAGGGCAATCAAGTTATATAGTATTGA
1713	tarc0013	CoreSet	4D	192.7	CACAGTATATTCACCACGTTATTGC	CTGCTTTGCTCCGAATTTTAA
1714	tarc0012	Other	4D	238.6	CACAAAATGATACTCCCTCCGT	GTTGTTGACCATAGTTCTGTGTAGGTA
1715	tarc0002	CoreSet	4D	251.5	CAGCGTCCTCAGGGTGGTG	ACTTGTTGCATATTCTCTTTCACAG
1716	tarc1507	CoreSet	4D	251.5	GAATTTATTTTAGCTGTCGATTTTTTG	GCGCATTTCTTGTGGGGTT
1717	tarc0010	CoreSet	4D	301.3	ATAAGGAGACAACAGACAGGCTTAG	ATATCTCAATTTGCACTTCCAGGT
1718	tarc0014	Other	4D	313.6	AAGGAAACTCACATGAACATCAAGTA	CCCACTGGCCGTTCGTTAG
1719	tarc0011	CoreSet	4D	346.5	AGAGAGTTGCGACCGATGTCA	ATTCATCCATGTTTTTTATGCCAC
1720	tarc0006	Other	4D	358.5	GTGCTTTACCAGTATAAACATTACCTC	CATACTTGTGCGGGTGCCTA
1721	tarc0003	Other	4D	368.0	ATCGCACGGCAATAACAAGC	GGTGGGGCTTCCAAATCAAC
1722	tarc2430	CoreSet	4D	369.3	GACTTGACAAGTGAACGCTTCAC	TGCCGATGCTGGACGATG
1723	tarc0005	CoreSet	4D	394.1	GCTCATTGGAACCGCAGA	CGGTGTACGCTCAGAACTCTT
1724	tarc1362	CoreSet	4D	422.2	TCATTCAACCATGATGAAGTAAAGATA	TTCATACTTCACTTAGTTTAGGTTAGGT
1725	tarc1350	Other	4D	423.7	GTCTACCAAATAGCACGACAACATC	GATTATCCACGGGTGCCTCA
1726	tarc0001	Other	4D	433.8	GGAACTGCGGGTTGTTCTCC	ACGGTCACCACGACGAGG
1727	tarc1342	CoreSet	4D	435.2	GTTGCTGCCAGCCCACTT	AACATATTTGTAACCGGAATCATGT
1728	tarc2434	Other	4D	442.2	CAGGTCCCCCACGTTCGC	GGGAAAACCCACCGACCA
1729	tarc1499	Other	4D	446.2	AAAAGTTTGTTATCTACGATAAGGCG	TTGTAAATTTGCTAGTTGAATTCCTACT
1730	tarc1500	Other	4D	455.5	ATTTTTGTAGTGGAAGTGGTGTGG	TCATATGGCCTACTGTCTGAACCT
1731	tarc0004	CoreSet	4D	465.1	TGGAGATTCTAGCATTGGTACGT	GGACCTGGTTAGTGCCATATCAT
1732	tarc2433	Other	4D	469.2	CTGCTGGAAGTCCTAAGGTGC	CCCAAAGCTGAAGAAGAAATAAATAT
1733	tarc0008	Other	4D	474.6	ATTACCGCTAAACAAGGTAACACTT	TGCTATAGCCTAATTTTATGTCCCA
1734	Inf17227	Other	4D	476.3	CCTGCACGCAAGGTAGATGTC	ATATTGCTGCACTGGTTCAAAACT
1735	tarc1351	CoreSet	4D	478.3	CGGAGCTCTGAGTCAAGGTTTG	CATATAGTAGAAGAACACCACCACGA
1736	tarc1441	Other	4D	481.2	TTAGATTACTGCTCTACATCCATCATTT	AAAAGTCCCTAGCAAACCAAGC
1737	Inf19400	Other	4D	481.6	GATGGCTCCTGTTTATTTATTACTGT	AGAATGATACACCTTGCTTGTCAGT
1738	snp3471	CoreSet	4D	483.1	TTCTGCATTTGTTCTGATTGCC	CAGTCTAATCACTAATGATATAGGATGAAG
1739	tarc1414	CoreSet	4D	487.5	TCATGTGGCAGGGCACGA	AGATGGTCGGCATCCGCA
1740	tarc0015	Other	4D	488.7	ATAGAGACTTACCTGCGTGCCA	GCAAGAAAGCCAATCTGACTTC
1741	tarc0016	CoreSet	4D	488.7	CAACGGCAAGTCGTTCACCT	TCGGCTGCGATCTTTCGG
1742	Inf46101	Other	4D	490.9	ACGTAAATAGCAGACTGACTATACAGGT	GTTTGGTGGCACAGTTTCGAT
1743	Inf15650	Other	4D	494.1	TTCTGCAACAACGCTTCGC	TGATTGCGTTGAGCTTCGAGT
1744	tarc1345	Other	4D	497.2	ATTCCTTCAGTATACAACTGCCACTT	GACAAATGGATAAATGGAGTTCATG
1745	tarc1319	CoreSet	4D	499.5	TCCCCTCCCTGATTCTCACA	TGCTCTGGTGCATTTGCATC
1746	tarc2435	CoreSet	4D	500.0	CCCCGTTGGGAAGAAAGC	GATCTGGTTTTCCTTTTTTTTAATCTTA
1747	tarc1375	CoreSet	4D	500.3	CGACTGCTCTGGTCACATACAA	CAGTTGCATTATCGCCGC
1748	tarc2431	CoreSet	4D	502.7	CCTTACTACGTGCTCCCCAGC	TGCACTCGGATTTAGCAGTAGG
1749	tarc1534	Other	4D	505.3	CATCAGGTTGAACTCTCAGTATCTTTT	TCTCTCTCGCACGCACGC
1750	tarc0009	CoreSet	4D	506.1	AGAACTGGCGAGATATAGCACC	TGAAGGCCTAGAGCCCTGG

Supplemental Table 4. List of amplicon sequencing primer sets for wheat.

No.	Name	Type	Chr	Pos[Mb]	F_primer	R_primer
1751	tarc2432	Other	4D	509.5	GTTTGAAACATTTCGAATGATTACG	CAGGTGAGACACCGAAATAGTCG
1752	snp4871	CoreSet	5A	0.3	GAATGTATCTACTGACTCCAGCGAA	CATTAACAGGAATGCGGGAAC
1753	tarc0660	Other	5A	1.2	ACTTCCCAATTGCTTCATTTTTC	GCTCATTCCCGAAGTTCTCAA
1754	tarc0633	Other	5A	2.4	GCTTTCCTTGCTCTGGTAGTGAAC	AGAGAGCGGCATATCCTTCGT
1755	tarc0672	Other	5A	6.7	GCAGCTCCTTGATGATGAAGAGT	GACAATACGATACAGCTTCTGGC
1756	tarc0673	CoreSet	5A	7.4	GGGAGAAGATCAAAATTCTGAGTATG	CACGAATAGAAGGGAGCGAAA
1757	tarc0645	Other	5A	9.7	CGTCACTGAGAGTATCGAGGCTG	CAGATTATAGAATAATACCGTGGACAAA
1758	tarc0649	Other	5A	9.7	CTTTTTTAAATACTTCAGCAGCGA	CGTGGCTTCTTGGGTCAGTC
1759	snp3197	CoreSet	5A	9.8	CTGAAGCAAGCATAGTTTTGTTAGAC	CACCCAAGACAAGCAATAGCTAAG
1760	snp1568	Other	5A	11.1	AAGCTGTTATGTTGGATCTTCTTACAA	CGTCTCTACCGCTGATACACTGG
1761	tarc1857	CoreSet	5A	11.5	GCACGGCCTGACGGAGCT	AATACAAACGGAAAATGGAGATTG
1762	tarc1863	Other	5A	14.8	AACCCTGTGGGACCATGTGT	TATTTATTAGTCTTCCTCTACCTTGTGT
1763	snp1062	Other	5A	20.0	TCAGATCTTGCACGGCAAAAT	CATGAGGTCTGAAGAATTCAACAAGT
1764	tarc0667	Other	5A	20.0	GAGGGTCATTGATTGATTCTACCA	TTAAGAATTTTACGAGTACTAAAGTTCG
1765	snp2378	CoreSet	5A	20.8	GTCATGCAGGCAACGGATC	CCTATGGAAAATGGCACGAATC
1766	snp3365	Other	5A	21.4	TCCTAGCGCCCATCCTTCTA	GGGTAGCGTGAGCACGAGC
1767	tarc1865	Other	5A	29.7	TGAGGGGGAAGAAAGGAGCTA	GCTTCTTCTTCCTGAGCTCATGAG
1768	tarc1866	CoreSet	5A	29.7	GGAGTGGTTTCTTCGTCTAACACAC	GTACTCCATATATAGAAAGGATCACAT
1769	tarc1864	Other	5A	36.0	ACTAAACATGAGTGGATACATCGTTCT	AAGAGTGTTAGTGATCTTTAAGTGGGA
1770	snp3530	CoreSet	5A	36.3	TTACATTTTCAATCACGCCGATA	CATCTTGGGAATGCCTTCTTGT
1771	tarc1859	Other	5A	37.1	TGTTTCCTTTGTTTAACTTTACTGTACA	CTGAGAAGGTAAAAGCCACTCGT
1772	tarc1862	CoreSet	5A	37.6	ACATGAAAAAATATGTTAAGAACGGAG	CATTGCTTATTTACGTTGCCCA
1773	tarc0666	Other	5A	46.7	TTGAAGTAACGGGACAGGACAC	CGGTGACTCGTTATCAAATCG
1774	tarc0669	Other	5A	47.5	GCACTTCGCTGATGGCTGT	AAGAAATGCAGAGTAATATAAACGAATA
1775	tarc0679	Other	5A	48.2	GCGATTTCGTTTCTTATGTAGGTG	CTGCTTGCTAATCCTTGACTGGT
1776	tarc0675	Other	5A	48.9	GGATTGCAGATGGCGGTG	CGGTCCTATAACAGTCCCATCCTA
1777	tarc0678	CoreSet	5A	55.0	ATCAAACAACGTGCCCTTGTC	CTGGAGAATTATACAGTCCAGTTACTAA
1778	snp7226	Other	5A	60.1	AACGTGGGCCACTTTCCTC	GCCGTCAAAAGAAGTTGCACC
1779	snp6237	Other	5A	63.4	TGGAATTGAGAGTATATGTTTACTTCG	TTTATCTAACACGTGCTTGGTTCC
1780	tarc0670	Other	5A	69.8	CTGTATTTCCCCGTGAGGAGT	GTGACCTTAATGCAGCTAGTCAGC
1781	tarc0631	Other	5A	70.0	AAGCAAGAGTGATCAATCATGGTAT	GCTACTGCAAGGCTCCTCCT
1782	tarc0671	CoreSet	5A	98.0	TTGACATGGATGCCACTTGTTT	CTTATAGAAAAGTTTCAAAGTTTATGTT
1783	tarc0643	Other	5A	109.3	ATCTTCCTTCAATTTTATAGGACATGA	AGTCCAGATCATAAATTTTATTTTGTCA
1784	tarc0638	CoreSet	5A	115.1	GGAGATTAGAGCGTGGACTTTTTAA	GGTAATTTTGGACTTACGAGTACCTGT
1785	tarc1860	CoreSet	5A	121.4	AAAATCAAGATGTATGTAAGCAAGAGAA	TCAAATATTCCGCTCGAAAGAAC
1786	tarc1861	Other	5A	129.6	GCACTGATTTATTCGTTGCTCG	AGATGACACGGTGGGCAAA
1787	tarc1867	Other	5A	134.1	TTGAGGGCTCGGCGTCGT	TCCGAATAAAAAACTCTTAGGAATCT
1788	tarc1858	CoreSet	5A	158.2	CCATCTATAATTTTCTATTACCTGCGT	GCAAGGGGAGAAAAGAGGATG
1789	tarc0665	CoreSet	5A	270.8	CTGATACGTAAAAGTACTCCTGGACAC	CTTAATGTGATACTCTTACTGACCTTCT
1790	tarc1838	CoreSet	5A	326.3	GCGATATATTCTCATTTTTTTTGGTT	TTGATCAGAATGTGCTACTTTGATAAG
1791	tarc0680	Other	5A	355.0	TTTTTTTTGTTACCCAACATAAGCT	CACTTGTTTCTTTTTATCATCTCATTTT
1792	tarc0652	CoreSet	5A	358.0	GTTGTGAGATCTGTTTACATGATCCA	TTGCACGACTAACTCTCATTTAATACTT
1793	tarc0640	CoreSet	5A	382.5	TCCCAGGGTAAGTTTGCTTCTC	GTCTGGTATTCTGCACCTCTAAAGTT
1794	tarc0642	CoreSet	5A	384.2	CGATTTGGTTGGTCTAGCTTGTT	ACAATGAGATAAGGTGAAGCACAGA
1795	snp5184	CoreSet	5A	422.1	TCGTACTGTGGATATTGGAGCAC	AGAAAAACATGCATATTTTCCATGA
1796	tarc0676	Other	5A	442.4	ATCTGTAATATGTGGCAAACTAAAGC	CGAAGTACTCAGCCTACATAAATTCC
1797	tarc1846	Other	5A	442.7	ACGGCCCCCTAGTGCGTG	AAAACTGCTACAACAATATACTGCTCTC
1798	tarc1855	CoreSet	5A	444.1	CAAACATCGATCAACTAGAATTTGTTAT	GCCAACTGAAATATTCTAGAGAAAGTAC
1799	tarc1819	Other	5A	444.5	GCAAATTTCGGTTAATAGCTAAAATT	GCAGCTGAAGCTTATGCAAAAA
1800	tarc1836	CoreSet	5A	445.3	TTCGTGTGTGTTTTGGGTGC	CGCCGGAACACAATCAGCT
1801	tarc1853	Other	5A	450.6	CATGAAATATTTTTTTTCCTAGGACAA	TTTAGTATTGTATTTCTGCTACTTGTCT
1802	tarc0658	Other	5A	451.5	AAGTTTTTGTTATTGTGTCGATGGA	GTTGGCTTGTTGCTGGTGAA
1803	tarc0662	CoreSet	5A	457.5	GTTGCCGTGTTTGTGTAGGTAATT	CAAGTATTCCTTAGTGTCAGAATCCATA
1804	snp850	Other	5A	459.0	CGTTATCTGTCTAGAATCGTGAAATG	GAGTACAAAGGAAGCGGTACGG
1805	tarc1828	CoreSet	5A	459.0	ATTCCATGTCATCTCAAGATGTTAGAC	AGGTTCAACTATCTCACATGCTCAA
1806	tarc0650	Other	5A	461.5	GAATCCACTCATAAGAGAGAAGGAAG	TGCTGCATATCTTGCTTATTTCTTC
1807	snp2172	Other	5A	462.9	GAAAAGAAGTAACATAAGGATTAGCGT	ATACATGGGGTTATTCTACAATCAATT
1808	snp8013	Other	5A	462.9	CACGAGGATTGTTGGGATAACCT	GAGGAACTAGACAGTAAGACCACCG
1809	tarc0655	CoreSet	5A	464.5	TGAAACATATGGAATAGATGGACGA	AATAACGGTGACAATGGCAAGA
1810	tarc0654	Other	5A	469.1	GTCAAAAGCAATCTTTGCCTGT	ATCGACTGAGTTTGCACCCC
1811	tarc0635	Other	5A	471.9	TTCAGAGATAATTACCGCAATACAGA	GATAAGAGCAAAGACATATCTTGGTG
1812	tarc1832	CoreSet	5A	473.6	TGCACTGTCTTATTGATGGAACAA	AAAGAAAGAAAGAGGGAGAAAAGC
1813	tarc1847	Other	5A	473.6	CATATTCCTCTACCAAGCATCTCTAAC	ACGAGTACACACACCTCATAGCAA
1814	tarc0677	Other	5A	476.7	ATCATACTTGAATGAAATATGGCTACAG	GCCTAAAGAAACCTTACATTACCTACT
1815	tarc1843	Other	5A	477.2	CTGCACGGCTCAGCCTGG	ACGGGATACCAGAATTGGCA
1816	tarc0636	Other	5A	481.9	TATTATTTAGTTTCTTGAATAGCAGTGT	ATCCACCATAACCTTTTTATGACTTT
1817	tarc1826	Other	5A	482.1	TTCCATTCACGGGCTCGT	TTTGAAGTTTGAAACAGTAGATAAAGTG
1818	tarc1849	Other	5A	491.6	CATTCATAGCCATGCCTGTAGAGT	CCACACTTTGCTTGCCCC
1819	tarc1841	Other	5A	492.9	GGCTTCATATCAGCGAGCAAAT	ATGAAAATTTCTGGAAAAGAGCATA
1820	tarc1852	CoreSet	5A	495.3	GCCTACCGTCTGCTGCTAATC	GCTTGGTAATTTGTCTTCCTTTGT

Supplemental Table 4. List of amplicon sequencing primer sets for wheat.

No.	Name	Type	Chr	Pos[Mb]	F_primer	R_primer
1821	tarc0639	Other	5A	495.9	GAAGTCCAGTCGGGTTGGTAA	GCATAGTTGCTAAAGGCGTAACA
1822	tarc0663	CoreSet	5A	506.1	CAAAATTTGTATTGATTTTACTGGGAA	GCACACATTGGTACAAAACTAATAAGT
1823	tarc0656	Other	5A	508.9	CAGAACATTCGTCAGGAAATCAG	TATATTCCACTTCTCTTTGCAGGC
1824	tarc1844	CoreSet	5A	510.9	CAACTTTGTATGCTAGTTGTTTGGG	TAAAAGATGCAGAATCAGGGATATAGT
1825	tarc1831	Other	5A	512.8	CAAAAATGAAATGTCAGAAAATTGC	AGGCAATCAAGGTTCATGAGACT
1826	tarc1850	Other	5A	513.8	TGTGCAATCACAATTTGACATTG	GGCTATTTTTTTGGGTGCATCT
1827	snp1469	Other	5A	520.0	TGTGTATGAAGGAATTCTTTGCG	GCTGCCATATCAGCCTACATTC
1828	tarc1827	CoreSet	5A	521.0	AAATCGTAATCCAATTGCAAAAA	CCAAGACTCTGTTCGGCTATCC
1829	tarc0664	Other	5A	524.1	AATCATCAGCTTCTATAGTAATTTAGTC	GAGCGAATCCTCCTCCTCCT
1830	tarc1822	Other	5A	533.3	AGTAGTACTGTGTTGAACAACTCTTTGA	ACCTTCTTCTCGAAGCACCGT
1831	tarc1856	CoreSet	5A	533.9	AATTCTAACTTCTAATGCTGTCATGAGA	GAACAAATGGTTAGTTTTGGTGCT
1832	tarc1837	Other	5A	535.4	TTTCAAGATTTTCCCTATACAGTAACAT	CATTCTGACGGGTTCTAATGTCTAC
1833	tarc0647	Other	5A	535.6	AAGATGATGAGGTAATTACAGATGCTAC	AGCTTCCCTCTCTTCCAACAAT
1834	tarc1848	CoreSet	5A	535.6	CGGTCAGGTATCTTGGAATTTTCT	CCAATTAAACACGCACACCCA
1835	tarc0651	CoreSet	5A	536.7	GTACAGAATTATTCAGGAGTTCTTTGG	TGATCTGATCTGATCGGCGG
1836	tarc0653	Other	5A	537.1	AGAACATAATTCATCCATCTTAGGTAGT	GAAGGTTCAAGTGGCAGCTCT
1837	tarc0648	CoreSet	5A	549.3	TTTCTCCTGCAAAGTCATTCGAT	TACAGTGTCAGTAGTACATACAATCATC
1838	snp6456	Other	5A	549.4	CTGATGATGTGAGCCCTGAAAG	CAGCAAAAATTCAGTACGAAATG
1839	tarc0630	Other	5A	555.5	TTTGGTCTTGCTTCTTTGTATTAGATT	AGTTGTGGTTTTGTGGATCTGTTC
1840	snp4980	CoreSet	5A	565.4	TGACTTGGGAGATTTTGATTCGA	GGATACACGATCACGCTGCTG
1841	snp5945	Other	5A	567.9	TTGTCAATTCAGATATGGGACCTAG	GAGAATATGAAGCGGTCACTTGA
1842	snp4390	Other	5A	569.5	TAGTGGCAATAACACAGGTAGCG	CTGGTCTTGTGTTAGGGCATG
1843	tarc0634	Other	5A	569.7	TGGGGGAGGAATACAACTGG	GGAATGGTAGCCGCCCTTT
1844	tarc1842	Other	5A	569.9	ATGAGATGGAAAATTGCATTGG	TCAAGGCTACTTATAAAATTTATGTTGT
1845	tarc1829	Other	5A	570.9	GAAGCTGATGAGGCGGTACG	TGGTATGCTCATAGAAGAGATCATCAC
1846	snp5623	CoreSet	5A	571.8	TCACGCAGCGGCAAGTTC	AGAACACACTCATGAAACAAATGGA
1847	snp3625	Other	5A	580.9	TGTAACTCAAAACCATATTCAACTACG	GGAAGGTTCGCTTTCATCCTC
1848	tarc1823	CoreSet	5A	580.9	AAATGTAGTGTCAAGATGATGAGTGC	CCCCTTCTTCTGGGGAGTTG
1849	tarc1834	CoreSet	5A	581.3	CCAATGTCACTTATCGGACTTCA	TAAATTAGTCTGCTTCAGCTTTTTCA
1850	tarc1835	CoreSet	5A	581.3	TTTCAGCATCTGCCTATTCTTTTAA	GTGCTTTTAGGTACTTCTGCTCAG
1851	tarc0644	CoreSet	5A	584.4	CCCAACTTTTTCTACCAACCCA	CTTCATGACCAGCATACCGAG
1852	tarc1825	Other	5A	585.4	TGTTGCCTTTGCATCATTCTTC	GGCTATTATTTCAGGATCTCGG
1853	tarc0657	Other	5A	585.5	CTTCACCGCAACCTCCAGT	TTGGTCCTCACGTTGTCCG
1854	tarc1821	Other	5A	586.1	TTCATGGGATGCAAAGAAAATAA	TGCAATCATGACCTAATAGAGCAA
1855	tarc0628	Other	5A	588.6	GCTTGGGGTACATCAATGATCG	CACAATGCCACCAGACACCAC
1856	tarc0661	CoreSet	5A	595.4	GCTTGCCTTTTAGCTGTACTGAAAT	AGCCTGAAACCCCAAAAAAG
1857	tarc0659	Other	5A	611.1	CTATAGGTAGCAATTGGTGCTTCTTT	TCCTCATGTGTTTATAATCAGGGAC
1858	tarc1839	Other	5A	611.3	TGCAGGACAAACGAGCACAAT	GGAGCAGTAGGGGCAGGAG
1859	snp583	CoreSet	5A	617.4	TTTCACCGCTCATGTCAATACG	GAAAGTTCACTTTTCAAACTCCTACG
1860	tarc0674	Other	5A	623.0	CAGGTATGTTGCTTCTTGTCAGTTATA	AATAATCCAGTAAAACTAGAAGTACAGA
1861	tarc0668	CoreSet	5A	635.9	TCCACTGAACTTGTCTGTTCGTG	CAACCAAATAAATCTAACTACATGCTGT
1862	tarc0627	CoreSet	5A	657.0	GCAAATCTTTAAGTTCCGTGGC	TTTGTGCTATATTATTCGTCTTCTACTT
1863	tarc1851	Other	5A	657.5	TGCCATTCATGGTAGTAACAATCA	TGGCTGTGCAGATGTTGTCG
1864	tarc0641	CoreSet	5A	662.5	ACGTATGTTTCTTCCTACTATCTCGG	GCATCGTCGTTCTTCTGTCCT
1865	tarc1824	Other	5A	662.5	TCCACAGTTCTGAAAAGAAACTAATAGA	TCTTGTTATGACTGAACCTTTTGCT
1866	tarc1830	Other	5A	663.4	TGTTTTTCAGCTTCACAGCCAG	CATGTCCTTAGACGCGACATTG
1867	tarc1833	Other	5A	664.5	TTTTCCAGGTTTGACTCTGTAAGC	TGAGCCCGCAAGAGACAGA
1868	tarc1845	Other	5A	665.6	TGTTAAAACTGGCAGAGATTCAGC	GCGCTTTTTAATTGCATTTTAATAT
1869	tarc1854	CoreSet	5A	666.7	TTCCTTAGGTTACCATACATACTGCA	CATAGTACATTGTTACCAATAAAACGTG
1870	tarc0632	Other	5A	667.1	TGCTTATTTTCAGTTTTGTAAAATCTTT	TTCCTTTCCAGTACCTCCAAGTG
1871	tarc1840	Other	5A	667.1	TCAGGTAGCCCCAATTATTCAAA	CATGTGGATTGCCTGAGGAAA
1872	tarc0646	Other	5A	667.3	AAGTAAATCACACAACTGTTTAAGCATA	GGAGGTAGGTCCGGGTCG
1873	tarc1820	CoreSet	5A	672.4	CTCACACCCACCCATGTAATG	GTGGCTGTAATAGAAACACATAGGTC
1874	snp7162	Other	5A	673.8	GACTGACCAGAACATATAGCGACA	GCTTCCGTTGCATCCATTTATC
1875	snp2802	CoreSet	5A	677.1	CTGGCGATGGTGCAGGTT	AAGCCCTGGGTTTCTGCTG
1876	snp2947	CoreSet	5A	688.3	TGAATTATGCAATCCTTGTGGTG	CCCCTTCGATCGACATGC
1877	tarc0629	Other	5A	689.6	GGAGAAGCACGGGGACTG	GACAAAACAATCAGTTGACGACG
1878	tarc0637	CoreSet	5A	689.9	TGCGTACATGTTTTGGTTAGGAATA	GGACAGAAATCAATCGCTAAAACC
1879	tarc1090	Other	5B	0.2	CCCTTTGCATGGTTCACGA	TTTTCGCTCTCTTCATTGTTATACG
1880	tarc1096	Other	5B	6.4	ACAGTTGCACCAGCACTGTGA	CATGTCAAATAGGTTGCTGTTGC
1881	snp757	CoreSet	5B	7.7	GATGAGGAGTACATGGGCACA	CACATACGGCACCATGCCT
1882	tarc1083	Other	5B	10.4	AATATACAGAGCATCAAATGAGAAGAAT	CCAAGTTTGGACAAACAGGAAGT
1883	tarc1086	Other	5B	13.7	CAGGTCCACAACACTATGTTACAAACT	ATTTGTTCCCATTTTATCCGTATCT
1884	tarc1117	CoreSet	5B	13.7	CATTTTTCTGTAACAACTACGGCTTT	ATACTGGCTCATATCACTGGATACACT
1885	snp3606	Other	5B	14.5	ACAAGACCTTGCTTGTACTCCTG	AAAGAGCTACCACTGCACCTAGAG
1886	tarc1079	Other	5B	15.3	TACTATGTTCTGCATTTTTACTGTCCA	GTCATGCCCACTTGCTCCTT
1887	tarc1100	Other	5B	19.4	GAGTAATAGAAAGGAAAATATGCCTTAA	CAGAAATTGAGGGGCACAGAA
1888	snp332	CoreSet	5B	20.8	CTCTGTTAATGATCAGCGTGACAG	CAATAGCAGTGGGATAGAACAATATC
1889	snp7211	CoreSet	5B	26.8	TTGTCAACTAAGCTATCCAGCTCTAGT	TTGCTACCATTATTGGGATAAATAATC
1890	tarc1098	Other	5B	26.8	AGGAGAAAATCCTCGCAACAGT	ATGACATTTTCAGACGAGACCAGT

Supplemental Table 4. List of amplicon sequencing primer sets for wheat.

No.	Name	Type	Chr	Pos[Mb]	F_primer	R_primer
1891	snp1143	Other	5B	27.0	TGTGGAACTGAAGAAGAAATCCG	TGGTAGTCAGACTTATTCATGTTCATG
1892	tarc1111	Other	5B	27.8	GGACACATGGATGCAGGTGG	CCTGTGGGCAGAATGGATAGA
1893	tarc1105	CoreSet	5B	35.5	CATACAGATTGTTTCCAACGCCT	CCTATCAATTATTTTGTCTACGGAAGT
1894	tarc2228	CoreSet	5B	38.2	ATCAAATTTGTGTTTTCTTGCTTTG	GATTATAAAATGCAGTTGGTTGAGTAAC
1895	tarc1080	Other	5B	47.7	AGACAAGCGGCAAACTGAATG	GGCAGAGCCGTAAATGGG
1896	tarc2227	Other	5B	54.7	GAAGTTCATGAGAAAACCAATGACT	AAAACTACTTTTTCATACTCTGTTAGTG
1897	tarc1084	Other	5B	55.2	CAGACAAAAGCTCTTGGTATTTCTAAC	GCTAAAGTGGATGCTCAAAGTGC
1898	tarc1119	Other	5B	63.4	GTAACACTACCAATATGTTTTCTGGTGT	GTGCAAATGTCCGTGGCG
1899	tarc2226	CoreSet	5B	63.5	ATGGTTGACTATAGTTATTCAATAATGT	ATGCACAGACTCTAAATTCAGTGGA
1900	tarc1124	Other	5B	70.3	CAAATTATATGGGTGATCTTAGGTCC	CTGATCAAGAATATGTTACCAACAGC
1901	tarc2234	Other	5B	73.5	CAAGCTGGCTTGCCATTATG	GCTGTTCTTATCAGCATTATGTGTTT
1902	tarc1126	Other	5B	75.2	TTTGCACAAGGAAGAAGAGTCG	CCATCACTGCTTGCGGAAA
1903	tarc1087	Other	5B	75.9	ATCAGAAATTTTGGGTGTGAGGT	GCCTTATGTGTCCTTCGCTCA
1904	tarc1121	Other	5B	102.3	CAAATGAAATCTGTCTAGAGAAAATGC	GAAATCTGATGGCTCGAACG
1905	tarc2233	CoreSet	5B	102.3	GCTTCTGGAGAAGTTGTCCATGTAG	CCAATAGGGCAAAATATAAGGACTCT
1906	tarc2230	Other	5B	103.3	AAGTTTATGCAACAGTGATGAACCT	GGCATTGTCTAAACGGGATACG
1907	tarc1114	CoreSet	5B	118.5	CACATTCACCTCAAAGAAACCAA	ACCATTGATGGATGTGTGTGTGT
1908	tarc2229	Other	5B	121.0	TCAGGAATTTTCGCCTGTAACA	TGTTCTCTAGTTAATACTGATAAAAGGT
1909	tarc2232	CoreSet	5B	122.8	GGTCTATTGTTTATTAAGTGACGACTG	CTCAAGAACAAAAGCAACATCAACT
1910	tarc1122	Other	5B	130.5	TGAAACAAATAACAGATACCAAAACACT	TTGATGCCTGAAAGATATAATTGAAG
1911	tarc1078	CoreSet	5B	131.3	TGGCGGTAAGCCCCATTAC	CAGGGTGGAGGGTATGCGT
1912	tarc1120	Other	5B	133.0	CCAGTTATCCCCAACCGTCA	GACAATTATTTCTTTACAGAGGCACTAC
1913	tarc2231	CoreSet	5B	139.2	TATGTATACCTTGCTTGAATGTCTTAAC	GGATTTTAAAGGAAGAAAATTGTCA
1914	tarc2225	Other	5B	162.8	AGAAGGTGTGATCATTCTGAATATCTC	AGTTAACGCTAACATTATTGTTGAAAAG
1915	tarc1085	CoreSet	5B	187.9	CTCCAGGCCCTATATAAATAACATTA	GCGTTCACCTTAACCAACCTCTA
1916	tarc2591	CoreSet	5B	293.5	TCTGCAATAAACAATTGCGTAGAA	CTCCTGGCTCTCATGTAAGATTTTA
1917	tarc1110	Other	5B	295.2	TAAGAATCAAATTCCCAATATTTTAACT	GAAGTTTTTGTGTACTGTTATTCACTCT
1918	tarc2222	CoreSet	5B	295.2	CAATATATTACCTGCTGAATCCTACTAG	TGCATTATCATCACAGCACTTTACA
1919	tarc1132	CoreSet	5B	302.1	GCACCCCTATTGTAGTTGTCCAGT	GAGATTTTGCAGCTTTAATTGGTAGT
1920	tarc1131	Other	5B	309.7	CTGGAAGGAGATTCATTTATCTTAGATC	ACCATAGAGCCAAACATGTCAAAT
1921	tarc1112	Other	5B	313.1	GCTCGAGGAATCTCTAGGATCTG	TGTACACCACACAATAAAAAACAACAA
1922	tarc1123	CoreSet	5B	357.1	CATGGATGCTCCATTCAATAGTG	TAAATTATCTATGTACAACTACCTGCTT
1923	tarc1109	Other	5B	380.3	CATGTCCTGGTTATCAATTATCCTGTA	TACACCATTAGAAAACAAAAATCAGC
1924	tarc2198	CoreSet	5B	380.8	GGTCCTAAAGTAAATTAGGAGTGTCG	TCATCTTCACGCGACATCCTT
1925	tarc1097	Other	5B	393.8	ACATGTTATTCTGAACTGCGTCG	ACCAAAAGAGGAACGAAGCG
1926	tarc1094	Other	5B	395.0	GAACAAAACTAACTAAATTCAAAAGATA	GAAAATGGCTGTGGGAAGTTTT
1927	tarc2188	CoreSet	5B	397.8	CTTCAAGCAGTGGAAACTGAACTAA	GTGAGAGTCTTGACCTTGATCATTG
1928	snp6689	Other	5B	404.2	GGTTTGACTCTCTGTGCGCTTA	AGAAATCTCGCGAATACAGCAA
1929	snp7127	Other	5B	404.2	GGTTTTTGTGCCTTACATTATCTCAC	GGCAGAATGTGCAGCCATG
1930	snp123	CoreSet	5B	410.5	GCAAGAAATAAGCCATTCTTATACG	TGCAAATGGAAAAAATGATGTTTA
1931	snp301	Other	5B	411.9	ATTTGATGCTAATTTGAATGCAATC	GATGGCACAGCTCACAGCG
1932	snp4422	Other	5B	415.6	GAAATTGAAGAGACAGATGAGCCA	CTCGTAGTTAAACATGGCCAGC
1933	tarc1081	Other	5B	425.9	TTCAAGCACTACAAGCATTACCCT	ATGTGATGCGAACAAGTATCTAATAGTA
1934	snp5742	CoreSet	5B	431.1	GCTGCGGTTTTATTCCTCCA	CATCAATCGGCTCAATAAGGTAGA
1935	snp5845	Other	5B	431.8	AGCAAATGGTTCCTCATCGG	ACAAGGAATCAGATGCACCGTA
1936	snp721	Other	5B	432.2	AGAGCTACCGCACGCAGC	TGTTTTGTTTTGCTTTTACTTCGA
1937	snp7210	Other	5B	432.2	TCGTATCTTTCGATGAACTTCTCAA	TGGATAGCTTAGTTGACAACTCGTT
1938	tarc1136	Other	5B	434.6	TTACGCAAAATGTCCGTGTCTT	TATTTATATGTGGCGCAGCCTC
1939	snp2181	CoreSet	5B	455.7	CCTCACTGCTGGTTTACCGG	AGTCTAAAAAGGTTGGCACCG
1940	tarc2211	Other	5B	461.3	TGCCGTGGAGCCTGTCAA	AGCTTCCATGATTTACCGCC
1941	tarc1127	CoreSet	5B	462.1	AATCGGTAATAGTTATGGATGTGACATA	GTTCAGATGCTCTGCCGACA
1942	tarc2200	CoreSet	5B	462.1	AACTGCTGTTTTTAACTGATGGAGA	GTTATGGATGTGACATAAAACTTCTTAA
1943	tarc2602	Other	5B	469.1	GAACAAAAAAGCTTAAGCTTCACAAT	TGCCGTTCTTCTTGAAACAGG
1944	tarc1129	Other	5B	476.6	ACATAGTACACTATTTTTTAAATTCCCG	AAGCAAGTTCACATGGGAAAAGTA
1945	tarc2196	CoreSet	5B	476.6	AAAGTACATTCTAAATCACATGACAGTA	GTGGATGTAGTTTGAATCTCTGGTAAT
1946	tarc2212	Other	5B	476.8	TCAGATGCCTGCGAGGAGAG	GGTCAGAATGCTGCATTTGG
1947	tarc2199	Other	5B	477.1	GAGTTCATATCTGTCCATACTTGTTCAT	ATTGCACCATTCCAGTCTTTTG
1948	tarc2187	CoreSet	5B	477.7	CAAGTATGCATGGATGCTATAGCTTA	CCCGTTGAGCAGCAGGATA
1949	tarc2600	Other	5B	478.5	TGTTAAATGTGTAAAAAAAGTTCTCTCA	AAAACTAGGTGGCGTGCCTG
1950	tarc1118	Other	5B	479.3	AAGAAAGAAACAGTCAGCACATGTT	GAAATGCGTGGTTTCTGCG
1951	tarc2191	Other	5B	483.0	GGCACAACATACTTACAATATATCCAAA	TGTTTGAAGAGACATCTTGAGAAGGT
1952	tarc2209	CoreSet	5B	485.9	GGTGGCAAGCCAGGTAGGAT	GAAGTCCAGTTTGATTCGTACCTCTA
1953	tarc1133	Other	5B	486.1	TGAAACTCCCCCATTGTTGC	GGATTCAGAGTGATGTTCCCTCT
1954	tarc1088	Other	5B	486.7	CGTATATTTTACAGATAATCTTGGCGA	GCTGGACTTCACACTTCACCCT
1955	tarc2213	Other	5B	487.6	GACCTGATAGAAATACAAGGGGGA	TCTTGTGCTCATGTAATATCATCTGC
1956	tarc2223	Other	5B	510.4	CTATTTTTGGTGTCCACATGCTG	GTTACTTGTCTGAACGAATTTGCTG
1957	tarc1093	Other	5B	515.2	GCGTTGTGTGCTACCTTTATTTGT	GCGACGGCTTTGTAGGTTTGT
1958	snp2373	CoreSet	5B	515.6	GCCTTGAGGAGCGAAAGTGTATAC	TGCACACGGAATCCTGAGC
1959	tarc2219	Other	5B	517.9	ACCTTCTTCGCTGCCATCTC	CTACATATACATGCGCGTCCATC
1960	tarc2190	Other	5B	519.1	CCCAACATTTTCGGTGAGTTTA	GTCCATCAAGCTTACAGTGCAAGT

Supplemental Table 4. List of amplicon sequencing primer sets for wheat.

No.	Name	Type	Chr	Pos[Mb]	F_primer	R_primer
1961	tarc2208	Other	5B	539.3	GCAGGACTTGCTACACTTTCCTAAA	AATGCCCTGAATCAAGTAAGTTTTT
1962	tarc2217	CoreSet	5B	554.2	CGTCTTCACGGATAGAGTACTATGC	ATTCTTAAGTTCCGCAACAAGGTA
1963	tarc2593	Other	5B	554.5	ATCACCTCCTCCTGTTGCTCA	TCGTTATCTTTGTTTGCCTTTCA
1964	tarc1134	CoreSet	5B	557.5	CTAATTCTTTCATGATATGTGATATACC	CAGGTAAAAGGCGACAGATGAC
1965	tarc2205	Other	5B	557.8	AACAATTCTTTTGGAAAAGGAAGTG	CAAAGGTGCCACGATGAACA
1966	tarc2597	CoreSet	5B	558.3	CAATCTGCAAAAGGAAAGTGCTA	CGGTCAATGACAACGGCG
1967	tarc1077	Other	5B	558.6	ATTGCCATCCCTTAACTCACG	GAAGAATGTGCAACAAAAACTCG
1968	tarc2590	Other	5B	565.8	CAGCTGCCAGTATTTTTTATCTGATA	AAAATCCTACCATGACAAGTACTCCTAT
1969	tarc2192	Other	5B	567.2	AATTAGTCTGCTTCTGTTTACAACTATA	CTGAAGATCTCGTCAAGGGTGT
1970	tarc2193	Other	5B	567.2	CCAAAGTAAGAAAATCACAGTAGCA	CTTGATACTAGCATCAGCATATGAGC
1971	tarc2186	Other	5B	568.4	CCCTGCCTTGATGTTTGAGA	TGCACACAGTTTCCAAGTGAAGT
1972	tarc2592	Other	5B	570.6	GTCATGTATGCTCCAACTATGGATT	GCTACTTTCTTTAATACAGAATACCGC
1973	tarc2202	Other	5B	570.9	TCAGGTGTTGGTAACCTGGTAACTA	AGGCATAGCATGTCTGGATGTACTA
1974	tarc2596	CoreSet	5B	570.9	GCATCAATATCTACATTGAGCATTTTAC	CACCAGTCCAGCCACAACAAC
1975	snp6718	CoreSet	5B	572.4	CATTTGTATTTCAAGATGGATTATCG	CGAGAAAGAGATCGATATGAATTGG
1976	tarc2221	Other	5B	577.3	GGATGTAAACAAATGTTTTCGTGA	TAGTTAATCCTTAGCAATCTTGCGT
1977	tarc1116	Other	5B	577.4	AGCAGAAAGGCAACCTAAAACA	ATCATAGGTACACCTTAACTGCTCAAT
1978	tarc2216	Other	5B	577.4	AATATTACCCCTCTGTCCTCGC	TATTAACTGTCAAGTGCAGTAGTTTCTA
1979	tarc2594	Other	5B	580.1	TGATGTGGTTCTGAACCAAGATG	TTCTTTTGCAGAAAGGGCAATA
1980	tarc1082	Other	5B	580.7	GCTAGTTATACAGAAGCATCCCAGAC	CCATAGTATGCAGCATGGACAG
1981	tarc1108	Other	5B	581.1	CCTACAAAATATACCTGGTGCATTATA	AAACCTCTTCATGTAGCTCAACGA
1982	tarc2599	CoreSet	5B	584.1	TTAACCTGAGATTGTTACCTTCTTGAC	TGCAGCAATCTTCACAGCTACTTA
1983	tarc2207	Other	5B	587.1	TCTCATTCACCTGAATACAAAAACC	AGAAACTGGCGAGAATGTTCAAG
1984	tarc2194	Other	5B	588.2	GCATCTCGGAGAAGGCAGC	TGTTTTAGTCTTGTTGGTTGGTCG
1985	snp3800	CoreSet	5B	589.4	CAGGGCTCACTGAGAGCTGTT	TATGGAACCACCCGTCTGAGTA
1986	tarc1107	Other	5B	589.4	ATGATAAACTGTTAACCGTGCGA	TATGGAACCACCCGTCTGAGTA
1987	tarc2195	CoreSet	5B	589.4	TTTAAATAGTTCGTAAGTTGTTTTTTTC	CCCTTTGGAGATGCAGTAATTTC
1988	tarc2598	Other	5B	590.0	GGAGAGTGATACTGCTGCATCTCA	AGGAGCATTACTCCTTTAATATAAAGAA
1989	tarc1128	Other	5B	597.9	GTTCAGAATCATAAAACCCTGATACAT	ACAAATGGTTAGTTTAGTAAAAGATAGA
1990	tarc2201	Other	5B	597.9	ACACCAAAGCGGTGTAATGTAAGT	CTGTTCAGCACGTAGCGTCAA
1991	tarc1089	Other	5B	600.5	GATGTTTCGTTGATCTGGTGGA	TCATTCAGTAGTTTCCCCATTGTTAT
1992	snp7014	Other	5B	601.3	AGAGATAGCACCAACAAAATGACA	TTCTATTTTAGTGCCAAGAATGTAATTC
1993	tarc2595	Other	5B	601.3	CTTCTCATGCGTTATGCTGAACT	TGAGATGTAAGAAAGCATTCACAACA
1994	tarc2220	CoreSet	5B	601.4	AACATGCTCTAAGAGAGATATGGACAA	GAAGAGGATATACAAGCTGATGAAATC
1995	tarc1095	CoreSet	5B	630.0	TAAGTCTTTGGAGAGATTCCAATATAAA	GGTAAAATATGTTGAACTTGTAGCCTT
1996	snp2388	Other	5B	636.6	CATCATCTGACCAGCAAACGA	TGCCTCAACTGTTGGCTGTATG
1997	snp4762	Other	5B	643.7	ATCACGGTCGTGGTGTCGTAT	TCTAAAACATTAAGGTGCCAGGG
1998	tarc1092	CoreSet	5B	643.7	GGCGACAAACACAACAGCG	GAGCTACAGAGAGTCAACTGACCC
1999	tarc1101	CoreSet	5B	653.7	TCGGGGCTTTTAATATTTCCTT	AAGACTTAATTACATTCAATCCGTTGTA
2000	tarc1125	Other	5B	654.5	ACTCCTAAATCATTAATTTTCTTGTGTC	AAAACTGGAGATGCAACAGGAGA
2001	tarc2203	Other	5B	662.5	TTTGCCTCACCCCTTTATATGTC	ATAAGGTACATAAAGAACATGCACCA
2002	tarc2601	CoreSet	5B	668.2	ACTTTATCCAAGTAAATGATTGACTTAT	AACCTTTCAATATTATAGAACACCAATA
2003	tarc2204	Other	5B	668.5	TGTAGCCATTGCAGAGTATAGATTGTA	GCTGAGATCCATGCACCCA
2004	tarc2603	Other	5B	669.5	TTGATTACAATTTTACAAGACACTGGA	ACTCACTAATTGGAGTTGGAGTCG
2005	tarc2224	Other	5B	670.8	CACATGCCACTTCAAAAATACAGT	GTGGGCGTGGTGGCTCGT
2006	snp6902	CoreSet	5B	680.9	AAAGAAGTCGTGTCGACTAGTTTTC	CAGTAATGTTAGTAGGTAATTCATCCTCA
2007	tarc1135	Other	5B	682.7	ATGGGTCTTGTTCACATTGCTG	TTGTGTTACCTTGAGAGAGGGAGA
2008	tarc1104	Other	5B	682.8	CTAGCTAACGACCAGAAAGTGAAGT	TGCGGTATTTGGAGTTCACATAGT
2009	tarc2218	Other	5B	682.8	GTCGAGAATGATGTCATCCCCT	CCTTCACGTTCCTTCACCAA
2010	tarc2215	Other	5B	687.8	TCAGCAGGTTTCTCCAATACTCAC	AGATCTGAAGTTGAAGAGGGGTG
2011	tarc1091	Other	5B	692.6	CTTTTTACACTGTCTCCACTTTTCC	GAAGCTACTTGAACGAGGCAGAT
2012	tarc1099	Other	5B	692.6	AAGAAAGAGGACAAAAAACGTGAAC	GCTTCTTCAGGTTTTCATCGG
2013	tarc2189	Other	5B	696.2	CACTATTTCTGATCCTACAATCTCCA	TGCATCTATCTGCTTGATTTACATATCT
2014	tarc1130	CoreSet	5B	700.2	GAACTGTAGTTTTTAATATTTTTGAGAC	GGGCACTGGCAGAAGCACTAT
2015	tarc2210	CoreSet	5B	700.2	AGAACATTGAGTGTTCCATTGACTG	CGCGGGTTCTGACTGTGTGT
2016	tarc1115	Other	5B	701.5	TAGATATGTCTGGTGTTGTTCATTAGTT	TGTCACAAGCCAACCCATATAGTAA
2017	tarc2197	CoreSet	5B	701.5	ATCTCTGCTACACTACACACAGAAATG	GCCAGAGAGATCTGTAGGATTCGA
2018	tarc2214	Other	5B	702.9	CATTATTGAATAGCTTCAGCCTATGA	ATTCTAGATGTCGATAATTTTTTCTATA
2019	tarc1102	CoreSet	5B	706.2	GTTTACCACCCCATACTCTCCAG	TGTCCAGCACAATGTTTTTCTTCT
2020	tarc1103	Other	5B	706.3	AAGTTGTTCCTATATCATCTGCTGAGT	TCAAAACGATGGCATATAAAGGA
2021	tarc1106	Other	5B	707.1	CTGTCAATCTCAGGGAAGCGA	GGAGCAACGAACAATGTGAAAT
2022	tarc2206	CoreSet	5B	712.7	GGGGCATCATGTAATTATCGC	TGGCTCAGGACGTCGTCG
2023	tarc1504	CoreSet	5D	0.6	ACACGGTGGATGGGGTGA	CCAATCCAATAATAACAACAACGA
2024	tarc1528	Other	5D	2.7	TCAATGACATAGTGACTTAGAAACACTT	CGACCCACAAAATTAGACGACG
2025	tarc0280	CoreSet	5D	5.8	AGTAACACGTAGAGCTGGGAATCA	ACTGTCACAGATTCAGGAAAGGG
2026	tarc1514	Other	5D	7.0	GGTAACCCACCTCAACTTCTCTCA	TTAGACAAGAAGAAGAAAACAAAACAAA
2027	tarc1478	CoreSet	5D	8.5	GTGCCTGCCAACATCCTATG	CATTTGGCCTATTATGCCTAAAA
2028	tarc1368	Other	5D	9.1	GCGAGCTTCTCCGCCAAG	CATGAACGTCAGCACAATAACATCT
2029	tarc1438	Other	5D	10.5	CCAGATCATCTGCCTCGGTGT	CAAAACAGGGATAACAACTCAACG
2030	Inf18112	Other	5D	11.4	CGGGATGTCATCTGTAGTGACG	GTCCGGGTTCTCGACCAGT

Supplemental Table 4. List of amplicon sequencing primer sets for wheat.

No.	Name	Type	Chr	Pos[Mb]	F_primer	R_primer
2031	tarc1386	Other	5D	28.9	GTATAGTACGTGCAGTCTCGGTCTT	CCTGCTTCTGCTCCCCAGTA
2032	snp8360	CoreSet	5D	29.2	ACCATAGTCGCCGTCTCCATT	TGCAATTTCCTAGACTTACTTGCA
2033	Inf63046	Other	5D	29.2	TGAATTCTTCAGATCCCATGCC	GGCCGGACCCACAGCTTG
2034	snp6289	CoreSet	5D	32.7	GCTGGGATGAAATATATTTGAAGTT	CCTCAAGGGTAAACCAGTATAACG
2035	tarc2467	CoreSet	5D	33.0	GAGCGTGCAAGATACATTCGG	TGCACCAGCCTAGTCAATACATG
2036	tarc1537	Other	5D	42.9	ATAAGCTAAAGGAAACAAGAATACAATA	GAATGTAGCTGCTTTAATATACAATTTC
2037	Inf16210	Other	5D	43.2	CGTAGAGAAAGTTCTTAGATGTACATGA	GCTTACCTTGAGAACCCCATG
2038	Inf4461	Other	5D	45.7	GGTACAGTTCCTGCATCAGTCTTGT	AAATGATGAGGATGTTGAAGCTAGAC
2039	tarc0284	CoreSet	5D	46.1	GGTCGATAGTATGTTTAGGCTTCCA	AGCGGTGGTTGCATAATGTGT
2040	Inf58711	Other	5D	50.8	GTGTACCGGGTTTTGGAATTG	TTAAAGGATGATGAGCAGCTTACTG
2041	tarc0285	Other	5D	52.1	GTATCTGAGGAATTTTTCAATATGGA	ATCACAAATGTGTTATTTGACTCTTCA
2042	tarc1395	Other	5D	58.3	TGCTTCCATGCCCCTCGT	AGATGAACAGAGCCGGTCAAA
2043	tarc0282	Other	5D	60.6	GCCCAGCTGCTAATCCAGTT	TTTGAGGAGATAACCCTCCTTTTA
2044	tarc1389	CoreSet	5D	66.8	CTGCTTGTTCTTCCTTTTTTTTT	TCTTCAGTGGAACACCATTATAAGTTTA
2045	tarc0286	Other	5D	93.1	TGGTAATTACTTTTCAGAAGAACAGAC	TTCTGGCAGGAATATACAGTACAGGT
2046	snp5366	Other	5D	107.6	TTCTGAAATTTGTAACTTGTTCCTTTT	TTAGATGTGTAACTACCTCAACTCCTAATC
2047	tarc2468	CoreSet	5D	107.6	AGATGTGTAACTACCTCAACTCCTAATC	TTCTGAAATTTGTAACTTGTTCCTTTT
2048	tarc2466	CoreSet	5D	108.5	CTTGGAGTATCCATCAATCAGGG	ATGCTACGGGATGGGCTTT
2049	snp6268	CoreSet	5D	160.1	GCTAGGCGCTCACCAAACC	AAGAGAATTGGGAAAGAAGAGACG
2050	tarc0281	Other	5D	184.0	CGATCATGGGACCACTACTATTACA	GGTTCTAACCCCATGTCCCA
2051	snp6052	CoreSet	5D	188.3	CCAATTTGACTGCAAGTTCCG	AGATGACTGTGGGGAATAACCATA
2052	snp4550	CoreSet	5D	192.3	GCAACAAATATTGAAAACCTTTGAG	GCAAATTGCAGCGACAAAGTG
2053	tarc2457	Other	5D	197.1	AGTCGAGCCTCCCCTTGC	ATGCTGGTGAGGATTCCGG
2054	tarc2458	Other	5D	211.0	CGCCTCGTCTGCCTCAGC	AGATATAGAAATCCCTTGGATTCCTC
2055	tarc0252	Other	5D	262.7	AGCAGCAGGCAGCTATACAGTCTA	CTGCAAACCCAAACTGAAACA
2056	tarc0261	CoreSet	5D	263.1	TATTGATGCCTGCAACTTAAATAAG	CACTAGGGCCACTTCTAGCGA
2057	tarc0229	Other	5D	266.1	TGCATAAAGTCCACTCTTCTAGATACG	GATAATAGTCAACATATTGCACACCATA
2058	snp5012	CoreSet	5D	267.8	TTTGGGCTGTGGAAGGACC	AAACTGCTACACACAAGAGTAAACAATT
2059	tarc2461	CoreSet	5D	267.8	TTTGGGCTGTGGAAGGACC	AAACAATTTTTTTTGCCCCTTATAG
2060	tarc0258	CoreSet	5D	269.7	TGGTAAACGAGTCGAGAGTTTGTG	GGAACAAGTGTTAGTAGCAGCACA
2061	tarc2442	Other	5D	269.7	CACCTAGAAAATAGAACATGTAAGCCT	CAACATATAATTCCTCAGGTTCTTGT
2062	tarc2453	Other	5D	277.8	TACTTTTTTTGACATGACAAGTATTTTA	ATCCTTCAAATGGATTATGATGACTAC
2063	tarc2447	CoreSet	5D	279.3	CCAGAGAGGCAAACATATTACGAA	AAAAAATGTGCATGTATCCTTGGA
2064	tarc0253	Other	5D	281.0	AGCTATATTTGTTCCGAGTCAGTTTAA	CGGTCGTAACAGGAACCGAT
2065	tarc2449	CoreSet	5D	298.8	GCCTCTTGGAGTACCAAGTTAAGA	AAAGGGAAAGTTGAAGAAATTGATC
2066	tarc1320	CoreSet	5D	309.0	CTCAAAGACGGGCAGGTAACT	CACTAATATAATCTGAAAAACACACACA
2067	tarc1435	Other	5D	321.7	ACAACAGCACCTTGACCCCT	ACTGACTCCTGCACCACCG
2068	tarc1263	Other	5D	322.4	TTCTCTTCTTCTCTTTAGAGTGCAGAC	GTGTACAGCCGATTCAAGGACA
2069	tarc0233	CoreSet	5D	326.7	GCTTTAGCCGTTTTCGCAAG	ACCTGTCTAAGCATCTTCCACATT
2070	tarc2446	Other	5D	331.4	CTTCAGCATCATATGCCCTTG	CTTAATGAATTTATTTGGCAAGTCAAT
2071	tarc1376	Other	5D	335.2	GCAGAGAAGGATGGCAATGGT	GGTTTTTCAGTTGATCGAAGTAGC
2072	tarc1398	Other	5D	339.1	AGAGGAATATTTTGTGTTGGGATGT	GGAATCAAGTGTAAGGAGTTAAGGAAG
2073	tarc2455	CoreSet	5D	339.1	CTATTTGACTAAGCACTATTAATTTGAA	ATTTTGTACAACTAGTTGCCGATGT
2074	tarc0232	Other	5D	346.0	TGTCCTTGGCCTTGTTACTTGTA	ACCGCTATTCAAATACACACCAA
2075	tarc1338	Other	5D	346.6	CAGAGTGGCAATTCCCCG	CTGGTTTTTGTGCCTTACATTACC
2076	tarc2443	Other	5D	354.7	CAGTTAATCAACAGGGAGTCAAAGA	GTTTCTACACTACAAAATTCCCTAATAG
2077	tarc1343	Other	5D	357.5	GTGTATAAGAGTATCTATACAACCAGGG	GATGGGGATATTTGTGGCG
2078	tarc1266	CoreSet	5D	365.3	AAGCACAAATCTATTGACCCTGTAC	GGAAAGCGACCTCCACTGAAG
2079	tarc2454	Other	5D	367.3	AGGTAAATGATTCATACTATGGAAAGG	TGTCCATTCCACCATCCTTTTA
2080	tarc0277	Other	5D	367.4	CTATCGCTCATGGCGTACAACT	TGTATGTCAATTTATTCCCTGTTCAA
2081	tarc1505	CoreSet	5D	376.4	CTGCCTGCTGCTGGTGGT	CTCATTTGCCCCCTCGCT
2082	tarc2452	Other	5D	384.0	TATTACACTCCTATACAAACATTGCCA	GCGAAATTACAAAAGTTGTGTCCT
2083	tarc1455	Other	5D	386.7	AGCTAATATTTTTTTAATGCAGAAATCT	TTTGTTGTGAAATTCAGACAGTTGA
2084	tarc0267	Other	5D	387.6	GCTGGTTGCAGTATCTTCAGGAT	GCTGGTTGCAGTATCTTCAGGAT
2085	tarc0269	CoreSet	5D	393.5	GACTTTCAGAGACAGACCACCCTATA	TTCATATTGAAATTTGGATATTTCCC
2086	tarc1432	Other	5D	397.3	CGGCTCCGCAGGGTCTATT	GCATAGTTACACGTTGACAGGC
2087	tarc0243	Other	5D	398.8	CCATTGCATGAAGAGATGAAGAATA	GTGGCATGGTTCCTAACCTGAT
2088	tarc2445	CoreSet	5D	398.8	AATTAACATCACTATCACAAGTGCCA	GGGAGGATACCCTGATCGTACC
2089	tarc2440	Other	5D	399.1	GCGGATGTGCAGAAGTTATCAG	TCAACAGCTAGGTATAGTGTACAAGTAC
2090	tarc1522	Other	5D	405.2	CTCCTCACAGCCACCCTCTTAT	GGGAGTCGGGCTGAGTCG
2091	tarc0278	Other	5D	408.0	AATAGAAATGCTGACTTGATTGCC	CTGAGAGGCTGAAGGCTTTACTAG
2092	tarc2463	Other	5D	410.1	AAACACAAAATTGCATCCAAAAGTA	CGGGTTTTTTTGTTTTATTTGTTGT
2093	tarc1487	Other	5D	412.4	CAGCCAGAGCAGCAAATACC	TGCGTTCCTATAACTAACACATTCTAAG
2094	tarc0230	Other	5D	420.3	CATATTTTCCATTAAGGACAACACTACA	CACTTATTTCGGTTGATAATGCAG
2095	tarc2441	CoreSet	5D	420.3	TTATACTGAACAGAATATATGAAAAACC	AAGAGAAGGCTTGAGATATTTACCG
2096	Inf107	Other	5D	420.9	CACACCATGTTTAAAGCACCG	GCTTCCCCTTCCTGTACTGATC
2097	tarc2448	Other	5D	422.4	CTTTGGCGGTGGCTTCAC	AGCCCCAACAAACCTAGTGATT
2098	tarc0276	CoreSet	5D	423.5	CCAAAATAGCAATGTAACATTACTGG	TCAGTTGAAAGTGAAGATGATATGTCTAA
2099	tarc2451	Other	5D	429.9	GTAGAGCCCGATGGTAAGATTG	ATTACCATCCGTCGGCGA
2100	tarc1444	Other	5D	431.8	CGAAAAACCTTGTAGAGCAGCC	TAGATGAACAGCTTTATCTGGAACTCT

Supplemental Table 4. List of amplicon sequencing primer sets for wheat.

No.	Name	Type	Chr	Pos[Mb]	F_primer	R_primer
2101	Inf48192	Other	5D	435.5	CTGCTCGAGCAGCTGCAG	CCTCCTCCCTGTGCTTCCTC
2102	Inf16696	Other	5D	436.2	CCTCATTCATGAGTTACACAGAATAAGA	CAAATTTGGGAGCACTCACGA
2103	snp1681	CoreSet	5D	437.8	CTGCTGATATACCTGAACCTACTACAT	ACTTGGTTGATGGATTCAACTTTTC
2104	tarc0245	Other	5D	438.8	CTGGTTTCTGAAACTTTCTGCG	AGGACCCTACAGCCTACATGG
2105	tarc1380	Other	5D	441.9	GCTTTGGACAACATGTGTGGAATA	CAATGTGAACTACAAGGGGGC
2106	tarc0237	CoreSet	5D	442.1	AACATTTGATGAAATAAAATGCTGA	CAAGAGAGGTGCTTACAACAACAC
2107	tarc1276	Other	5D	442.5	GATGAACCTACATGCAATAAAAAGGT	TCGTTAGTCCCTCCCTCGC
2108	tarc1347	Other	5D	446.4	ATGCAACATTTTCTAATTATGTTTTTAT	GAAGCTGAGCGACCACATTG
2109	tarc2456	Other	5D	447.2	GCACAGCATGTTGTTAACCTATGC	CACTTCTACTTTGGCCACCTTTG
2110	tarc1275	CoreSet	5D	450.2	CTCCCTCGCGGGTCTCAT	CACAATCACCAAGGTCCAGC
2111	tarc2462	Other	5D	450.7	AATTACAAACTAAATAAGTAGTGCCTTT	GATGATCTTGTTTTGATAAAAAATTAGC
2112	tarc0263	CoreSet	5D	460.7	TGGTTGACTCGGGCATCGT	GTCGGTCCAGCGGTGAGC
2113	tarc0234	Other	5D	465.4	GGGATGCTAACCATGCTATACTAAC	CTACTGCAACAAGGACCTGTCTG
2114	tarc2465	Other	5D	466.2	GCTCATATGCACCTGGACACG	CACTTGCCGAGCATCATTAGATATA
2115	tarc0256	Other	5D	467.6	GTGTCGGAACTGCTGGTAGTTAA	TCACATCAACAGCTCTGTCTTCC
2116	tarc2459	CoreSet	5D	470.0	TCTGAAAATATCTTGACTGGTCTGC	CCTCCTGACTAGAATCAGTAGAATTTTG
2117	tarc0236	Other	5D	475.7	AGATGGCAGAGAAAGAAGGGC	CATCCTCGGGGATCGACG
2118	tarc2450	Other	5D	477.3	CCATCGCAATTCCAAAAATC	GGAGACGGCGAGGATGGG
2119	tarc0235	Other	5D	479.8	TTCTGTCTTTGACCATAGATACTGATAAG	GGTTGCTGATCAAATTCACAATAG
2120	tarc1501	Other	5D	480.4	TGAGGGTCAGCAACTGCGT	GTTCCCCGACCACTCCGT
2121	snp678	CoreSet	5D	480.8	GAGGCGGGGTCGTACAGC	TCACGGAGCATGAGGCAAG
2122	snp2500	Other	5D	482.0	CTGATGAGTCAAATCTAACAAAATGAA	CAGAAATAGATCTTCCATACTTCTTTAGC
2123	tarc0257	Other	5D	482.1	GCTCTGAAAATCAACGAGTCGC	CCTAGAGTTATGCTATCAACAAGGTC
2124	tarc1385	Other	5D	483.2	TCGGCAAGCACTGGACGC	TGCAATCACTCCTCTTCCAACTT
2125	tarc0247	Other	5D	487.0	TTCGCTACTTCATACTTGAGCTTACT	CAGTAAGCACAGCTTCATTTTTCAT
2126	tarc0275	CoreSet	5D	488.4	AAGTCCACCAACAGGCAATCC	CACTGCTTGACCTCAGGAATTG
2127	tarc0241	Other	5D	496.0	GGCACAACACAACTTATCAAGGA	GAACCCTTCAGCGTTAATAGAAAT
2128	tarc1348	CoreSet	5D	498.5	TGGTTGTACCATTGTTTAACTTTTTT	AGTTCTTTCAAGAACCAACTTTCAA
2129	tarc0265	Other	5D	510.6	TATTATCAGCAATTGTGTTAGCAATG	AGAGTTGCTCAATCATAGGACCTT
2130	tarc1424	CoreSet	5D	515.3	GGTCGTCGATCCTGGGTCT	CGGCTATCTTCAATCTTGATTTCTG
2131	tarc2444	Other	5D	518.8	CGATCAACACGCTGATCACAG	TGGTGGCCTTCATCGGCC
2132	tarc1382	Other	5D	520.7	ACCAACATTGGGACTTCTGTTTTA	CTTTAGAAGAGCTAGAGAAGGCGA
2133	tarc1361	Other	5D	521.3	GTTGGCGGTCGCACTCAG	CAAAGATCAATATAAAGTCATAACAAGC
2134	tarc1377	CoreSet	5D	521.4	TTGATGCCGCCCTGCTTT	GAGTAGTCTATCTTTCTACTATGCCAAG
2135	tarc0251	CoreSet	5D	528.1	TCAACTAAAGGCTTGAATGGAATG	CGCCATCCTCCTACAACATCAA
2136	tarc1548	Other	5D	528.8	GCCCTCAGCAATAGCCCCT	TACTGGCAAACAAGCAGGGTT
2137	tarc1270	Other	5D	529.2	TGAAAATTTTGTTGCTAATTGCCT	GCACCACTAGCATTTAGCATCTAAGT
2138	tarc1379	CoreSet	5D	530.4	GCAGACCCCTTACATCCACG	GAGAGCAATATACCCTGTGTACTAACC
2139	Inf18733	Other	5D	531.5	CATGGTAGAGGCAATTCGGGT	CCATGAGCAGGTAAAAAGTGCC
2140	tarc1538	Other	5D	531.9	TGAGCATCTCAACGCACAGC	TACTTCGGCCATATAATCCCG
2141	tarc1354	Other	5D	533.0	GGTGCCCGTGCATCACCT	TGAGCTCCTTGTACTGCTGCA
2142	Inf15521	Other	5D	536.5	ACGAAAAGGTATTATCATATATTGTTGT	GTCTGCCATTCACAAAATTGGA
2143	tarc1371	Other	5D	536.9	GGTCATGTGCTCGGCTGAG	GGCCTCATCCAGCAGTCCAT
2144	tarc1323	CoreSet	5D	537.8	GGAACTCCTTTATTGTAGGGAACTTAT	AAAAAAAGAGATTAATAGTAGGTCTCGA
2145	tarc1547	Other	5D	538.7	CTAAAACATCAAGCCATATGTTGACT	GCAGGTTCTTGTTGCGGACT
2146	tarc1466	Other	5D	538.8	CTCCTCAAAGAAGAAGCTGGATG	CCGACTTGGGGTAGGCGA
2147	Inf18910	Other	5D	539.5	ACCACGCAATGAACTAGCACC	GGTGACTAGTTGTCAAAGTACCACTTAC
2148	tarc2464	Other	5D	539.6	TTCACATTGATATTTTTATATTTACACA	AAAAATATACAGCTGCTGGGCAC
2149	snp2821	CoreSet	5D	540.3	CACAAGCAGAGGGCATGCA	GGAAGAAAAATGAGCGCCA
2150	tarc1287	Other	5D	540.3	TTTTCTGATGTTCAAAAGCCTAAAG	AAATTCGAAGGGAAATGGTCTG
2151	tarc0271	Other	5D	542.0	AAGGCAGGTTGACACAATTCAC	TAAAGTACCACCGGGGACGA
2152	snp6409	Other	5D	542.7	AATAGTATGATGAAAGGTAGCAAGACC	GACTCAATTACACTAACTCATCGCAA
2153	snp7071	Other	5D	543.1	TTCAAAGGTGCTCGAAAGGAC	AGGCTTCTGCTGCTCCAGG
2154	tarc1489	Other	5D	543.3	CATGGCAGGTAGGTAGAGTCCA	CCGGCAGTCTAAAACGTCGA
2155	tarc0250	Other	5D	544.6	GCTACCAAAACTCAAGAAGTGTAACAG	GAAGATTCTGACAGAGAGGGCAA
2156	snp6189	Other	5D	544.7	CTCTCGCCTTCCAAAATAGTCAT	TCTTGGTTGTCGATGCCAAA
2157	tarc0274	Other	5D	544.7	GCTGGTACACTTTGCGAGATCA	CCATAGCCATCGTCATAGCTTCT
2158	tarc0239	Other	5D	545.2	ACAGGTACGGGCGATGCTAT	TGCAATTCCCTGTTTTTGTAGC
2159	Inf12263	Other	5D	546.7	TCTTTCCTGCGGCGAGTG	GACCGTGGATGTCGGTTCTG
2160	tarc1391	Other	5D	546.8	CAGTTAAGGTAGAATGGCGTGG	ACCCCTCTGCAACACATGC
2161	tarc1332	CoreSet	5D	546.9	GCTTGAATTGTTCTGAAGAAATGTT	AGTAATTCTCAACCCTGCTGGC
2162	Inf24648	Other	5D	546.9	CAGGACCGAGAAAATTATGACG	AGCTGTGTAGCTAATTATTCAAGCG
2163	snp699	CoreSet	5D	547.3	AACACAAAGTGAGCCATACACATG	CGACCACACTATACATACTGAAGTCC
2164	snp6872	Other	5D	548.0	TGTCACAGAATCCATCTGCCA	TCACACTCCAGTATACAGAGAAGCG
2165	tarc0260	CoreSet	5D	548.3	CCAGCAATATCCACCATGAACA	CCCTTCTAAATGTCACTCGACTGTT
2166	tarc0254	CoreSet	5D	549.6	CATTGCACCACAACTTCTCATACA	GTTTTAATCTTAAGCGTTATAAGGGTC
2167	Inf41284	Other	5D	549.8	AGGCCTTCTTGATGGATGCA	ACCGTAGGACACCCGCAA
2168	tarc0242	Other	5D	549.9	CATTACCTGCACATCAAATTATCAT	AGTGTATGCTTTCGTTTCAAGTTAAT
2169	tarc0246	Other	5D	549.9	TGGATCACAAATGATTGAGCCA	GCGATAAAGCAGAAGTCTAATGAAAA
2170	tarc0244	Other	5D	550.4	CGAAGCCTCTTGATGACAGCA	TCCATCCGGCAACAGACAA

Supplemental Table 4. List of amplicon sequencing primer sets for wheat.

No.	Name	Type	Chr	Pos[Mb]	F_primer	R_primer
2171	tarc0272	Other	5D	551.8	ATAATAAAATGGAAGTCAAAAGCGT	CATGTCTTCGCCCTAATATGCT
2172	tarc0238	Other	5D	552.0	CCCTTCACATGATTGTACTGACG	AAGAGTTTTTCAAGGGAATTTAGTG
2173	tarc0262	Other	5D	552.0	TGTTATGAATCTTTTGAGAAATATCAACA	TCTTGTCAACTGTCCAGAAATGTCT
2174	Inf10288	Other	5D	552.7	ATCTCCGATCGACGGCAA	ATCAGCTTCATAGTTTGCGCAC
2175	tarc0270	Other	5D	553.7	TTCTGGAGCAGGTATGTTGGC	AGAAAAGATATTTGAACTTATCATGGTTTA
2176	tarc0231	Other	5D	554.4	TCCAAGTTATAGCTAATGCCTGG	CATTGGAAGAGCTATTAAGACTTCAAG
2177	tarc0259	CoreSet	5D	554.5	CTTTTGTAAATGCACCCTTCGA	GTTTTTGCTCCATTTCAGTACTCAT
2178	tarc0240	Other	5D	556.6	CAACATTAAGCTGTGTCCTGATGTAT	CATATTTATTTAGCAGAAAATATGTAGCAC
2179	tarc0264	CoreSet	5D	558.3	CAAGATGTTTCATGGGGACG	GATGGTTCTGTATCCTTTTTACCGT
2180	tarc1485	Other	5D	558.3	TATTTTAAATGTCATCAAATAATTAGCT	TATGAATTTTTTATTATAACTCAATCCT
2181	tarc0273	Other	5D	558.4	GGTGTGTAGGGGCTTTTGGAG	ACATGCTTTCTTTAAACAGCACG
2182	tarc2460	Other	5D	558.4	ACATGCTTTCTTTAAACAGCACG	GGTGTGTAGGGGCTTTTGGAG
2183	tarc0255	Other	5D	559.4	ATTCCCTTAGCCAGTCAACAATG	GAAAGATAAGGAGGAAATGAGGAAT
2184	tarc0279	Other	5D	559.4	TGCAATACAATACTACTGGAAGAAAGA	CTTGGTAGGTGCGTTCGGA
2185	tarc1474	Other	5D	559.9	AACCTAGCCATTACTGGGTGACTT	CAAGAAGGACCTGCAAGTTAAGA
2186	tarc1325	Other	5D	560.2	GCCGAAATACAAAATACATGCTGA	TTCTAAATATAAGACCTTTTAGTGAACC
2187	snp1427	Other	5D	561.7	GACAGCAGCAGGGGTTCGT	CTGAACCTAAAAGCAATGCTTCA
2188	tarc1459	Other	5D	561.7	CAACAATGCTCACTTTTATCTGCT	TACTGTCACATAGTACGTATATGCCAAT
2189	tarc0266	Other	5D	561.8	TTACCTCCAACTCACAGGATAATGT	GCATCCTTCCTTCTTTTATCCAAG
2190	tarc0248	Other	5D	562.0	GGCACAAGTCATGGAGCATG	TACAACATATACAATGGGATGGAATC
2191	tarc0249	CoreSet	5D	562.0	CCGTCGTGTCATGTGTCGA	ACTTTGAGCAGTCCACAGTTTGA
2192	tarc1337	Other	5D	562.1	ATCAGGAGAAGCTCCAGGACAG	CCTAATTAAGTGATAATCATCCAAGTAA
2193	snp4561	Other	5D	562.7	ATTTCTGAAGCAAGCAAACAAATC	CGAATCCAATCGAATCCTAAGCT
2194	snp4113	CoreSet	5D	562.7	GGGTTGCCATTCAAGAATTTTG	AATATTATGCTCAAAGGAACTAAAGTAGAA
2195	tarc0268	Other	5D	562.7	GAGCGACACGTTTGTGAAGGA	AATATATCAATGTCAATGGTAAAACAAGA
2196	tarc1417	Other	5D	562.8	AGACCAAAATGACTGATGGAGAAG	GGTATTGCCAAGGTGGATATGC
2197	snp6937	CoreSet	6A	0.6	TTACATGTTGGTAGTAAGTAGTGGTAGC	AAAAATCAGAATGCAAATAAGAATCA
2198	tarc0732	Other	6A	0.6	CATCATCAAGGAAACAGCAAAAT	ACATGGCAAAAAGTAAGAGGCAT
2199	tarc1892	Other	6A	0.6	CCGACATAAGGAATGTTTCTGTAGA	GACTTGTTGTTTGCCTATGTCAGA
2200	tarc1903	CoreSet	6A	0.8	GCTTCGGCATCGTGCTGT	GTCATGGTCGGCCTGTCAA
2201	tarc1910	Other	6A	0.8	CGGTCGACCTCGGTTTGG	CCGTGGTGATTTCTCCCG
2202	tarc1898	Other	6A	0.9	TGGGCAGATTTCAGTCAGGAA	AATTGTCGCTTTCCAGTATCAAGT
2203	tarc0693	Other	6A	1.5	TCTTAATCTGGCACTGTATATGTTTCA	ACAAGGCATAACCAAGACGTATGT
2204	tarc0717	Other	6A	1.5	GGCTGCTTCCAAACCTCCA	CTTCATGTCCAAATCTTTCTCCA
2205	tarc1893	Other	6A	1.5	CATCAAAGATGGTAAAAACTAGTCATCA	TGCTTACTTTGGCCCTTTTTTC
2206	tarc1899	Other	6A	1.5	CGGTTTCCATCGTTTGGTAGG	CCTTTGGATCGCAAGGGG
2207	tarc1904	Other	6A	2.4	TGGACATCTTTCGAAGTTCACTG	GCTAAGACCAATGATTTGGCTG
2208	tarc0720	CoreSet	6A	2.8	CCTGGGTCACAAACAGGAAAG	CCATCTATTTTTTGGCTCCGA
2209	tarc0730	Other	6A	3.9	AGGTGTTGTCCTTCTTGAGCATC	GGCTTGGCATGGCTCTTCTT
2210	tarc0728	CoreSet	6A	5.3	TGTTATTGTTTGGTTTATTCTGTATCAT	CAGGGGAGAGCAGGAGCG
2211	tarc1900	Other	6A	5.3	CCAAACCAACAAAGACGGACA	CCGAATCGAACCCAACGG
2212	tarc1894	Other	6A	6.6	GATGGCGTTAGATGACAGGTGAT	CTATTACAACATAAGCATGATAACTTGA
2213	tarc1897	Other	6A	6.7	CATTGGCCGCAAATCCAC	GCCTAGAGGGAAAAGAATTCAGAT
2214	tarc1901	Other	6A	6.7	ATAATGTCGGGGGCGTGA	GATAGTAGCATCGAGGAAGAAAATG
2215	tarc0682	CoreSet	6A	7.2	ACGCCACAAAGCAAATGGTT	ATGTCCAATGGTCGGATTTTG
2216	tarc0694	Other	6A	9.3	AACCATTGGCATCACCATAACTC	AAGGGATTCACTCCTTCCACAG
2217	tarc0681	Other	6A	10.5	GCACTTCATACATCTGAAATTTTCTCTA	GTGCGTCTTCCAGTTGATCG
2218	tarc0686	CoreSet	6A	11.2	CTGAGAATACTGTAAGCCTCCCAT	AATTTATTAAACTTCCAAGTCAGGGA
2219	tarc0707	Other	6A	12.0	GATAAAACGTAAGAGAATCTGTATCTTT	GAAATAATAATCAATACTTACATGGCGA
2220	tarc0731	CoreSet	6A	12.3	ACATTTGGCAGATAACTAGTGCGT	CATGTTGTTTGGTTCTTGATACTTCA
2221	tarc1907	Other	6A	16.2	TCTTTGTTTCCTTTTTTGTCTTTTG	AAACATGATCTCAAAGTTTCAACGA
2222	tarc0718	Other	6A	17.6	CCCACAGAACAACACAAAAAAGA	CCTGCCAGACGCTGAGGC
2223	snp1335	Other	6A	24.8	CTATCACTGTCAATCTCCTGCATTC	CACTTAGGCACCTTTCACTTCG
2224	tarc1908	Other	6A	24.8	GCCAAGGACTACCTGTCGTCA	GGGTGATGGATTCGATTGTTG
2225	tarc1895	Other	6A	25.6	TTAAAAGCAACAGAGATCTTAGTTCTTG	CAGCTTAATTTTTGATTGGGGC
2226	tarc1906	Other	6A	26.8	TCGCCGAGACCTGCAACC	CGGTGAGCAGCTTTGACG
2227	snp8510	Other	6A	30.5	TTGTACATATAGACGGATGGTCCTG	CAAGGGAAACAACATTCTTATTGA
2228	snp612	Other	6A	31.4	ACTGATTGTTAGTTTCTTACTGCAATAC	TGCATGAGAAAGGGTTTCGAG
2229	snp3322	CoreSet	6A	32.7	CTCGATTCTTTTACTGTCATTCATGA	GTTTGCTCACCGAGTCTTTTAACTA
2230	tarc0722	CoreSet	6A	39.5	TATCAGCTACTCGGTGACCCAA	TTTAGAGACTAATAGGACAAGGGGC
2231	tarc1909	Other	6A	52.3	TTGTGCAAATTCCTTTTGTTCTG	CGAGCTTAAGATAGCCAAAGCATAC
2232	tarc0708	Other	6A	54.2	TTCTACAAAACACCATTGACATCTTATT	GTTACTTTTGCGAGACAGTCAGC
2233	tarc1902	CoreSet	6A	55.5	GGTTCTCACTTTACGTTAACTCCAA	CCCCCACCACTCCATCCT
2234	tarc0726	CoreSet	6A	60.0	TCCTTTATACGGCGCTATACGTAG	TCAGGCTCAGCTTGTGATCAAA
2235	tarc0725	Other	6A	60.2	AATTGTGGTGAAAATAGGCTCATC	TTTATATTAATCATTAAGGTTTGTGAGT
2236	tarc0702	Other	6A	61.4	CAGAAATCTTGCAGTGTACAGACG	CTGAATGCCTTAGCTCAGCCA
2237	tarc0698	Other	6A	63.6	TTGAGAAAATATTATAAACATTTAGGAA	TTGTAGACAATCATGTCTGATAGAGATA
2238	snp2687	Other	6A	64.3	GATGTTGCGACAAAGAGATTGC	TGTTACAATGAGTTCGGCTTCC
2239	snp2685	Other	6A	64.3	ATGGCACCCCTGTTACATGAG	TCAGCTAGCTGACCTCAACAATT
2240	tarc0733	CoreSet	6A	65.7	CTACTGAGGTTGGGGACGGT	AGCGACAAGTTACTCAAGCCTG

Supplemental Table 4. List of amplicon sequencing primer sets for wheat.

No.	Name	Type	Chr	Pos[Mb]	F_primer	R_primer
2241	tarc1905	Other	6A	65.9	GAGCATCAGCACCGTTGAGTT	AGCCAATCTACCGTGATCGTG
2242	tarc0703	Other	6A	70.6	ATTTATATCAAACTTGATCCATGAAGAG	TGCTAACTGTAAAATCATGTAATTTGTA
2243	tarc0692	CoreSet	6A	70.9	GGCAGGTGTCAAGAGAATATGG	AGGGCTCAAAATCCTTTCTTATGT
2244	tarc0704	Other	6A	71.4	ACATCGCAATTCACAAATCCAG	ACCATACATCGAATTTACTTGATTGA
2245	tarc0714	CoreSet	6A	73.3	TTAATGAGGTACAAGGGATATTTCTTTT	CAACCATCAGCACTCTCCGC
2246	tarc0695	CoreSet	6A	109.5	CAGAATTTGCATTAGACATCCTCTT	AACAAGAACATATGAAATATGAACTAAT
2247	tarc0684	CoreSet	6A	315.6	AATTAAACAAAATGATACTTTGAAGGAA	CTAAATTGTCATTCTTGAGATCTGCA
2248	tarc0710	CoreSet	6A	386.9	TAAGAGCGTTTTGACACTACACTAGTAT	TGCTACACATCTTTGTAAGTGATTGC
2249	tarc0687	CoreSet	6A	455.7	ATTACAAAAATGTGCTGTAGTAGTTCG	CGAAGGCACTCCATGATACG
2250	tarc0713	CoreSet	6A	501.2	CCGTGATGGATATCATATCTAGTAGACT	CGACTCTCACAGGTGGGCA
2251	tarc0688	CoreSet	6A	502.7	AAGATGATGACGAGGATGATGATG	ACGAGTGCAAGAAAATCTACAAGAA
2252	tarc0712	Other	6A	502.7	GCAACTGACAAATGCCTTAACG	AATGTGGTTTCATAATCATGGGG
2253	tarc0701	CoreSet	6A	504.5	GCATCAACAACAAGACAATTAACATG	TATTTTCTTTACGCTCCTAGCCAC
2254	tarc0700	Other	6A	516.4	CAAGGAGTAGCCCAGGTAGAGTG	GGCTGGGGAGGTTCTACTATGTAT
2255	tarc0685	CoreSet	6A	532.2	GTCGTACATAGAGCCAGAATGCT	ACCAACTGCAGTATTTCCTTGATAAT
2256	tarc0716	Other	6A	532.6	TCTTTTTTTACGTCAACTTATGATCGT	GAGAAAGAGCATCAACAGCGG
2257	tarc0689	Other	6A	533.4	TCTCTTGGAATACCAAGTTCAATTTAA	CTAAGTTACAGTGACAGGAAAATTCC
2258	tarc0719	Other	6A	535.8	AGAATATTAGCGCATGATCATATACTAA	AACTTTTTCCAGCCTATTTCACG
2259	tarc0691	Other	6A	541.4	ACAAAACAGTAGCACTCCAATGTATT	GAGTTCGCTCTGGGTTTGCA
2260	snp741	Other	6A	542.8	CGGAATGCATATAAGTCCTGTCAAC	TATGCATGGATTGGCAAAACAC
2261	snp1474	CoreSet	6A	544.2	AAAGTTCAATATCAGCATTGCTGAT	GTCTTTGAATCCAATTTGCCAGT
2262	snp4929	Other	6A	544.5	CCGCTTGGGGTTGTACGA	CCCATTCGGCACATCAGC
2263	snp3269	Other	6A	545.8	TATTTTCCTGTTGGGTATGGAATT	GCTAGTTGAACCATCTTGGAACAG
2264	tarc1884	CoreSet	6A	545.8	CAGCAGCAAAGAGCGACG	CAAGTTGATTGTTCTTCCCACACT
2265	snp5074	Other	6A	548.4	TGCATTACATTGCATAATACTCGC	GATGTGCAGATACCAACAAACCA
2266	snp6508	Other	6A	548.4	GCAAGAGTCCTTTCACAGCGT	ATTTTCGCATAATGGACTTTAAGC
2267	tarc0690	Other	6A	548.4	CAGGTTCCACTAATACGTGAGACAT	GCAAGAGTCCTTTCACAGCGT
2268	snp224	CoreSet	6A	550.1	TACACTCCATTTGCAGATTGTTAAGT	TAAATACTCAAGATAACTGGCAATGG
2269	tarc0696	Other	6A	550.1	TTACAGAATCGTCCCAGGTATACG	CCGACTGAACCCTGCCTG
2270	tarc0727	Other	6A	556.5	TTATGAAAGATGGTGATGGCGA	CCCTTGTGCGGTTAAGTAACG
2271	tarc0709	Other	6A	559.0	TTCATCATATTCATCTTCATCATGTTAC	GACATCAAGACGTATAGTGCTCTGAC
2272	tarc1880	Other	6A	565.0	CACCATTTTTTTGTTTATATTCTGTTTG	AGCTACCTAAAATTAGGCACTACCAA
2273	tarc1882	Other	6A	565.3	AAAAAGCGGTTCTCTCCACAGTA	TAATGTTGAGAAATCTTTCAAAATCG
2274	tarc1888	Other	6A	567.9	TCAGCTTCTCCGGTCATTTTG	ACATGTGAGCACACACATTAGGG
2275	tarc1891	Other	6A	569.1	AGCTCCTATTTGAGCGTGAAAGA	ACCATCCTAAATGCCTGTTACTTG
2276	snp6550	CoreSet	6A	570.4	TGAAAATGTTATGATTTTACAGGTCG	TGCTGCACCAAACAAGCATAA
2277	tarc1869	Other	6A	570.9	ATGTATTTACTTAATGTTCATGCGGA	CACTGAAACCTATTGAGGAGAATAATAG
2278	snp3023	Other	6A	573.5	ACCTGGCACCTTGTTATGTTGA	AGTTCTACATGCATGCAAACACC
2279	snp6194	Other	6A	573.5	GGTGCCTATGTTTCTGAGCTGTA	ATACCCTCAGCACAATGTAGTTTGTAC
2280	tarc0729	Other	6A	573.5	TGCAAACAACCTGGCACCT	TAACCTATATCTATCTACCGCTACAGGA
2281	tarc1890	CoreSet	6A	573.5	CACAAACCAAACCGATTAGTCAA	GCAGACTTTGTGAAGGACGTG
2282	tarc1871	CoreSet	6A	574.5	TGATCCGTTACGAGAAGCAAGA	TCGTGAGCTTAGACAAAAGCAAAT
2283	snp259	CoreSet	6A	580.2	TTGTTCAGGTCTTCGATCTTTCTAC	GGACACCTAAATGGTGCAGGA
2284	tarc0699	Other	6A	581.8	GGAAACGGTGAGACTCTGAATAGTG	ACTCTTGGCATGGTTGGGG
2285	snp3491	Other	6A	581.9	TCATATTAAACTGATGAAGACTTATCCC	CCATGAACAACATGGTACATTGCT
2286	tarc1886	Other	6A	597.4	TATGGAAGAGATAAAAAGGGGTAGAC	AGAAATGTTGGTTGGGTATGAGC
2287	tarc1870	Other	6A	597.8	TTAAATACAAGAGAAAAGGCTGAATCT	TGAAATTTGACATACTGGATAAGTTGA
2288	tarc1875	CoreSet	6A	599.9	TGGTTACATCACGGGTGGTAAGTA	TCCACCAGACGCCCTTGA
2289	tarc1874	CoreSet	6A	602.7	ATATGGACACAGAGCCAATCAATC	TAATTTCTGCATATTTCTATCTCTACCC
2290	tarc0697	Other	6A	603.2	GGTATACTTCTCTTCTCTCTTTAGTCTT	TAGAAACATAAGAGTTCCAGTAGAAAAC
2291	tarc0721	CoreSet	6A	603.4	GGTCAATTTTTGTTTTCTAAGATAGCTC	AACATCCATATGTACTCGTAGAAACAAT
2292	tarc0711	Other	6A	605.4	ATTCTCTTGATTTTTACTGTTTACTAAA	CTAATAGTTTTGCACAGTAGAAACGG
2293	tarc0724	Other	6A	606.8	CTTGTCCAGCTTCTGGCAAA	GTTTGATTCAGTCCTGGTTTGATAA
2294	tarc1887	CoreSet	6A	606.8	CAGTGGCGGGACCTTTGAG	CCACAAGATCAGCAAGAAGAAGAA
2295	snp6316	Other	6A	608.6	CTGCATATTGGAATCCAGGTTG	ACAGCAGGTAAGCATGGTGATG
2296	tarc1872	CoreSet	6A	609.2	GCACACCGGTCACTTTTTACTT	TCGCCGTTTTGCTATCCTG
2297	tarc0683	Other	6A	609.3	GAATTGATGGCTTCGCCAC	GACGATCAACATTTCAAATGATGAC
2298	tarc1878	Other	6A	609.3	CGTTCGTGGAGAATGTTAAGAATC	ATTTATTTAGCCAAACATTTGTTTAGAT
2299	snp3066	CoreSet	6A	609.4	CTGTAGCTTCTGGTTGCTGATTC	GCACTGTCTTTAGTGAGCTATATGGAT
2300	tarc1868	Other	6A	609.5	CAGGCAGCATCCACAGGG	TTATTCACATACAACTAGCAGCAACAT
2301	tarc0715	CoreSet	6A	610.9	CATTTAACTGTCTGTCAAAAAGGC	TAAATTTCGGCATTATTATTCCGT
2302	tarc1883	Other	6A	610.9	GCGCTGCTAGGCCACTCG	AAGAATAAAAAGGTCCATCTTTTCTATG
2303	tarc1889	Other	6A	610.9	CTCGGGGTTCATGTTGTGC	AGACCCCCACGTTGGCAC
2304	snp2054	Other	6A	611.3	AAAACAATGAATCAGAAAGTAAGCATA	TGCTTCTCCACCATCATCGG
2305	tarc1873	Other	6A	611.3	CGAGGGTTGTTAGTATTGAATAGCG	GTCAGTGTTCATAAAACAGGGGA
2306	tarc1881	Other	6A	611.8	GCCTCTTCTTTTACCTACTCAAGTTC	CTTGGGTTTTGACCTTATATCCTTTA
2307	snp5747	CoreSet	6A	611.9	AGCCTGTGTCAGATGTCAGTGC	CTGTGTCGGAGAACTCCTCG
2308	tarc1877	Other	6A	611.9	GCAAGTCGTTCTACGAGGTGTATG	GGAAGATCATGCCATGCCTG
2309	tarc1885	Other	6A	613.3	AATTGGTTGAAGGACAACAAAATC	CAACCTTTTGGCGGTAATATCA
2310	tarc1879	CoreSet	6A	613.5	GGAGGAGTGCTGATGGTAAGAA	CCTATGTCCCGTCCTTTTTCAA

Supplemental Table 4. List of amplicon sequencing primer sets for wheat.

No.	Name	Type	Chr	Pos[Mb]	F_primer	R_primer
2311	tarc1876	Other	6A	614.6	GCATCTTCGGTGGATCAAGTGT	GTGTGGGTCTATGTATGTTTATGTTTAT
2312	tarc0723	Other	6A	614.8	GGATATGGGTGTCGTGCGTG	CGCAGAAAGAATGATGCCC
2313	snp2795	CoreSet	6A	615.5	TTGAAGTATATCTTCTCCATGGTGTTA	CCTAAAAGTTGGTGCGGCG
2314	tarc2308	CoreSet	6B	0.2	CCCTTCAGACCATGGTTCCA	CCCACATGACAAATAGTCGAGAA
2315	tarc1191	Other	6B	3.4	GAAACAAGAAGCTAGATGCAAGTTG	GGAATATTTGCGGATTTCTGTTAG
2316	tarc1189	CoreSet	6B	4.5	GAGAAAAGTACAAAATTTGGGTGC	TTATTTTATGATCAAATTTGAGATATGC
2317	tarc1171	CoreSet	6B	4.9	CGCAGATTCTTTTAGATGTTGCG	CGTTGCCTGGACAGCAAAG
2318	tarc1186	CoreSet	6B	11.3	TGGTGAAGAAGAATAATAGACCCTTTAG	GAAACCAAGGCCCATACCAA
2319	tarc2285	Other	6B	12.7	CCATTTATGATGCACCTAACACCT	AAGGTCTCCCAGTGCAAGCA
2320	tarc2292	Other	6B	12.7	TCTCTTGCACCACAAAGTCGTC	TCACTTAGTGGCTGCTAATTTTACAA
2321	tarc2312	Other	6B	15.8	CGCGGTTAACCAAGGTTGTCT	TTCGCATCAAGATCACGGG
2322	snp4633	CoreSet	6B	17.4	GCGATGTTGTAAATTAAAAATGGAAT	CACCACAGTATCCGACAGTCTGTT
2323	tarc1194	Other	6B	19.1	CCTGCTGGCCTCTCATGTTCT	ACTTCAGAGATGGCTTGAGATCG
2324	tarc2279	CoreSet	6B	27.3	CTTCGAGGAGGCCAGGGT	TCCATGATAACTAGCGTATTTACTGTTC
2325	tarc1195	Other	6B	36.2	ATCATTTATTGGTCAAGTATGACGC	TGTTCTAGTGTAAAACTTGGCTAAACC
2326	tarc1201	CoreSet	6B	42.0	CGCACTCGTGGAACAAACTT	CCATCGTGTTGTTGGAGAATCC
2327	tarc2297	Other	6B	42.0	TAGAGCTGTAGGTTCAATGCCG	GGACGTTCTTCGTCAGGTTCG
2328	tarc1149	CoreSet	6B	48.4	CAAGTGTCAAGGCATCAGTTAGTGT	TCTCATAGTTTGACCAGCTTGTCTTAT
2329	tarc2295	Other	6B	48.4	CTGTCAGCGCTATCTATCGTCG	CTGCTGTAATTTAACTCCGTCCC
2330	tarc1137	Other	6B	65.0	AAAGCTCTGCTGTTGCCTTGA	ACAGCCCTCAACGAGTACTGAAG
2331	tarc1164	Other	6B	75.0	GAACAAAGTGATGAATATCCTTACAGTA	ATTTCCAGTCACTGCCAGTTCA
2332	tarc2289	Other	6B	92.5	GTTAGCCTCCTGATCTATTATTTGTGTA	TGAAAGGTATTCTTCAAACTTCTCCA
2333	tarc2281	CoreSet	6B	95.5	AGAGATCAGGAAATACTTTTACGGC	TTCCCTCTAGGTTAAATATGCAACA
2334	tarc2293	Other	6B	98.9	GAAAGCTTCTTTCACATTGATAAGC	ACTGGTTCTCACTTTATGTTAACTCTGA
2335	tarc2299	Other	6B	99.0	AATCGATCCACTGTCGCCC	CATAAACCTCAGGACCCTTGATG
2336	tarc2275	Other	6B	100.3	TGGAAAAAAAAAATCAGAAATCAGAG	GTTTCATGCTTACATGCTGCG
2337	tarc1142	Other	6B	116.6	TTTGAAGTAAATAGTCAAGCCATTATAA	TCACAGTTTGTTGAGGATTTTATTTG
2338	tarc2309	CoreSet	6B	119.8	CAATGGGAGCTACAAAGTCAAGA	CATATAGCAAACCAACCAAAATGA
2339	tarc2304	Other	6B	120.6	CAAACACCTGAAAAGGATGTCAA	AGCATGATGGTTAACCAAACGA
2340	tarc2291	Other	6B	122.8	TTACAGGTAGGACATCGTTATAAAATAA	GGGCACACGTAAGAGAATTAGTTCT
2341	tarc2306	CoreSet	6B	126.2	TTGCTCTTCTCCTCCCCGT	GCCAAGGAATTTGACAATGACA
2342	tarc2300	Other	6B	126.6	GCTCCTGCTGCCGGTGCT	CGAGGATCTTCTCACAGATCGAATA
2343	tarc2280	Other	6B	127.3	GCCACTCCTTTCGTGCCTAC	ATTACCCGCGTGACTTGCTT
2344	tarc2298	Other	6B	127.3	GTATAAGCATTTCGAGAGGTACACG	CACCATCGAGTTCGACATCAT
2345	snp8284	Other	6B	127.7	CCTTGGGTTCAATTTCAGATCG	TCTAAGCAGAAACACACCCCC
2346	snp6494	CoreSet	6B	129.3	ACTAGACTTCATCTGCAAGACAGTGATA	GAGCCCTATTTTCAACTTCCGA
2347	tarc2278	Other	6B	129.3	TAACGAAGGCAAAGCAAATGATA	TGCTGCTGCAGCCATGCC
2348	tarc2284	Other	6B	135.8	TGTGTCATCCTCATAGGTGTTTTTC	TTTGATAGCTATGCCCTCGCT
2349	snp7689	Other	6B	142.5	TGGAGATAAGCTGCTGCGG	AAGAAAAACTCAGTTCTAAAGAACAGC
2350	snp2937	CoreSet	6B	143.4	CAGCAAGTGCAGCAAGAGAGA	CATGAGACCTGATATTGATAACATCG
2351	tarc2305	Other	6B	143.4	CAGCAAGTGCAGCAAGAGAGA	TGATATTGATAACATCGACGAGTACG
2352	tarc1188	Other	6B	144.0	CAAACTTAATATTCACTCTTGCCTTGT	ACTCTTGCTCGTGATATCGAGG
2353	tarc2303	Other	6B	144.9	GAATTTCTTTGGACTGATCCATTCT	ATGACAACAGGATGTGTCGCTAT
2354	tarc2310	Other	6B	150.8	GAATGTTATTCTGTTTTCTTCAGGCT	CGTCAGATAAAAACACAGGCCT
2355	snp7896	CoreSet	6B	151.1	TAGGAAACCGTGTTGTCCTGTTC	GAGCCAACCCCACATTGAC
2356	tarc2302	Other	6B	154.0	AGTGTACACTGATCTTAACAAGTCGC	CCTGTGATATAGGAACATCGCTTC
2357	tarc1147	Other	6B	155.8	GACTTCAACAGAGTCTCCGACG	TATTGATTGAGTAACTAGCATATGTTGT
2358	tarc2294	CoreSet	6B	164.4	TCTTCATGGATTATTATGCTCTTTTGT	TTGATGAAAAATACAAGCATGTCAGT
2359	snp3634	Other	6B	173.6	AGCGGACCACAATTTTATGGA	AAAAAGAACATGGATTAGATCCTCA
2360	tarc2282	CoreSet	6B	180.0	GGAGGCCTGAATGTACGGTTAG	GAACATAAGATAAATTTGATGGCAGTAT
2361	tarc2301	Other	6B	187.8	AATATTTTAGGTCAATTGCTATTTCACT	GTGTATTTTTGGCTAAGCAAGGAA
2362	tarc1169	Other	6B	189.2	CAGTTCGCCCTATTTCGCC	TGGTTTTACCTGTAATTACCTGAGAGT
2363	tarc2274	Other	6B	191.8	TGGTCCGTTCCAAGTCCG	TGTGGCATCGAGAAATCCGT
2364	tarc1198	Other	6B	194.4	CGTGACGAATCAAGTGTGGCTA	TCCAAAGTATGATGTTCATTGGCT
2365	tarc2276	Other	6B	195.2	CGGCTGAGGTGAATGAGGC	AGCAGTTGTTGACCCCACG
2366	tarc2311	CoreSet	6B	195.2	AGATGGGGGGTGGCAGTT	CTGTGTTTGGGATTCCAGAGAAG
2367	tarc1159	CoreSet	6B	203.9	CGCTGCCACACCCACGAT	TTCCCTTCGTACATGACACATCTC
2368	tarc2277	Other	6B	204.6	AACAGGCTAAACGAGAACGCTC	TCAAAGTTCAAAGGAAACCCG
2369	tarc2307	CoreSet	6B	207.9	GCAAAATCCCTGGGAATTTTCT	TGGTAGCGACTGGAGTTGGTG
2370	tarc1184	Other	6B	226.4	ATGCTTGATCATAACGTCGGG	GACAGCCATCTAGCTCCTACCTT
2371	tarc2296	Other	6B	226.4	CCAAACCATGACCAAATTAGAACTA	CCTCTTAGCTAACTTGGATTGTGTTAGT
2372	tarc1166	Other	6B	226.9	GCAGCCTGTGGGAGGTGA	AACTACCTCAGCGCCTCACAG
2373	tarc1173	Other	6B	231.7	CTTGCAAGCAAACTCTCCAGG	CTGGCACAAGGTTTGAAGCA
2374	snp5748	Other	6B	236.6	GTACCTCCTGCATCAACTGACCT	ATTGTCAGATGAACGATGAAAATCT
2375	tarc1183	Other	6B	239.3	TTGACTGGAAGGAAGCCGC	CACAACTGAATATCTCTCCCAAACA
2376	tarc1152	CoreSet	6B	248.4	CCACAGATTATTGATGCTTCCG	GTTCATGAAGGAATAGATCTGACATTT
2377	tarc1185	Other	6B	258.0	ATTTTGAATGGTTCTTTGTCCAAA	ATCTACTCAAGTGTTGTGTCAAATCG
2378	tarc1170	Other	6B	259.3	AATCCAGTGGGCGGACAGTA	ATGAATCTAATGAAATTCATTTGATACT
2379	tarc2290	CoreSet	6B	259.3	CCCCAATTGGAATGATTGAACT	TTTCTCTAGCACGCTACAAGTTACG
2380	tarc1175	CoreSet	6B	259.9	TGAAGCCGTTGCTCAGACTG	CAATTCCGATCCACGTTTTACATA

Supplemental Table 4. List of amplicon sequencing primer sets for wheat.

No.	Name	Type	Chr	Pos[Mb]	F_primer	R_primer
2381	tarc1138	Other	6B	261.5	GAACAATTACATCTGAAAAGTTAGCG	CACACATACACCGTGAGGAACA
2382	tarc1162	Other	6B	262.5	CTTAGAATTTGTTTGTTATGTTGTCGT	CAGCAGCAAAAAAACAACACAATA
2383	tarc1150	Other	6B	274.2	AAGCAAATCTAGGTTCCCTATACTCC	GAAGCATGGACAGGACTTCTACAA
2384	tarc1197	Other	6B	278.7	GCCAAGGGTCGCCTCTGG	GTTAGTTTCTTCACGCAGGGG
2385	tarc1145	Other	6B	287.1	CAAGAGGTAAGCAGCAAAGTTATTG	CTCATTGAACTAAATCAAGTTTTTCAG
2386	tarc2283	CoreSet	6B	287.1	ACTACCACAACTCAGTAATGATTTATTA	CCATTTAGAAGCATGCATGGAT
2387	tarc1174	Other	6B	295.8	ATTAGATGTCAACAACTGTAACTGTACT	GAGGGAAAAGAAGTCAAAGATGTTA
2388	tarc2288	Other	6B	295.8	TTTGCATTGACGAACAGATAACAA	GTTGGCACTGCAACAAGAACA
2389	tarc2286	CoreSet	6B	296.4	GCACCCAACCTTCGAGCC	TGTTTGTCCATCCTTGTCTATTGTAA
2390	tarc2287	CoreSet	6B	306.8	CGACCCTGAATTATTTTATGGG	AGAGGACAAGGAACAACAAATTTTC
2391	tarc1167	Other	6B	315.3	CAGACAACATGGCATATATTGCAT	TCCAAGTCTGAAAATACATAACTGATCT
2392	tarc1139	Other	6B	315.7	CATACTGATCCTACAGCATTTGCA	TATTATGCAACTGGCTTGAGGG
2393	tarc1196	CoreSet	6B	337.2	GAGTCTATTCACAAGTTTCTGCACG	TCCATTTCATGATGCTCTTTCCT
2394	tarc1199	Other	6B	352.1	TTGCTTTGTGCCATCACCTG	GGTAACCTCCCGAGCATAAGA
2395	tarc2241	CoreSet	6B	366.7	TCTTTTCCACGAAAATACTCAACAC	GACTGCAAGATTTTTGACACATTCT
2396	tarc1157	Other	6B	377.9	CCAGAGTTCACTTTCAAGGACTCTT	TTCAACTAATAAAAAATGATTAACTGTC
2397	tarc1153	Other	6B	382.8	TATGGCTTGATAATGGGAGGC	TGGCCGTGTTTTCTCATAGTCTA
2398	tarc1158	Other	6B	384.8	GAAGAAGTTTCCTTAGCACACTAATACA	ACATACGATATGTAGTGACCAGAGAAAG
2399	tarc1141	Other	6B	387.1	CGCATGTCTTTTTGTGACTATTCC	AAAACTAACTCTGAATCCTCCACTATAT
2400	tarc1190	CoreSet	6B	402.2	GAGCAAATGGGGAGAATAGTAAAGA	GGTGCTGGTCGTGCTACTACC
2401	tarc1140	Other	6B	405.0	CAAGTGTACAACTCTGGTACTAGATAAC	ATCTGTATATACCATCAGACTATTCAAA
2402	tarc2260	CoreSet	6B	415.9	CTCACAGGAAAATCCTCAACCAT	GGTAGGAGTCATCGTATGATCTGC
2403	tarc1161	Other	6B	417.2	GCACCAGCTAAAAAGGTACGGA	CACTGGAATGATACTAATGCGGAC
2404	tarc2267	Other	6B	417.2	CATATGTCTATTATGGCTCTCGGTT	TAACAGCTTGTATTATCTAATTTTGAAT
2405	tarc1193	Other	6B	423.2	GCAACAGCACTTGCCATCCT	TTAAAACTATATTGTTCATTTATCAACG
2406	tarc1146	CoreSet	6B	437.5	AGAGCACAAGATAAATGTTTTAAGAGAG	TGATACAATCCGTGTATCCCTGA
2407	tarc1155	Other	6B	438.9	TGCGTACAATAGACCCAAAGTGAT	TGCTTGTCCTATTTTTATTCCCG
2408	tarc1192	Other	6B	439.1	CTTGCATGTTGTAGCTTGGAAAT	GTTGATTCTCATATAGATCAGGGACTAT
2409	tarc1163	Other	6B	440.8	CTATGGTACTTATCCTAGTCCATCGTAG	TCTCGTAGCTGGACAGAAAATGA
2410	tarc1176	Other	6B	446.8	GAACCCAATCGCAGAGCC	TGCCTACTCAAACGTATGTATCGA
2411	tarc1168	CoreSet	6B	447.1	CCTGCCGGTGACGACACA	CATCAGTGCGCTAGTGTGAGTACA
2412	tarc2244	Other	6B	447.1	CTCTTGCACCAACTGCAACAT	CTGGAATCGAGCCCCCAT
2413	tarc2258	Other	6B	447.9	AGTTATCAAAGTCAGCACCAATCTG	TGATGCTTCTGCGGTCACTG
2414	tarc2257	Other	6B	451.9	CCCTAGCTATCCCAGGAAAAATATA	CTTGGTTGCTGATTAATCTTACCC
2415	tarc1178	Other	6B	452.2	AAAATGGTTTGGAAGTGTATTTCG	TCTAAGATTGCCAGAGTTAAATGGTT
2416	tarc2253	CoreSet	6B	457.2	CATGGCAGGAGACTGGGTATAA	AGGAAATAGAAATCATGAAACAAATATC
2417	tarc2251	Other	6B	459.6	AAAGTAATTTAATTTACAATTGAAACTT	GTTGCTGGTTTCGAGAGATGAA
2418	tarc2256	Other	6B	459.8	CATCAACAGGAACCTGGTAATATAATG	TTTCTAGTGCTGATTGCTAAGGCT
2419	tarc2266	Other	6B	460.1	CCTGTCTTACAGAATGCCAATGTAAT	CAACAATTTCTAAAGTGAAGAAAAAAAT
2420	tarc2264	Other	6B	460.5	TCTCAATTCCTGATTGGCGG	ATCAAGTATAGTCGAAGTACAGCTTTGT
2421	tarc2268	Other	6B	460.5	ATTCATTTAATTGTTGAAGAGATTTTAT	AATATAATAGACATTATTCACACATCGG
2422	tarc1154	Other	6B	461.3	TCTATTTTCAGCTTAATCGACATCC	GATGACACCATGTATGTGCTGATTAT
2423	tarc2249	CoreSet	6B	461.3	GCGATGTGTTCCTTAGCTCATC	AGCTGTTGTGCCTGTATAATAATAGC
2424	tarc2265	Other	6B	470.8	AAAAAGTATTGTGTTCAAACTACGTTAA	GTGTAACAATCTTTAGTTAGCTGCTGTA
2425	tarc2252	CoreSet	6B	470.9	ACCTGCAGGTCCAGAGGTCA	TACCATACAGTGCTGTCCTCAAGA
2426	tarc2263	Other	6B	472.6	GTCGACTGTTACACATCTTATTGATGT	ATTACACATAAGGCGCAAGTGC
2427	tarc2243	CoreSet	6B	475.7	TCGTACGTTGACAACATTTTTTTTA	GAATTGTTCTCCCCTGTTTTTG
2428	tarc2272	Other	6B	489.8	TCCAAACAAGCCCCGCTC	GACCAGTCACACAACCTACATACATA
2429	tarc1181	CoreSet	6B	497.0	AATGCGCCAGCTCTTGCC	GTGTGCCTGTGATGTCCTACATA
2430	tarc1160	CoreSet	6B	523.2	TCCTGTTCAGCTATTTCATGCC	GTTAACCGGCAAAACAGTTATATCT
2431	tarc2247	CoreSet	6B	525.1	GTATTTTCATATGCTTCAGGAACTAAAG	GCTTCCTGGTTTAGTGCACATG
2432	tarc1182	Other	6B	539.6	TCAAATGCAGGGAGACAGGATA	GGTACATTTCTCACACACTTAAAAGTAT
2433	tarc2255	Other	6B	552.2	TACTCATGAAAAAAAAGGGGAACAG	ACATTCCATAGTACTACCAGCAGGTT
2434	tarc2269	Other	6B	555.3	GGAAGGTTGCACTCTAGGCGTA	TCGGTTCGAGAATAACAGTACCA
2435	tarc2239	CoreSet	6B	556.5	CAGAATGATTCTTTTGCTATCGTTAT	CCTAGCATAGTCAAGGGAGTACACAA
2436	tarc1172	Other	6B	560.6	ATGTTTCACTTTCACTGCTCTTTGTA	CGAAGCAAATATATACAGACAGAGAATA
2437	tarc2262	CoreSet	6B	570.1	TTGTGCACTCCACTGTAATGTGTT	CGTTCCTCGTTAAAGGAACCA
2438	tarc1180	CoreSet	6B	578.4	TATCGTGAATGGCATGAAAAATAA	TTAGGTGCTCCTCATGGATGTTC
2439	tarc2259	Other	6B	582.9	CCCAATTCGTTCTAGTTAGAAAATACTG	TGTTTTAGTGAGATAGATACTAATGCGT
2440	tarc2246	Other	6B	583.6	TGATCTCTTACATTCTGTTACATTCATT	ACTACTAAAACGGAAGGAATAAAGTTTA
2441	snp6599	CoreSet	6B	591.3	GCAGCCCTTTCTGTATTTATATGTCT	TCCAAATTATTAGTAGTGTCAATTGTCTAT
2442	tarc1200	Other	6B	604.2	CACAGGTAAGGCTAATTTGTCGTG	GAATGTAAGTGGGGCAAGAGC
2443	tarc1156	Other	6B	606.4	GGCTTCTACTATTGATCATTTTGTTCT	CCAGTCTCCGCATTCAGCC
2444	snp221	Other	6B	614.1	GTTACTGCTCTGAGCATTGGTCAT	CATCAGCCGGTCATTTCAGA
2445	tarc2271	Other	6B	618.9	TGCAGGTTACGTCTGAGTAACCTT	AAATTGCATTAGGCACACAATTAAA
2446	snp4339	CoreSet	6B	623.7	ATCGGTGTATCCCCTCTGCA	AACAAAGGTAATGCACTCACTCGT
2447	tarc2242	CoreSet	6B	623.7	TTCCCATCATTCAGGTTTTCAA	CACCTGCGCTTACATCGG
2448	tarc2248	Other	6B	632.9	TGCAGAGAAAGGATCAGGAGTGT	AGTTCTACGCCCTGCATGGA
2449	tarc1148	Other	6B	633.0	GCCATAAATTGCATGAATCGG	GTAAATGGTTCATCGCTATGATGTT
2450	snp670	CoreSet	6B	633.5	TCCCCATCCACCAATACCAC	CTACCCCTTCGTATAGTCATGATTG

Supplemental Table 4. List of amplicon sequencing primer sets for wheat.

No.	Name	Type	Chr	Pos[Mb]	F_primer	R_primer
2451	tarc2273	CoreSet	6B	633.5	CCCAGCCGACTTAAAAACGT	AAAGAAAAACAAACAGTGCCGA
2452	tarc2235	Other	6B	633.9	GACACCGCTCGAGAGACAAGA	GGTCTGGAGAGGCGACAAATA
2453	tarc2236	Other	6B	641.3	TTCATCTACCTAATGAAATACATGTGAC	TCTGCAGTGCCCTGTTATTTAGTT
2454	tarc2238	Other	6B	641.4	GGCCCCAAGATCGAGGAAG	AAGAGGAATGATAGTACCCAAAAGC
2455	snp1679	CoreSet	6B	642.3	CAAACATGATTAAGCCACTTTCG	AAGAGAGTGACGTCGCACTGC
2456	tarc2261	Other	6B	644.4	CAGTGGAACAAAATAAGTTTCATGTAGA	TTCTTTCTTATGTGGATCTTTATTCTTT
2457	tarc1165	CoreSet	6B	645.5	AATCTCGTCGTTAGAAAAGCAGG	TATTCGTACAAATGCCAAATGCT
2458	tarc2237	Other	6B	645.5	CGAAAGGATAAAGCTGAGAGAGG	TTCTCATAAGGATTTTTGCTCTGTC
2459	tarc2245	CoreSet	6B	645.5	CAGTCAGGTTGAAGCTATACTGAAAA	TAGTTACATCATTCGATGACCACG
2460	tarc2254	Other	6B	645.5	GGCCGCTCTTACTCCTGCT	GGAGAAGACGATGATACAACACGA
2461	snp634	Other	6B	652.6	GTTACATGTGTCTTGGCAGCGT	CCGTGCAACATCTAACATGCAC
2462	tarc2240	Other	6B	652.6	TCAGTGGAGCAGCTTTTTGGTA	GGCGGTGACTGAGCCTCAT
2463	tarc2604	Other	6B	659.6	GTCCCCTCAGCCTGACTCTG	AAAGGAGGGAGCAGGGCA
2464	tarc2250	CoreSet	6B	675.4	GTTTGAAAACTAAGCGTGCATCTAT	CAGTGAAAGGGCATATGCTATCC
2465	tarc1151	Other	6B	685.3	GAGCTCAAGACTCGGTTCAGCT	CAAACCTTGCAGAGATCAGCG
2466	tarc1177	Other	6B	686.0	GCTGTTTAATCCTCGACAGTGTTT	TTGACCTAAATTCTTACCTCTCATCAG
2467	tarc2270	Other	6B	692.0	AGGTCGAAGAGACGTTCCAGAG	GGAACATTACTTCCAGCTACTTCAGT
2468	tarc1179	CoreSet	6B	694.1	CCTGAAACAAAGAATAGGTATTTTTATA	TTGAAATATACCTTGCAGAAATAGACA
2469	tarc1144	Other	6B	705.2	GGTTTTGCTGATGCTGGACA	GCATTGTAGACTCTGGTCTTTAGGAA
2470	tarc1143	CoreSet	6B	718.9	GCTTATCTGCTATTTTGATCCTTGTT	GAGGACAAAGTTATGAAGGCACA
2471	tarc0319	CoreSet	6D	0.8	CACTTGCTCCATCAGGTTTCG	CACATCGCATCTGCATCCA
2472	tarc0326	Other	6D	0.8	TTCAGTTAGTTGTCCAGTATGGTAATAGT	GTTGGTGTTAAATGCTTGCTGTAA
2473	tarc0312	Other	6D	1.4	GTGCAGCCATTTGTCCAAGT	TCTTATGCAGAACTAGTCGGCG
2474	tarc1420	Other	6D	1.6	AGCTCATCGACTGACCAGAGAAC	CCTGCCATCACCACTCCCT
2475	snp2338	Other	6D	1.8	CCATATCATATATTTTTTTGGGAAGT	TGAAATAATTTAGGCAAAAGCAGC
2476	tarc0329	Other	6D	1.9	GAACAGAGAAGGTTCTGAAGTCATTT	CCACTCTCGCTCATATTATATGCA
2477	tarc0315	Other	6D	2.0	CAATATGAGTGTACAAAGTGCAAGTTAT	CGTCAATCTTTACATGTTCCACAG
2478	tarc1281	Other	6D	2.2	GCTGCCTTGCACTAGATGCC	TGGTTATGAACAGCTAGACTAGAACACT
2479	tarc1492	Other	6D	2.2	ACATGGGCCGACAACACG	GACCGTTTTTGGTATCTTCGC
2480	snp984	CoreSet	6D	3.4	CTGATCCACCAATTCCGTATCTT	GGAAATCAGTATTCAGCTAGTAGCG
2481	tarc2484	Other	6D	3.6	GAATTTGGAAAAGAGGGTACTACAAC	CTGAAACAGAATACATGCTCTGCTC
2482	tarc1267	Other	6D	3.8	ATGAGTTTCCAGGTGCTAAACATTA	TGCGGTGATGACAGTGCTTG
2483	tarc0318	Other	6D	3.9	GAGGAGCTGTTGATGGGCTG	GGGAGAGCATCTTAGTCGTGG
2484	tarc0320	CoreSet	6D	4.0	AATCAAATTGTCATGGCGTGG	GGACTTTTTTGCTAGTGTAACATAGATA
2485	tarc0322	Other	6D	4.8	TCACTAATTCTTGCTTAAAATGAACG	CGTTGGTAACAAAACACTGGGT
2486	tarc0324	CoreSet	6D	6.0	AGTAAGACCAATTTATTAGATCAATGAGC	CGGAAGGGATTTATACCAGTCG
2487	tarc1460	Other	6D	6.3	CACGATCTCATTAAACTCCTCCG	CGTGGTGTTTGCACCCTATG
2488	Inf15852	Other	6D	7.0	GACATTCTTCGCTGGTGATTACAC	GCATGTTATTTAGTGCAGCATACAA
2489	tarc2483	CoreSet	6D	7.1	ACATGGCATATTCAGGTAATATACGA	TTGACACAGAACCGTGCGG
2490	tarc2489	Other	6D	7.1	GCAACCGCAGCATCAGTAGT	TGTGCAGGCCTGCTAGCG
2491	Inf16385	Other	6D	8.7	TGAGTGTTTTCCCTAGAATCTGTCC	GGAGGTGCACACCTCAGCTACTA
2492	tarc2486	Other	6D	9.4	CCTGGCGACAAAACACACATG	TTTAAGCTGGAGACGCAAACTG
2493	Inf41669	Other	6D	10.9	TACTCAAGTGCTGAGGGTGGTG	CCCATCATCTGTAGCAGATCAGTTA
2494	Inf18587	Other	6D	13.6	AGGTTATCATTTCTTTCTTACAAGGG	AAATACGCAACAAAGTGTTTAATATTG
2495	tarc1295	Other	6D	14.6	GTACACGTACAGTGCGCGTATTA	TTCATTTAACTTTGGAACCGAGG
2496	Inf55850	Other	6D	18.0	GAGTGGCACTCTTACCGCATG	GGAGAAAACAAACAGAGAACAGTG
2497	tarc0325	CoreSet	6D	23.4	CCAATGTCAGAACCGACCG	CCGATAGGAGGGTCGAGTATGAT
2498	tarc1407	CoreSet	6D	24.4	GATCAATTGCTGATTGCCCA	CGTAAGCTGGACTGGAATGTTC
2499	tarc2480	Other	6D	24.6	CCACCAATTAAGTTACTAGATGCGAC	GGTGCAAATGGAGGGAATACTG
2500	tarc1471	Other	6D	45.9	CGCAAACAATCTACTGCCACA	TCACAAGCTGAGCCTGAACCT
2501	Inf56138	Other	6D	45.9	AAGACCGGCATGGATGTTTG	CCGTTCCTCGACCTCTCGT
2502	tarc0321	Other	6D	47.7	CAGATAGTTCTGGACTACAATGATCAGA	TCGATTGGCTTGCCGAATA
2503	tarc0314	CoreSet	6D	52.5	GGTGAGAACTATCACAGTGGCATAA	ATTTTTTTAGTACCAGTTTCTGAGCAT
2504	tarc0316	Other	6D	52.7	TCTGTTTCTGTAACTGATTAATGAACTGT	GGTATGAGCAAATCTTTTAGCGT
2505	tarc2481	CoreSet	6D	52.7	AAGAAGTGCATTTCCTGATTAATAATC	ACCAAAAAAAAAATCATCTCACATG
2506	tarc1516	Other	6D	52.8	ACTCTGAAATGAATCTCTGAACTAATC	GTTGTTCATTGCTTTGCAACTTCT
2507	tarc1387	CoreSet	6D	53.8	AGTCTTGCGCATGGTCACAC	TGTCAGCTTTACACACCACATCAA
2508	tarc1310	Other	6D	55.9	GACGTATACATTTTGATATTATACATCC	GAAAAACTTCCAAACAAAAAAGGTATA
2509	tarc2488	Other	6D	56.6	AGAACGTGCGGCTGGATC	AAGAGCAAGAGGAAGAAGAAGAAAG
2510	tarc1447	Other	6D	58.0	GTTGAACCATCTTTCGAGAACG	CCAACTATTGGCAAAACAGGGA
2511	tarc0328	Other	6D	59.6	GATCTCAGTGGAAATGCTTCCA	GCCTCTATAGCACAGGTGTCAAA
2512	tarc0317	Other	6D	62.1	CAGCTTGTAGTTGATCTTGTAATTCAT	CGATCATTCATGCATTGGACTG
2513	Inf36205	Other	6D	65.9	TTATGTTCATATGCACTAGCTACATGAC	ATGTAGAAGGTGAGCCAGTTGTG
2514	tarc0330	Other	6D	69.9	GTAAGGATGAGGATTCACTCTGCA	TGATCACACAACTGCATGTTACACT
2515	tarc1284	Other	6D	79.1	ATCATCTAGTTTTTACACTCGTAACCAC	CTTGGTCACCCCTGCTTTTG
2516	tarc1355	CoreSet	6D	81.8	CGGGCAGGACAGCTTGTAG	CTATTTTGGAACGGAGGGAGTAG
2517	tarc0327	Other	6D	82.6	TTACAGTTGGGAAAATAATAAAGAAGG	CATTTTATTCTAGGTAAGTGAGCGG
2518	tarc0323	Other	6D	86.1	ATAGTCCTCTTCTCTATAATGCTTTAACG	CGACCTGCAAATATTAACTTCGA
2519	tarc0311	CoreSet	6D	86.5	AAGCGCATTTTGGTCACTTCA	GACCAACCCATGCAGTGTAATTA
2520	tarc1476	Other	6D	87.5	GAACATGAGGTCCAAAGAACTCG	AACCTGGAACAAAATCAGGAGC

Supplemental Table 4. List of amplicon sequencing primer sets for wheat.

No.	Name	Type	Chr	Pos[Mb]	F_primer	R_primer
2521	tarc2482	Other	6D	91.5	GCTCGAAAATTCGGAGTTTCTC	GCGAAAGATTCATGCTTGACAA
2522	tarc1400	Other	6D	93.6	CATATTAAAGGAAATCTTTTAACTTCAT	GCTGTAGAATTTTGCACCATCTTAG
2523	tarc2485	CoreSet	6D	93.6	CAAGATGGCGCAACATTTTATA	TTGTTTGTATCTCATGATATTTTTTTAA
2524	tarc2487	CoreSet	6D	93.6	CATATTAAAGGAAATCTTTTAACTTCAT	GCTGTAGAATTTTGCACCATCTTAG
2525	tarc1427	Other	6D	95.3	TGCCACTGCTCAATGGACACT	GGCTGCAGGTCTAGTTGGGTA
2526	tarc1300	Other	6D	97.3	GCGACGAGGCTCAATCTGATAG	AGAGATGGAAAGATATGGAAGTGGA
2527	tarc0313	Other	6D	114.2	GGAGGTTACAGTGCTGAGGCAG	GTGGCTATATAAGGTGAAAGAAAACC
2528	tarc1436	CoreSet	6D	143.4	GAAGGAAACTAAGACGTGGCAGT	ATAATTTTTAAGTGTGAATACAGCCG
2529	tarc0290	Other	6D	227.4	CCTGGGAGTAAACAATCGCC	CCTGGGAGTAAACAATCGCC
2530	tarc1261	Other	6D	227.4	ACCTGGGAGTAAACAATCGCC	TGTATAGCTTTCTGTTTTACTTAACTGA
2531	tarc1349	CoreSet	6D	267.5	TTCTACATACACTTATTGTCTTTTCTCC	ACTAAATTCAATAAAGTTGCATAATGGA
2532	tarc0302	CoreSet	6D	269.1	CCGTATGTTATTGTTTCCGTTGC	AGCACAGCAAGAATTATACACTTACAA
2533	tarc0306	Other	6D	270.1	CCACCAGGAGGTTCGTGGTA	TGTGGATTATGCTATGCAAAGTAGTATT
2534	tarc0289	CoreSet	6D	271.6	GCATGATTGAATTATTTATCCTCCA	TCTAGTCATTTTAACTCCATGCACATA
2535	tarc1330	Other	6D	273.8	AACGTAGTTAAATTCTACCGCCG	CCACGACTGCTTTTCCCTCC
2536	tarc0307	Other	6D	296.7	CCAGTTCATGGAGTGAAAGCA	CAAGTATCACATCAAAATCATGTCAAC
2537	tarc0296	CoreSet	6D	304.8	CGACAGCAATTCAGTGGATTCTAG	ACATTGGTCAGCTACATTTTAATCCT
2538	tarc1403	Other	6D	305.7	TGCATCATATACAATGGGAACGA	TCATTGTCCCACGATTAAAGTTATTA
2539	tarc1425	CoreSet	6D	307.9	TTAACCAGCGGTACTTTAACGCT	CAATCAGGTTTCTTGGTCATTTATTC
2540	tarc0309	Other	6D	308.3	AAAAAAAGTCCAGAACTGACAGGAG	ACACCCTCTGATTACAGGAACGT
2541	tarc0297	Other	6D	310.0	GAACACCACGGTACCACTTGG	GGAGTGTATATTTGCGACCTGGAT
2542	tarc1401	CoreSet	6D	330.1	GGGTGACGGATAAGCTCCTCTC	TTATGGCGAACCATTTGTTGTT
2543	tarc1524	Other	6D	331.6	CACTGACTGCGCTGCAAAATT	CATCACTTAACAGATCTTGAGACATACG
2544	tarc1515	Other	6D	351.1	TCCCTCTGCCTGGGGAGG	TGGTGCTCGGCGACCTTAT
2545	tarc1394	CoreSet	6D	351.2	CATTCAGGAAAGACAGTTAGACGG	TGTAGCTGCTGCTGCCGC
2546	tarc0287	CoreSet	6D	355.9	TTTTGAACTTACGAACATATTACAACC	AAGCCAGCAGAAGGATAAACATTA
2547	tarc0308	CoreSet	6D	363.5	CGTCACGTCGTCAAGCCTG	AAAAACCAAAATCATGTACCTGTGT
2548	tarc1344	Other	6D	370.8	ACAGAACATCGGGCGGGG	AATATTGGATGGTGGTTCTGTTTTT
2549	tarc1439	Other	6D	373.4	CAAACATTAATAGAAGAAGAACGAGACA	CAGTTTTGATACTGTGACTTTCAAGTG
2550	tarc1512	CoreSet	6D	381.3	TTCTTTAATAACACAGAAGTGCTTCCT	TCTAGTCGTTGTTTGCCCTCG
2551	tarc1437	Other	6D	383.7	ATGGCTTCTATTCTGTCTTCTGTTTTA	AACTGTTGAGAGTCACTCCGTAAAA
2552	tarc1369	Other	6D	384.4	GGGCTGTGGTGGAGAAAGAGA	TTGCACACATCCTGTGCTCAG
2553	tarc1541	CoreSet	6D	387.0	AAAATATCAAGCCAATAAAATTCACC	GGATTAATTAGTTTAGTTTGTCCGAGTA
2554	tarc1352	Other	6D	391.9	ATTCCACAAGTGCCTGAACATAA	TACAAAGAAAAGACACTCCAGCG
2555	tarc1496	Other	6D	401.2	GCCTCAGACACGTTTTGGGT	GGTTAGGTGAGATGAAGACATTTTG
2556	tarc1529	Other	6D	401.3	ATTAATAATTTGCCGGATCTGTGT	CTTCACGGGTGAACGGGTAT
2557	tarc0298	CoreSet	6D	402.1	CAAGGAATACAAGAAAACAAGAAAATATAC	TCTCAGCAAAAGAATTGATGCTG
2558	tarc1312	Other	6D	403.8	TAATTTTACATTAGCAAGTGCATCATT	TATCTATACATCATGTTGGACTGTGGA
2559	snp2175	CoreSet	6D	406.3	TACCTCTGTTTAATAATGTCTTGAATTTAG	GCATTGCGTTGAACTTCATGA
2560	tarc1497	Other	6D	408.7	GTGGAAACATCTAATATGAAGAATTTTG	GGTGATGAAGGCGGCAAAT
2561	snp7616	Other	6D	409.3	ACTTCATCTTTCAATTTGCAGATTG	CTACTCGCAACTTCACAAGATGTG
2562	tarc1467	Other	6D	411.9	ACTGTTATTTCTATACGAACTGTAGAGA	TGTATGAAGTTTTCCTTGTCTTTTTTT
2563	tarc1363	CoreSet	6D	414.0	GAATATCTACAACGTGGTCCAATAGT	TTGTCCGGGTGGAAAGCC
2564	tarc1428	Other	6D	419.3	TTCTACCTATACATGTGAAAAGATAACT	AAAACAGATTAATTGTAGATACAAAGTG
2565	tarc1296	Other	6D	420.4	CCGTGAAGTACAAGACCCTGATCT	AAAGAAAATAGTCAAGTTTTCAAGTCAG
2566	tarc1465	Other	6D	421.9	ATTTGGTGTTACTTTGTTCATCTGTAGT	GGCTGAAATTTTCCCACAAGA
2567	Inf18585	Other	6D	424.8	CAGATTTTGCAGCATTCCAAAC	TTGTTAAAATGCCTGTATCTTTTCC
2568	tarc1408	Other	6D	430.8	TTCCTCCCCAGGGGCACT	AAATAAATGGGCAGTTTTTAATGAAG
2569	Inf31129	Other	6D	431.3	GTTATATTTAGCAAGTACCTATGCCTG	AAGTTGTTGCAGATTAATAAATGGCT
2570	Inf17785	Other	6D	435.3	TAGATTTGTCTAGATATGGATGTATTGC	CAATCGCCTCCGTGCTCG
2571	tarc1286	Other	6D	436.9	TGGATAGAAGCATCTTTTACATTTTCT	CAAATGTTGGCAACTTTTGGTTT
2572	tarc1445	CoreSet	6D	436.9	CCTGAAGCTATTTCTAGGGCACAT	CAACATGCACCAAGTATTATTAACG
2573	tarc0293	Other	6D	437.9	GACATGGCATTAGCACCATCTG	GTGGATGCAACTCAAAAACATGA
2574	tarc1468	Other	6D	441.9	TGAAATGATTCTCTCTAGTCTGGAAAC	CACAAAGTGTGTCCTCTGCCG
2575	tarc1411	CoreSet	6D	448.8	CTGAGCTTAGTTCGATGCTGAAAT	GTTGAGAAGGAGTATACAGAAACTATGC
2576	tarc2476	Other	6D	449.5	CAGTGCATGCATTGTGAACATAAC	CTGGCTAACCACCACGGC
2577	tarc0295	CoreSet	6D	450.1	GGTATGCGATCCACCTTTTGA	TTAAGGCTCCTTTACTATATGAGCAA
2578	Inf24244	Other	6D	451.0	ACATTACCTGACACACGACCTACC	CAATAAATGACAGTATTGTGCATGTGTA
2579	tarc1288	CoreSet	6D	452.8	TGGATGAATTGATGCAAGTCGT	ATGAAGACCTTGGTGATGAAGTGTA
2580	tarc2478	CoreSet	6D	456.5	CGGATGTTCTCCACGCTTG	TCAGGAACCTGCGTGACTCTG
2581	tarc0310	Other	6D	456.7	ACATACAAAGGATCAAATTAACACAGA	GTCCAAAACGCTGCACTCTTAA
2582	tarc0304	Other	6D	457.1	TGTTGTAACTCTGCATAATATATGTCTTCT	CAAAGTGAACTGTATAGGAGCCATTC
2583	tarc0300	Other	6D	457.8	TGTTCATTGGTCAGTTGTCGTGTA	GGCCTTCTTATCCGACAAACATA
2584	tarc0305	Other	6D	458.0	GCATCTTTGGTAAATCGTAGGAGG	AATGGGATTGGAAACATGAACA
2585	snp2965	CoreSet	6D	458.4	AGAAAAAGGAGAGGGTAAATCAGG	GCCACCGCTGACACGACA
2586	snp6939	Other	6D	458.4	AGGGACATTCTCCTCTCGCA	TGTTGCCAGATTGATGGGTCTA
2587	snp8060	Other	6D	458.8	ATTAATAATTCTATATAGCGACCAGCC	AACAAGAATTATCATTGCCAGCTAC
2588	tarc0292	Other	6D	458.8	TGCAGGGCTTGTGACAAAACT	CCACCAAAGCAATACCTTCCA
2589	tarc1311	Other	6D	459.8	ATTTCTATTTAGTCTCTTCAACTTCACT	TTTTCCTACAGAAAGATTAGTGTTAAGT
2590	tarc0288	CoreSet	6D	460.7	TTACTTAGACTTCGTTTGGCATTTC	ATCGATGGAAGTGAAGCAAGTAC

Supplemental Table 4. List of amplicon sequencing primer sets for wheat.

No.	Name	Type	Chr	Pos[Mb]	F_primer	R_primer
2591	tarc1390	Other	6D	460.8	CAAGTGTGCTGATGGTGATTAGATT	CTCCGTGATGCGATGTGGT
2592	tarc1412	Other	6D	461.3	ATATTTTATTGCTACATGATGACTAACG	CGGCCTTTCACCGTGGGA
2593	snp1924	CoreSet	6D	461.4	GATGAATTGCAGTAAGGATGACTAAA	TGAAGTTGTTCAGCATGCCATAT
2594	tarc0291	CoreSet	6D	461.4	GGACACGCATTGCATGTATCTC	TGTACTTTACTAATGCCCATTCTAACAC
2595	Inf21244	Other	6D	461.4	CGTCTTGGTGAGACCCACACA	GCGGTAACGTACCAGTCTCGT
2596	tarc2469	Other	6D	461.4	ACTGTAATGATTTGTGGATCTCACAC	ATCGATATAGCAAATTAACATGGAGAG
2597	snp6274	Other	6D	461.7	CGAACAATGCAAACTGCAGACT	AAGCTGTGGTCTCATTTGCG
2598	snp5591	Other	6D	461.7	CCATGAGTTCTTTCTTGATGTATGA	CCTTTGAATTTAGAGGGGGGAA
2599	tarc1282	Other	6D	461.8	TACAAACACACTAAAATTCTCATTTATG	ATATTGCAAAACTTTTCCACAAGC
2600	tarc0303	CoreSet	6D	462.3	AACAGTAGTTCTGAAGCGAAGCC	GGTGAAACCATAATCCATAAGTGATT
2601	tarc1442	Other	6D	462.4	TATGTGCCCGGAAGAAATGAT	CAGCCTGATCAATAAACATATTGC
2602	snp4056	Other	6D	462.5	AATTCAGAACCAGTTCTTTTATACTTGA	AAACTCTGAATCTAGCTCTCTTTTCC
2603	tarc0294	Other	6D	462.5	CTCCATGTTCCTCCGCTTTG	TGATCTGTTTGGGGTTCCAGAT
2604	snp5931	CoreSet	6D	462.6	AGTTGCGTTGCAAAGAGATCG	GTACCTGAACAAACTCTTTATCTTCATT
2605	Inf41118	Other	6D	462.6	CAAGGATTCCATCTTGTTGGGT	GAGGTGGTGAAGCAAGGTAATTAG
2606	tarc2475	Other	6D	462.6	CCAGAAGCATCGCTCTACTCGT	GTTAAAGTCTTTCCGGTCCACAC
2607	tarc2470	Other	6D	463.7	GGAGGGTGTGCTAGGTACTTCG	TACTCGCTCAAAGCTATATTACGGT
2608	tarc2477	CoreSet	6D	464.7	GATTGTTCCCCTGCCAAGTAA	CATAAGAGGGCAGAAAAGGCT
2609	tarc2471	Other	6D	464.8	ATGTTTCTGGATCTACACGATGATG	AAGAGCTTGGAGTCAGATGATGG
2610	tarc2479	Other	6D	465.1	AAAGCTTAAGCGCACTTGGTT	ACAAGAATGTGGTGTGCTAAATCA
2611	Inf11494	Other	6D	465.2	CGGGGCCGTTCTTATTCTTG	CAGCGACCAAATAAACAGAACA
2612	tarc2474	Other	6D	465.2	GCCGATTGCGGTCGTCAG	GAATCTGGACCCTGCCAACA
2613	Inf28366	Other	6D	470.0	TATCACCATTCTAAGAGGTAATGGACT	ATCTGTTCTTATCCTGACCAGCG
2614	tarc1413	Other	6D	471.9	GGTTCATTTGGTAAATGCTTTCTAA	AAACTAACTTGTCCTTTTAAGAGCAGA
2615	snp203	Other	6D	472.9	CAACTTACTTGGTGTCTGGTTCTTC	ACAAAGGCGCAAAAAATATACCC
2616	tarc0299	CoreSet	6D	472.9	AAGCATCTTATCTTACTTTTTTGAATACC	TCATCATCACTGCTATTCTTGAGC
2617	tarc2473	Other	6D	472.9	GACAGCCAAGGAGAAAGACATG	GTAGCAAGCCAGCAACAAAGG
2618	tarc1539	Other	6D	473.1	CAGTCGCAAATCATAGAAAGGTT	GGACTAGAAAGAGTTTCGGCATC
2619	tarc0301	Other	6D	473.2	GGTGGATTGTCGGTGCCAA	CATATGCCCAACTTTTTTTCGT
2620	tarc2472	CoreSet	6D	473.4	GGGATGCAATTGTTGTCTCTTCT	AAGGCATTAACTGGTTGACTCG
2621	tarc0742	Other	7A	1.7	TGAAGAGGACGACAATCCCAAC	GGTAGATGCGCCATGCCA
2622	tarc0771	Other	7A	4.6	CCAGGTAACGAACCAATATTTGTG	CGACCGACCATGTCGTTGTAG
2623	tarc1954	Other	7A	6.3	GTAAGCGAAGTGGCACCTCAA	AGTTGCGCTCTCCTGTAACGA
2624	snp3979	Other	7A	7.6	TCATCATAACTGGAGCTGGTGG	TTCCTGGTCCAACTTTGTCTGAC
2625	tarc0782	Other	7A	7.6	CACTCCGTTGGTGAAATAAATGAT	GGGTTCCATTATGCCTATCTGC
2626	tarc0807	Other	7A	7.6	AGAAGTCAGATTTTGGAACTAAACAGA	TGCAGCGCATGAGTGGGA
2627	tarc1947	CoreSet	7A	8.4	CACACATGTACACATAGGTGTTGTCTT	TTTCTTGTGACACTTGATCATCTTTT
2628	tarc0797	CoreSet	7A	12.8	CATATAAACACAAATCAATCCATTTTTC	GTTGGGAGTTCAGAACACCTTGT
2629	tarc1948	Other	7A	13.8	GTAATTGCATCCATTTTCTTGTAAGA	CCATAAAGAGATTTCAAACCACAAAC
2630	tarc1945	CoreSet	7A	14.6	GAAATCAGCAAAGGCCCTCT	TCCCTGTCTCCAGGATATATAATATAGA
2631	tarc1942	Other	7A	18.9	ACAATTGCTTTGACTACATTTCTCCT	GTCAGATTCAAATCTCAACCAAGAA
2632	tarc0769	Other	7A	20.3	TGCCAAGTAAATCTGTTTCTCCAC	GATCAATCTTGACAACACTATGCGTA
2633	tarc1941	Other	7A	20.3	AAAGATGCTGGAGGTCATACAGC	GGTATTTCGACAGTAGTGGTGAGGA
2634	tarc0787	CoreSet	7A	22.9	TTTGAGGATGTGGTACGCTTTCT	CTAATTGTGTGGTTGTTGTTTTCG
2635	tarc1952	CoreSet	7A	28.0	AAACAGATTAGCCTCATATTTGCAG	CAGAAGCGAACAGCATCGTAGTAC
2636	tarc0781	Other	7A	30.1	TTGACTGGATAACCACTGTCTTGAC	GCCTCGTCTTTCGCCTTTG
2637	tarc0806	CoreSet	7A	30.1	CTTCAAATGGGACCAAACACAC	CCTACTAGCCAGGACAACCGC
2638	tarc1955	Other	7A	31.1	TTCCAAAGCACTCTCAGGCTC	CATATGTTCTCATGGAGACACGC
2639	tarc0741	Other	7A	32.0	ACAAATAAAGTAGGAAACAATATGGTAA	TCATGGGAAGAAGACTTATTAAATGAT
2640	tarc1951	Other	7A	35.0	CCCTCCAGCAAAATTATGGTAAG	GTGACCAAGAATTATCCTACAGGTAATA
2641	tarc0775	CoreSet	7A	42.0	AGAGAGCAGAGCAATGGACCTTA	GAAAACAGAATTATTTTGCACCACTT
2642	tarc0759	Other	7A	42.3	TTAATGTTCTTGTTTCTGACATCTCG	TAAGAGCGTTTTTGACACATTTTTT
2643	snp4614	CoreSet	7A	53.0	CCTGCATCTGCAACACTCACTAT	AAAAACTTAACACCGAAATTCCAT
2644	tarc1958	Other	7A	59.4	GTCGCTCATACAGTTTTGCCA	TGGATGATAAAATCAGTGAGCTAGG
2645	tarc0758	CoreSet	7A	64.9	CCTGATAAAAATCGAGCATAAAAATT	TGCTTATTCTTCTTTTAGAGAAACTATC
2646	snp8390	Other	7A	67.6	GCCTGACTATTACCTCTTTTGCTTC	CATCAATCAGGTTGACGCAGAG
2647	tarc0777	Other	7A	67.6	CCTTCTACTAATGACATCTGACCACG	AACCAACCATATCTGGCATCC
2648	tarc0752	CoreSet	7A	68.8	TCAGCGAGTGTCCAAACCAAT	GCCCAAGGCAACGATGTG
2649	tarc1950	Other	7A	68.8	AGTGGTGTCGGCTTTCGTCT	CAAACAAAAAACAGGGAGGCA
2650	tarc1944	Other	7A	69.2	TTTTTTCAAGTGGCAGAATGATTAT	TATCCAGGGCAAATTGACTACG
2651	tarc1957	Other	7A	76.1	GAGGTTAGGGATGTTCAGGCA	AATAAGTTTAAATAGAGAACTGACACAC
2652	tarc1953	Other	7A	81.1	GGGAAGCATGACTTGAGAGAAGT	CTAAAAAGCTGAAACATAAAAACCATAA
2653	snp3831	CoreSet	7A	83.6	GCGAGGAGAGGCTGGAGC	CAGCTTTGTATGAATACATTTTGCC
2654	tarc1946	Other	7A	83.9	TTCCCTCCTAGCTACCATACGTC	GGACAGAGGAGGAGAAGAAAGC
2655	snp4426	Other	7A	84.7	GGAGCAGAACCTCGTTGCA	CAGTTAGCTTCACTTCCACTCTTG
2656	tarc0744	Other	7A	84.7	ATGCAACTGGTCACCTTTAGATTAT	TTTTTAGATTTAGTATTCCTACAGTGTG
2657	tarc1943	Other	7A	84.7	TAGAGAATGGGTGGTAAGTAATACAAAC	CTGTAGGAATACTAAATCTAAAAACAGC
2658	tarc1959	CoreSet	7A	85.2	TAGCTTTGCAGTATTATGCTGACG	GCTGACAAAATGCACATTGTTATG
2659	tarc1949	Other	7A	85.6	ATTGAACAATCAAATTAAAAGTCAAACT	AGCTGCAAAGTATGACAGTATGGATAT
2660	tarc0747	Other	7A	92.8	CCAACGATGCGGAGGAGG	CGGAAAAAAATCCCTTGACG

Supplemental Table 4. List of amplicon sequencing primer sets for wheat.

No.	Name	Type	Chr	Pos[Mb]	F_primer	R_primer
2661	tarc0788	Other	7A	93.1	AAGCACCTGAGAAGCCCAAG	GCAAAAGAGAGAATAAATAAATAATCAT
2662	tarc0734	Other	7A	100.8	TGGGAAAACCTATTTCTGTTGTCTT	CACTGATACTGCTTTTGACCGC
2663	snp4181	CoreSet	7A	108.8	AATGAATGTCCAAATCAATGTTGG	GAAGTACCAGAAGAGGACTGTCG
2664	snp4180	CoreSet	7A	108.8	GCTCGCCGATAACATGTACGT	TAGACCTTCTTGACTCACTGGACTTAT
2665	tarc1956	Other	7A	108.8	CGCCCGCCCTTGGTTTAC	TTTCGACTACACTTGGAATTCTATCA
2666	tarc0735	Other	7A	118.5	GCAGGGCTCAACCCAGGC	ATATCATCAATCTTTAAGATCACTCTGA
2667	tarc0786	Other	7A	127.6	GCGACATTATGATTCTTGATGATTA	TGCCGAGTATGCACTTCCGT
2668	snp7731	Other	7A	127.8	GGATTACATAAATAATGTTCTCAATGTG	TGGAGAAATATCCAACCCATAGTTC
2669	snp2820	CoreSet	7A	127.8	AGATTGAGAACTTTGACTGATGGC	ATTACGGTTAGTTTGCATATACGTTAGT
2670	tarc0753	Other	7A	128.9	GTGTTCACATATGGTTGGGCAG	TTGGGATTATTTGGTCTAGCCTC
2671	snp363	Other	7A	129.1	TTGTGTTATATGATCTGACTTCTGAGG	AATTCAACAAGGAGACAGTCCAA
2672	tarc0755	Other	7A	155.6	AAAAAAGTACCCTCGGCAGC	AGAACAATCGTCGTCTCCCACT
2673	snp3187	Other	7A	157.4	TCTGTTACCTCTCAATTATGGCAAC	GTTGCGTCAAACTCTGATACAAATAT
2674	tarc0740	Other	7A	159.5	ATGCAAAAAACATGACTTAGAATCATAT	AAACCAAGAGACATATAAGAGCAAATAA
2675	snp334	Other	7A	166.3	TTCACTAGGAGGAGTGAAGCTGTT	GTAAATTTAATGAGCATGGATCCTCT
2676	tarc0760	CoreSet	7A	166.3	CCATATTCGGTATGTCCAACAAGT	CTTCGGCTTCCGCTTCGC
2677	tarc0746	Other	7A	167.3	GGTTCAAATTGTGACAATAATAGTTATA	CTTACTAATATTTTCTTCCTGGTCCTAA
2678	snp305	CoreSet	7A	169.3	TAGCACATGGTACATCTTGTGTGC	TGAGTTTATTCTGTTCCGGCG
2679	tarc0778	Other	7A	174.9	GAATTGTGGAGATAATATACAGCTAAAA	CGAGTTATCTTCCCTGAACTACCT
2680	tarc0795	CoreSet	7A	175.8	CATTCAACTTTCCATCAGATGTTG	TTGCTTTCTGGTGCTCATTTGT
2681	tarc0770	CoreSet	7A	201.5	GCAGTGTGACTTGCGAATCTTTT	GAAGATAGAGCGTTGGTTTGAGC
2682	tarc0802	Other	7A	206.7	CGAAGCAAAGCATGACTATCGT	TTTCTGAAACGCTGCATTTATAATT
2683	tarc0793	Other	7A	211.6	TGAAACAACCTATACTTCTTAGTAACGA	GTCCTCTGAATTATGGACGCAA
2684	tarc0751	CoreSet	7A	217.6	TCAAGCTCGGCGTCCATG	TCAACCATGTACAGCACCTCGT
2685	tarc0754	CoreSet	7A	218.0	ATTATTGCTAATCCTCATCCTTTCC	GCACGCCCAATCTCATCTG
2686	tarc0772	CoreSet	7A	229.1	TGGCTCCTGTGCTGTGACG	AAGAAGTGAGGTATGAGCTAGGGG
2687	tarc0743	CoreSet	7A	232.6	CTGCTCCCCGTAAATGTTCG	CAACTCGGCGGTTCTGGAT
2688	tarc0776	Other	7A	232.6	TAAATGTGTTCATGTTAACCTCAAAATA	CAAGAATTGAGTTCTGAATAATCTGAG
2689	snp5887	CoreSet	7A	232.7	CAATATACAGGGAATGAGCAGACAA	CTTTTTTTTGTCAATAAGGAGGTGT
2690	tarc0784	CoreSet	7A	235.5	TGTAATAACCCAAATTCTGGCTAACT	TATTTGTTGTTGTTGTCTTAGTCGCT
2691	tarc0736	CoreSet	7A	236.6	TATGTGACTTTTTTAATCTCTTGTTTCT	TCTGCAATCAATGATCCCCC
2692	tarc0796	Other	7A	237.4	CAGCTTTTCAGTTGTTCATGCC	AGGAAAAAAAATGTAACATATAAAAATGG
2693	tarc0737	CoreSet	7A	238.0	TGCAATAACTATAATCCAAGTGTCGT	GCAGCACAGCCGGAGAATC
2694	tarc0785	Other	7A	239.0	CTTTCTACCTAAGAATGACATGTTAAAT	GCAGTGCAAATCTGTGATGGTAA
2695	tarc0739	Other	7A	253.4	ATTTCTGCTGATTTTAGTTATTTTATAT	TAGTGAAGTAAAGTTGTGCTATGTTATT
2696	tarc0783	Other	7A	260.9	CCAATCTGCTGGTTTTCCTGA	TTTCCCCACTGTACATAAAATACTCC
2697	tarc0768	CoreSet	7A	262.5	GTGTGCAGTATGCTTACTTAGATCATC	CGTTAGTTGCCCCATTCCG
2698	tarc1928	Other	7A	427.8	GAACAAATGACATGATGATGACTTCA	GATGGTGTGTGCTTGTTGCG
2699	tarc0764	Other	7A	435.7	CGAAAGGCAGCCTTACACACA	AAAAAAATACTGGACAGATCAGCG
2700	tarc0738	CoreSet	7A	436.8	ACCAGTGATAGTGCCTAAACCAGA	TGTTTCTCTACCATTTCTTTTTCCA
2701	tarc0767	Other	7A	438.1	GGGCAGTGCTCTTGCTGCT	CATTCGTGAAGCACTTTGTTATGT
2702	tarc0766	Other	7A	440.4	TCCATCCCTATCAACAACAAAAAT	GCCTTTAAGGAAAGCTGTCGC
2703	tarc0790	CoreSet	7A	440.7	GAATCACTGAAACTGCTAACTCCG	GCCACTTCACAAATGCAACAA
2704	tarc0789	Other	7A	443.4	ACCATTGTTGTCTGCGTGCA	GAAAAGAATATCAGACAGAATAGTCAGC
2705	tarc0798	Other	7A	445.0	GTTGTTTGGCAGTGCTCTTAACT	TGTTGCTGCGTTATTTGCG
2706	tarc0801	Other	7A	447.8	TGCTATAGAAGATCAGAACTCTGAGGA	CAACCCAAGGTAATTGATATAACGA
2707	tarc0749	Other	7A	452.4	CGATAGTGGAAGTTAGTCATGTTATGAG	CTGTGAACATTAAGCAGGCATTATC
2708	tarc0765	CoreSet	7A	459.0	CAATGCAGGGAAAAAAATATTAGTT	CCATCATTGATGTAGTTTGCTCTT
2709	tarc0794	CoreSet	7A	490.9	GGCCATTGCTACATCAAACCT	AAGCATTGATTCTTCCCCAGTG
2710	tarc0761	CoreSet	7A	491.0	ATTCTCCTTTTAAATTATTGCCATCTAT	ATCTCCAGGAGTTTTTGCATACC
2711	tarc0804	Other	7A	529.3	GGCAACCTAGCATTTCCCC	CTCCCTCTCGCTTTCTCCTG
2712	tarc1929	Other	7A	539.3	CGTGAGCATTAAAATTAACTCCTAACT	GATTTGTTGACACCGTAAGTGAATATA
2713	tarc0803	CoreSet	7A	556.2	GCGAGCTGCTTAGAGTCATACC	CGCTGGCAAGAAAAAGGC
2714	tarc1914	Other	7A	561.6	ACGAGTTATTTTCGACAGATTTAAGTC	ATGGTTGACAACATAACCAACTTCT
2715	tarc1915	Other	7A	561.6	TGTGGATGCTCACTTGCCC	GGGAGTGCATCGAACATTTG
2716	tarc1933	CoreSet	7A	561.9	TACAGGGTCTTAGGGACTCCTAAAC	ACAGCTCTCGAAGGTAATCCTCTAT
2717	tarc1931	Other	7A	562.9	AAACAGACCAGACCTGTGTGATACA	GAAGATATAGCATAACAACACCAACG
2718	tarc1922	CoreSet	7A	563.5	CATATCAAGCAACTCTGAGCAGC	ATTTCTGTCCCTGGTTGAGAATG
2719	tarc1935	CoreSet	7A	580.5	AGGATTCTAAGGGATGCAGTTAAAG	ACATTGCTTTGCATAAAGTCGG
2720	tarc1920	Other	7A	583.2	CAATTTTTGCAGTGAAAAGAAGTTT	TTGTTCTTCATTCCTGAAGTGATGT
2721	tarc1940	Other	7A	584.4	ATCTCGCAAGCTCTGGTAAAACT	GGCATAGTGTTCAGTGATTATCTTCT
2722	snp4062	Other	7A	585.1	GCACCTGCAGAAAGAGGGTC	TTCTGCCGACCGTAGGGG
2723	tarc0779	CoreSet	7A	594.6	TCTGGACGATTTGGTACTAATTCAA	CCTAAAAACAGAAACTACGCCATC
2724	tarc1917	Other	7A	595.1	ACTATCAGCTGCTCTGCAATGTG	AAGATTTGTGTCAGTGGAGGGC
2725	tarc1934	Other	7A	598.2	CGACGCACTTGGAAGCGC	GTGTGCAACAGGCAGAGGG
2726	snp2437	CoreSet	7A	608.7	GCGTCCCAAGTTGACAATCC	TTATTATTCACGCTGCAGTCCC
2727	tarc0800	Other	7A	608.7	CCTCAGATCCAAAATAAGTGTCGT	GGTAGTATTTACAAAACCAAGATTGAGG
2728	tarc1938	Other	7A	608.7	CAAACACATACCCCATTCCAATC	GAGGAGAGGGAGGAGGACG
2729	snp1502	Other	7A	610.9	AACTGAAGAGTTGCCGTGCAG	CTTATTTTAAGAAAGGTGATGACACTG
2730	snp808	Other	7A	610.9	CCATTTCTTTGCCTCCCACT	GGCCAAGAAAATCATAGTTTGGA

Supplemental Table 4. List of amplicon sequencing primer sets for wheat.

No.	Name	Type	Chr	Pos[Mb]	F_primer	R_primer
2731	tarc0762	Other	7A	610.9	TCATTGTTATGAGCTGCTTGGC	TGAAGAATACCAATGAAGTAGGACAATA
2732	tarc1925	Other	7A	612.4	CAGATGAAGCAAACTACGCCG	GCATGCAGAGAATTTGGGTCA
2733	tarc0750	CoreSet	7A	613.0	AACTATCCCGTAGGCTCCTCTTC	GGCACCCTAAACTAGTAAACTTCGT
2734	snp7432	Other	7A	625.7	GCAGATTGGGCAGGTCACA	CATGCAGGTTGTGATGAAAAGC
2735	snp7549	CoreSet	7A	625.7	CCCAACCATATCTCACTTCCGA	CTTTGGAAACAGATGCTTCTCG
2736	tarc1919	Other	7A	635.5	TCTCATAAGCTGCAGGTAAAAAATT	AAGCAACGGACACAACGTAGTT
2737	tarc0792	Other	7A	638.2	AGTTATCCCGTGGCTGATACTAAA	CGGTCAAGGAGAAACTATCAAAGA
2738	tarc0757	Other	7A	641.8	TCAATCTTGGCAAGACGTGGTA	GTGGTCGGTGCTTGGGTTA
2739	snp593	Other	7A	642.0	TAGTATAGAAAATGGTAGGAAAAGCCTA	GCCGCAGATGAGGTGTTCAT
2740	snp2775	CoreSet	7A	659.4	GCTAATAGCTACCGCCAAAGGT	GAGATGAGCACATATACCGTGTTG
2741	tarc0774	Other	7A	659.4	GAGATGAGCACATATACCGTGTTG	GCTAATAGCTACCGCCAAAGGT
2742	tarc1926	CoreSet	7A	671.0	CAGCCTCTATTTCCTTTAGCTCAA	CACAGTTCTTCGACAAATTTAATATCC
2743	tarc1911	CoreSet	7A	671.4	GACTTGAAGCTGCAGAACGCT	CAAAAACTTTAGACTGCAAACAACA
2744	tarc1927	Other	7A	671.9	TTTGCATGAAGAGTTGGAGAGG	GCCCCAACCATCAGAGACG
2745	tarc1916	Other	7A	672.0	CAAAACTCAAGAAGTTAGTAGTATCAAG	CGTCAAAGGCCAAGATGACTG
2746	tarc0791	Other	7A	681.0	GGATGAGATGAGATCAAGCCG	GGAACCGCATGTATTAGTTTATCAA
2747	tarc1936	Other	7A	683.5	GTGTATCATCAAGCATACTAAGCTACG	CGCATGGTTCGCTTGGAATA
2748	snp4187	CoreSet	7A	692.3	GCTCAGTTGGTATAGCAGGAAAA	TCATCACCTGTAAATAGAGAATAGCG
2749	tarc1912	Other	7A	692.3	CCTCGGCTCCTCCAATTTCT	GCCGCAACTTCATGGTAGACA
2750	tarc0745	Other	7A	692.9	CAGAGTCTTCCTGTGTGTGTTCG	CATCATCAACTTGCACTAAACCATC
2751	tarc1924	CoreSet	7A	692.9	TGTCTTTTTCTTTGAACCTATCTTACG	CATAGAGCTGAAAGTGCTACTGGA
2752	tarc1937	Other	7A	701.4	GTACCATTGAAGTGGCGGC	GCGTGCGTGCTCACACAC
2753	tarc1923	Other	7A	701.5	TAGGCGAAACATCCCCAAGT	ATTTTCCAGCATCCAATAGTCATC
2754	tarc1913	Other	7A	701.7	CGGGAGAACATTAGTATGTATCGG	GGAATGAATGCTGCATGGC
2755	tarc1930	Other	7A	701.7	GTTTGTACCTAATCTTGTACTATTTTGG	CTGGCTACAGAAAATGCTGTGG
2756	tarc0773	CoreSet	7A	706.8	GCTTCTCAAGTGACTCAAGCCTA	TTAGGAGTCTGTCGACGAGTGGT
2757	tarc1921	Other	7A	706.9	GCCACGTGTTTCAGTTTGTGC	AGAGGTAGCTCCAAGAAATATGAGAA
2758	snp7184	Other	7A	708.1	CTGAGCAAAGATGTAGAAGATGTCAT	CAGGATTGGACTCGGTTGGA
2759	tarc1932	Other	7A	708.1	GAACTGGACTCAGGGGAACTTG	AAACTCTTTACCCTCATGATCCAA
2760	tarc0799	Other	7A	709.8	CCTTCTTCAATTGGCATTATTTTG	TTTCCGGCTGCCTTCGTT
2761	snp2929	Other	7A	712.1	TTATTATGCACTCTTACTTTCTAAGCATT	AAATGGTTGAACTCCACTTTACAGT
2762	tarc0780	CoreSet	7A	714.6	AGTACATCAGCAGACATTGCATTCT	GTTGAGCTTTCTCGACTCTGGTC
2763	snp1271	Other	7A	715.7	TCATCATCGCCATCAACAAAAC	TGATGTCTTGTACCATCATTGTTTG
2764	tarc0756	Other	7A	719.6	CTCATCAGCCATCAAATATTAGCTT	CTATACCAGGCTTGAGGAAATAGTC
2765	snp5873	CoreSet	7A	724.1	CGATAATTTCTTCGCAGAAAACG	GCAAGCAGAAGGGGATGCA
2766	snp179	Other	7A	724.1	ACTGCCTTGGAGCCTCCG	CCCATGACTCGTGTCGTCA
2767	tarc0763	Other	7A	724.1	GAAACGGTCTTGCCTGCG	TGACTAAAATACTCTGTGAATTAAACTC
2768	tarc0805	CoreSet	7A	726.8	CATAGCTAATTCTTAAAGATGGCAAA	CGGTTCCCGTCTAAAGCTGATA
2769	snp7592	CoreSet	7A	731.3	TGTTACTCTGGATTTCAGGTACTGTG	CAGAACTAACATGTCTTTCAATGCC
2770	tarc0748	CoreSet	7A	733.4	CATCATGAGTAAGGAAACACACGA	TCTTTGCATCTTTCATGAGGCTA
2771	tarc1939	Other	7A	733.4	ACAATCAGGACATCCGACACTAAC	TGTACCTGACTGGAAACCTGAAGT
2772	tarc1918	Other	7A	733.8	CCACCAACACAAAAGTGGTATACACT	CGTTTGTGAGGAGTGTGAGAGTC
2773	tarc1253	CoreSet	7B	2.9	GATTTGAGATTTTTTACCAGCCG	CAAGATAAGAAAAACTACTATAAATGGC
2774	tarc1217	CoreSet	7B	4.9	GGCACACAGAGGTAGCACAGATT	ACTCCTTACTTGCTCCCTGTGC
2775	tarc1254	Other	7B	4.9	AGCCGTCAGGGCCAACAT	CGACAAACGACCTTCAACAGC
2776	snp1525	CoreSet	7B	5.9	GATGATAAGCCATCCAGCTCTTG	CAGTAACACAGAACCCTTTCTTATGTA
2777	snp1089	CoreSet	7B	15.6	TTCGCCGGTTCGAGAACAT	GACAAGACAACATACACAGAAGGC
2778	tarc1255	Other	7B	18.2	ATCACTCGTACTGAAGAGCTAAGAAGA	GCCTGTCATTCCACAATCGTAGT
2779	tarc1207	Other	7B	41.5	AAGAGCATAGACATCAGAATGAGGTT	ATGACCTGCATGAGTACACATCG
2780	tarc1244	CoreSet	7B	43.9	GCTGTATGGAGAAGTTAACAGTGCTA	CCATGTGTTATATTGTTCAAGTAAGTCC
2781	tarc2326	Other	7B	47.0	AAGCGGTAAGAATAATAAAAGAAAATG	CTCGCTTGATCCTGATGGCT
2782	snp4017	Other	7B	48.2	GAGTTCTGCACCAAATGTGCA	GCATCCCAGGTGAACTGAGGT
2783	snp4018	CoreSet	7B	48.2	GCAGGCTTGACGAGACGTT	CAATGAAATGATACCACAAGTCTTGT
2784	tarc1248	Other	7B	51.1	AAGCATAAAAAAACACAGGAGACG	GTTGCGATTCTAATGATCCATAGAC
2785	tarc1219	Other	7B	53.6	GGAAAAAAAGGAACAGCAGACG	GAGGGAAGACGACGACGATG
2786	tarc1237	Other	7B	59.1	TCTTTTTATCCTGTAGCTGGCAAC	CATGGAACAAGAAAATGACAACG
2787	snp518	CoreSet	7B	59.6	GTGGCCAATTTACTGAATCTGAAC	TAAGCAAACTGTATCAGCATCCG
2788	tarc1246	Other	7B	59.6	ATGGCTCTAATATCGGTCTTCTTTC	AATTAAGTTTGATAAACCTATACCCACA
2789	tarc2337	CoreSet	7B	62.7	CTCTCGTACTGGTTCGACTTCG	AAGCGGCAGTCATGGTGAA
2790	snp4516	CoreSet	7B	64.7	TTTTGTTTTTTGGACGGCACT	CCCAGGTCAGCTTTGATCACC
2791	tarc1227	Other	7B	64.7	AAATACTGTACTGGTAAATTAACTTCCT	TTATTGTTATGGATCGTTGACCCT
2792	tarc2327	Other	7B	64.7	AGAGTGACTTGTAGATACTTAAAATGCC	AACACCTTAAAAAAATCTTTATAACTTG
2793	tarc2329	Other	7B	68.3	TGATTTGTCTGTCTACCGTAGTTTCC	AGCACACATCAGTGAAGCTCTTATC
2794	tarc1239	Other	7B	69.7	GCCAATGTGATTGGGTAACG	ATAAGATCATATGGTAATAGCTGGATAC
2795	tarc1251	Other	7B	70.5	GGAGTAGCCGATCAAAAGGTCA	CATGTTAAAAAAAAACTGCCTTCTCT
2796	tarc1204	CoreSet	7B	100.0	TACAAATTGTTCTGTCGAGGGATAG	CATTGTGCTGAAATTGGGGC
2797	tarc1245	Other	7B	108.3	TGCCTAATTTTCTGTAGTTTGTGTTT	AATATCTCCGGCAACTCCCTT
2798	tarc1215	Other	7B	112.0	CCATCAGTAAACTCTAGGGAGGAAAG	CAGTATATCTTGTGCACCTATTTGACA
2799	tarc2324	CoreSet	7B	114.5	ACCGGAGCTTAGAAAGGCG	TAGCAAATATTTGCTTGGATGAGTT
2800	tarc1206	CoreSet	7B	129.5	TCTTCACAATTAAAAAAAATAGCGG	ACTATCTTGCCACTCCCCCA

Supplemental Table 4. List of amplicon sequencing primer sets for wheat.

No.	Name	Type	Chr	Pos[Mb]	F_primer	R_primer
2801	tarc1252	Other	7B	156.6	TTAATAACTCTGCATGTATGCTTTCAG	CTCTTCAGGCATCAAAGCGA
2802	tarc1212	CoreSet	7B	156.7	GAACAGCAAACACATTCTGCAA	AAAAAAATATCTCGCTCCATCCA
2803	tarc1250	Other	7B	159.5	GTGGTTCAGACTAAGCAAAAAAAGA	ACATAATGAACAGGTTACACATAGCATT
2804	tarc2333	CoreSet	7B	164.1	AACCCTCTGGATGCTCAACTG	TTGGACAAGTCTGCACAACGA
2805	tarc2325	Other	7B	215.0	GCAATCGTAGAAGTACCTACTTTTATAC	ATGAAAAATCTTTACGTTATTTTCTTGA
2806	tarc2334	CoreSet	7B	222.7	AAGAAGCATTCTTTGATGACATTATTAG	TTGTCCAAGAATAAAGTCAACGATG
2807	tarc2335	CoreSet	7B	223.2	GGGAAGTCGACGTCATGGTAG	GAATCAAATATATAGCAGAAGATTCTCC
2808	tarc2336	CoreSet	7B	228.8	ATACAAATAAACCAAAAAATCTCTGATA	ATGGCTCCAGAAAGTGCGA
2809	tarc2330	Other	7B	229.9	TGCAAAACTGTCTTCCCATTGT	CAAAAGAATTGTGGCTTATTCTCCT
2810	tarc2331	CoreSet	7B	231.7	GTTGAGAGAATACATCCGGGTGT	ACGGGCCTCAACCCTCGT
2811	tarc2332	Other	7B	231.7	ACGAAGTATGAGGCGTTCCG	CTACTGCAAAAGAATGGCCGT
2812	tarc2328	Other	7B	291.0	TTACAACTGCTACAAAATCACAGAAAG	AACATTAACAATTATGAATGTTGAAACA
2813	snp4380	CoreSet	7B	337.6	TGCAGCAGGAAGGTGGAAAT	GCACTGGTGCTCCAGATATTG
2814	snp4145	CoreSet	7B	393.3	TCCCCCAAAACTTGCACG	TAAGGGCAGATGGAAAACCG
2815	tarc1234	Other	7B	419.0	TGGGAGAGGTGTGGATGTGG	GAGTAATTTGAGGAAAGGTCCTATGT
2816	tarc1257	Other	7B	450.6	CTGATTAATATATTTGCTCCAACTTCTT	CATTTGGTCATACCCCTGGAT
2817	snp1261	CoreSet	7B	451.1	TTCAGTAACCATCCAAATCAATAGG	ATTATGTACTAGCAATACAGAATTCGC
2818	tarc1243	Other	7B	454.5	CCATTAGCCCCATCGAGCT	CCTGGAATAAAGGTGTTTCATCAC
2819	snp6322	CoreSet	7B	457.4	CAGGAAAGGGGAAGTGTTACATTAC	CATTTCAACTGGGCCATTGTT
2820	tarc1205	Other	7B	457.4	TTCAGCCTCCCAGAACGATG	CAGGAAAGGGGAAGTGTTACATTAC
2821	tarc1224	CoreSet	7B	480.3	CTCAGTGTACATCCCTCCTGAAG	TATTTTCTTTTTCCCCTGCTTGT
2822	tarc1213	Other	7B	494.7	CATCGGTTCTTCCATTTCAGCT	CTTCCAACTCCCGCCCAA
2823	tarc1240	CoreSet	7B	517.3	TAGCAATTAGTATATACAACAAGAATCG	TTAGCTTGCTATTGTACTTTGAGGC
2824	tarc1223	Other	7B	518.4	AGCCCAAAATCACGTACAACC	GTCAATAGAACAGAGTAGATGATGTGTA
2825	tarc1210	Other	7B	522.2	AATTCAGTACAAGTGAGATCAACGGT	GTTACCAGAGCCACCAAATTAATC
2826	tarc1220	CoreSet	7B	522.5	GGCATCGATATGCTGCTATTTG	TTTGTTTGGGATTCATTTGAGATAA
2827	tarc1231	Other	7B	528.8	CAAATGTACAGCAATAGTACATATTTCC	TAGTACCAATGGATGTTTTTTTCAGA
2828	tarc1249	CoreSet	7B	565.1	GTGTTTTCAACAGTGTATGATCAAGTC	GATCAGGCTCCGGGCTCC
2829	tarc1202	CoreSet	7B	566.5	GCATGACATTTATTTTGGATCGG	TTCAGGTTAGTATGCAAATGGAGG
2830	tarc1208	Other	7B	583.5	TCCTAGCAGCTCATAAAAAGCATT	AAGGAGCAGAGGGAGTTCTCAT
2831	tarc1232	Other	7B	587.9	GCCGGGATTCTCTTTTTTTGT	ACAAGCAAAACAGATAATTAAGCCTT
2832	tarc1256	CoreSet	7B	593.6	GGCATTTTGGACATTGACAGA	TTCCACTCGCTCCAGTTCATG
2833	tarc1218	CoreSet	7B	597.5	AGTTTGGACATAAGATTTTAACTATACC	GCGCCTAATCCCAAAAAACA
2834	tarc1235	Other	7B	601.1	TGCGGCAGGTCAAAGCTG	TTCTCACGGGGTGGTTTCAC
2835	tarc1236	Other	7B	602.1	AAATACCGTTCTTGATAGCAGGG	CATCAAGGCTTTTCCCCACA
2836	snp594	CoreSet	7B	603.3	AACAGACAGAGACACAGGAGGATAAA	TTTCCACTTAGTAACACATGGTACGA
2837	tarc2319	CoreSet	7B	620.7	GGGAATGTGGTTCATGAACTCTT	TTGGGGATACATACACATATGCTG
2838	tarc1225	Other	7B	626.1	CTTAGCGATGGAAGAACGAAATA	ACTAGTGATCAGTCATGCAGCGA
2839	tarc1233	CoreSet	7B	626.1	TGAATTAACCGTGTGATATTTGCA	ATCTACAAAGTTGCACATGGTTCA
2840	tarc2314	Other	7B	626.1	TCAACAAGATTGGAGCTCACTTG	GGCTGAGGGTCGACACGA
2841	tarc1259	CoreSet	7B	627.4	AATTACCAGTAGGCTTTCCACAGA	AGGAGAATCAACTGATTAAAGAGGAA
2842	tarc1241	Other	7B	628.0	TATACATTCTGATCCCATATCGCTT	ACATGGTCAGTTTGTCACCCC
2843	tarc1216	Other	7B	628.5	CCTGGTATTCAGCTTCAACGG	TTATTTTAGTTGCTTTCTTTCTTTCCA
2844	tarc2313	CoreSet	7B	629.6	ATCATATTCTTTTGTTCACCCAGC	AACACTGGCATCAAATGTTTCG
2845	tarc1226	Other	7B	632.7	GGTGTGGGGGTGTATAGCAGG	GATGGCATGTGCTGGGCG
2846	snp3928	Other	7B	645.1	CGATTAAAACATGTTGACTATGTTTG	GCGGACTCAACTAGCTGTACATATA
2847	snp6588	Other	7B	645.1	GACCAAAATATTCTATTGATGCAATC	GAAGGGCTGTCTCTGCTATGC
2848	tarc1242	Other	7B	647.0	TTTATATTGCCGTGCTCTTGACTT	CAGCGCCACGATATAGACACA
2849	tarc1211	Other	7B	653.0	TGTGAGATTTTCCTATCGTCATTTG	CATGCAGAGAGCTCACTAGCTTATG
2850	snp836	Other	7B	655.0	TACGAGGAACTGATGGCATACG	CGTCGTTCTGCTCTGAACTGTG
2851	snp320	CoreSet	7B	657.9	TGGACCCGACTCATCCAACA	TGCCCCGATGAACCTTACG
2852	snp394	Other	7B	657.9	CATCTCGTCTTTGGGGTTGATTA	CAAAGCATGAGCTAAATATGGTACTAC
2853	snp395	CoreSet	7B	657.9	GCAGTCCTTGCCTCTTTGAGTT	GCAGTTCGTTGTATGTGAAAATGT
2854	tarc2322	CoreSet	7B	659.2	TTTGTATTAGTGTCAATATTTATTTGCC	AGTCAAAAGAAATTAAAACATTGAGATA
2855	tarc1258	Other	7B	659.3	CAAGAACAACAATAATAAGGAAACGA	CTGGAACTTGCATTTCATTACGA
2856	tarc2318	Other	7B	659.7	CGAAAAGATGGCCTGCATTTA	TTCCGTCTTTTTGCTCCTCG
2857	tarc1229	Other	7B	670.3	TTGATCTAACAATGTGGTCTGTCG	TTTGCTATTTTAGTTTCTTGTGGATG
2858	tarc1228	CoreSet	7B	679.8	CAGTGTTTGCCTTTATTGCGT	CAAGTGGATGGTCTTTTTTATAGGTTA
2859	tarc1238	Other	7B	680.1	AGTTACTTGAAGCTGTAGCATAGCG	GCAAGAATGTGATATGATGATGTAAATG
2860	snp2191	CoreSet	7B	700.4	AGCGAGCAGTGCCAGTGC	GAGGCTTAGGTTTGTCGGCT
2861	snp4864	Other	7B	700.8	CCGAGGACGACGACTCTCTTC	TGTCTGTCATTCAATCTCAAACACAC
2862	tarc1260	Other	7B	700.8	AAGCCAAAAAGAATGTGTGCG	CGGTTGCTCAGATGGATCAA
2863	snp2193	Other	7B	701.2	GGCTCCATCGTCCATTTAGATT	ACTGCACGCCTGCATACG
2864	tarc1230	Other	7B	701.2	GAGAATCCTGCGGAGAGTGC	CAACAGAATAGAGAATCAACTGTTTAGG
2865	snp7780	Other	7B	706.8	AAGGACATAACAGAGTGTCGACCTT	ATGCTCGTTCTTCCCGCC
2866	tarc1203	Other	7B	706.9	CATTCCGCAGTACCTACACAGG	CCTTGGCGAGCTCCTCGA
2867	tarc2317	Other	7B	706.9	GCCTGTACTCTAGTGTCTAACCACG	AAAAAGTAACTGCAAAGTCATTGAGAG
2868	snp4593	CoreSet	7B	708.1	GAGAAACATGGTGAGATAGTTCTCTTAG	CTCTGCATCCATCACATCAACA
2869	tarc1209	Other	7B	708.1	GGGAGGCATAGAGCTTGTACCA	TTAACTCAAGCACAAAGCGAGATT
2870	tarc2320	CoreSet	7B	708.1	ATGAGGATAAGAGTTTGATGGCAG	AAAATGAGATGTTAAAAAGGAAAGAATA

Supplemental Table 4. List of amplicon sequencing primer sets for wheat.

No.	Name	Type	Chr	Pos[Mb]	F_primer	R_primer
2871	tarc2316	Other	7B	709.0	CCCACAAGAAATGGAGACGATG	CATCTGGGTGTCATCAAGGGTAT
2872	tarc1247	CoreSet	7B	711.4	AACTTGAAAATCTTGGATGAAAACA	CCTTGAGGCATGAGCACACA
2873	tarc2323	CoreSet	7B	711.4	AACATTTGTGAAATACATATGAAAAGG	CATCAAAGTGGAAATGGTAATTCCT
2874	tarc1221	Other	7B	716.3	GGGGATTCTCGTGCTGTTCT	ACCATAGCATCGAACTTCCACA
2875	snp180	Other	7B	730.2	TTTTCCTCTTTATGCCGACTTG	GCAAAAAAGCAGAGCAGAGGA
2876	snp182	CoreSet	7B	730.2	TCTCTTAACACCCTGCAACTATGTT	TTATGTAACCAGTGCTATGAACAGATG
2877	tarc1214	Other	7B	730.2	CCAGACAAGGATTTTCTCCTACG	TACATATTATTTCTGTCAATCGGGG
2878	tarc1222	Other	7B	730.2	GTGCTGCTTTGCGGACCA	GCAGTCTGAAGTGAATTTCCTCG
2879	tarc1322	CoreSet	7D	1.3	GGGTAAGAGTCTTTTATCATTTGAAGC	TTCATTTTTTGACTGCATGGACA
2880	tarc2503	CoreSet	7D	2.0	GATTCGTGGATTGGGTTCGA	ATTTGCTGGTTGCTGTTATGGA
2881	tarc0393	Other	7D	2.2	CCCATGCGGTGTTGACTACA	CACAACCAAGGTAAACGCTATCG
2882	tarc0373	Other	7D	4.1	GATGGTCAGAGCTTAGGCGG	CTCCTACACGATGAAGCAGAACT
2883	tarc0395	Other	7D	4.6	TCTAATACTCCATTGCAACTTGTGAT	AAGATGATGTCTAGTGTCTCTAAGCAA
2884	tarc0359	CoreSet	7D	5.2	ACAACCTGTGCCCGCTTG	TCTTCTCTTCTTTTGGTTCCACAG
2885	tarc0382	Other	7D	5.4	TAGCTGAACTTGCGAATCAGG	GTCCAAAGTGATCGTACAAAAGG
2886	tarc1461	Other	7D	5.4	CACCATAAATTCTAATGGCCTATCAT	CAAAAGAATAAGAAAAAGGGTAAACAA
2887	tarc0396	Other	7D	5.5	GCAGTTGTCCTGGTAAGACGCT	CCTCCTCCATCAGCAACACAT
2888	tarc0394	Other	7D	9.3	TGGAACATCCAGCCGCAA	ATAGTTCCAATGTTGACAGAAGTACGA
2889	snp8425	Other	7D	10.0	TGAATTTTTAATTTGGATCGATTTTA	CCCCTGTTACATCATTCAATTG
2890	tarc0362	Other	7D	10.0	GCAGTTATTAGTTGGTCCATCAGG	AAAAACAAGACATTGATAGAAAACCAG
2891	tarc0384	CoreSet	7D	10.7	TGTTCAGTGACTCCACCCTGC	ACATCAACCCGGTTATATAATGTT
2892	tarc1525	CoreSet	7D	13.3	AGAACTCTGCCATATTCCATTGTC	TGAGATAAAATGTCTGCTCAGAGATAGT
2893	tarc0366	Other	7D	23.5	TTATGTAGCCATTAAGGTCAGCC	GCGCACAGAGGTACAATTTCAA
2894	tarc1406	CoreSet	7D	25.4	GAAGCAGCAGTTTCAAGTCCG	AAGAGGGAAAACTTTTAATAATCTACGA
2895	tarc0371	Other	7D	29.9	GCAGTGCACCACCAGACAAG	AGGAATCATCAAGCGAGTCATATA
2896	tarc1790	Other	7D	30.3	CCTTCGGTATGATCAACAAAGCT	TTTTATTCTGTAGTATCTGCATCTCCC
2897	tarc1421	CoreSet	7D	32.9	TAAGTTCTGTTGGTGGTCATCTTTC	GGATCTCCCCGACCAAAAT
2898	tarc1313	CoreSet	7D	34.4	AGTCATTTGGACGTCTGAGCTG	GCCGACGAAAAAAATCAAAATA
2899	tarc1433	CoreSet	7D	41.1	CTTAGCGGTGACTAAGGTGATGA	AAAAACAATCCAGCAGAAAACG
2900	tarc0377	Other	7D	42.6	GCATCTCCAAACTGTAAAACCGA	GTTGCCTTTGATTTCGCAAG
2901	tarc1331	Other	7D	42.7	ACATTTTGGTTTATCTCTCTTTGTACG	AAATTTGGGTCTTGAGGGAAGA
2902	tarc1449	CoreSet	7D	51.8	CCTGTTTAGATCACCATTTTCCGT	GGGTTGTACTCGTTCAGGTGTTG
2903	Inf43076	Other	7D	63.7	TGTTTGTTAAGATTATTCAGCTCTTGTT	CAAAATGTTCCAGTGAAGTTAGCTC
2904	tarc0386	Other	7D	63.9	GTCTACAACCACACTGCCATACC	TTTGCTTCGATTTCAGTCTACTACG
2905	tarc0372	CoreSet	7D	64.7	GGTGGAGCTTCATTAAAGATGATG	TAGAGCCGCAGAAACGAGAAG
2906	tarc2508	Other	7D	64.7	TTATTGGTAGACACTGCTTGTCCTATT	TCAGAATGTCGTGTCTTGATGCT
2907	tarc2506	Other	7D	66.6	CAGGTATGCAAACTTCTGTGACATC	TACACCTTAATCAACAACTCGATGAT
2908	tarc0381	Other	7D	71.2	GTTGGTCGAACAGGAAGCG	AAGATGACAAAAAGGCACATCAT
2909	tarc0392	CoreSet	7D	71.6	CGTACAGTATGTTATGCACTGGC	AATGTGCTCGTTGGTGAAACTG
2910	tarc0388	Other	7D	72.9	GCAGTTGTGACAGCAGATCGAA	CATTTACAATTCCTGCATACCGA
2911	Inf16986	Other	7D	72.9	CCATCCAGGTTTGAACCTTGTG	CCCAAGACATTGCAAAAAGATAAA
2912	tarc0389	Other	7D	73.4	TGGATTTTTGTTATCACCACCAGT	GTCGTGGAAAATGGAAGGAGA
2913	tarc0387	Other	7D	74.4	CGACGATGATGCGCCTACAC	AAGAAACGGATGTGTTGAGTGAGT
2914	Inf5396	Other	7D	77.4	AATCTTGTTGGGAAGAAGATCGA	GGGGACACTACTTAGCAGCAGC
2915	tarc0368	Other	7D	82.7	GCGGTTTCAACCGGATAATAAT	CAACCTAGCATCTATTGCTTTGC
2916	tarc0378	CoreSet	7D	84.3	ACAACTTGTAGAACAGAGCCTCG	TCTTGTTGTGGTTTTGCTTGCT
2917	Inf15383	Other	7D	85.0	CATATTTACCGTTCGGTCGCA	ATCTCGAGTCCGGGCTAATTT
2918	tarc0380	Other	7D	88.2	TGTGGTGGACTTGGTATGTTTGA	TTGGGTCACCTGACATTCGA
2919	tarc1378	Other	7D	89.5	GAAGGAAATAGAGAAAGACCTTATGC	AGCCCTCCCATCCCCAAC
2920	tarc0390	Other	7D	89.6	ATCATTGAATGCCAAACCACTTA	CTTGCCAGGTAACGAGCTATGT
2921	tarc1356	CoreSet	7D	90.6	CGGTTTTACAAGTTTTTTTTTGTTTACT	GGGGACAAACTACACCAGGCT
2922	Inf18158	Other	7D	90.9	GCACCAGAGCGGAAAGCAG	TCTTCGTCTTCCTTGGGTCAA
2923	tarc1542	Other	7D	91.1	CGCTACTTCTCCACGCCTTC	CATCTATCACAGCATAGCAGCAG
2924	Inf42408	Other	7D	101.6	GAGGAAAACTGCAGATGCCA	ATTCGAATCATCACACGGCTTA
2925	tarc0370	CoreSet	7D	104.6	AAGTAAAAACACCAAAGTGAAATCAGA	GGTTTGGGTCATTTAGTGTTGCT
2926	tarc0361	Other	7D	104.9	ATCTGATTACAGTATAGTGAAGTCACCG	AAGGTTCGTAACAACTTAACAAGTCC
2927	tarc0383	Other	7D	109.3	CCACTAACAATGGATCTACAACAGT	CGCTGAAGCAACATACCACATG
2928	tarc2500	Other	7D	110.6	TACTAGGAACTCATGTGAACAATTCTTT	TAGATAGCATGCATCACATATTTTAGG
2929	Inf17173	Other	7D	111.6	TTAGTTTTTCTAGCCATGCTGGTC	TCCACCCTTGCTGGTGATCT
2930	tarc0369	Other	7D	113.2	CAGATGAGTATTACCGACTGCGT	CTGTTCGGAAACTTGCATCAC
2931	tarc0367	Other	7D	113.4	ATTCCATATTCATTGGCAAATCA	GTTATCTGATGTTGTAGGGGGC
2932	tarc0385	Other	7D	117.8	TACAATCGTTACCCGTTACCTGT	GCTCGGACAAATCCTCCTG
2933	tarc0374	CoreSet	7D	123.0	AGCGTAGCACAACGACCAGA	ATACACCTTAATCTAAATGGCACTACA
2934	tarc1415	Other	7D	127.0	TGTTCGTTTTGTTTACCACCAGA	ATTGATTGTGAACGAGGAAGGAT
2935	tarc0365	CoreSet	7D	153.6	CATCCACAATTAGTTTCTCATTTAGTT	CTTATCTGAAACACTTTTGTGAATGA
2936	tarc0376	CoreSet	7D	159.4	ATTGGGTTTTTGCCCTTAGGA	TTGAGTGACTTCTGATTTAGTCAATTC
2937	tarc0391	CoreSet	7D	164.8	AGCTTTTCACCAGGGATGACG	CAGACTAAAGAATACGCAGATGGA
2938	tarc1456	Other	7D	174.2	TCCCATACCATGCTGAAGTCAT	GAAACCTGTGAAAGCAAAGGAAT
2939	tarc0375	CoreSet	7D	184.9	TCTATCTTGTGATGCACGTCTGC	TTCCTAAGTGTAATGACTTCCGTG
2940	tarc1546	Other	7D	199.6	CATCCGTCGCTTACAATACCG	AAAAAAAAATCTGAAAATCATCTAAATA

Supplemental Table 4. List of amplicon sequencing primer sets for wheat.

No.	Name	Type	Chr	Pos[Mb]	F_primer	R_primer
2941	tarc1404	Other	7D	210.4	CTATAAGGGCGTCCAATAGTGTTT	AGCATTACGAATGTGTGATACACAAC
2942	tarc2502	Other	7D	224.5	GGATTCAGGTGAAAAATATAGCCA	ATACCAAAAAACAAAATCGATGGTAT
2943	tarc0364	Other	7D	236.8	GCTCTAACTCTGAACAAGAGAGGG	ATTTGGCAAGATGTTTCTCAACG
2944	tarc2504	Other	7D	263.8	ACGGCGTTCTGCACTGCG	CGTGCCTGACTACACATACCATTA
2945	tarc2507	Other	7D	263.8	CCACACGTTCCACATGTCGT	CGAACTTCCCGTATGTGCGT
2946	snp2772	CoreSet	7D	265.7	CTTTGACAGCTGCCGGTAGAA	AACCAAAACAACAGGTGAAAAGA
2947	tarc2501	Other	7D	266.4	CTACCTGACAATTTGGTATGTTTTCTTA	TGAGTAGTGAAATTTTCCACAGTTTTAG
2948	tarc2505	Other	7D	270.7	GAGATTTGTAATACCACCGTGGAG	GGTCAGCGACGCCATCTC
2949	tarc0360	Other	7D	280.6	AGTAAAGCTCTATTGGGTTGTACCAG	GGCAAATGATAAACCTGAAACG
2950	tarc0363	Other	7D	317.4	GGAATCGACCAATGATCTACGAC	AGCTTAAGGAAGTAAAAAATATGCG
2951	snp2208	Other	7D	370.2	GGTGCGTTGTCCACTGGGA	CCACTGCAACCAAGATGCCT
2952	tarc0334	Other	7D	372.9	GTAATTGTCATAATTGTAGCTTTGATTG	CACACACATTCAACTATGGACTGAC
2953	tarc0350	CoreSet	7D	378.5	CGAAGACAATACCTTCCACAAGCT	TGAGTTCTGTTGACAATTTGACATACTAG
2954	tarc1273	CoreSet	7D	387.9	CCAGTAGTGCAATACCTACATAACAGA	CCGAACTAAATGCAGCTCATG
2955	tarc0342	Other	7D	391.6	CCTTTCCTACTAATGTTCTCCGC	GGCTGAACTAAACAGGGCGA
2956	tarc0351	Other	7D	392.6	AGTGCAGTATACATATAGCTTTCAAATCA	CATCGTTTCCAACTGATATGGC
2957	tarc2498	Other	7D	396.5	TCAGAAATACACGTACCCAGAAGTATC	GCTGTACCTCCACTACAACGACC
2958	tarc0358	Other	7D	397.4	TTAGGACTCACTTGTTTGCTGATATG	TTTCAGATTATTCATTCCTATTCCG
2959	tarc2496	Other	7D	406.5	GCTTGGATCTGCAAGATCTCAA	GAGGCCGATCCCTTTAGCTC
2960	tarc1526	Other	7D	424.1	GCATCTTGTGCTGGATTTGTGAT	CGGTTACAACCCACTTAAACATTG
2961	tarc0345	Other	7D	428.0	GGAATTGAGAGGTACAATTTGTGTCTA	CAGCACAAGCCATAGCTTCTTTT
2962	tarc1434	Other	7D	444.4	GAGTTGACGAAACAATTCCTACG	TGATGACGCAGGTCGCCG
2963	snp1537	Other	7D	451.1	ATAAGATTAACCAGTGGATCGACC	AGTGGGAGCTCTGAGTTCCAGT
2964	tarc0353	CoreSet	7D	451.2	CCAAAAATCGTCTAGCCATCAATAT	CAAGAAGTAGCACTGGCAGGTC
2965	tarc0347	CoreSet	7D	458.5	ATCTGGAGGTATGGTGCTTTAGC	CTAGAGGAAACTTTGATGTTTTATCTCA
2966	tarc0343	Other	7D	488.8	TGATGGCCTTTTGTGCCTTAA	GAAGCTAGTACAATTGAAAAGAACACG
2967	tarc2491	CoreSet	7D	495.9	TCCAGTACAAATGTTATGTTTGTTTATA	CCCGGCATAGCAGGAATCA
2968	tarc0352	Other	7D	498.0	ATCCAGCGAATCTATAAAAACGA	TTCATTTTGCTGTAAGGAAGAGGA
2969	tarc1262	Other	7D	498.3	ATGGCCCTTTAGTTTGTAGGAATAG	AAAAGCCGTGGTAGGTTTTATGA
2970	snp2273	CoreSet	7D	501.0	GGCCACAACGACCACAGTG	TGTGAACCGGCAAGATATGATC
2971	tarc1530	Other	7D	501.0	GGGAGCGATGAAGAGGGATG	CGACTTCGGCCAAGAGGG
2972	tarc2497	Other	7D	518.8	CATTAATGCGAGCCCACATC	TCCTTCCCGTCGGCCATG
2973	snp1902	Other	7D	530.0	TGAGGTGAAAAGCTGTCTTCTCA	GCCAACTTATATTGTGAGACGGAG
2974	tarc1272	CoreSet	7D	530.1	AAGAACTTGGAGGGTGCGAA	GTGGCCGTCACGTTTTTCT
2975	tarc1502	Other	7D	530.7	TGTCATCGGACCACATTTTTTG	CACGCAGTCTCAAGGCACC
2976	tarc1339	Other	7D	531.1	GCTCCTAACCAGTTTTTCTTTTCC	CTCCTTCTGTTTTCGTTTTTTTGA
2977	tarc0339	CoreSet	7D	531.9	ACAGGCTGATCTACTTCATGCTTATA	CTATTCTTTTAATTATATTTCTGGGACG
2978	tarc0331	Other	7D	535.3	ACTCAGGAGTGCTTGGATCGA	CAAGACAGCAAAGGATTAAGATGG
2979	tarc0338	CoreSet	7D	549.5	TGTCGCCACACTTTGCTTCA	AACAACCGTATCAGCAGAAATGA
2980	tarc0336	Other	7D	550.3	ACATCCATTGTACAGAGGAATTGC	GTACAGGCTGCCATGCAAGTT
2981	tarc0346	Other	7D	552.5	AAATCAGACTCTGAACATTTCATAGACTA	GACTTAGTATTTCCATTCATGTTATCAAT
2982	tarc0335	Other	7D	554.6	TTTTATTCCAGGAAAATTACAACAAT	ATCCCATGGTTCAAGATCAGC
2983	tarc2492	Other	7D	554.9	CCCCGAAACCGGAAGAGA	CCACGCCCATTTCCTCAC
2984	tarc1482	Other	7D	560.7	CCATCCTCTTACCAGAGAATTTTGT	ATGCTTTCATTACCATGAATAAAGATAT
2985	tarc1303	Other	7D	560.8	TCACGTGCAGGCTGGCAG	CTCCCTTGAGCCCGCTGG
2986	tarc1293	Other	7D	561.7	GCCAGGAACACAAAAATGACAA	TGGTGGCACATGGACGGG
2987	tarc1457	CoreSet	7D	568.9	TTGATGTGTTTTTTTCTCCACG	GAACAGCATGCAAAATGAAGTGA
2988	tarc1462	Other	7D	569.0	CTCTGTCTCTGTCATGCTGTGTGT	AGATTCAGGTGTGCGTGCG
2989	tarc1301	CoreSet	7D	573.8	GTTGCAGATCGTTATTGCTTTCA	CTTGCTTTATATCATCCAGACCGT
2990	tarc1479	Other	7D	579.8	GGAACCATAGCAGTTCTGGACG	GACACTTGCCAGCATTGGAG
2991	tarc1458	Other	7D	583.1	GCAATCTTAGTACTCCCTCCATTCTC	TATCAAAAATACATTTTGCATCAATATA
2992	tarc2494	CoreSet	7D	587.8	CAAACATGGCTTCTGTGTCACAA	GGGTATGGGCAATAATTTCGTC
2993	Inf18911	Other	7D	588.5	GAAGAGCCTGCCATACATTGA	AAGTGCATTCATATTCTTTAAAACCC
2994	tarc1291	Other	7D	596.9	TCCAAACACTTCCAGACAAGTTTC	AGCTCATTGTCCATCGCATTAG
2995	Inf40472	Other	7D	596.9	TTGCGGTTCTCTGGTGCAT	TCCAAACACTTCCAGACAAGTTTC
2996	tarc0349	CoreSet	7D	603.4	CAGATCTTGTAGTGATTTGAGAATCG	GCCAGTGGTGAAACACAGCTA
2997	tarc1488	Other	7D	605.7	GGCCAACCACTCGATGTCAG	GCACCAGCAGCAGCAGCG
2998	tarc2490	CoreSet	7D	607.0	CCGGCTCTTCGCTTCCTC	TCAGATGGCATGGGCTTTG
2999	tarc1544	Other	7D	611.2	ATAAACAGTGAGCCGTCCTAAGAAT	ATGCAGAATTGGAGATGATGGTA
3000	tarc0348	Other	7D	611.6	AAAGATCTACAAGTACATCTGAGACAATG	TGCTATTCAGTATCTCGGTTTCTTCT
3001	Inf6338	Other	7D	611.8	TCAGGCGGTACCCGCTGC	AGGGGACGATCACACACGA
3002	tarc1264	Other	7D	612.9	GAGCTCAGCAAGATCGCCAT	ATGAGGTGATCAAGAAAGCCG
3003	tarc1396	CoreSet	7D	614.5	GAGGGAAGGTAAGCAGTACTCAAAA	TTATGCTATCACTAAATTTTATCACTGG
3004	tarc0356	Other	7D	617.1	CAGCTACTCGGAACTTTGATAAGG	TGTACGAATTTCAGTACATCCATTTTT
3005	tarc1527	Other	7D	618.0	ATGGTCAGATCCCTGACACGA	TCTCCACTCCATGTTGGTTCCT
3006	Inf39519	Other	7D	618.3	TAATAACAATCAGAAACTTGATCTCACT	CTCAGCAGTAGCTCACTCAAGCTAC
3007	tarc0357	Other	7D	619.4	TGTATGTCTAAAGTTCAGAAAGAGCTAAAC	AGAATGTTATGGATGGGAAAAATG
3008	tarc2499	Other	7D	619.4	CTCTTTTTGTATTCATTATACTGTGGG	TAGGGCTCAACTAGAAACAGGATG
3009	tarc0341	Other	7D	619.6	AACCTGATACCCGCTGGAAC	TTACGGAGATTGTAGGATTCACAGT
3010	tarc0340	Other	7D	619.9	TGACGTGCGTAAACAGAGGC	GTTGCCGGTACAATTTATTGTTT

Supplemental Table 4. List of amplicon sequencing primer sets for wheat.

No.	Name	Type	Chr	Pos[Mb]	F_primer	R_primer
3011	tarc1531	Other	7D	620.5	CGACCCATAAGGTGCGAGTG	CTTGTTTTTGCTAAGCCCTCTG
3012	tarc2493	CoreSet	7D	620.5	CAAAGTCTTCATGATGGGGATTAA	GCCCTGCTGTTTCGCTGA
3013	tarc0355	Other	7D	621.0	CCTTTCGCTGCTTCAGCTTTAC	GAAGTGGAGTCAAGATGCTTAGTGA
3014	tarc0344	Other	7D	621.5	GTGGCTATTCAACCTTTTTCCAT	GTGGTAATATGCTGTTGACATCGA
3015	tarc1422	Other	7D	621.5	CATGCGACCAACCCTCCA	GAAGAAACTCACAAACAAGAATTATTCA
3016	tarc0332	Other	7D	621.6	ATGTAACTGTTGGCAAGATAAGTATG	TCACATATACTTTCATTGTAGATTATGC
3017	tarc0333	Other	7D	623.5	TCATCGCTCGCATGAGCAC	CGGGTTTGATCACTAAAAACCA
3018	tarc1292	Other	7D	625.4	TTTTGATGAAGAGCTGACATTGGT	CACCTGCGGCTTCAACAATA
3019	tarc1370	Other	7D	625.5	CTAGGTGTCATCTTTTTACGCTTATTT	CACATAAATGACTGTTTCCTTTTGTAA
3020	Inf4994	Other	7D	627.1	CCATCATTCCTCGAGGTCAGG	CACATTGTCTTGGCCAAAGATAA
3021	tarc1353	Other	7D	627.3	GCTAAAATCCTGTGCTCGTGTT	CATCTCTCCTCTTGTGAGCTTTCT
3022	tarc1285	Other	7D	627.4	GCCCTCATGGCAAATTCG	GCGTTGCATGTTGACTTAGTAAGT
3023	tarc1493	Other	7D	627.4	AAGTTCTTAGAAACCCCAATGTTCT	TCGAATCGACGAAAGGTGTATG
3024	Inf11097	Other	7D	629.8	ACCACGACAGAAGCAACACG	AAATGTCTTGCCCTTCCTTCC
3025	tarc2495	CoreSet	7D	630.2	AGAAAATATAAATGCCCTGCAACTAT	AATCGGAAAAAAATGATGTATGGA
3026	tarc1279	Other	7D	630.5	CACAGAAGCTCGGCACTGG	TAGATCCTCGGCTGGCTCC
3027	tarc1304	CoreSet	7D	631.8	TGCCAGTTTCTATCTATAGCATAATGAT	TGTCCACTGCCTTTAACCCAC
3028	tarc1381	Other	7D	632.0	GAAAAATACAATACAATACAATGCTGG	TCGGCGACTAATGGAACACA
3029	Inf44953	Other	7D	633.3	CCTCCTCCATGGAGAGCTGA	TGTCGGCGAGCAGTTGGC
3030	snp4679	CoreSet	Un	2.6	AACTGCAAGGTTCCGTCTGAGT	CAGGATCACAGGACTTGCAGAT
3031	tarc0074	CoreSet	Un	10.2	TCTTAACGATGGAAAAGAGATGC	GACCCACAGAAATTAAGCCTCC
3032	tarc2073	Other	Un	15.9	TAACTCTGTTATTTGGAAAGTTGCC	GAAAAGAATAGATTGGCTCCACAA
3033	tarc2053	Other	Un	23.3	GATCACTCTCAAATGGTTTTCCG	TCTATTGGCTGCTGCAATGGT
3034	tarc0911	Other	Un	23.7	AAAGAATAGGCGACCATGTGG	TGTTTGGTGATTCTGAAGTCTGTG
3035	tarc0017	CoreSet	Un	29.2	CACTCCTGTCCTCAGTTGCCAT	GAACTACAAAATTGGAGTGGAATGA
3036	tarc0007	Other	Un	29.5	TGCATGGAGGCCGAGAGC	CAACCATCTGAATCAATTCCTGTC
3037	tarc1733	Other	Un	32.9	GTTGGATTTACTAACAATAAAATGCGT	CGTGATAAGATTATTGCTATTGGAAA
3038	tarc0948	CoreSet	Un	35.3	CAAATCAGCAGCAGCATCCC	GAGGGAGCTTTTACCCTGTGA
3039	tarc2131	Other	Un	39.2	GCTTTCCAGATTTTCCAGGCT	GGATAAAAGGCTTCGTAAGTAAGTGT
3040	tarc2582	Other	Un	42.2	TAGACCAAGCGAACATTCTCCA	GAATTCAGCCTCTGTATTCTAACCT
3041	tarc1113	CoreSet	Un	65.4	GGGGCAAGGTAGGGTTGG	GTCAAAAACAAAATGTTATGTGATCG
3042	tarc0283	Other	Un	67.7	TCGCTCGAGAAGCTGACACA	TGTTTCCTCATATACAAATTTTCTTCAT
3043	tarc0705	Other	Un	71.3	GGGCAACAGTAGTGGATTCAGT	CAGCCTCAATGGATTTTCTTTATTAT
3044	tarc1896	Other	Un	71.3	CCGTAATGGGTTAAAAGGAAATAAT	CCTGTCCTACTGCTTAATGCGC
3045	tarc0706	Other	Un	74.6	CCCTAATCAGAAAGAGAGAAAGCA	GATGTGATTTTGTTTGGTGTAGACG
3046	tarc2315	Other	Un	89.0	GCAAAGGATTAGCGGCTGAAC	AAGCCACCTCCACCACCTG
3047	tarc0379	Other	Un	91.5	ACAACCCAACCAGGCTTAGACA	TGGCTCTTCTCTTGAATTAACATAGTA
3048	tarc0354	CoreSet	Un	92.7	GACAAAAGGAGAACTGCTTCGG	TCTATTTATTGCTAATTCTGGAACTGG
3049	tarc0337	Other	Un	93.1	CATCTACGAGAAGCAGTTATCTGTCT	ACTGCAGCATATAACATCCCACT
3050	tarc1410	CoreSet	Un	127.0	AAATATATGAAAGATGCAAGTGTTTTGT	AAAAGGCATTTCAATTCCAGACA
3051	tarc0459	CoreSet	Un	249.6	CAAGCCCTGCACCTCCCC	TGCGATGAACACCATGAAACTAA
3052	tarc2321	Other	Un	277.7	TGTTAACTTGGATACTTCATTTCCTACA	TGATATGCACGACGACAGCG
3053	tarc1187	Other	Un	290.2	ACAACCTACATTGTCTAACATTTATCTT	AAAATCTCTTCTCAGGGTTGCC
